# Diagnostic and Clinical Performance of Single‐Use Versus Reusable Gastrointestinal Endoscopes: A Systematic Review and Meta‐Analyses

**DOI:** 10.1111/jgh.70637

**Published:** 2026-08-07

**Authors:** Natalie Tyldesley‐Marshall, Yen‐Fu Chen, Jefferson Buendia, Anna Brown, Thomas Matthews, Janette Parr, Shivashri Chockalingam, Adel Elfeky, Amy Grove, Lazaros Andronis, Stuart R. Coles, Mandana Zanganeh, Bu Hayee, Anjan Dhar, Shaji Sebastian, Ramesh Arasaradnam, the SUMU‐Endo Project

**Affiliations:** ^1^ Centre for Evidence and Implementation Science University of Birmingham Birmingham UK; ^2^ Department of Health and Welfare University of Taipei Taipei Taiwan; ^3^ Department of Pharmacology and Toxicology University of Antioquia Medellin Colombia; ^4^ Evidence Synthesis Ireland University of Galway Galway Republic of Ireland; ^5^ Centre for Health Economics at Warwick University of Warwick Coventry UK; ^6^ Warwick Manufacturing Group University of Warwick Coventry UK; ^7^ Department of Gastroenterology King's College Hospital NHS Foundation Trust London UK; ^8^ Department of Gastroenterology County Durham and Darlington NHS Foundation Trust Darlington UK; ^9^ Inflammatory Bowel Disease Unit Hull University Teaching Hospitals Hull UK; ^10^ Institute of Precision Diagnostics for Translational Medicine University Hospital of Coventry & Warwickshire NHS Trust Coventry UK

**Keywords:** comparative effectiveness, duodenoscopes, lower gastrointestinal, risk of bias, upper gastrointestinal

## Abstract

**Background:**

Endoscopic procedures are fundamental in the diagnosis and management of gastrointestinal disorders. However, concerns over pathogen transmission have driven the development of single‐use and partially single‐use endoscopes (reusable endoscopes with disposable components). A comprehensive synthesis of comparative evidence is essential to inform decisions regarding clinical adoption.

**Methods:**

We systematically searched MEDLINE, EMBASE, Web of Science, Cochrane Library, and trial registries for randomized controlled trials, non‐randomized comparative studies, diagnostic accuracy studies, and case series. Outcomes assessed included technical performance, diagnostic accuracy, infection risk, and adverse events. We also included systematic reviews estimating infection risk associated with exogenous pathogens for reusable gastrointestinal endoscopes. Summary data were pooled using a random‐effects model where appropriate, and other findings synthesized narratively. The protocol was registered prospectively on PROSPERO (CRD42024496913).

**Results:**

Of 6979 records screened, 50 studies (965 960 patients) were included. Our meta‐analyses demonstrated that in endoscopic retrograde cholangiopancreatography (ERCP), crossover from single‐use (EXALT Model D) to reusable duodenoscopes occurred in 7% of cases (95% CI 6–9). Across endoscope types, single‐use and partially single‐use devices demonstrated similar or slightly reduced technical performance and diagnostic accuracy compared with reusable counterparts. Estimated infection risks from contaminated reusable endoscopes ranged from 0.01% to 0.8%, although these figures are limited by high risk of detection and reporting bias.

**Conclusions:**

The current evidence does not support wholesale replacement of reusable gastrointestinal endoscopes with single‐use or partially single‐use models. More robust and standardized data are needed to quantify infection risks from reusable endoscopes and inform evidence‐based policy and procurement decisions.

## Graphical abstract

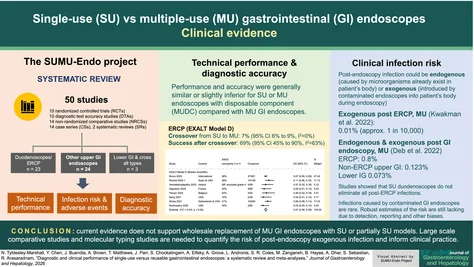

**Abbreviations**
DTA: diagnostic test accuracy
ERCP: endoscopic retrograde cholangiopancreatography
GI: gastrointestinal
IQR: interquartile range
MU: multiple‐use
MUDC: multiple‐use endoscopes with disposable components
NR: not reported
PROM: patient‐reported outcome measure
RCT: randomized controlled trial
SD: standard deviation
SU: single‐use

## 1 Introduction

Traditionally, endoscopes are used repeatedly following reprocessing after each use, but concerns have been raised that reprocessing may not be as effective as previously thought, leading to reported outbreaks of transmission of infections between patients [1]. Single‐use (SU) endoscopes are an attractive alternative for infection control [2, 3], due to the potential to eliminate the risk of cross‐patient transmission of pathogens. However, SU endoscopes have the disadvantage of an increased environmental impact associated with creating and disposing of large amounts of plastic compared to multiple‐use (MU) endoscopes. On the other hand, MU endoscopes require resource‐intensive procedures and well‐trained personnel for reprocessing, special space and equipment for storage as well as repair. Therefore, the better choice between SU and MU endoscopes is unclear.

Partially SU endoscopes, that is, MU endoscopes with disposable components, such as MU duodenoscopes with a disposable cap (which we refer to as MUDC in this paper for brevity), and MU endoscopes covered by a disposable, protective sheath (referred to as sheathed MU endoscopes) have emerged as further options. Concerns have been raised about the clinical performance of SU or partially SU endoscopes, as well as their potential cost‐effectiveness compared to MU options [4, 5, 6].

Gastroenterology is the largest user of endoscopes with over 22 million procedures conducted annually in the United States [7], and therefore, decisions concerning SU versus MU endoscopes used in gastrointestinal (GI) tracts could have major clinical, economic, and environmental impacts.

Robust evidence is needed to support healthcare decision‐makers and practitioners to decide whether, and under what circumstances, SU or partially SU endoscopes may be preferred over MU endoscopes. Prior to determining wider economic and environmental impacts, novel SU and partially SU endoscopes need to demonstrate their clinical performance and safety for patients. Although a few systematic reviews have examined specific types of SU or partially SU GI endoscopes [8, 9, 10], no prior systematic reviews have comprehensively evaluated these factors across different types of GI endoscopes. There is an urgent need for evidence to inform decisions regarding the adoption of these novel endoscopes in clinical practice globally [11]. In this review, we therefore aimed to investigate how SU and partially SU GI endoscopes compare to MU GI endoscopes with regard to technical performance, diagnostic accuracy, infection risk, and adverse events.

## 2 Methods

For this systematic review and meta‐analysis, PRISMA guidelines were followed [12], and a completed PRISMA checklist is available (Table S1). The protocol was prospectively registered (PROSPERO CRD42024496913), and deviations are reported (Data S1).

### 2.1 Search Strategy and Study Selection

In July 2023, the Cochrane Library, EMBASE, MEDLINE, Web of Science, and trial registries were searched from inception to date of search. Searches were updated in March and September 26, 2024. The search strategy, designed by an information specialist (A.B.), used index terms and text words related to GI endoscopes/endoscopy combined with terms related to single‐use, disposable, reusable, and multiple‐use, with no language restrictions. Additional searches were undertaken to identify systematic reviews on clinical infection risks for GI endoscopes (Data S2). Reference lists of systematic reviews were checked for additional citations.

The study Advisory Group, including gastroenterologists and health technology assessment experts, advised on inclusion/exclusion criteria. Studies evaluating the use of SU endoscopes that aimed to replace functionally similar MU endoscopes for diagnostic or interventional endoscopic procedures of the GI tract were included. Studies of SU devices that were functionally different from MU GI endoscopes were excluded, such as sponge capsule devices that did not allow visualization of the GI tract; endoscopic equipment used as part of laparoscopic procedures; and other non‐endoscopic health technologies or screening/diagnostic tests. We excluded studies evaluating solely SU cholangioscopes such as SpyGlass (Boston Scientific, Massachusetts, USA) as they tend to be used when there are specific clinical needs and are not seen as a substitute for similar MU counterparts. SU robotic endoscopes were also excluded as they were primarily used only in research settings.

We included randomized controlled trials (RCTs), diagnostic test accuracy (DTA) studies, non‐randomized comparison studies, and case series, if the case series included ≥ 40 patients/procedures and reported details of SU or partially SU endoscopes. We additionally included studies (including systematic reviews) with data only from MU endoscopes if they provided an estimation of infection risk associated with exogenous pathogen transmitted by contaminated MU endoscopes, or allowed this to be calculated for comparison. Simulation studies were excluded, as were studies not published in English or without an English version available. Publications preceding 2010 were excluded to maintain relevance to current clinical practice. Documents without empirical evidence such as clinical guidelines, position statements, narrative reviews, and commentaries were excluded.

Eligible studies had to recruit either patients undergoing diagnostic or interventional procedures of the GI tract and their family/carers, or healthcare staff who are involved in the preparation, operation, cleaning, reprocessing, and disposal of endoscopes. Animal studies were excluded.

Eligible studies had to assess the technical performance, diagnostic accuracy, clinical effectiveness, infection risk, and/or adverse events of SU or partially SU endoscopes. Records retrieved from searches were imported into EndNote (version 21), where duplicate records were removed. Two authors independently screened (N.T.‐M, Y.‐F.C., J.B., T.M., S.C., A.E., J.P.) the title and abstract of the remaining records in Covidence against pre‐specified eligibility criteria. Discrepancies were resolved by discussion and consulting a third author. Potentially relevant records were tagged for key study characteristics, and full texts obtained for further screening. Full‐text articles were assessed by two authors (N.T.‐M, Y.‐F.C., J.B., T.M., S.C., J.P.) independently against the eligibility criteria, and reasons for exclusion were documented (Table S2). Discrepancies were resolved by discussion and consulting a third author. Where required information for determining eligibility was not available, original investigators were contacted, and if no reply, the study was excluded.

### 2.2 Data Extraction and Risk of Bias Assessment

Data were extracted by two authors (N.T., Y.‐F.C., J.B., T.M., S.C., J.P.) independently using forms designed in Covidence. Discrepancies were resolved by discussion and consulting a third author [13, 14]. Data items extracted included first author/year, country, funding, aims/purposes (including comparisons made), device model/s, characteristics of study (details of participants/procedures/endoscopes, study design, setting), outcomes, and authors' conclusions.

Quality assessment was undertaken by two authors (N.T., Y.‐F.C., J.B., T.M., S.C., J.P.) independently. Discrepancies were resolved by discussion and consulting a third author. Quality assessment was appropriate to design using the National Institutes of Health Quality Assessment Tools for systematic reviews and case series [15], Cochrane Risk of Bias 2.0 Tool for RCTs [13], ROBINS‐I for non‐randomized comparative studies [16], and QUADAS‐2 for DTA studies [17].

### 2.3 Data Analysis

Studies were categorized by broad endoscope type (duodenoscopes, other upper GI endoscopes, and endoscopes used for lower GI tract), and then grouped by specific endoscope models. Comparative data from fully paired DTA studies and RCTs were prioritized over non‐comparative data, with the latter mainly presented in the appendix. Data from full texts were used for the main analysis, and additional data from abstracts were included in the sensitivity analysis. All statistical analyses were performed using Stata (Version 18). The designs, patient populations, and outcomes reported in studies evaluating SU and partially SU GI endoscopes were diverse. Where data were available, we pooled proportions or risk ratios using a random effects model for studies of the same endoscope model. We assessed heterogeneity using both the *χ* ^2^ test and the *I* ^2^ statistic [13]. Where data were insufficient or too heterogeneous for meta‐analysis, a narrative synthesis including summaries of findings, summary tables, and forest plots (for visual presentation without pooling) was conducted. We did not use funnel plots and related methods to assess potential small study effects/publication bias because the numbers of studies included in individual meta‐analyses were too small.

### 2.4 Role of the Funding Source

The funder of the study had no role in study design; in the collection, analysis, and interpretation of data; in the writing of the report; and in the decision to submit the paper for publication.

## 3 Results

**FIGURE 1:**
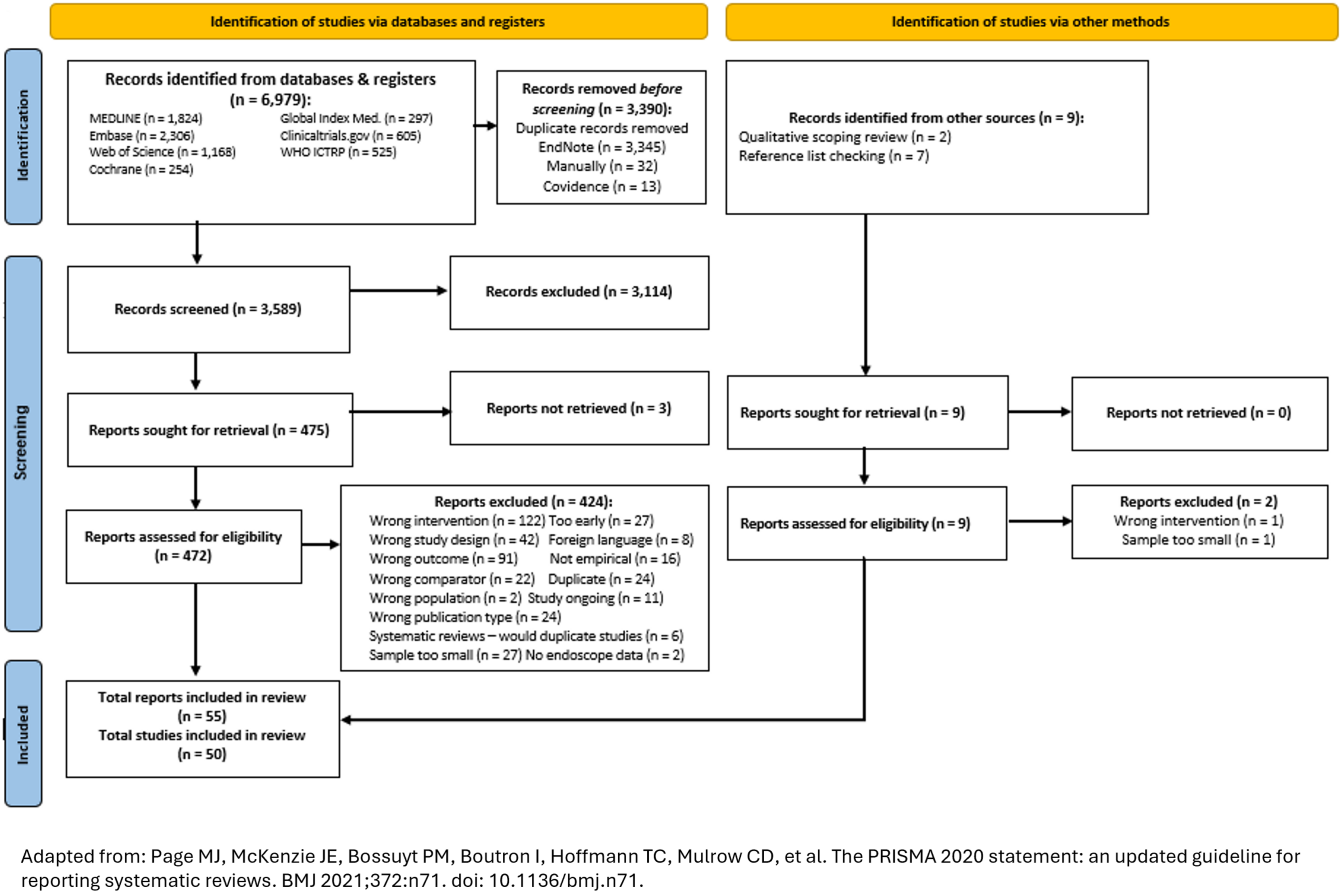
PRISMA flow diagram for literature search and study selection process.

After de‐duplication, database searches identified 3589 records (see Figure 1). In total, we retrieved 481 records (including 10 from additional sources) for further assessment. Three could not be retrieved. Four hundred twenty‐six were excluded, leaving 55 documents describing 50 studies that met our inclusion criteria [18, 19, 20, 21, 22, 23, 24, 25, 26, 27, 28, 29, 30, 31, 32, 33, 34, 35, 36, 37, 38, 39, 40, 41, 42, 43, 44, 45, 46, 47, 48]. Thirty‐one studies published as full‐text articles informed our main analyses [18, 19, 20, 21, 22, 23, 24, 25, 26, 27, 28, 29, 30, 31, 32, 33, 34, 35, 36, 37, 38, 39, 40, 41, 42, 43, 44, 45, 46, 47, 48]; 19 studies published only as conference abstracts and trial registration records provided supplementary information for sensitivity analyses [49, 50, 51, 52, 53, 54, 55, 56, 57, 58, 59, 60, 61, 62, 63, 64, 65, 66, 67].

Of the 31 studies with full‐text publications, 12 (including one systematic review) evaluated duodenoscopes/endoscopic retrograde cholangiopancreatography (ERCP) [19, 20, 21, 22, 23, 25, 26, 27, 31, 36, 37, 41], 16 assessed other upper GI endoscopes [18, 24, 29, 30, 32, 33, 35, 38, 39, 40, 42, 43, 44, 46, 47, 48], and two evaluated endoscopes for lower GI tract [34, 45]. One systematic review covered the risk of endoscope‐related infections across all types of MU GI endoscopes [28] (Table S3).

Of the 29 primary studies published in full‐text, eight adopted an RCT design [32, 33, 34, 36, 37, 38, 43, 45], eight adopted a DTA design [29, 30, 35, 39, 40, 46, 47, 48], three were non‐randomized comparative studies [22, 25, 31], and 10 were case series (see Table 1) [18, 19, 20, 21, 23, 24, 26, 27, 42, 44]. Fifteen studies were single‐centre studies [18, 21, 29, 30, 34, 35, 36, 38, 39, 42, 44, 45, 46, 47, 48]; six involved two centres [24, 31, 32, 33, 37, 43]; and eight involved more than two centres [19, 20, 22, 23, 25, 26, 27, 40]. Only three SU (EXALT Model D duodenoscope, Boston Scientific) or partially SU (EG Scan, IntroMedic & EndoSheath TNE‐5000, Vision Sciences) GI endoscopes had more than two full‐text publications meeting our inclusion criteria (Table S4).

**TABLE 1:** Key characteristics of included studies.

Study and type of publication^a^	Study design	Comparison and risk of bias^b^	Endoscope(s) evaluated	Country	Total sample size	Age	Technical performance	Diagnostic accuracy	Infection	Other adverse events	Clinical and PROM
Duodenoscopy/ERCP (23)											
**Bang et al.** [36] **Full text**	**Randomized controlled trial (RCT**)	SU versus MU Some concerns in multiple domains	Exalt Model D (Boston Scientific), TJF‐180 (Olympus)	USA	105	Mean (SD): 67 (14) versus 61 (18)	Y	N	Y	Y	N
Gambaccini et al. [50] Abstract	Non‐randomized comparative study	SU versus MU Moderate risk of confounding	Exalt Model D (Boston Scientific), MU (details NR)	Italy	175	NR	Y	N	N	Y	N
Razzak et al. [52] Abstract	Non‐randomized comparative study	SU versus MU Moderate risk of confounding	Exalt Model D (Boston Scientific), TJF‐180 (Olympus)	USA	133	Mean: 70 (±12) versus 69 (±14)	Y	N	N	Y	N
Gromski et al. [53] Abstract	Non‐randomized comparative study	SU versus MU Moderate risk of confounding	Exalt Model D (Boston Scientific), TJF‐160/TJF‐180 (Olympus)	USA	614	Median (IQR): 61 (17) versus 57	Y	N	N	N	N
Gupta et al. [54] Abstract	Non‐randomized comparative study	SU versus MU Moderate risk of confounding	Exalt Model D (Boston Scientific), TJF‐180 (Olympus)	USA	50	NR	Y	N	N	N	N
Shahid et al. [22] Full text	Non‐randomized comparative study	SU versus SU Serious risk of confounding	Exalt Model D (Boston Scientific), aScope (Ambu)	Spain, USA	201	Mean: 63 versus 65	Y	N	N	Y	N
Liao et al. [57] Abstract	Non‐randomized comparative study	SU versus SU Moderate risk of confounding	EXALT Model D (Boston Scientific), aScope (Ambu)	USA	109	Mean: 58 vs. 55	Y	N	N	Y	N
Machado et al. [55] Abstract	Non‐randomized comparative study	SU versus MUDC Moderate risk of confounding	Exalt Model D (Boston Scientific), TJF‐Q190V (Olympus)	USA	156	Mean: 64 (IQR 54–72)	Y	N	N	Y	N
Bruno et al. [26] Full text	Case series	SU only Non‐comparative	EXALT Model D (Boston Scientific)	11 countries^c^	551	Mean: 60	Y	N	Y	Y	N
Napoléon et al. [20] Full text	Case series	SU only Non‐comparative	EXALT Model D (Boston Scientific)	France	60	Median: 66	Y	N	Y	Y	N
Persyn et al. [21] Full text	Case series	SU only Non‐comparative	EXALT Model D (Boston Scientific)	Belgium	52	Mean (range): 65 (9–98)	Y	N	Y	Y	N
Slivka et al. [23] Full text	Case series	SU only Non‐comparative	EXALT Model D (Boston Scientific)	Netherlands, USA	200	Mean: 63	Y	N	Y	Y	N
Muthusamy et al. [27] Full text	Case series	SU only Non‐comparative	EXALT Model D (Boston Scientific)	USA	73	Mean: 64	Y	N	Y	Y	N
Fugazza et al. [19] Full text	Case series	SU only Non‐comparative	EXALT Model D and SpyGlass DS II (Boston Scientific)	Belgium, India, Italy, Saudi Arabia, USA	66	Mean (±SD): 67 (±16)	Y	N	Y	Y	N
Venkatachalapathy et al. [65] Abstract	Case series	SU only Non‐comparative	EXALT Model D (Boston Scientific)	Republic of Ireland and UK	59	NR	Y	N	Y	N	Y
**Forbes et al.** [37] **Full text**	**RCT**	MUDC versus MU **Low risk of bias**	ED34‐i10T2 (Pentax), ED34‐i10T (Pentax)	Canada	520	Mean (±SD): 60 (17) vs. 61 (17)	Y	N	Y	Y	N
Straulino et al. [60] Abstract	Non‐randomized comparative study	MUDC versus MU Moderate risk of confounding	ED34‐i10T2 (Pentax), MU (details NR)	Germany	71	NR	Y	N	N	Y	N
Barakat et al. [31] Full text	Non‐randomized comparative study	MUDC versus MU Moderate risk of confounding	Evis Exera III (Olympus)/TJF‐Q190V (Olympus), TJF‐Q180V (Olympus)	USA	106	Mean (±SD): 14 (±4)	Y	N	N	Y	N
Hutfless et al. [25] Full text	Non‐randomized comparative study (retrospective cohort study)	SU versus MU Serious risk of confounding	SU (details NR), MU (details NR)	USA	823 575	NR	N	N	Y	N	N
Shiratori et al. [66] Abstract	Non‐randomized comparative study	SU versus MU Serious risk of confounding	SU (details NR), MU (details NR)	USA	135 720	NR	N	N	Y	Y	N
Pona et al. [56] Abstract	Non‐randomized comparative study	SU versus MU Serious risk of confounding	SU (details NR), MU (details NR)	USA	58	NR	Y	N	N	N	N
Lee et al. [59] Abstract	Non‐randomized comparative study	MUDC versus MU Moderate risk of confounding	MUDC (details NR), MU (details NR)	South Korea	196	NR	Y	N	Y	Y	N
Kwakman et al. [41] Full text	Systematic review	MU only Infections subject to detection and reporting bias	TJF‐180 V (Olympus) duodenoscope for 2 outbreaks, Olympus (model NR)	The Netherlands	3 studies	NR	N	N	Y	N	N
Other upper GI endoscopy (24)											
**Kang et al.** [29] **Full text**	**Randomized, fully paired diagnostic test accuracy study**	SU versus MU Unclear risk in patient flow	EG Scan III (IntroMedic), GIF‐H260 (Olympus)	South Korea	50	Mean (±SD): 43 (±15)	Y	Y	N	Y	Y
**Sami et al.** [40] **Full text**	**Fully paired diagnostic test accuracy study**	SU versus MU High risk in patient selection	EG Scan II (IntroMedic), GIF‐260H/GIF‐H180 (Olympus)	UK, USA	200	Mean: 58	Y	Y	N	Y	Y
**Huynh et al.** [35] **Full text**	**Fully paired diagnostic test accuracy study**	SU versus MU **Low risk of bias**	EG Scan II (IntroMedic), QF 180 (Olympus)	Australia	48	Mean (±SD): 56 (± 1)	Y	Y	N	N	Y
**Sami et al.** [30] **Full text**	**Fully paired diagnostic test accuracy study**	SU versus MU **Low risk of bias**	EG Scan II (IntroMedic), GIF‐H260 (Olympus)	UK	50	Mean: 59	Y	Y	N	Y	N
**Halland et al.** [51] **Abstract**	**Fully paired diagnostic test accuracy study**	SU versus MU Unclear risk in patient selection	EG Scan II (IntroMedic), MU (details NR)	USA	54	NR	Y	Y	N	Y	Y
**Jin et al.** [58] **Abstract**	**Fully paired diagnostic test accuracy study**	SU versus MU Unclear risk for index test and reference standard	EG scan (version NR), MU (details NR)	South Korea	27	Median (range): 56 (26–80)	Y	Y	N	Y	N
**Aedo et al.** [48] **Full text**	**Randomized, fully paired diagnostic accuracy study**	SU versus MU **Low risk of bias**	EG Scan I (IntroMedic), GIF‐XGIF‐160 (Olympus)	Mexico	96	Mean (range): 50 (18–79)	Y	Y	N	N	Y
**Choi et al.** [46] **Full text**	**Fully paired diagnostic accuracy study**	SU versus MU Unclear risk of bias	EG Scan I (IntroMedic), MU (details NR)	South Korea	129	Mean (±SD): 64 (±14)	Y	Y	N	Y	N
**Cho et al.** [47] **Full text**	**Fully paired diagnostic accuracy study**	SU versus MU Unclear risk of bias	EG Scan I (IntroMedic), MU (details NR; including GIF‐Q260)	South Korea	13	Mean (±SD): 72 (±14)	N	Y	N	Y	N
Eqbal et al. [18] Full text	Case series	SU only Non‐comparative	EG Scan II (IntroMedic)	Australia	40	Median (IQR): 60 (57–67)	Y	Y	N	Y	N
Chung et al. [44] Full text	Case series	SU only Non‐comparative	EG Scan I (IntroMedic)	South Korea	50	Mean (±SD): 49 (±14)	Y	N	N	Y	Y
Dowds et al. 2024 [63] Abstract	Case series	SU only Non‐comparative	SU (EvoEndo, model NR)	USA	78	Range: 8–21	Y	N	N	Y	Y
Lee et al. [64] Abstract	Case series	SU only Non‐comparative	SU (EvoEndo, model NR)	USA	49	Median (range): 14 (7–20)	Y	N	N	Y	Y
**Shariff et al.** [39] **Full text**	**Randomized, fully paired diagnostic test accuracy study**	Sheathed MU versus MU **Low risk of bias**	Ultrathin Transnasal EndoSheath TNE–5000 (Vision Sciences), IF‐Q240Z (Olympus)	UK	25	Median (range): 59 (24–76)	Y	Y	N	N	N
**Sami et al.** [43] **Full text**	**RCT**	Sheathed MU versus MU Low risk for participation rates but some concerns for other outcomes	Ultrathin Transnasal EndoSheath TNE‐5000 (mobile unit/hospital), GIF‐180 (Olympus)	USA	209	Mean (±SD): 65 (±9) versus 64 (±9) versus 66 (±9)	Y	N	N	Y	Y
Streckfuss et al. [42] Full text	Case series	Sheathed MU only Non‐comparative	Ultrathin Transnasal EndoSheath TNE–5000 (Vision Sciences)	Germany	55	Mean (±SD): 53 (±16)	Y	N	N	Y	Y
Peery et al. [49] Abstract	Case series	Sheathed MU only Non‐comparative	Ultrathin Transnasal EndoSheath TNE–5000 (Vision Sciences)	USA	264	Mean (±SD): 55 (±9)	Y	N	N	Y	Y
**Jin et al.** [38] **Full text**	**RCT**	Sheathed MU versus MU Some concerns in > 1 domains	Modified GIF‐V70 (Olympus/Shenda), GIF‐V70 (Olympus)	China	1120	Mean (±SD): 35 (±11) versus 35 (±10)	Y	Y	N	Y	Y
Jin et al. [61] Abstract	Non‐randomized comparative study	Sheathed MU versus MU Serious risk of confounding	Sheathed gastroscope (Shenda, model NR), MU (details NR)	China	54	NR	Y	N	Y	Y	N
**Li et al.** [32] **Full text**	**RCT**	SU versus MU High risk in outcome measurement and reporting of results	PR‐IPD002 (Shenzhen PengRui), GIF‐260 (Olympus)	China	144	Mean (±SD): 44 (±11) versus 43 (±10)	Y	N	Y	Y	N
**Luo et al.** [33] **Full text**	**RCT**	SU versus MU Some concerns in > 1 domains	ZING‐W200B (Huizhou Xianzan), GIF‐HQ290/GIF‐H290 (Olympus)	China	110	Mean (±SD): 37 (40 ± 13) versus 39 (43 ± 14)	Y	N	Y	Y	Y
Van der Ploeg et al. [24] Full text	Case series	SU only Non‐comparative	aScope Gastro (Ambu)	The Netherlands, Norway	60	Mean (range): 62 (54–74)	Y	N	N	Y	N
**Römmele et al.** [62] **Abstract**	**RCT**	SU versus MU Some concerns in > 1 domains	SU (details NR), MU (details NR)	Germany	92	Mean: 70	Y	N	N	Y	N
**Wang et al.** [67] **Abstract**	**RCT**	SU versus MU Some concerns in > 1 domains	SU (details NR), MU (Pentax, model NR)	China	42	NR	Y	N	N	Y	N
Lower GI endoscopy (2)											
**Xu et al.** [45] **Full text**	RCT	SU versus MU High risk in selection of reported results	EndoFresh XZING‐W200B^d^ (HuiZhou Xzing), EG‐600WR (Fujifilm)	China	42	Mean (±SD): 40 (±7) versus 41 (±9)	Y	N	N	Y	Y
Wei et al. [34] Full text	RCT	SU versus MU High risk in randomization process	XZING‐C200^e^ (HuiZhou Xzing), CF‐HQ290ZI, CF‐H290I, PCF‐TYPE‐Q260AZI (Olympus)	China	90	Mean (±SD): 43 (±14) versus 43 (±13)	Y	Y	N	Y	N
Multiple types of GI endoscopy (1)											
Deb et al. [28] Full text	Systematic review	MU only Infections subject to detection and reporting bias; uncertain causality (mixed endogenous and exogenous pathogens)	All MU GI endoscopes (details NR)	Multiple countries	118 studies	NR	N	N	Y	N	N

Abbreviations: IQR, interquartile range; MU, multiple‐use; MUDC, multiple‐use endoscopes with disposable cap; NR, not reported; PROM, patient‐reported outcome measure; SD, standard deviation; SU, single‐use.

^a^ Full‐text articles were highlighted with a white background; RCTs and fully paired diagnostic test accuracy studies and systematic reviews were emphasized with bold font. If a study only reported findings in its trial registration with no full‐text article available, this was reported and analyzed as an abstract.

^b^ For brevity we highlight key risk of bias (if present) in this table. Full results for risk of bias assessment are available (see Supporting information Data S5).

^c^ Australia, Canada, China, France, Germany, Hong Kong, India, Italy, Netherlands, Singapore, South Africa, and the United States.

^d^ SU gastroscope for endoscopic rubber band ligation of internal hemorrhoids.

^e^ SU colonoscope.

### 3.1 Duodenoscopes/ERCP

Twelve studies with full‐text publications assessed duodenoscopes and/or ERCP [19, 20, 21, 22, 23, 25, 26, 27, 31, 36, 37, 41]. Five of them (two RCTs [36, 37] and three non‐randomized comparative studies [22, 25, 31]) provided evidence directly comparing SU versus MU [25, 36], MUDC versus MU [31, 37], or SU versus SU duodenoscopes [22]. The remaining included six case series of SU duodenoscopes [19, 20, 21, 23, 26, 27] and a systematic review of transmission of infection associated with MU duodenoscopes [41]. Only one study [37] was judged to be of low risk of bias (see Table 1).

#### 3.1.1 Technical Performance

Ten studies reported diverse measures of technical performance, including overall procedure success rates and crossover from SU/MUDC to MU duodenoscopes; success rates for specific procedures; measures of image quality and maneuverability; time to completion of specific procedures; overall duration of the endoscopic procedure and time required pre‐ and/or post‐endoscopic procedures (for preparations and/or cleaning). The remaining two studies [25, 41] focused on infections related to ERCP.

**FIGURE 2:**
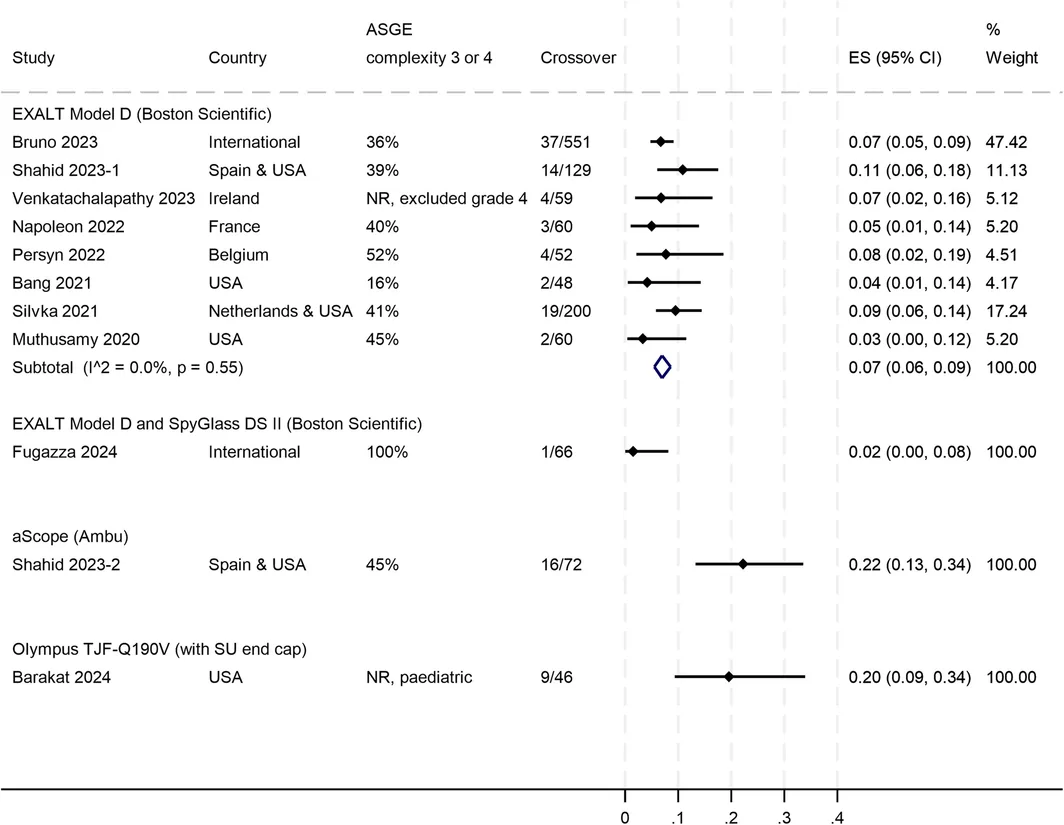
Crossover from single‐use (SU) or multiple‐use duodenoscopes with disposable cap (MUDC) to multiple‐use (MU) duodenoscopes.

Crossover from SU or MUDC to MU duodenoscopes was reported in 10 studies (see Figure 2). The pooled crossover rate based on data from eight studies evaluating EXALT Model D (Boston Scientific) was 7% (95% CI 6%–9%, *I* ^2^ = 0%). The pooled rate of successful ERCP following cross‐over for these studies was 69% (95% CI 45%–90%, *I* ^2^ = 63%; Data S3), with a high level of heterogeneity. Sensitivity analyses produced very similar results (Data S3). Comparative evidence for other technical performance measures was limited and generally suggested similar or slightly worse performance for SU compared with MUDC and MU duodenoscopes (Data S3). No studies of duodenoscopes evaluated diagnostic accuracy.

#### 3.1.2 Clinical Infection Risk and Adverse Events

One non‐randomized comparative study using data from an administrative database with serious risk of confounding bias reported lower odds of hospitalization for infection following ERCP for SU versus MU (7‐day infection, odds ratio 0.32 [0.17–0.60]) [25]. The earlier findings appeared to be refuted by later analyses reported in a conference abstract by the same research team, which showed no significant difference in post‐ERCP infection between SU and MU duodenoscopes (7‐day infection, odds ratio for SU versus MU 1.30 [0.98–1.73]) [66]. Two systematic reviews provided estimates of post‐ERCP infections arising from transmission of exogenous pathogens through contaminated MU duodenoscopes [28, 41]. The risk of clinical infection was estimated to be 0.01% (95% prediction interval [PI] 0.0093%–0.0116%), or approximately 1 per 10 000 ERCP procedures in a Dutch study based on published cases of outbreaks of infection. The authors stressed that due to likely under‐detection and underreporting, the true risks could be much higher [41]. The other systematic review by Deb et al. covered all GI endoscopy and included 118 published studies [28]. The incidence of post‐ERCP infection was estimated to be 0.8% (3452/433414) based on 51/88 studies providing sufficient data for calculating the incidence. However, the authors included infections caused by both exogenous and endogenous pathogens and did not distinguish between them. Very few infectious and noninfectious adverse events were reported among other included primary studies of SU and MUDC duodenoscopes (see Data S3 and Data S4). There were limited data on patient experience/outcomes (Data S4).

### 3.2 Upper GI Endoscopes

Sixteen full‐text studies assessed other upper GI endoscopes, including four studies of standard size SU [24, 32, 33] or sheathed MU [38] endoscopes and 12 studies of ultrathin SU [18, 29, 30, 35, 40, 44, 46, 47, 48] or MU endoscopes with SU sheath [39, 42, 43] met our inclusion criteria. Twelve of these studies (eight fully paired DTA studies [29, 30, 35, 39, 40, 46, 47, 48] and four RCTs [32, 33, 38, 43]) provided comparative evidence against MU endoscopes. The remaining four studies were case series [18, 24, 42, 44]. Eight studies published only as conference abstracts provided additional data for sensitivity analyses [49, 51, 58, 61, 62, 63, 64, 67]. Upper GI endoscopy was undertaken for the investigation of various upper GI symptoms [24, 29, 32, 33, 38, 40, 42, 44, 48], including GI bleeding [46, 47], and screening and/or surveillance of Barrett's esophagus [39, 43] and esophageal varices [18, 30, 35]. Only four studies (all of DTA design) were judged to be of low risk of bias (see Table 1 and Data S5) [30, 35, 39, 48].

#### 3.2.1 Technical Performance

All 16 studies reported some measures of technical performance of SU/sheathed MU against standard‐size MU upper GI endoscopes. Successful intubation or procedure completion rates for SU or sheathed MU endoscopes ranged from 85% to 100% (Data S3 and Data S4). Image quality and operators' assessment tended to be similar or slightly worse for SU/sheathed MU compared with MU endoscopes (see Data S4).

#### 3.2.2 Diagnostic Accuracy

**FIGURE 3:**
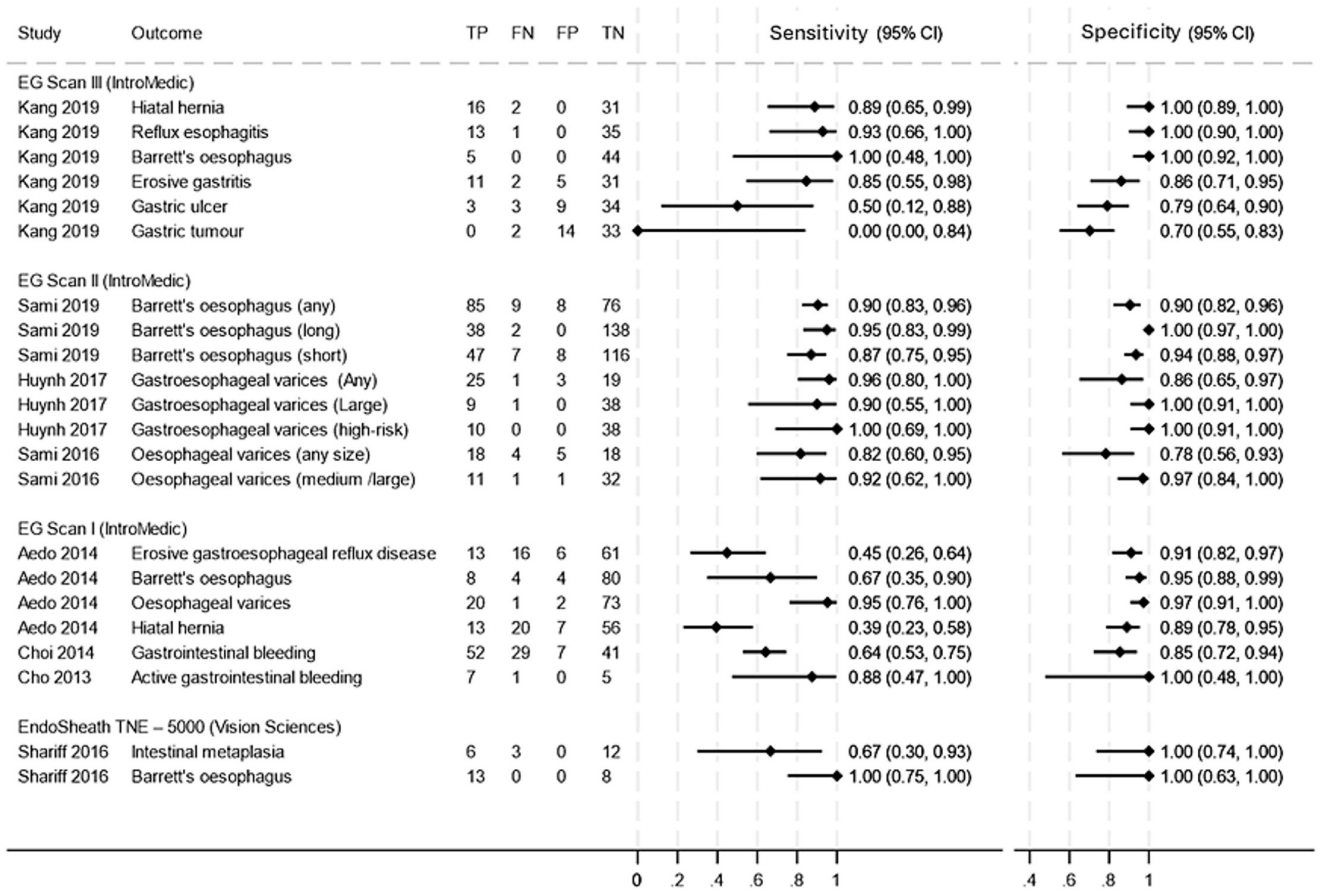
Diagnostic accuracy for SU and sheathed MU upper GI endoscopes compared with MU endoscopes.

Twelve studies reported outcomes related to diagnostic accuracy, all of which used findings from standard size MU endoscopes as the reference standard (Data S4). Seven studies reported sensitivity and specificity for various upper GI conditions for ultrathin SU or sheathed MU endoscopes (see Figure 3). SU and sheathed MU endoscopes had similar or lower sensitivity and specificity compared with MU endoscopes; the accuracy might be adequate for esophageal conditions for screening/triaging purposes but might be inadequate for detecting other GI conditions. Three studies reported greater patient preference for unsedated transnasal ultrathin SU endoscopes over sedated MU endoscopes (Data S4) [30, 35, 40]. No serious adverse events or clinical infections associated with SU or sheathed MU upper GI endoscopes were reported in included studies. Self‐limiting epistaxis was common for transnasal endoscopes (Data S4).

### 3.3 Lower GI Endoscopes

Two RCTs evaluated SU endoscopes used in the lower GI tract. Xu et al. (*n* = 42) compared an SU gastroscope with a MU gastroscope for endoscopic rubber band ligation of internal hemorrhoids; Wei et al. (*n* = 90) evaluated an SU versus MU colonoscope for detecting adenoma and mucosal resection.

Both trials were judged to be of high risk of bias (Data S5). Similar procedure success rates, therapeutic outcomes (for rubber band ligation of hemorrhoid), adenoma detection (for colonoscopy), and adverse events were observed between SU and MU endoscopes, although the image quality and detection of diminutive polyps were lower, and time taken to perform endoscopic mucosal resection was significantly longer for SU versus MU colonoscopes (466 [SD 181] vs. 206 [110] seconds, *p* < 0.001) (Data S3 and Data S4). No serious adverse events and clinical infection were reported.

## 4 Discussion

This systematic review comprehensively searched and critically appraised evidence on comparative performance of SU, and partially SU GI endoscopes (MUDCs and sheathed MU endoscopes), against MU endoscopes. Our findings suggest that SU and partially SU GI endoscopes had similar or slightly worse technical performance and diagnostic accuracy compared with their MU counterparts. Blanket replacement of MU GI endoscopes by their SU or partially SU counterparts is therefore not recommended with the exception of duodenoscopes and in situations where patients are at high risk of acquiring or transmitting infection, in line with current endoscopist consensus [68].

Two previous systematic reviews of SU duodenoscopes included only four [69] and seven studies [8], respectively, and did not cover comparative evidence. A further review examining sheathed flexible endoscopes in different clinical areas though did not quantitatively assess diagnostic accuracy [70]. Not only did our review fill in these important gaps, but also we were able to assess how SU, partially SU and MU GI endoscopes compared across multiple outcomes. Informed decision‐making, whether in policy and practice, relies on being able to see the whole picture.

Although our review included over 30 studies published as full text and nearly 20 additional studies published as conference abstracts, very few studies provided comparative data on infection risk between SU/partially SU and MU endoscopes. Those that reported this had serious limitations. Even the largest RCT for MUDC versus MU duodenoscopes (*n* = 518) [37] was substantially underpowered for detecting important differences in infection risk due to the rarity of events. Emerging evidence from studies based on large observational datasets reported conflicting findings [25, 66]. Data on the risk of post‐procedure infection associated with transmission of exogenous pathogens by contaminated MU GI endoscopes were also very limited.

Only five out of 50 studies included in our review were rated as having low risk of bias. Lack of randomization, unclear arrangement for allocation concealment, unclear reporting of deviation from study protocol and methods of analysis, and unclear impact of lack of blinding on outcome assessment were among the main concerns for comparative studies, whereas inadequate description of patient selection (e.g., whether consecutive patients were enrolled) and exclusion was an issue for diagnostic test accuracy studies and case series.

Some issues related to technical performance, such as a lower rate of successful procedures with SU or partially SU GI endoscopes, may be related to the design and materials used for manufacturing. For example, Barakat et al. reported that endoscopists experienced increased stiffness of the SU cap region for a MUDC and challenges with alignment at the ampulla for cannulation in pediatric patients [31]. These issues might be mitigated by better design. Unfamiliarity with new devices could also contribute to less favorable technical performance outcomes of SU for novice users at the initial stage, although a recent study of SU duodenoscopes suggested that the learning curve was relatively short and the operator's proficiency was achieved after around 10 cases [71].

The proposed advantages for SU ultrathin transnasal upper GI endoscopes are their portability allowing use in office/community settings outside traditional endoscopy suites and elimination of the need for sedation [58]. These advantages could in theory improve accessibility of upper GI endoscopy particularly in population screening settings and in remote areas. Whether the advantages outweigh the drawback of lower procedure completion rates and diagnostic accuracy and in some cases inability to obtain biopsy may be context dependent and require separate evaluation for different settings. Qualitative research has shown that the accuracy and validity of test findings are important considerations for patients in addition to convenience and (dis)comfort of the procedures [72]. All studies of SU ultrathin transnasal endoscopes used standard‐size MU upper GI endoscopes as the comparators; comparisons with MU ultrathin transnasal endoscopes (which can be decontaminated and vacuum‐packed for use in remote areas and community settings) may be warranted in future studies.

To the best of our knowledge, this is the first systematic review that has summarized clinical evidence across different types of SU and partially SU GI endoscopes and included direct comparative evidence against MU. We examined and supplemented our findings with emerging evidence reported in conference abstracts. Searching clinical trial registries and including conference abstracts in our review allowed us to take into account emerging evidence in this rapidly evolving field, as well as partially mitigate the risk of publication and outcome reporting bias [13]. Following advice from the Advisory Group, we only included studies published from 2010 onwards, as evidence reported in studies published earlier may have questionable applicability due to technological advances in GI endoscopy.

Our review has limitations. Only English language articles were considered. We could not quantitatively examine potential publication bias and outcome reporting bias using funnel plots and related methods due to lack of sufficient studies, and these biases could not be ruled out. Data reported in conference abstracts may be more susceptible to inaccuracies as they tend to be preliminary findings and have not been subjected to the same peer review process as full‐text articles. We are aware that new technologies such as capsule endoscopes are also SU devices that enable visualization of the GI tract but could not accommodate these within the scope of this review. We planned to, but did not, assess the overall certainty of the evidence using the GRADE framework [73]. Given the small number of studies associated with individual endoscope models and many weaknesses in study designs, most of the assessments would result in a designation of very low certainty for comparative evidence. A major challenge for quantitative synthesis of findings across studies in our review was the diverse outcomes measured and reported in individual studies. Development of core outcome sets for evaluating GI endoscopy may facilitate quantitative synthesis of evidence in the future. We were also unable to carry out planned subgroup analyses for clinical infection risk due to insufficient data.

This review provides a comprehensive examination of clinical evidence related to SU versus MU GI endoscopes. For a well‐informed decision in clinical practice and policy, the evidence needs to be considered alongside evidence on comparative cost‐effectiveness and environmental impact through economic evaluations and life cycle assessments that cover the whole life cycle of the endoscopes [74, 75]. This is an area of active research, where emerging evidence including that from our broader SUMU‐Endo project (https://fundingawards.nihr.ac.uk/award/NIHR152311) indicates the need for a nuanced approach informed by comprehensive assessments that take into account patient, clinical, economic and environmental considerations for different clinical and service delivery scenarios.

In conclusion, this systematic review appraised and synthesized growing clinical evidence of SU and partially SU compared with MU GI endoscopes from 50 studies. The volume of evidence varied substantially between individual SU/partially SU GI endoscopes—only three models had more than two full‐text publications. SU and partially SU GI endoscopes generally have comparative/slightly inferior technical performance and test accuracy compared with MU endoscopes. Higher patient preference for ultrathin, transnasal upper GI endoscopes was reported in some studies compared with standard size MU endoscopes. Reliable evidence comparing the risk of infection and adverse events between SU, partially SU, and MU GI endoscopes is lacking, as are reliable estimates of the risk of transmission of exogenous pathogens by contaminated MU GI endoscopes. Consequently, a blanket replacement of MU endoscopes by SU or partially SU endoscopes cannot be recommended except when there are particular concerns regarding infection transmission during endoscopic procedures.

Large‐scale randomized comparative studies and/or molecular typing studies are needed to reliably quantify potential reduction in infection risk related to transmission of exogenous pathogens by SU/partially SU endoscope. As with all novel medical devices, ongoing collection and evaluation of comparative clinical evidence are essential in view of continuous advance in the design and manufacturing of SU and partially SU endoscopes as well as in reprocessing methods for MU endoscopes.

## Funding

A.B, A.G., J.B., L.A., M.Z., N.T.‐M., R.A., S.R.C., and Y.‐F.C. received payment from the project funding from the National Institute for Health and Care Research (NIHR) Health and Social Care Delivery Research (HSDR) Programme (project number NIHR152311). Payments were made by the NIHR to the University Hospitals Coventry and Warwickshire, the University of Warwick, and the University of Birmingham. T.M. was part supported by the Health Research Board (Ireland) and the HSC Public Health Agency (Grant number ESI‐2021‐001) through Evidence Synthesis Ireland. Y.‐F.C. also received a grant paid to the University of Warwick to support the attachment of a Fellow (T.M.) of Evidence Synthesis Ireland to the project.

## Conflicts of Interest

B.H. received payment or honoraria for lectures, presentations, speakers bureaus, manuscript writing, or educational events from Boston Scientific EMEA and Pentax EMEA. J.P. received funding for PhD Studentship; Stipend; PhD topic unrelated (Public Health Intelligence Challenges for Local Authorities Responding to COVID‐19) from NIHR. T.M. received a EUR1000 educational fund from Evidence Synthesis Ireland and Cochrane Ireland and EUR8000 grant paid to University of Warwick from Health Research Board (Ireland), HSC Public Health Agency (Northern Ireland), National University of Ireland, Galway, Evidence Synthesis Ireland, and Cochrane Ireland. S.S. holds grants from Taked, AbbVie, Tillots Pharma, Pfizer, Johnson & Johnson Innovative Medicine, and Biogen; received consulting fees from Takeda, AbbVie, Merck, Ferring, Pharmacosmos, Warner Chilcott, Johnson & Johnson Innovative Medicine, Dr. Falk Pharma, Biohit, TriGenix, Celgene, and Tillots Pharma; has received payment or honoraria for lectures, presentations, speakers bureaus, manuscript writing, or educational events from AbbVie, Takeda, Celltrion, Pfizer, Biogen, AbbVie, Johnson & Johnson Innovative Medicine, Merck, Warner Chilcott, Falk Pharma, and Eli Lilly; and received support from attending meetings and/or travel from Takeda, AbbVie, Merck, Ferring, Pharmacosmos, Warner Chilcott, Johnson & Johnson Innovative Medicine, Dr. Falk Pharma, Biohit, TriGenix, Celgene, Tillots Pharma, Alphasigma, and Eli Lilly. a.d. has received consulting fees/speaker fees/advisory honoraria from Takeda, Alfasigma, Tillotts Pharma UK, Dr. Falk Pharma UK, Lilly, AbbVie UK, Pharmacosmos, Cook Medical, Boston Scientific Corporation, and Johnson & Johnson Innovative Medicine. He has also received financial support to attend conferences and meetings from Tillotts Pharma, Dr. Falk Pharma UK, Johnson & Johnson, Synmed UK, Pharmacosmos, Celltrion, and Lilly. L.A. is currently receiving financial support from NIHR through an Advanced Fellowship.

## Supporting information


**Data S1:** Deviations from review protocol.

**Data S2:** Search strategies.

**Data S3:** Additional forest plots.

**Data S4:** Additional evidence from included studies.

**Data S5:** Risk of bias tables.

**Table S1:** PRISMA checklist.

**Table S2:** Reasons for exclusion at full‐text stage.

**Table S3:** Types of endoscope and study design of included studies.

**Table S4:** Number and types of studies evaluating individual fully or partially single‐use GI endoscopes.

## Data Availability

All available data are presented in this article and associated supplementary materials.
