# Supplementary material for: Diagnostic and Clinical Performance of Single‐Use Versus Reusable Gastrointestinal Endoscopes: A Systematic Review and Meta‐Analyses
Source: J Gastroenterol Hepatol. 2026 Aug 7;41(9):2571–87. doi: 10.1111/jgh.70637 (PMC13534234; doi:10.1111/jgh.70637)
Supplement: Supplementary file 1 [file JGH-41-2571-s001.pdf]

Diagnostic and clinical performance of single-use versus reusable gastrointestinal endoscopes: a systematic review and meta-analyses

Supporting information Data S3: Additional forest plots

Authors

Natalie Tyldesley-Marshall, Yen-Fu Chen,* Jefferson Buendia, Anna Brown, Thomas Matthews, Janette Parr, Shivashri Chockalingam, Adel Elfeky, Amy Grove, Lazaros Andronis, Stuart R Coles, Mandana Zanganeh, Bu Hayee, Anjan Dhar, Shaji Sebastian, Ramesh Arasaradnam on behalf of the SUMU-Endo Project

*Correspondence to: Prof. Yen-Fu Chen

Centre for Evidence and Implementation Science, Park House, Edgbaston, University of Birmingham, Birmingham B15 2TT, United Kingdom

y.chen.25@bham.ac.uk

Department of Health and Welfare, College of City Management, University of Taipei, Taipei, Taiwan

yfchen@utaipei.edu.tw

Additional forest plots

1. Duodenoscopes technical performance

1.1 Additional main analyses

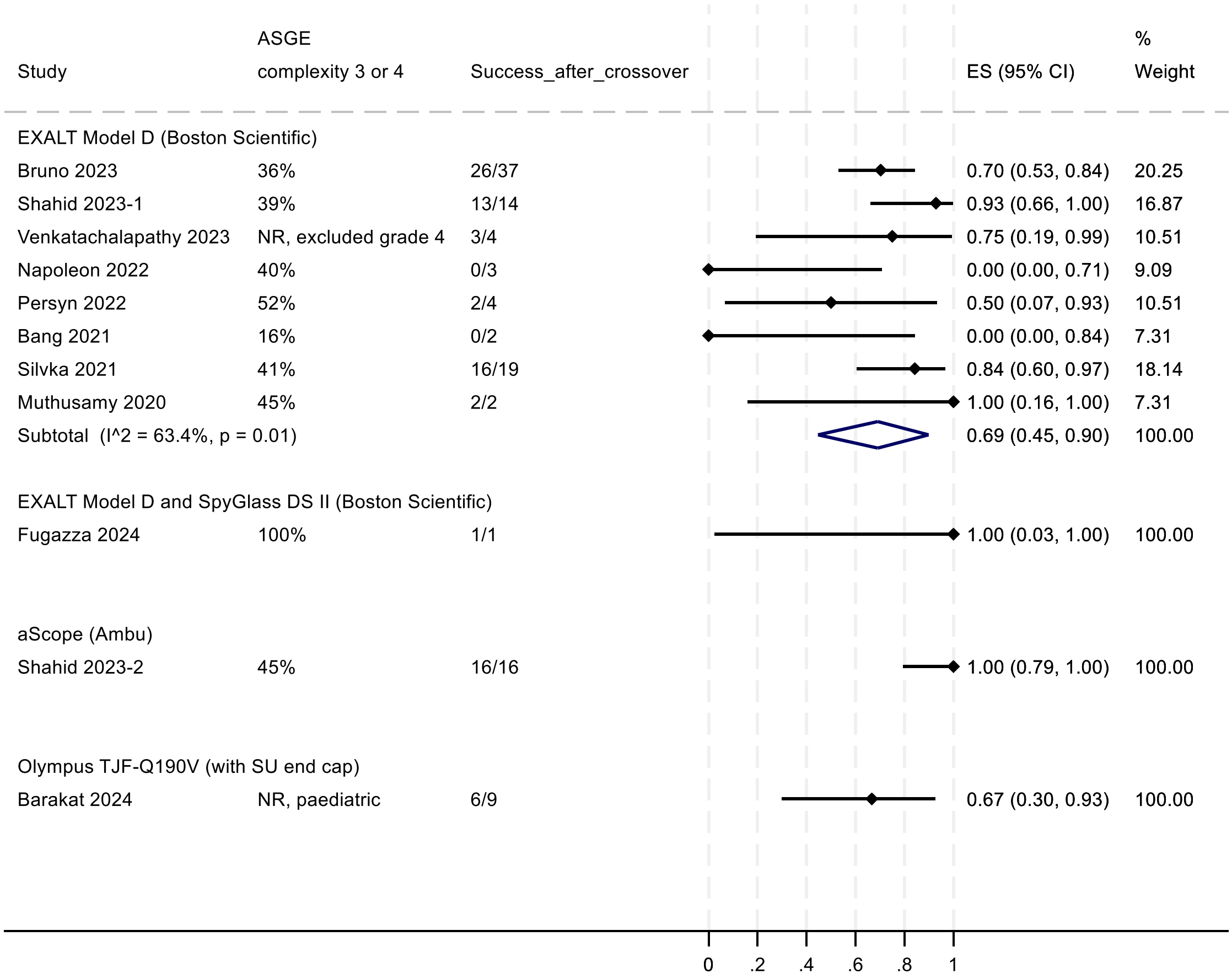

Supplementary Figure 1 Single-use duodenoscope: success rate after crossover – full-text only ·

Comparison and Study	Publication type	Design	Country	Comparison	Outcome	SU_success	SU_total	MUDC_success	MUDC_total	MU_success	MU_total	Difference in success rates (95% CI)
SU vs MU												
Bang 2021	Full-text	RCT	USA	SU vs MU	Cannulation	46	48			50	50	-0.04 (-0.11, 0.03)
Gambaccini 2023	Abstract	NRCS	Italy	SU vs MU	Cannulation/ERCP	25	25			140	150	0.07 (0.00, 0.13)
Razzak 2023	Abstract	NRCS	USA	SU vs MU	ERCP	47	53			75	80	-0.05 (-0.15, 0.05)
Gromski 2021-2	Abstract	NRCS	USA	SU vs MU	ERCP	47	51			514	514	-0.08 (-0.15, -0.00)
MUDC vs MU												
Forbes 2023-1	Full-text	RCT	Canada	MUDC vs MU	Biliary cannulation			231	241	219	234	0.02 (-0.02, 0.06)
Forbes 2023-2	Full-text	RCT	Canada	MUDC vs MU	ERCP			245	259	235	259	0.04 (-0.01, 0.08)
Gromski 2021-3	Abstract	NRCS	USA	MUDC vs MU	ERCP			49	49	514	514	0.00 (-0.03, 0.03)
Lee 2023	Abstract	NRCS	South Korea	MUDC vs MU	ERCP			96	101	81	95	0.10 (0.01, 0.18)
SU vs MUDC												
Machado 2023-1	Abstract	NRCS	USA	SU vs MUDC	Oesophageal intubation	51	52	104	104			-0.02 (-0.07, 0.03)
Machado 2023-2	Abstract	NRCS	USA	SU vs MUDC	Duodenal intubation	49	52	104	104			-0.06 (-0.13, 0.01)
Machado 2023-3	Abstract	NRCS	USA	SU vs MUDC	Biliary cannulation/ERCP	45	52	104	104			-0.13 (-0.23, -0.04)
Gromski 2021-1	Abstract	NRCS	USA	SU vs MUDC	ERCP	47	51	49	49			-0.08 (-0.16, 0.00)

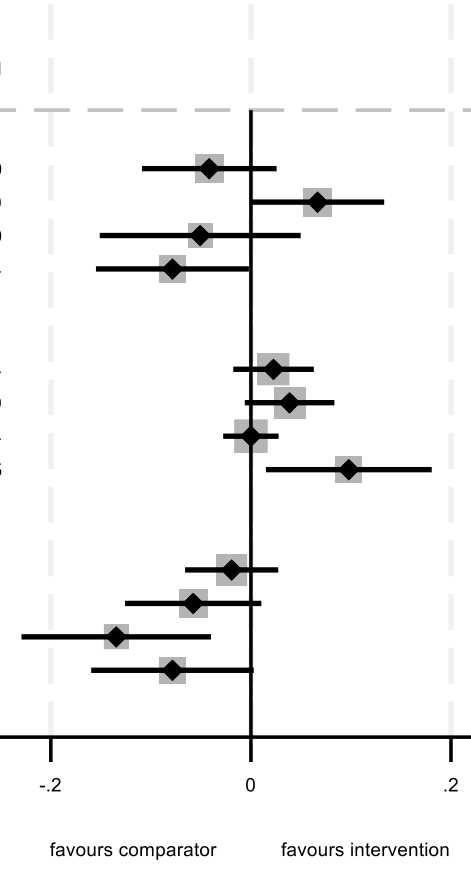

NOTE: Continuity correction applied to studies with zero cells

Comparison details (Intervention vs comparator):
SU vs MU
Bang 2021 SU: EXALT Model D (n=48) vs MU: TJF-180 (n=50)
Gambaccini 2023 SU: EXALT Model D (n=25) vs MU: unspecified (n=150)
Razzak 2023 SU: EXALT Model D (n=53) vs MU: TJF-180 (n=80)
Gromski 2021-2 SU: EXALT Model D (n=51) vs MU: TJF-160/180 (n=514)
MUDC vs MU
Forbes 2023-1 MUDC: ED34-i10T2 (n=241) vs MU: ED34-i10T (n=234)
Forbes 2023-2 MUDC: ED34-i10T2 (n=259) vs MU: ED34-i10T (n=259)
Gromski 2021-3 MUDC: TJF-190 (n=49) vs MU: TJF-160/180 (n=514)
Lee 2023 MUDC: unspecified (n=101) vs MU: unspecified (n=95)
SU vs MUDC
Machado 2023-1 SU: EXALT Model D (n=52) vs MUDC: TJF-Q190V (n=104)
Machado 2023-2 SU: EXALT Model D (n=52) vs MUDC: TJF-Q190V (n=104)
Machado 2023-3 SU: EXALT Model D (n=52) vs MUDC: TJF-Q190V (n=104)
Gromski 2021-1 SU: EXALT Model D (n=51) vs MUDC: TJF-190 (n=49)

Supplementary Figure 2 Successful intubation, cannulation or ERCP procedure for comparisons between SU, MUDC and MU duodenoscopes (all data)

1.2 Sensitivity analyses

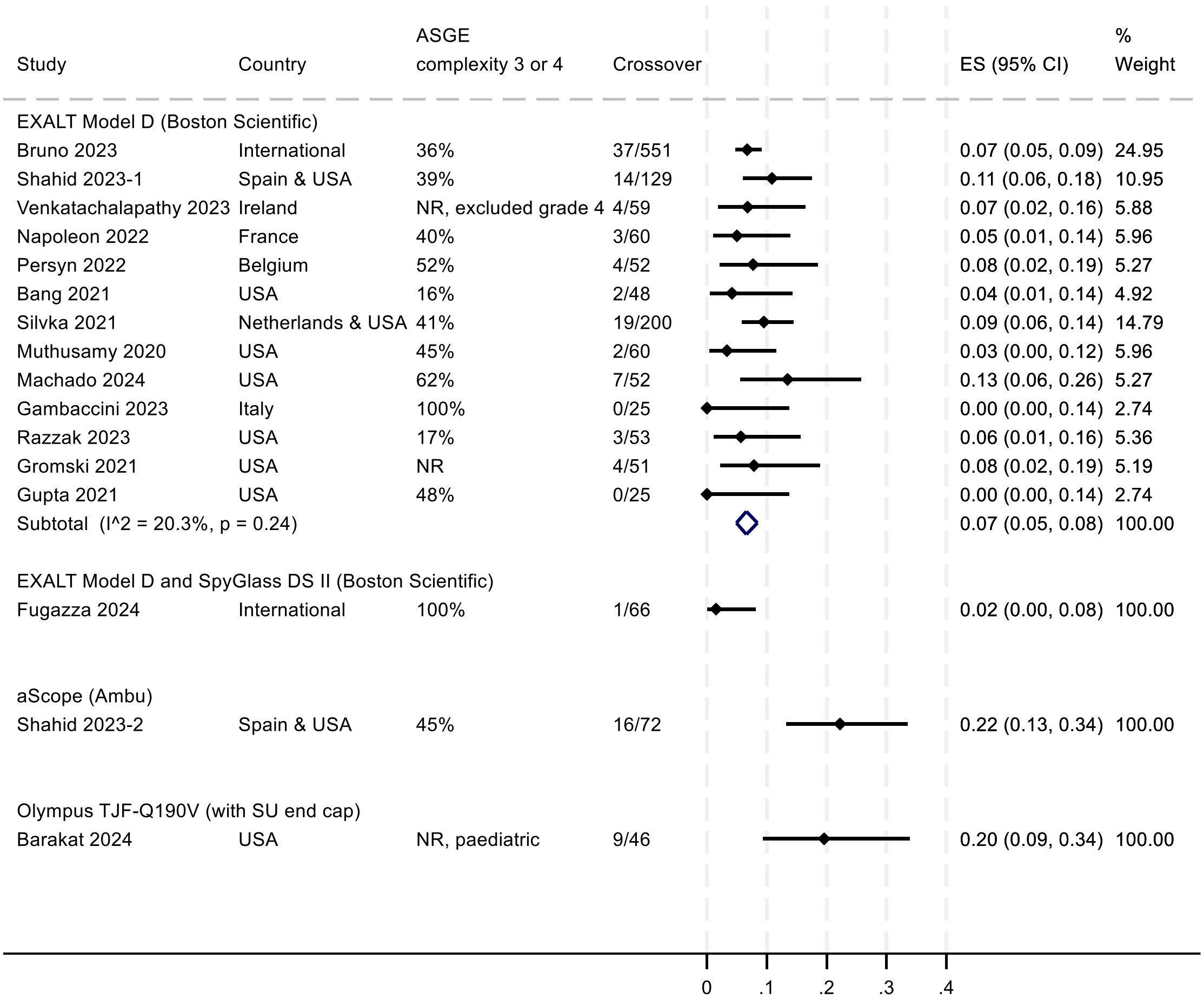

Study	Country	ASGE complexity 3 or 4	Crossover	ES (95% CI)	% Weight
EXALT Model D (Boston Scientific)					
Bruno 2023	International	36%	37/551	0.07 (0.05, 0.09)	24.95
Shahid 2023-1	Spain & USA	39%	14/129	0.11 (0.06, 0.18)	10.95
Venkatachalapathy 2023	Ireland	NR, excluded grade 4	4/59	0.07 (0.02, 0.16)	5.88
Napoleon 2022	France	40%	3/60	0.05 (0.01, 0.14)	5.96
Persyn 2022	Belgium	52%	4/52	0.08 (0.02, 0.19)	5.27
Bang 2021	USA	16%	2/48	0.04 (0.01, 0.14)	4.92
Silvka 2021	Netherlands & USA	41%	19/200	0.09 (0.06, 0.14)	14.79
Muthusamy 2020	USA	45%	2/60	0.03 (0.00, 0.12)	5.96
Machado 2024	USA	62%	7/52	0.13 (0.06, 0.26)	5.27
Gambaccini 2023	Italy	100%	0/25	0.00 (0.00, 0.14)	2.74
Razzak 2023	USA	17%	3/53	0.06 (0.01, 0.16)	5.36
Gromski 2021	USA	NR	4/51	0.08 (0.02, 0.19)	5.19
Gupta 2021	USA	48%	0/25	0.00 (0.00, 0.14)	2.74
Subtotal (I^2 = 20.3%, p = 0.24)				0.07 (0.05, 0.08)	100.00
EXALT Model D and SpyGlass DS II (Boston Scientific)					
Fugazza 2024	International	100%	1/66	0.02 (0.00, 0.08)	100.00
aScope (Ambu)					
Shahid 2023-2	Spain & USA	45%	16/72	0.22 (0.13, 0.34)	100.00
Olympus TJF-Q190V (with SU end cap)					
Barakat 2024	USA	NR, paediatric	9/46	0.20 (0.09, 0.34)	100.00

Supplementary Figure 3 Single-use duodenoscope: crossover rate– sensitivity analysis including conference abstracts

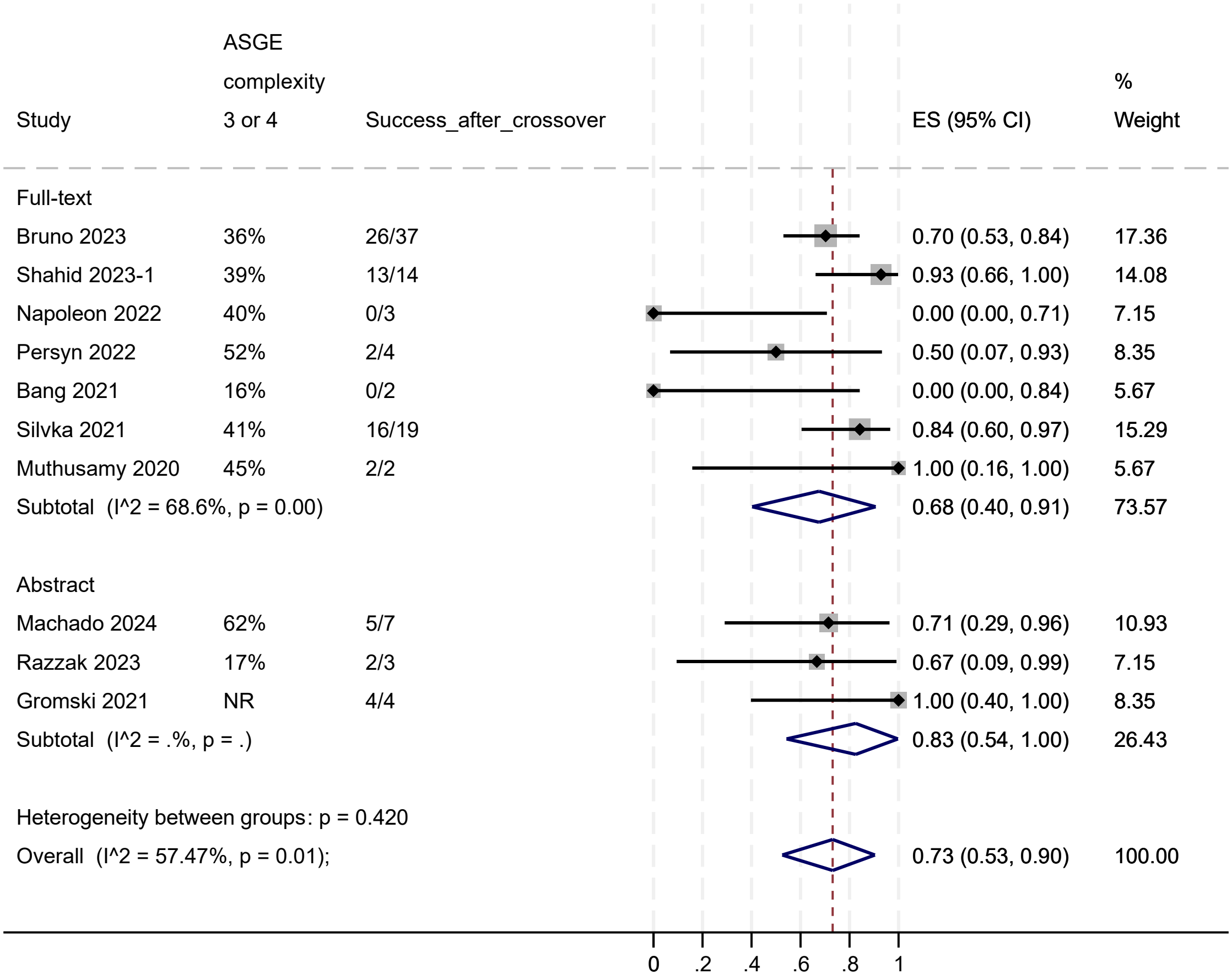

*In Machado 2024, SU (EXALT Model D) was crossed over to MUDC (TJF-Q190V).

Supplementary Figure 4 Single-use duodenoscope: success rate after crossover – sensitivity analysis including conference abstracts

2. Duodenoscopes – selected infections and adverse events

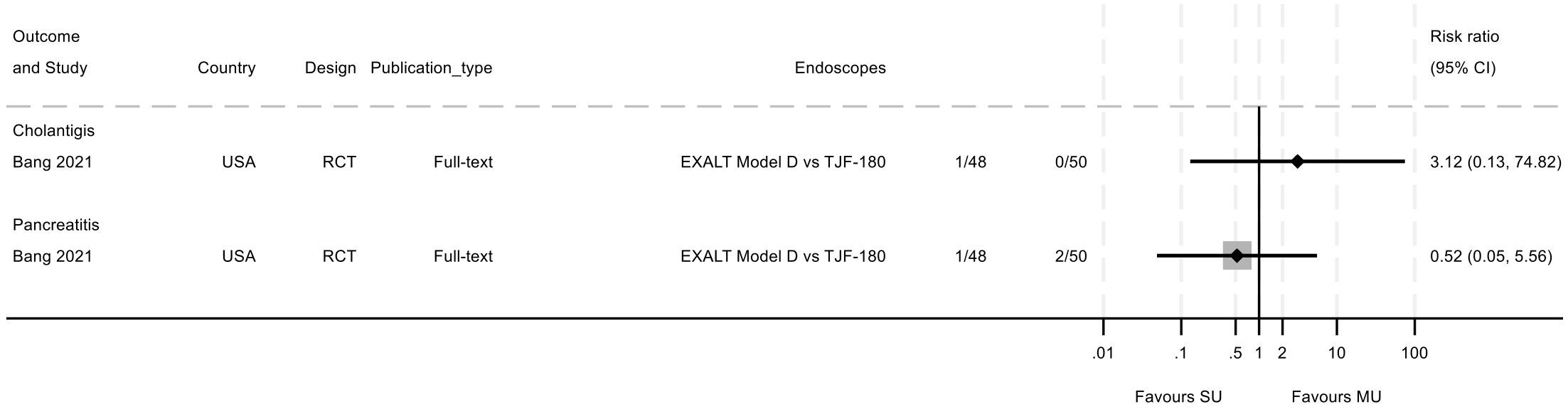

Supplementary Figure 5 Comparative evidence for SU vs MU duodenoscopes – cholangitis & pancreatitis

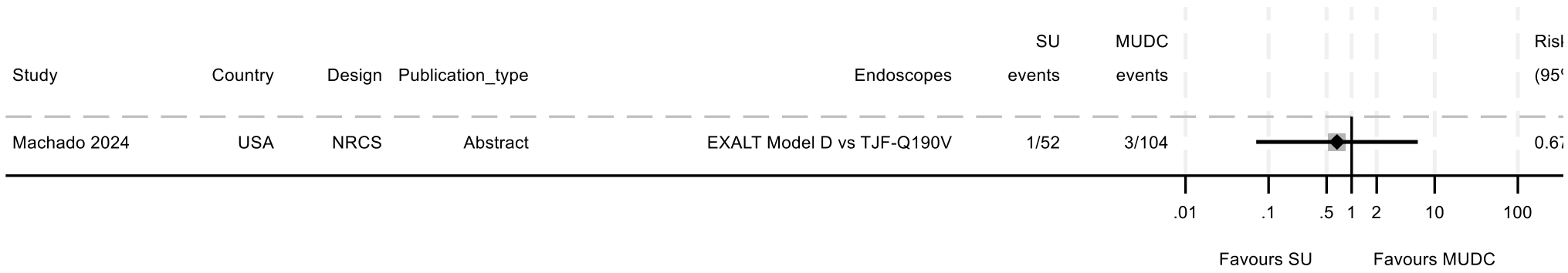

Supplementary Figure 6 Comparative evidence for SU vs MUDC duodenoscopes – pancreatitis

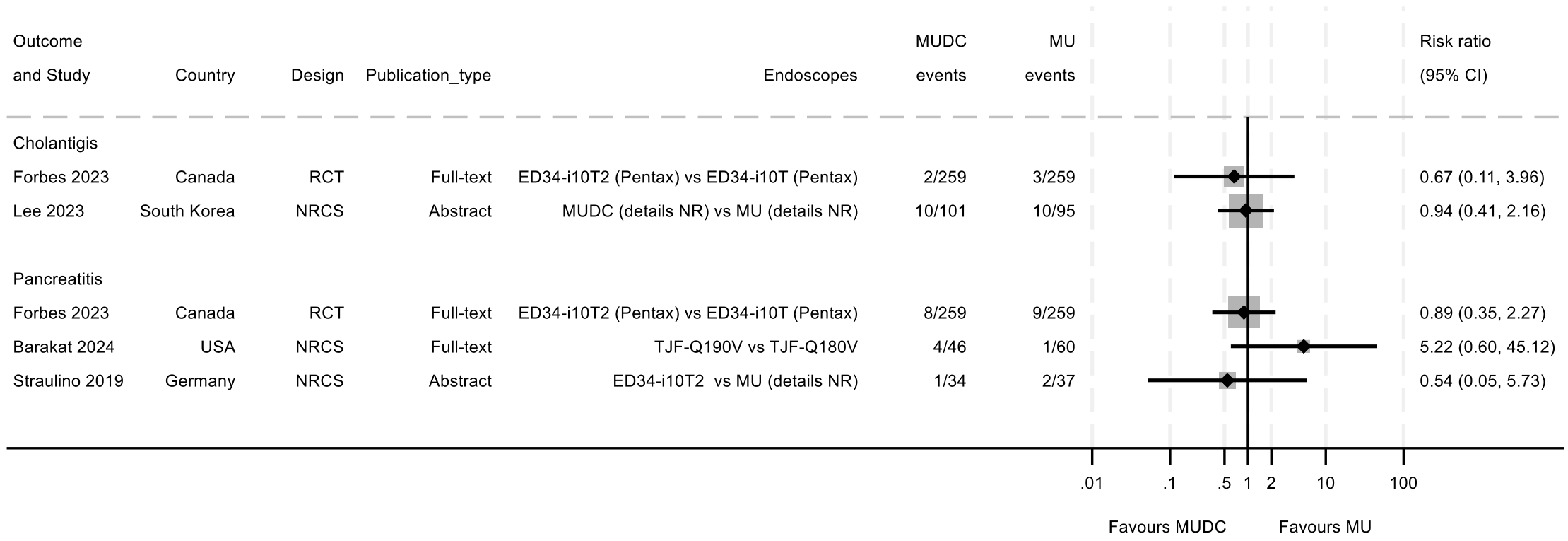

Supplementary Figure 7 Comparative evidence for MUDC vs MU duodenoscopes – cholangitis & pancreatitis

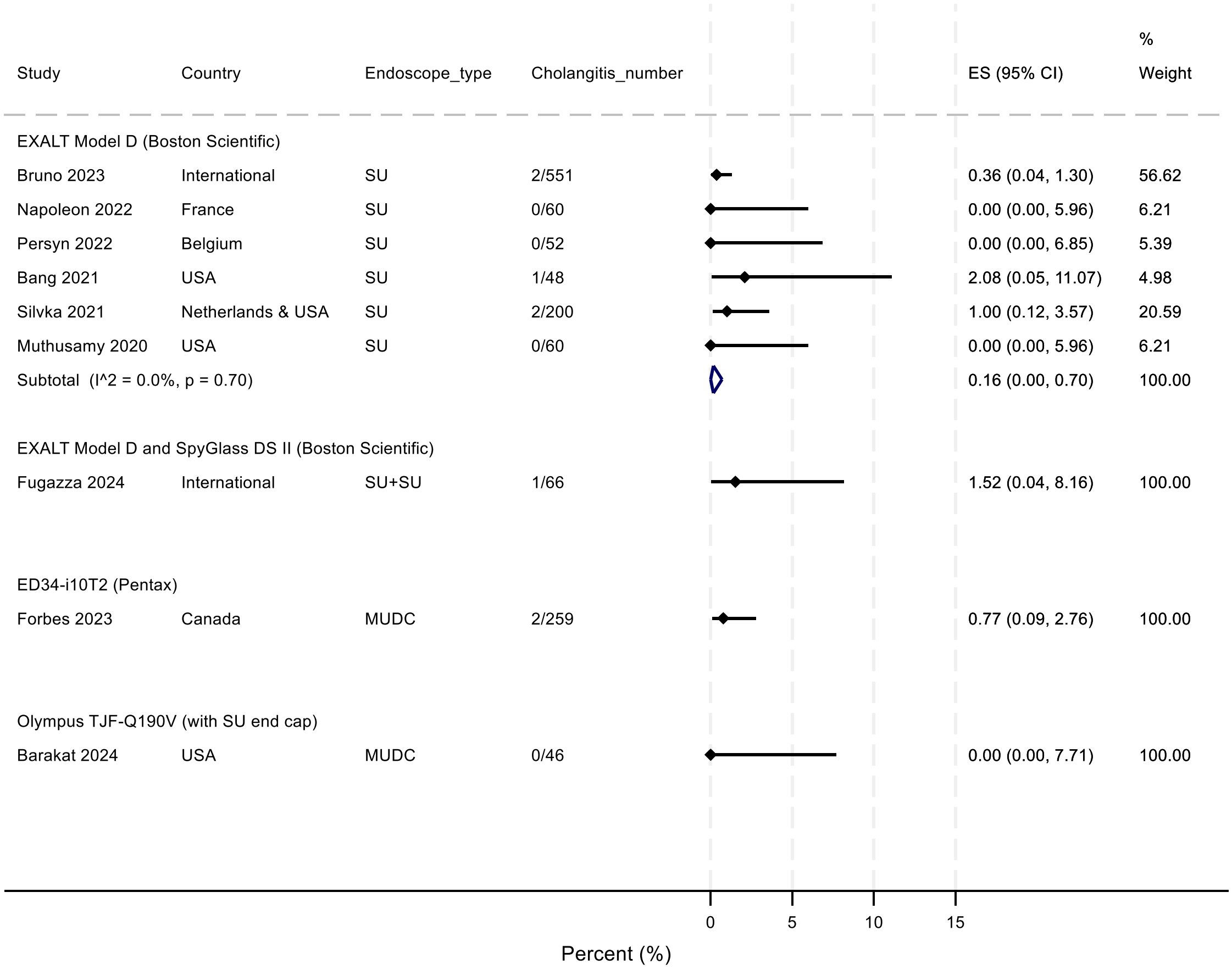

Supplementary Figure 8 Cholangitis - SU and MUDC duodenoscopes – main analysis (full-text only)

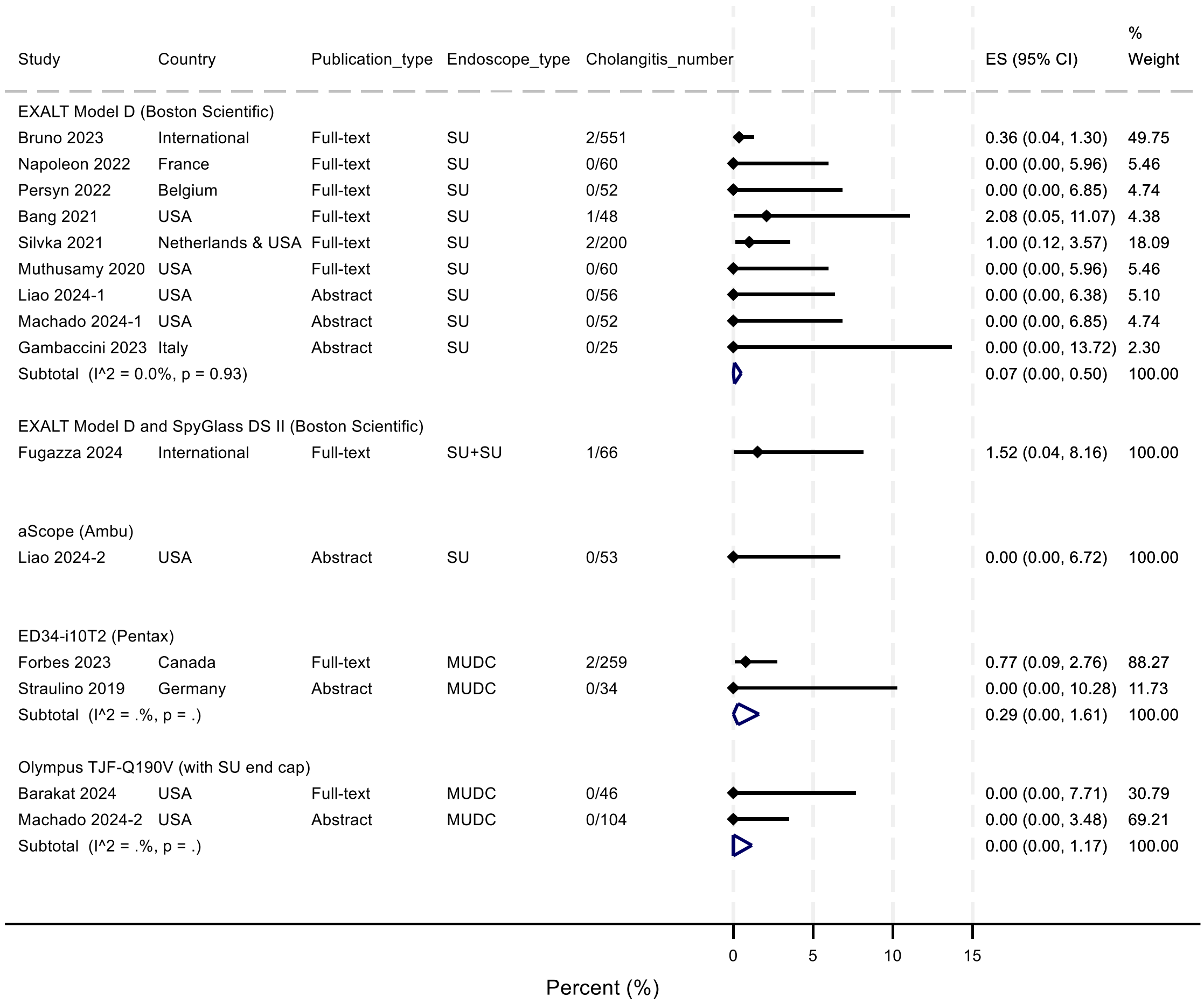

Supplementary Figure 9 Cholangitis - SU and MUDC duodenoscopes – sensitivity analysis (including abstracts)

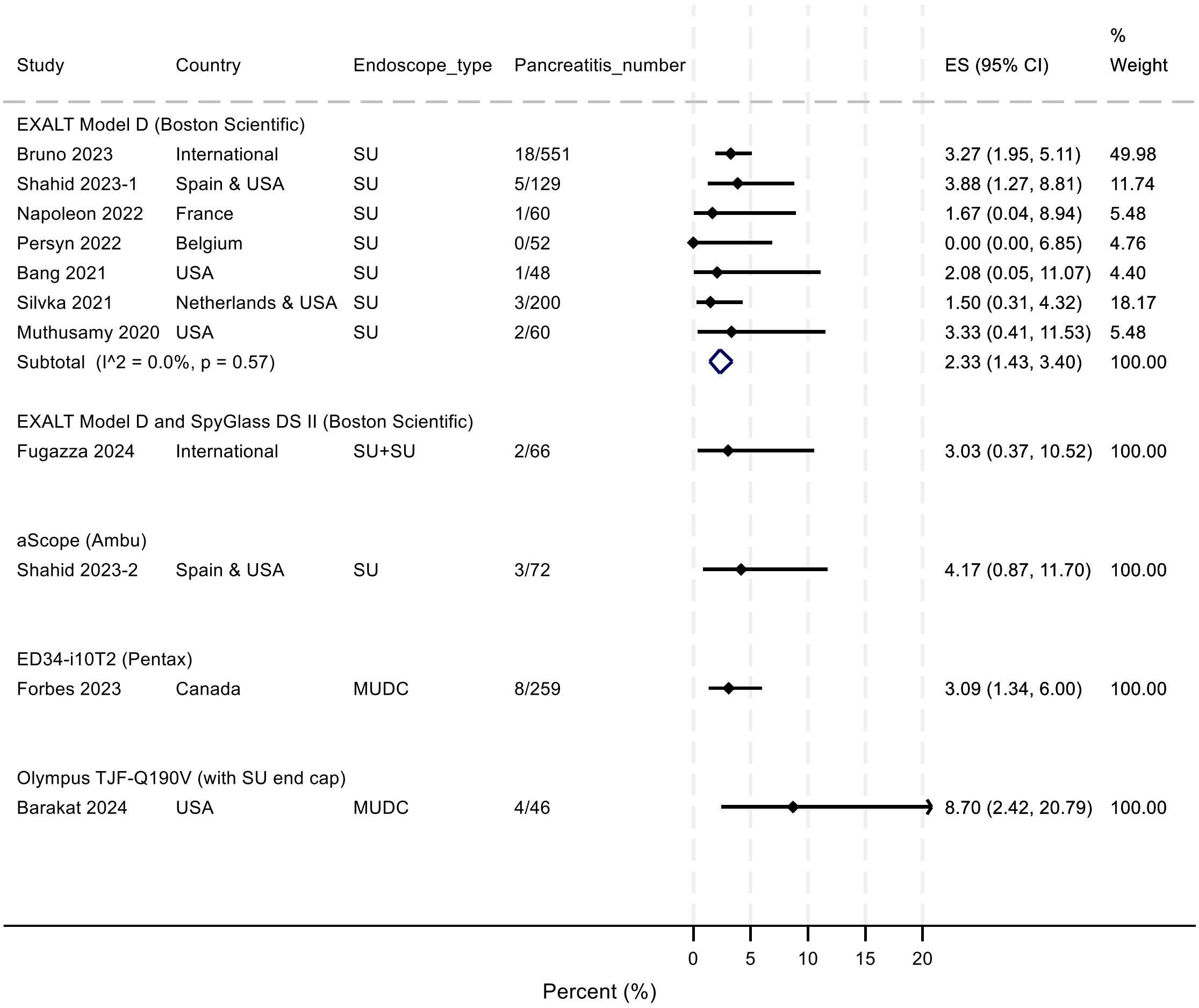

Supplementary Figure 10 Pancreatitis - SU and MUDC duodenoscopes – main analysis (full-text only)

Study	Country	Publication_type	Endoscope_type	Pancreatitis_number	ES (95% CI)	% Weight
EXALT Model D (Boston Scientific)						
Bruno 2023	International	Full-text	SU	18/551	3.27 (1.95, 5.11)	29.32
Shahid 2023-1	Spain & USA	Full-text	SU	5/129	3.88 (1.27, 8.81)	12.62
Napoleon 2022	France	Full-text	SU	1/60	1.67 (0.04, 8.94)	6.83
Persyn 2022	Belgium	Full-text	SU	0/52	0.00 (0.00, 6.85)	6.04
Bang 2021	USA	Full-text	SU	1/48	2.08 (0.05, 11.07)	5.63
Silvka 2021	Netherlands & USA	Full-text	SU	3/200	1.50 (0.31, 4.32)	17.14
Muthusamy 2020	USA	Full-text	SU	2/60	3.33 (0.41, 11.53)	6.83
Liao 2024-1	USA	Abstract	SU	0/56	0.00 (0.00, 6.38)	6.44
Machado 2024-1	USA	Abstract	SU	1/52	1.92 (0.05, 10.26)	6.04
Gambaccini 2023	Italy	Abstract	SU	3/25	12.00 (2.55, 31.22)	3.13
Subtotal (I^2 = 21.5%, p = 0.24)					2.05 (1.02, 3.33)	100.00
EXALT Model D and SpyGlass DS II (Boston Scientific)						
Fugazza 2024	International	Full-text	SU+SU	2/66	3.03 (0.37, 10.52)	100.00
aScope (Ambu)						
Shahid 2023-2	Spain & USA	Full-text	SU	3/72	4.17 (0.87, 11.70)	57.54
Liao 2024-2	USA	Abstract	SU	0/53	0.00 (0.00, 6.72)	42.46
Subtotal (I^2 = .%, p = .)					1.69 (0.01, 5.09)	100.00
ED34-i10T2 (Pentax)						
Forbes 2023	Canada	Full-text	MUDC	8/259	3.09 (1.34, 6.00)	88.27
Straulino 2019	Germany	Abstract	MUDC	1/34	2.94 (0.07, 15.33)	11.73
Subtotal (I^2 = .%, p = .)					2.64 (0.91, 5.02)	100.00
Olympus TJF-Q190V (with SU end cap)						
Barakat 2024	USA	Full-text	MUDC	4/46	8.70 (2.42, 20.79)	30.79
Machado 2024-2	USA	Abstract	MUDC	3/104	2.88 (0.60, 8.20)	69.21
Subtotal (I^2 = .%, p = .)					4.23 (1.37, 8.29)	100.00

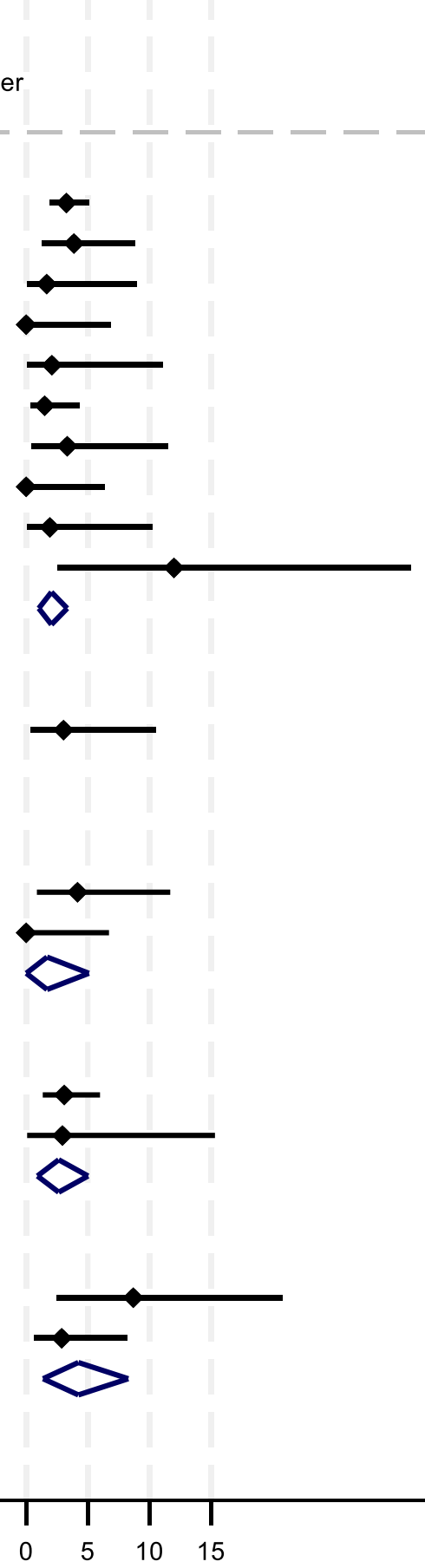

Percent (%)

Supplementary Figure 11 Pancreatitis - SU and MUDC duodenoscopes – sensitivity analysis (including abstracts)

3. Other upper GI endoscopes – technical performance

Study	Publication type	Design	Country	Comparison	SU_success	SU_total	MU_success	MU_total	Difference in success rates (95% CI)
Wang 2024	Abstract	RCT	China	SU vs MU	20	21	20	21	0.00 (-0.13, 0.13)
Römmele 2024-1	Abstract	RCT	Germany	SU vs MU	47	47	45	45	0.00 (-0.04, 0.04)
Römmele 2024-2	Abstract	RCT	Germany	SU vs MU	19	22	24	24	-0.10 (-0.24, 0.05)

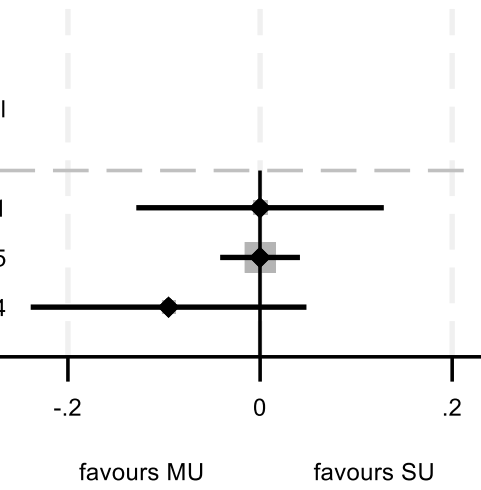

NOTE: Continuity correction applied to studies with zero cells

Supplementary Figure 12 Comparative evidence for procedure completion/success rates for SU and partially SU vs MU upper GI endoscopes (all data)

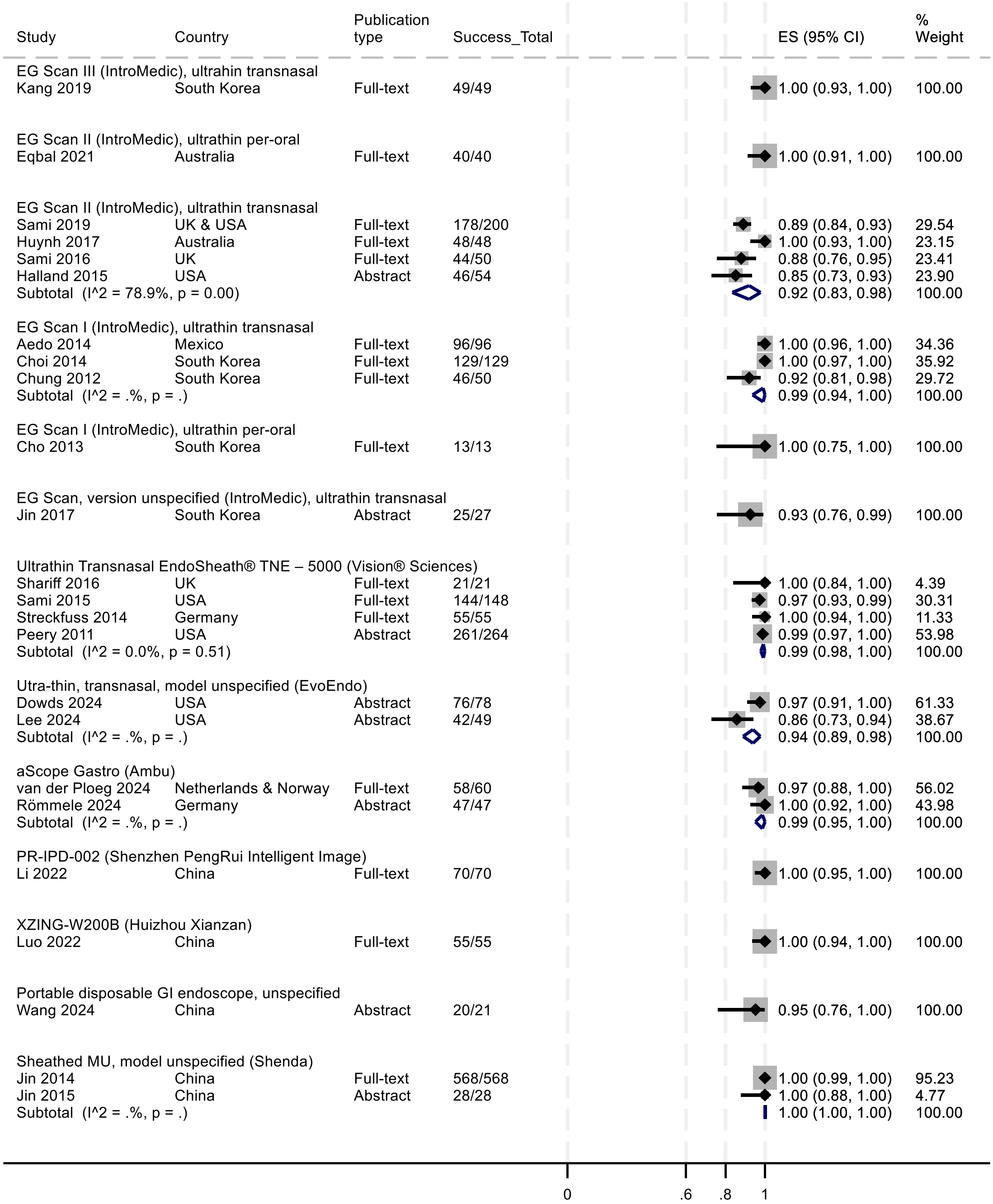

Supplementary Figure 13 Procedure completion/success rates for SU and partially SU upper GI endoscopes (all data)

4. Endoscopes for lower GI tract – technical performance

Study	Publication type	Design	Country	Comparison	Endoscope type	Outcome	SU_success	SU_total	MU_success	MU_total	Difference in success rates (95% CI)
Wei 2022	Full-text	RCT	China	SU vs MU	Colonoscope	Caecal intubation	45	45	45	45	0.00 (-0.04, 0.04)
Xu 2024	Full-text	RCT	China	SU vs MU	Gastroscope for treating internal haemorrhoid	Endoscopic rubber band ligation	21	21	21	21	0.00 (-0.09, 0.09)
Xu 2024	Full-text	RCT	China	SU vs MU	Gastroscope for treating internal haemorrhoid	Endoscopic mucosal resection	19	19	17	17	0.00 (-0.10, 0.10)

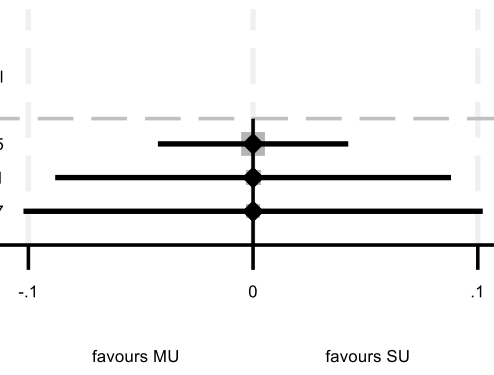

NOTE: Continuity correction applied to studies with zero cells

Supplementary Figure 14 Comparative evidence for procedure completion/success rates for SU vs MU endoscopes for lower GI endoscopic procedures (all data)
