# Supplementary material for: Diagnostic and Clinical Performance of Single‐Use Versus Reusable Gastrointestinal Endoscopes: A Systematic Review and Meta‐Analyses
Source: J Gastroenterol Hepatol. 2026 Aug 7;41(9):2571–87. doi: 10.1111/jgh.70637 (PMC13534234; doi:10.1111/jgh.70637)
Supplement: Supplementary file 2 [file JGH-41-2571-s002.pdf]

Diagnostic and clinical performance of single-use versus reusable gastrointestinal endoscopes: a systematic review and meta-analyses

Supporting information Table S3: Types of endoscope and study design of included studies

Authors

Natalie Tyldesley-Marshall, Yen-Fu Chen,* Jefferson Buendia, Anna Brown, Thomas Matthews, Janette Parr, Shivashri Chockalingam, Adel Elfeky, Amy Grove, Lazaros Andronis, Stuart R Coles, Mandana Zanganeh, Bu Hayee, Anjan Dhar, Shaji Sebastian, Ramesh Arasaradnam on behalf of the SUMU-Endo Project

*Correspondence to: Prof. Yen-Fu Chen

Centre for Evidence and Implementation Science, Park House, Edgbaston, University of Birmingham, Birmingham B15 2TT, United Kingdom

y.chen.25@bham.ac.uk

Department of Health and Welfare, College of City Management, University of Taipei, Taipei, Taiwan

yfchen@utaipei.edu.tw

Table S3. Types of endoscope and study design of included studies

	Duodenoscopes / endoscopes for ERCP procedure	Other upper GI endoscopes	Endoscopes for lower GI tract	More than one types of GI endoscopes	Total
**Total**	**23 (1*)**	**24 (1*)**	**2 (1*)**	**1**	**50**
Full-text	12 (1*)	16 (1*)	2 (1*)	1	31
Conference abstract	11	8	0	0	19
**Comparative evidence**	**15**	**17**	**2**	**0**	**34**
Fully paired diagnostic accuracy studies	0	10	0	0	10
Randomised controlled trials	2	6	2	0	10
Non-randomised comparative studies	13	1	0	0	14
**Non-comparative evidence**	**8 (1*)**	**7 (1*)**	**0 (1*)**	**1**	**16**
Systematic review	1 (1*)	0 (1*)	0 (1*)	1	2
Case series	7	7	0	0	14

*Additional study covering multiple types of GI endoscopes (i.e. those shown in the "More than one types of GI endoscopes" column) that also contributed data towards the specific type of endoscopes.
