# Supplementary material for: Diagnostic and Clinical Performance of Single‐Use Versus Reusable Gastrointestinal Endoscopes: A Systematic Review and Meta‐Analyses
Source: J Gastroenterol Hepatol. 2026 Aug 7;41(9):2571–87. doi: 10.1111/jgh.70637 (PMC13534234; doi:10.1111/jgh.70637)
Supplement: Supplementary file 3 [file JGH-41-2571-s003.pdf]

Diagnostic and clinical performance of single-use versus reusable gastrointestinal endoscopes: a systematic review and meta-analyses

Supporting information Table S2: Reasons for exclusion at full-text stage

Authors

Natalie Tyldesley-Marshall, Yen-Fu Chen,* Jefferson Buendia, Anna Brown, Thomas Matthews, Janette Parr, Shivashri Chockalingam, Adel Elfeky, Amy Grove, Lazaros Andronis, Stuart R Coles, Mandana Zanganeh, Bu Hayee, Anjan Dhar, Shaji Sebastian, Ramesh Arasaradnam on behalf of the SUMU-Endo Project

*Correspondence to: Prof. Yen-Fu Chen

Centre for Evidence and Implementation Science, Park House, Edgbaston, University of Birmingham, Birmingham B15 2TT, United Kingdom

y.chen.25@bham.ac.uk

Department of Health and Welfare, College of City Management, University of Taipei, Taipei, Taiwan

yfchen@utaipei.edu.tw

Table S2. Reasons for exclusion at full-text stage

Authors	Year	Title of publication	Reason for exclusion
Abudayyeh, S.; Hoffman, J.; El-Zimaity, H. T.; Graham, D. Y.	2009	Prospective, randomized, pathologist-blinded study of disposable alligator-jaw biopsy forceps for gastric mucosal biopsy	Wrong intervention
Aerts, M.; Reynaert, H.; Roelandt, P.; Caenepeel, P.; Dewint, P.; Lemmers, A.; Colle, I.	2024	Position statement on how can we can implement the Greendeal in our gastrointestinal and gastrointestinal endoscopy department in Belgium	Wrong outcomes
Agrawal, D.; Rockey, D. C.	2012	Disposal of endoscopic accessories do we know, do we care, do we care to know? A survey of endoscopy nurses and technicians	Wrong publication type
Agrawal, D.; Shoup, V.; Montgomery, A.; Wosik, J.; Rockey, D. C.	2017	Disposal of Endoscopic Accessories After Use: Do We Know and Do We Care?	Wrong intervention
Agrawal, D.; Tang, Z.	2021	Sustainability of Single-Use Endoscopes	Wrong outcomes
Albert, J. G.; Lucas, K.; Filmann, N.; Herrmann, E.; Schroder, O.; Trojan, J.; Sarrazin, C.; Euzem, S.; Friedrich-Rust, M.	2014	A randomized trial of guidewire use for biliary stricture cannulation in endoscopic retrograde cholangiopancreatography (ERCP): Superior success rate of a new single use guidewire	Wrong publication type
Ali, B.; Keihanian, T.; Abidi, W.	2022	Feasibility of a New Generation Single Use Duodenoscope in a Patient With Roux-en-Y Gastric Bypass	Wrong publication type
Ali, R.; Diehl, D.; Shieh, F.; Fischer, M.; Tamura, W.; Rubin, D.; Storm, A.; Okolo, P.; Kumbhari, V.; Wild, D.; Halpern, Z.; Neumann, H.; Pochapin, M.; Gross, S.	2013	Deep enteroscopy with a conventional colonoscope: Initial multicenter study using the naviaid balloon system	Wrong comparator
Ali, R.; Wild, D.; Shieh, F.; Diehl, D. L.; Fischer, M.; Tamura, W.; Rubin, D. T.; Kumbhari, V.; Okolo, P.; Storm, A.; Halpern, Z.; Neumann, H.; Khara, H. S.; Pochapin, M. B.; Gross, S. A.	2015	Deep enteroscopy with a conventional colonoscope: initial multicenter study by using a through-the-scope balloon catheter system	Wrong intervention
Allen, J. W.; Hahm, T. X.; Polk, H. C., Jr.	2003	Surgeon-led initiatives cut costs and enhance the quality of endoscopic and laparoscopic procedures	Wrong comparator
Allen, T. W.	1993	Infection transmitted by endoscopy and bronchoscopy	Wrong study design
Almario, C. V.; May, F. P.; Shaheen, N. J.; Murthy, R.; Gupta, K.; Jamil, L. H.; Lo, S. K.; Spiegel, B. M. R.	2015	Cost Utility of Competing Strategies to Prevent Endoscopic Transmission of Carbapenem-Resistant Enterobacteriaceae	Wrong outcomes
Anastassiades, C. P.; Salah, W.; Pauli, E. M.; Marks, J.; Chak, A.	2012	Cap-fitted, forward-viewing endoscope for therapeutic ERCP in patients with a Billroth II gastrectomy	Wrong publication type
Anderloni, A.; Di Leo, M.; Mutignani, M.; Dabizzi, E.; Tarantino, I.; Maselli, R.; Repici, A.	2016	First national registry on a new disposable cholangioscope for single operator cholangioscopy	Wrong intervention
Anonymous	2024	51. Jahrestagung der Gesellschaft fur Gastroenterologie in Bayern e. V.	Duplicate
Anonymous,	2023	Gastrointestinal health and the environment: a bidirectional relationship	Wrong study design
Anonymous,	2023	NASPGHAN Annual Meeting Abstracts	Wrong study design
Asayama, N.; Oka, S.; Tanaka, S.; Sumimoto, K.; Hirano, D.; Tamaru, Y.; Ninomiya, Y.; Shigita, K.; Hayashi, N.; Nishiyama, S.; Chayama, K.	2016	Clinical usefulness of a single-use splinting tube for poor endoscope operability in deep colonic endoscopic submucosal dissection	Wrong intervention
Asghar, M.; Khan, Q.; Dhillon, S.; Puli, S.	2024	SINGLE USE DUODENOSCOPE PERFORMANCE OUTCOMES IN ENDOSCOPIC RETROGRADE CHOLANGIOPANCREATOGRAPHY: A SYSTEMATIC REVIEW AND META-ANALYSIS	Systematic review for checking only

Azab, M.; Kam, K.; Lin, E.; Pham, J.; Oh, N.; Wright, A.	2020	The Costs of Guideline Adherence: A Retrospective Evaluation of the Economic Impact of Snare vs Forceps Removal of Diminutive Colon Polyps in an Academic Medical Center	Wrong comparator
Baddeley, R.; Aabakken, L.; Veitch, A.; Hayee, B.	2022	Green Endoscopy: Counting the Carbon Cost of Our Practice	Wrong outcomes
Baddeley, R.; Aabakken, L.; Veitch, A.; Hayee, B.	2022	Single-Use Duodenoscopes	Wrong study design
Baddeley, R.; de Santiago, E. R.; Maurice, J.; Siddhi, S.; Dhar, A.; Thomas-Gibson, S.; Hayee, B.	2022	Sustainability in gastrointestinal endoscopy	Wrong outcomes
Baeg, M. K.; Lim, C. H.; Kim, J. S.; Cho, Y. K.; Park, J. M.; Lee, B. I.; Lee, I. S.; Choi, M. G.	2016	Portable disposable ultrathin endoscopy tested through percutaneous endoscopic gastrostomy	Wrong intervention
Bakis, G.; Howell, D. A.; Wright, E.; Bosco, J. J.; Stefan, A. M.	2010	ERCP catheter based intraductal endoscopy: Indications and initial experience	Wrong publication type
Bal, P. J.	1984	Decontamination of gastroscopes: apropos of the efficacy and the cost	Foreign language
Baliga, C.; Ross, A.; Furman, C.; Gluck, M.	2014	The spread of multi-drug resistant bacteria following ERCP: Time to redesign the duodenoscope or enhance reprocessing?	Wrong outcomes
Bang, J. Y.; Rex, D. K.; Sherman, S.; Webb, D.; Sieber, M.; Schmitt, B.; Kressel, A.; Easler, J. J.; McHenry, L.; El Hajj,, II; Fogel, E.; Watkins, J. L.; Lehman, G. A.	2016	Impact of Implementation of Double-Reprocessing Protocol in the Prevention of Duodenoscope-Associated Carbapenem-Resistant Enterobacteriaceae (CRE) in a Single Tertiary Referral Center	Wrong outcomes
Bang, J. Y.; Rosch, T.; Robalino Gonzaga, E. S.; Tharian, B.; Inamdar, S.; Perisetti, A.; Thakkar, S.; Yachimski, P.; Jamidar, P. A.; Muniraj, T.; Sethi, A.; Draganov, P. V.; Varadarajulu, S.	2020	TECHNICAL EVALUATION OF DUODENOSCOPE PERFORMANCE USING A NEWLY DEVELOPED ASSESSMENT TOOL	Wrong outcomes
Bang, J. Y.; Sutton, B.; Hawes, R.; Varadarajulu, S.	2019	Concept of disposable duodenoscope: at what cost?	Wrong outcomes
Bapaye, J.; Chandan, S.; Naing, L. Y.; Shehadah, A.; Deliwala, S.; Bhalla, V.; Chathuranga, D.; Okolo, P. I., 3rd	2022	Safety and efficacy of over-the-scope clips versus standard therapy for high-risk nonvariceal upper GI bleeding: systematic review and meta-analysis	Wrong intervention
Barakat, M. T.; Ghosh, S.; Banerjee, S.	2022	Cost utility analysis of strategies for minimizing risk of duodenoscope-related infections	Wrong outcomes
Barakat, M. T.; Liman, A.; Gugig, R.	2024	Initial experience with duodenoscopes with single-use end caps in pediatric ERCP: infection prevention comes at a cost	Duplicate
Barakat, M.; Ghosh, S.; Banerjee, S.	2020	775 COST UTILITY ANALYSIS COMPARING DUODENOSCOPE REPROCESSING/STERILIZATION, NOVEL DUODENOSCOPES WITH DISPOSABLE ENDCAPS AND FULLY DISPOSABLE DUODENOSCOPES	Wrong outcomes
Barkun, A.; Liu, J.; Carpenter, S.; Chotiprasidhi, P.; Chuttani, R.; Ginsberg, G.; Hussain, N.; Silverman, W.; Taitelbaum, G.; Petersen, B. T.; Technology Assessment, Comm	2006	Update on endoscopic tissue sampling devices	Wrong study design
Bartles, R. L.; Leggett, J. E.; Hove, S.; Kashork, C. D.; Wang, L.; Oethinger, M.; Baxter, L.; Brandabur, J. J.	2018	A randomized trial of single versus double high-level disinfection of duodenoscopes and linear echoendoscopes using standard automated reprocessing	Wrong outcomes
Baxter, L.; Brandabur, J.	2016	Risk and Opportunity: A Large Health System Surveys Its Duodenoscope and Linear Echoendoscope Reprocessing "First, Do No Harm"	Wrong outcomes
Bazarbashi, A. N.; McCarty, T. R.; Al-Obaid, L. N.; Skinner, M. J.; Thompson, C. C.	2019	Efficacy and Safety of Novel, Disposable Endoscopic Scissors in Patients with Roux-en-Y Gastric Bypass: a Single-Center Feasibility Study	Wrong intervention
Beilenhoff, U.	2015	ERCP and reprocessing in focus: what can we do to prevent or manage infection outbreaks?	Wrong outcomes

Beyna, T.; Arvanitakis, M.; Schneider, M.; Van Gossum, A.; Deviere, J.; Neuhaus, H.	2018	Motorized spiral enteroscopy: Final results a two-center prospective clinical trial	Wrong intervention
Beyna, T.; Arvanitakis, M.; Schneider, M.; Van Gossum, A.; Deviere, J.; Neuhaus, H.	2016	Novel motorized spiral endoscopy: First results of a european prospective trial	Wrong intervention
Bhenswala, P.; Andalib, I.; Gress, F.	2021	Preliminary clinical evaluation of a novel single use duodenoscope: A single center experience	Sample too small
Bichard, P.; Auroux, J.; Chelbabi, F.; Fior-Gozlan, M.; Germain, E.; Leroy, V.; Zarski, J. P. H.	2009	Combining analysis of per ERCP bile aspiration and endobiliary brushing improve the diagnostic yield of cytology in malignant strictures of the biliary tract. mono-centric prospective study	Wrong intervention
Bilal, M.; Berzin, T. M.; Cohen, J.; Sawhney, M. S.; Pleskow, D. K.	2020	ERCP in patients with COVID-19 infection - is a single-use duodenoscope the safer option?	Not empirical
Billi, P.; Casadei, A.; Bianchi, S.; Grementieri, P.; Cortini, C.; D'Imperio, N.; Maiolo, P.	2002	Disposable versus reusable biopsy forceps in gastrointestinal endoscopy: A prospective, randomized, cost-effectiveness evaluation	Wrong publication type
Blaut, U.; Marecik, J.; Gniady, J.; Ejma-Multanski, J.; Thor, P. J.	2005	The use of endoscopic Doppler probe - Case series report	Wrong study design
Blosser, R.; Blosser, A.	2021	The feasibility of use of the exalt model-d single use duodenoscope for ERCP performed in an outpatient ambulatory center	Sample too small
Bomman, S.; Ashat, M.; Nagra, N.; Jayaraj, M.; Chandra, S.; Kozarek, R. A.; Ross, A.; Krishnamoorthi, R.	2022	Contamination Rates in Duodenoscopes Reprocessed Using Enhanced Surveillance and Reprocessing Techniques: A Systematic Review and Meta-Analysis	Wrong outcomes
Bonisa, K.; Neelakanta, A.; Layell, J.; McLain, J.; Lana, H.; Betts, W.; Kester, S.; Passaretti, C.	2019	Endoscopic retrograde cholangiopancreatography (ERCP)-associated carbapenem-resistant enterobacteriaceae (CRE) before and after implementation of ethylene oxide (ETO) sterilization of duodenoscopes	Wrong outcomes
Bornschlegel, K.; Dentinger, C.; Layton, M.; Balter, S.; France, A. M.	2012	Investigation of viral hepatitis infections possibly associated with health-care delivery - New York City, 2008-2011	Wrong outcomes
Boston Scientific Corporation	2019	Initial Case Series With Exalt Single-Use Duodenoscope	Not empirical
Bourguignon, C.; Destrumelle, A. S.; Koch, S.; Grumblat, A.; Carayon, P.; Chopard, C.; Woronoff-Lemsi, M. C.	2003	Disposable versus reusable biopsy forceps in GI endoscopy: a cost-minimization analysis	Wrong intervention
Bourigault, C.; Le Gallou, F.; Bodet, N.; Musquer, N.; Juvin, M. E.; Corvec, S.; Ferronniere, N.; Wiesel, S.; Gournay, J.; Birgand, G.; Le Rhun, M.; Lepelletier, D.	2018	Duodenoscopy: an amplifier of cross-transmission during a carbapenemase-producing Enterobacteriaceae outbreak in a gastroenterology pathway	Wrong outcomes
Bozzi, R.; Inzirillo, A.; D'Avenia, E.; Inzirillo, M.; Dewerra, C.; Cattaneo, D.	2013	Endotics system, a new way to undergo the endoscopic valuation of lower GI tract : Our initial experience	Sample too small
Bretthauer, M.; Hoff, G.; Thiis-Evensen, E.; Grotmol, T.; Larsen, I. K.; Kjellevold, O.; Skovlund, E.	2002	Use of a disposable sheath system for flexible sigmoidoscopy in decentralized colorectal cancer screening	Wrong intervention
Broide, E.; Shalem, T.; Richter, V.; Matalon, S.; Shirin, H.	2020	The Safety and Feasibility of a New Through-the-scope Balloon-assisted Enteroscopy in Children	Wrong intervention
Bruno, M. J.; Beyna, T.; Carr-Locke, D.; Chahal, P.; Costamagna, G.; Devereaux, B.; Giovannini, M.; Goenka, M. K.; Khor, C.; Lau, J.; May, G.; Muthusamy, V. R.; Patel, S.; Petersen, B. T.; Pleskow, D. K.; Raijman, I.; Reddy, D. N.; Repici, A.; Ross, A. S.; Sejpal, D. V.; Sherman, S.; Siddiqui, U. D.; Ziady, C.; Peetermans, J. A.; Rousseau, M. J.; Slivka, A.	2023	Global prospective case series of ERCPs using a single-use duodenoscope	Duplicate

Bruno, M.; Bosco, M.; Carucci, P.; Pacchioni, D.; Repici, A.; Mezzabotta, L.; Pellicano, R.; Fadda, M.; Saracco, G. M.; Bussolati, G.; Rizzetto, M.; De Angelis, C.	2009	Preliminary experience with a new cytology brush in EUS-guided FNA	Wrong intervention
Bulut, M.; Hjorne, F.; Knuhtsen, S.; Stigaard, T.; Bremholm Hansen, L.	2022	Sterile laparoscopic transgastric ERCP with single-use disposable duodenoscope	Wrong study design
Burdick, J. S.; Hambrick, D.	2004	Endoscope reprocessing and repair costs	Wrong study design
Cai, M. Y.; Zhu, L.; Xu, X. Y.; Xu, J. X.; Zhang, D. F.; Zhang, Z.; Li, Q. L.; Qin, W. Z.; Feng, L.; Xu, J. G.; Li, P.; Zhou, P. H.	2023	Endoscopic mucosal resection of gastrointestinal polyps with a novel low-temperature plasma radio frequency generator: a non-inferiority multi-center randomized control study	Wrong intervention
Cai, M. Y.; Zhu, L.; Xu, X.; Li, P.; Feng, L.; Xu, J.; Zhou, P. H.	2022	ENDOSCOPIC MUCOSAL RESECTION OF GASTROINTESTINAL POLYPS WITH A NOVEL PLASMA RADIOFREQUENCY GENERATOR-A NON-INFERIORITY MULTICENTER RANDOMIZED CONTROL STUDY	Wrong publication type
Cai, M.; Zhu, L.; Xu, X. Y.; Xu, J. X.; Zhang, D. F.; Zhang, Z.; Li, Q. L.; Qin, W. Z.; Feng, L.; Li, P.; Xu, J. G.; Zhou, P. H.	2022	ENDOSCOPIC MUCOSAL RESECTION OF GASTROINTESTINAL POLYPS WITH A NOVEL PLASMA RADIOFREQUENCY GENERATOR: A NON-INFERIORITY MULTICENTERRANDOMIZEDCONTROL STUDY	Wrong publication type
Camp, R.; Rutkowski, M. A.; Atkison, K.; Niedzwick, L.; Vakil, N.	1992	A prospective, randomized, blinded trial of cytological yield with disposable cytology brushes in upper gastrointestinal tract lesions	Too early
Canard, J. M.; Cellier, C.; Houcke, P.; Laurent, J.; Gorce, D.; Landi, B.	2000	Prospective multicenter study comparing a standard reusable sphincterotome with a disposable triple-lumen sphincterotome	Too early
Canard, J. M.; Debette-Gratien, M.; Dumas, R.; Escourrou, J.; Gay, G.; Giovannini, M.; Greff, M.; Grimaud, J. C.; Helbert, T.; Marchetti, B.; Lapuelle, J.; Napoleon, B.; Palazzo, L.; Ponchon, T.; Rey, J. F.; Sautereau, D.	2005	A prospective national study on colonoscopy and sigmoidoscopy in 2000 in France	Wrong comparator
Cannistra, F.	1996	[Emergency endoscopic treatment of digestive hemorrhages of the gastroduodenal tract (Forrest 1a, 1b)]	Too early
Casini, B.; Spagnolo, A. M.; Sartini, M.; Tuvo, B.; Scarpaci, M.; Barchitta, M.; Pan, A.; Agodi, A.; Cristina, M. L.; Study, Grp	2023	Microbiological surveillance post-reprocessing of flexible endoscopes used in digestive endoscopy: a national study	Wrong outcomes
Castellani, D.; Antonelli, E.; Sabatino, G.; Giuliano, V.; Morelli, A.; Bassotti, G.	2008	A simplified method for anal ultrasonography: assessment of patient satisfaction and the simplicity of the procedure	Wrong intervention
Castellani, D.; Antonelli, E.; Sabatino, G.; Morelli, O.; Baldoni, M.; Clerici, C.; Morelli, A.; Bassotti, G.	2009	A simplified method for anal ultrasonography: preliminary report	Too early
Castro Filho EC; Perazzo H; Guimaraes RAP; Machado L; Fernandes FF; Perez RM	2018	Reliability and safety of transnasal compared to conventional endoscopy for detecting oesophageal varices in cirrhotic patients.	Wrong intervention
Cattau, E. L.; Leal, R. J.; Ormseth, E. J.; Aycock, R.; Ward, J. D.; Thompson, B. F.; Dragutsky, M.; Towne, C.	2015	The effect of endocuff TM-assisted colonoscopy on adenoma detection rate: A randomized trial in community ambulatory surgical centers	Wrong publication type
Cazacu, I. M.; Vasile Balaban, D.; Pinte, L.; Jinga, M.; Bhutani, M. S.; Saftoiu, A.	2019	Endoscopic ultrasound-through-the-needle microforceps biopsy in pancreatic cystic lesions: A systematic review	Wrong intervention
Cennamo, V.; Botter, A.; Landi, S.; Graziosi, F.; Bassi, M.; Dabizzi, E.; Ghersi, S.; Cerone, G.; Bonfiglioli, R.	2023	CAN SINGLE-USE VERSUS STANDARD DUODENOSCOPE IMPROVE ERGONOMICS IN ERCP?A COMPARATIVE SIMULATION-BASED PILOT STUDY	Wrong study design
Ceresoli, M.; Tamini, N.; Gianotti, L.; Braga, M.; Nespoli, L.	2019	Are endoscopic loop ties safe even in complicated acute appendicitis? A systematic review and meta-analysis	Wrong comparator

Chahine, A.; Han, J. Y.; Shah, S.; Bernal, E.; Simoni, R.; Lee, J.; Samarasena, J. B.	2021	ID: 3522406 THE COST OF REUSABLE DUODENOSCOPES AT A HIGH VOLUME ACADEMIC CENTER - IS IT TIME TO SWITCH TO SINGLE-USE DUODENOSCOPES FOR ERCP?	Wrong publication type
Chang, P. W.; Bui, A.; Zhou, S.; Sahakian, A. B.; Buxbaum, J. L.; Phan, J.	2024	Increasing Antibiotic-Resistant Infections With Inpatient Endoscopic Retrograde Cholangioscopies (ERCP) Is Associated With Higher Mortality in the United States	Wrong outcomes
Chen, Y. K.; Parsi, M. A.; Binmoeller, K. F.; Hawes, R. H.; Pleskow, D.; Slivka, A.; Haluszka, O.; Petersen, B. T.; Sherman, S.; Deviere, J.; Meisner, S.; Stevens, P. D.; Costamagna, G.; Ponchon, T.; Neuhaus, H.	2008	Peroral cholangioscopy (PO) using a disposable steerable single operator catheter for biliary stone therapy and assessment of indeterminate strictures - A multi-center experience using spyglass	Wrong intervention
Chen, Y. K.; Parsi, M. A.; Binmoeller, K. F.; Hawes, R.; Pleskow, D. K.; Slivka, A.; Haluszka, O.; Petersen, B. T.; Sherman, S.; Deviere, J. M.; Meisner, S.; Stevens, P. D.; Costamagna, G.; Ponchon, T.; Neuhaus, H.	2009	Peroral cholangioscopy (POC) using a disposable steerable single operator catheter for biliary stone therapy and assessment of indeterminate strictures - A multi-center experience using SPYGLASS	Wrong intervention
ChiCtr	2024	Clinical trial of disposable electronic colonoscopy for examining comfort and endoscopic mucosal resection	Study ongoing
ChiCTR2400079874; Binzhou Medical University/ Binzhou Medical University Hospital, No	2024	Evaluation of the Efficacy of Self-made Magnetically Controlled Capsule Endoscopy Sugar Coating Traction Aids Combined with Traditional Chinese Sugar Painting Technology in Observing the Efficacy of the Cardia	Not empirical
ChiCTR2400081465	2024	Application of disposable visual ultra-fine diameter appendiceal endoscopy	Study ongoing
ChiCTR2400081801	2024	Clinical study on the efficacy and safety of single-use electronic lower gastrointestinal endoscope combined with medical endoscope image processor for lower gastrointestinal examination	Study ongoing
ChiCTR2400083860	2024	Management of difficult common bile duct stones using peroral cholangioscopy based on ALARA concept: a multi-center, prospective non-randomized concurrent controlled trial	Study ongoing
Cho, J. H.; Jin, S. Y.; Park, S.	2024	Carbon footprint and cost reduction by endoscopic grading of gastric intestinal metaplasia using narrow-band imaging	Wrong outcomes
Chuah, T. L.; Hong, M. C.; Foroughi, B.	2024	Consumers' preferences for endoscopes: a discrete choice experiment	Wrong outcomes
Chung, J. W.; Park, S.; Chung, M. J.; Park, J. Y.; Park, S. W.; Chung, J. B.; Song, S. Y.	2012	A novel disposable, transnasal esophagoscope: a pilot trial of feasibility, safety, and tolerance	Duplicate
Cohen, J.; Haber, G. B.; Kortan, P.; Dorais, J. A.; Scheider, D. M.; Cirocco, M.; Habib, J.	1997	A prospective study of the repeated use of sterilized papillotomes and retrieval baskets for ERCP: quality and cost analysis	Too early
Conti, C. B.; Cereatti, F.; Salerno, R.; Grassia, R.; Scaravaglio, M.; Laurenza, C.; Dinelli, M. E.	2023	Disposable Duodenoscopes: Evidence and Open Issues	Not empirical
Corrigall, D.; Shaw, K.; Beg, S.; Morris, D.	2023	DOES CYTOSPONGE TRIAGE REDUCE THE CARBON FOOTPRINT OF MANAGING PATIENTS WITH ACID REFLUX AND BARRETT'S OESOPHAGUS?	Wrong outcomes
Cosentino, F.; Tumino, E.; Passoni, G. R.; Morandi, E.; Capria, A.	2009	Functional evaluation of the endotics system, a new disposable self-propelled robotic colonoscope: in vitro tests and clinical trial	Too early
Costa, E. A. M.; Moreira, L. L.; Chagas, T.; Barreto, M.	2019	Adverse events in gastrointestinal endoscopy: a cohort of 62,088 procedures performed	Wrong outcomes
Craig, P.; Verghese, G.; Deltetto, I.; Ryder, I.; Hide, J.; Devereaux, B.; Taylor, C.	2023	Cost-effectiveness of single-operator, single-use peroral cholangioscopy for the removal of difficult biliary stones and the diagnosis of indeterminant biliary strictures in Australia	Wrong outcomes
Crockett, S. D.; Skole, K. S.; Hernandez, L. V.; von Renteln, D.; Agrawal, D.; Pohl, H.; Shimpi, R. A.	2023	Practical steps to green your endoscopy unit: how to get started	Wrong outcomes
Croffie, J.; Carpenter, S.; Chuttani, R.; DiSario, J.; Hussain, N.; Liu, J.; Mishkin, D.; Somogyi, L.; Tierney, W.; Petersen, B. T.	2005	ASGE Technology Status Evaluation Report: disposable endoscopic accessories	Wrong study design

CTRI/2023/12/060728; Boston Scientific India Private Ltd, Yes	2023	Direct visualization and treatment of pancreatico-biliary system with a single-use cholangiopancreatoscope during a pancreatico-biliary surgical procedure.	Wrong intervention
Cunha Neves, J. A.; Rodriguez de Santiago, E.; Pohl, H.; Lorenzo-Zuniga, V.; Cunha, M. F.; Voiosu, A. M.; Rommele, C.; Penman, D. G.; Albeniz, E.; Siau, K.; Donnelly, L.; Elli, L.; Pioche, M.; Beilenhoff, U.; Arvanitakis, M.; Weusten, Blam; Bisschops, R.; Hassan, C.; Messmann, H.; Gralnek, I. M.; Dinis-Ribeiro, M.	2024	Perspectives and awareness of endoscopy healthcare professionals on sustainable practices in gastrointestinal endoscopy: results of the LEAFGREEN survey	Wrong outcomes
Cunha Neves, J. A.; Roseira, J.; Queiros, P.; Sousa, H. T.; Pellino, G.; Cunha, M. F.	2023	Targeted intervention to achieve waste reduction in gastrointestinal endoscopy	Wrong outcomes
Dabaja-Younis, H. K.; Schechner, V.; Firan, I.; Khamaysi, I.; Levi, G. D.; Lurie-Weinberger, M. N.; Weissman, A.; Guriel, Y.; Geffen, Y.; Hussein, K.	2023	Identification and control of two outbreaks of unrelated New Delhi metallo-beta-lactamase-producing carbapenem-resistant Escherichia coli traced to the same endoscope defect	Wrong outcomes
Das, A.; Cangelosi, M. J.; Muthusamy, V. R.	2022	A Cost-Effectiveness Analysis of Exalt Model D Single-Use Duodenoscope Versus Current Duodenoscope Reprocessing Methods	Wrong outcomes
Das, A.; Cangelosi, M. J.; Muthusamy, V. R.	2020	SINGLE-USE DUODENOSCOPES FOR ERCP: A COST-EFFECTIVE STRATEGY TO PREVENT INFECTIONS ASSOCIATED WITH REPROCESSING	Wrong outcomes
Davavala, S.; Abraham, P.; Desai, D.; Joshi, A.; Gupta, T.; Samant, H.	2016	Single use versus reuse of endoscopy biopsy forceps: A survey of patient preference	Wrong intervention
De Jong, D.; Volkers, A.; De Ridder, E.; D'Haens, G. R.; Fockens, P.; Duijvestein, M.	2022	TOWARDS A GREENER ENDOSCOPY ROOM: RECYCLING PLASTIC WASTE	Wrong comparator
de Jong, D.; Volkers, A.; de Ridder, E.; Neijenhuis, M.; Duijvestein, M.	2023	Steps Towards a Greener Endoscopy Unit	Wrong outcomes
de Jong, D.; Volkers, A.; de Ridder, E.; Neijenhuis, M.; Duijvestein, M.	2023	Steps Toward a Greener Endoscopy Unit	Wrong outcomes
De Palma, G. D.; Giglio, M. C.; Bruzzese, D.; Gennarelli, N.; Maione, F.; Siciliano, S.; Manzo, B.; Cassese, G.; Luglio, G.	2018	Cap cuff-assisted colonoscopy versus standard colonoscopy for adenoma detection: a randomized back-to-back study	Wrong intervention
Dean, R.; Salvatore, B.; Varunok, P.; Ashraf, K.	2023	Single Use Duodenoscopes Are Financially Viable for ERCP: A Real-World Study Within a Community Setting	Wrong outcomes
DeMarco, D. C.; Odstrcil, E.; Lara, L. F.; Bass, D.; Herdman, C.; Kinney, T.; Gupta, K.; Wolf, L.; Dewar, T.; Deas, T. M.; Mehta, M. K.; Anwer, M. B.; Pellish, R.; Hamilton, J. K.; Polter, D.; Reddy, K. G.; Hanan, I.	2010	Impact of experience with a retrograde-viewing device on adenoma detection rates and withdrawal times during colonoscopy: the Third Eye Retroscope study group	Wrong comparator
Dennis, M. J.; James, M. J.; Wherry, D.; Doran, J.; Morris, D. L.	1989	Choledochoscopy via the cystic duct: evaluation of a new ultrathin disposable choledochoscope	Sample too small
Deprez, P. H.; Horsmans, Y.; Van Hassel, M.; Hoang, P.; Piessevaux, H.; Geubel, A.	2000	Disposable versus reusable biopsy forceps: a prospective cost evaluation	Wrong intervention
Desai, M.; Campbell, C.; Perisetti, A.; Srinivasan, S.; Radadiya, D.; Patel, H.; Melquist, S.; Rex, D. K.; Sharma, P.	2024	The Environmental Impact of Gastrointestinal Procedures: A Prospective Study of Waste Generation, Energy Consumption, and Auditing in an Endoscopy Unit	Wrong outcomes
Desilets, D.; Kaul, V.; Tierney, W. M.; Banerjee, S.; Diehl, D. L.; Farraye, F. A.; Kethu, S. R.; Kwon, R. S.; Mamula, P.; Pedrosa, M. C.; Rodriguez, S. A.; Wong Kee Song, L. M.	2010	Automated endoscope reprocessors	Wrong study design

Ding, H.; Li, Y.	2023	Effect of endoscopic loop ties in acute appendicitis on wound infection rate: A meta-analysis	Wrong intervention
Docimo, S., Jr.; Sucandy, I.; Luhrs, A.; Snow, T.; Pechman, D.	2023	TAVAC: choledochoscopy disposable scopes, and the single-stage vs. two-stage approach to choledocholithiasis	Wrong study design
Dolwani, S.; Saleem, H.; Thompson, I. W.; Allison, M. C.	2002	A comparison of three types of biopsy forceps in the endoscopic surveillance of Barrett's oesophagus	Wrong intervention
Duarte, P.; Benjamin, W.; Patel, P.; Boger, P.; Schmidbaur, S.; Albers, D.; Schumacher, B.; Meining, A.; Rahman, I.	2021	Bougiecap dilatation device: Novel endoscopic method for treatment of oesophageal strictures-results from a multicentre study	Wrong intervention
Durante, L.; Zulty, J. C.; Israel, E.; Powers, P. J.; Russell, R. G.; Qizilbash, A. H.; Morris, J. G.	1992	INVESTIGATION OF AN OUTBREAK OF BLOODY DIARRHEA - ASSOCIATION WITH ENDOSCOPIC CLEANING SOLUTION AND DEMONSTRATION OF LESIONS IN AN ANIMAL-MODEL	Too early
Ebigbo, A.; Tadic, V.; Schlottmann, J.; Braun, G.; Prinz, F.; Wanzl, J.; Ayoub, M.; Kraus, L.; Scheppach, M.; Nagl, S.; Schnoy, E.; Weber, T.; Probst, A.; Messmann, H.; Rommele, C.	2023	Evaluation of a single-use gastroscope in patients presenting with suspected upper gastrointestinal hemorrhage: a pilot feasibility study (One-Scope I)	Sample too small
Ehrlich, D.; Chittajallu, P.; Phan, J.; Paredes, H. E.; Issa, D.; Thaker, A. M.; Kim, S.; Sedarat, A.; Muthusamy, V. R.	2021	ID: 3526115 THE EXALT MODEL D SINGLE-USE DUODENOSCOPE IS SAFE AND EFFECTIVE IN POST-MARKET EXPERIENCE AT A TERTIARY MEDICAL CENTER	Sample too small
Ehrlich, D.; Muthusamy, V. R.	2021	Device profile of the EXALT Model D single-use duodenoscope for endoscopic retrograde cholangiopancreatography: overview of its safety and efficacy	Wrong study design
El, Hajj, II; Shah, R. J.	2016	Digital single-operator cholangioscopy: fully disposable yet valuable	Wrong study design
El-Demerdash, T.; Yousef, M.; Abd-Elsalam, S.; Helmy, A.; Kobtan, A.; Elfert, A. A.	2019	Reuse of Biopsy Forceps may be Associated with Risk of Transmission of HCV in Egyptian Patients Undergoing Gastrointestinal Endoscopy	Wrong intervention
Ell, C.; May, A.; Wurster, H.	1999	The first reusable multiple-band ligator for endoscopic hemostasis of variceal bleeding, nonvariceal bleeding and mucosal resection	Too early
Elli, L.; La Mura, S.; Rimondi, A.; Scaramella, L.; Tontini, G. E.; Monica, F.; Soncini, M.; Topa, M.; Bortoluzzi, F.; Sorge, A.; Cavallaro, F.; Nandi, N.; Noviello, D.; Piagnani, A.; Maregatti, M.; Caldato, M.; Vecchi, M.	2024	The carbon cost of inappropriate endoscopy	Wrong outcomes
Elnour, A.; Subramanian, K.; Viswanath, Y. K. S.	2023	A Systematic review and Narrative Synthesis of Health Economic Evaluation of single use duodenoscopes	Wrong outcomes
Epstein, L.; Hunter, J. C.; Arwady, M. A.; Tsai, V.; Stein, L.; Gribogiannis, M.; Frias, M.; Guh, A. Y.; Laufer, A. S.; Black, S.; Pacilli, M.; Moulton-Meissner, H.; Rasheed, J. K.; Avillan, J. J.; Kitchel, B.; Limbago, B. M.; MacCannell, D.; Lonsway, D.; Noble-Wang, J.; Conway, J.; Conover, C.; Vernon, M.; Kallen, A. J.	2014	New Delhi metallo-beta-lactamase-producing carbapenem-resistant Escherichia coli associated with exposure to duodenoscopes	Wrong intervention
Fichtl, A.; Tacheva, V.; Sturm, N.; Hamesch, K.; Wichmann, D.; Mayer, B.; Muller, M.; Wagner, M.; Seufferlein, T.; Walter, B. M.	2024	Impact of power consumption and power saving for GI endoscopy (power on study) on reducing CO₂ emissions	Wrong outcomes
Figueiredo, M.; Yzet, C.; Grinberg, D.; Lambin, T.; Cottinet, P. J.; Rivory, J.; Pioche, M.	2022	How to reduce the ecological impact of gastric peroral endoscopic myotomy: a small effort for the environment	Wrong comparator

Fireman, Z.; Jacob, H.; Eliakim, A.; Goldfarb-Albak, S.	2005	Innovative technology facilitates colonoscope disinfection: ColonoSight (R) Intrapull (TM) power assisted disposable technology isolates infections agents from physician, nurse and patient, no high level disinfection (HLD) necessary between procedures	Wrong outcomes
Forte, L.; Shum, C.	2011	Comparative cost-efficiency of the EVOTECH endoscope cleaner and reprocessor versus manual cleaning plus automated endoscope reprocessing in a real-world Canadian hospital endoscopy setting	Wrong outcomes
Fritscher-Ravens, A.; Fox, S.; Swain, C. P.; Milla, P.; Long, G.	2006	CathCam guide wire-directed colonoscopy: first pilot study in patients with a previous incomplete colonoscopy	Sample too small
Fugazza, A.; Lamonaca, L.; Craviotto, V.; Spadaccini, M.; Capogreco, A.; Paduano, D.; Pugliese, N.; Vespa, E.; Colombo, M.; Buono, A. D.; Gabbiadini, R.; Badalamenti, M.; Alvisi, C.; Aragona, G.; Bassi, M.; Benedetti, A.; Canani, M. B.; Beretta, P.; Calcara, C.; Antonio, C.; Canfora, M. L.; Cantu, P.; Camellini, L.; Cengia, G.; Cereatti, F.; Costamagna, G.; Cugia, L.; De Angelis, C.; De Bellis, M.; De Grazia, F.; Del Vecchio Blanco, G.; Di Giulio, E.; Diamantis, G.; Dinelli, M.; Fabbri, C.; Feliciangeli, G.; Gabbrielli, A.; Gambitta, P.; Gllotta, R.; Lauri, A.; Leoni, P.; Luigiano, S. C.; Macarri, G.; Manes, G.; Manfredi, G.; Manno, M.; Mantovani, N.; Mariani, A.; Masci, E.; Missale, G.; Mosca, P.; Mussetto, A.; Mutignani, M.; Occhipinti, P.; Parodi, M. C.; Radaelli, F.; Salerno, R.; Traina, M.; Tringali, A.; Venezia, G.; Paganelli, M.; Ayoubi, M.; Carrara, S.; Maselli, R.; Anderloni, A. A.; Repici, A.	2020	DUODENOSCOPE-RELATED INFECTIONS: AN ITALIAN PICK IN 2019	Wrong outcomes
Fujiwara, K.; Kondo, T.; Fujimoto, K.; Fujie, M.; Kanogawa, N.; Ogasawara, S.; Kato, N.	2023	Use of disposable endoscope for variceal sclerotherapy	Sample too small
Funk, S. E.; Reaven, N. L.	2014	High-level endoscope disinfection processes in emerging economies: financial impact of manual process versus automated endoscope reprocessing	Wrong outcomes
Gael, R.; Philippe, B.; Michele, F. G.; Jean, A.; Letoublon, C.; Bricault, I.; Leroy, V.; Decaens, T.	2014	Prospective evaluation of the performance of per ERCP bile aspiration combined to endobiliary brushing in the diagnosis of malignant strictures of the biliary tract	Wrong intervention
Gamble, H. P.; Duckworth, G. J.; Ridgway, G. L.	2007	Endoscope decontamination incidents in England 2003-2004	Wrong outcomes
Garman, K. S.; Peery, A. F.; Daugherty, N.; Hoppo, T.; Bream, S.; Sanz, A. F.; Spacek, M.; Connors, D.; Faulx, A. L.; Chak, A.; Luketich, J. D.; Jobe, B. A.; J. Shaheen N	2011	Yield of transnasal endoscopy in an unselected general medical population	Wrong intervention
Gastmeier, P.; Vonberg, R. P.	2014	Klebsiella spp. in endoscopy-associated infections: we may only be seeing the tip of the iceberg	Wrong outcomes
Gatenby, P. A. C.	2011	Modelling the carbon footprint of reflux control	Wrong outcomes
Gluck, N.; Fishman, S.; Melhem, A.; Goldfarb, S.; Halpern, Z.; Santo, E.	2014	Aer-o-scopeTM, a self-propelled pneumatic colonoscope, is superior to conventional colonoscopy in polyp detection	Wrong patient population
Gluck, N.; Melhem, A.; Halpern, Z.; Mergener, K.; Goldfarb, S.; Santo, E.	2015	The Aer-O-Scope colonoscope system is safe and effective for colonoscopy in humans	Wrong study design

Gluck, N.; Melhem, A.; Halpern, Z.; Mergener, K.; Santo, E.	2016	A novel self-propelled disposable colonoscope is effective for colonoscopy in humans (with video)	Wrong intervention
Goetz, M.; Malek, N. P.	2018	Novel triple lumen catheter for ERCP tissue diagnosis	Sample too small
Gonzalez-Candelas, F.; Guiral, S.; Carbo, R.; Valero, A.; Vanaclocha, H.; Gonzalez, F.; Bracho, M. A.	2010	Patient-to-patient transmission of hepatitis C virus (HCV) during colonoscopy diagnosis	Wrong outcomes
Guidi, MartÃn; Curvale, Cecilia; Hwang, Hui Jer; MÃ¡laga, Ignacio; De MarÃa, Julio; Matwis, Sonia; Schenone, VerÃ³nica; Barcos, Ana; MatanÃ³, RaÃºl	2017	ReutilizaciÃ³n de materiales de colangiografÃa endoscÃ³pica. Estudio prospectivo de minimizaciÃ³n de costos	Foreign language
Han, J. H.; Cho, Y. S.; Park, S. M.; Chae, H. B.; Yoon, S. M.; Seo, E. K.; Youn, S. J.	2011	How much does cap-fitted gastroscopy improve the examination of the duodenal ampulla?	Wrong intervention
Han, Z. L.; Lin, B. T.; Wang, Z. J.; Chen, X.; Xi, Y. Y.; Wang, J. F.; Qiao, W. G.; Huang, Y.; Lin, Z. Z.; Huang, S. H.; Chua, T. Y.; Liu, S. D.; Luo, X. B.	2023	Evaluation of a novel disposable esophagogastroduodenoscopy system in emergency, bedside, and intraoperative settings: Pilot study (with videos)	Sample too small
Harper, J.; DeVries, A.; Danila, R.; Lesher, L.	2012	Reported endoscope reprocessing breaches, Minnesota, 2010-2011	Wrong publication type
Hayee, B.; Chung-Faye, G.	2016	Endoringstm increases ADR even in high-risk screening colonoscopy: Results of a single centre pilot study	Wrong publication type
Hearing, S.; Fletcher, J.	2019	Roboscope-2 centre initial experience	Wrong intervention
Henniger, D.; Lux, T.; Windsheimer, M.; Brand, M.; Weich, A.; Kudlich, T.; Schottker, K.; Hann, A.; Meining, A.	2024	Reducing scope 3 carbon emissions in gastrointestinal endoscopy: results of the prospective study of the 'Green Endoscopy Project Wurzburg'	Wrong outcomes
Henniger, D.; Windsheimer, M.; Beck, H.; Brand, M.; Lux, T.; Hann, A.; Meining, A.	2023	Assessment of the yearly carbon emission of a gastrointestinal endoscopy unit	Wrong outcomes
Henriot, P.; Castry, M.; Luong Nguyen, L. B.; Shimakawa, Y.; Jean, K.; Temime, L.	2022	Meta-analysis: risk of hepatitis C virus infection associated with hospital-based invasive procedures	Wrong outcomes
Hernandez, L. V.; Agrawal, D.; Skole, K. S.; Crockett, S. D.; Shimpi, R. A.; von Renteln, D.; Pohl, H.	2023	Meeting the environmental challenges of endoscopy: a pathway from strategy to implementation	Wrong outcomes
Hernandez, L. V.; Ravi, S. K.; Guda, N. M.; Gresenz, A.; Luo, J.	2022	MICRO-COST ESTIMATES OF PROCEDURES IN A COMMUNITY-BASED AMBULATORY ENDOSCOPY CENTER: WHEN DO WE SWITCH TO SINGLE-USE ENDOSCOPES?	Wrong outcomes
Herrin, A.; Loyola, M.; Bocian, S.; Diskey, A.; Friis, C. M.; Herron-Rice, L.; Juan, M. R.; Schmelzer, M.; Selking, S.	2016	Standards of Infection Prevention in Reprocessing Flexible Gastrointestinal Endoscopes	Not empirical
Hicheri, C.; Azimuddin, A. M.; Kortum, A.; Bailey, J.; Tang, Y.; Schwarz, R. A.; Rosen, D.; Jain, S.; Mansour, N. M.; Groth, S.; Vasavada, S.; Rao, A.; Maliga, A.; Gallego, L.; Carns, J.; Anandasabapathy, S.; Richards-Kortum, R.	2024	Design and Evaluation of ScanCap: A Low-Cost, Reusable Tethered Capsule Endoscope with Blue-Green Illumination Imaging for Unsedated Screening and Early Detection of Barrett's Esophagus	Wrong intervention
Hiramatsu, K.; Naito, T.; Nosaka, T.; Takahashi, K.; Ofuji, K.; Matsuda, H.; Ohtani, M.; Nakamoto, Y.	2020	EFFICACY AND SAFETY OF HOOKKNIFE ELECTROSURGICAL KNIFE IN ENDOSCOPIC SUBMUCOSAL DISSECTION FOR THE TREATMENT OF SUPERFICIAL NON-AMPULLARY DUODENAL EPITHELIAL TUMOR	Wrong intervention

Hoffman, D.; Cool, C.	2023	SINGLE-USE GASTROSCOPE USAGE AND IMPLICATIONS IN A HIGH PROCEDURE VOLUME FACILITY: A CASE-STUDY	Wrong outcomes
Hogan, R. B.; Santa-Cruz, R.; Weeks, E. S., Jr.; Alexander, L.; Hogan, R. B., 3rd	2009	Cost-minimization analysis of jumbo reusable forceps versus disposable forceps in a high-volume ambulatory endoscopy center	Wrong intervention
Holodniy, M.; Oda, G.; Schirmer, P. L.; Lucero, C. A.; Khudyakov, Y. E.; Xia, G.; Lin, Y.; Valdiserri, R.; Duncan, W. E.; Davey, V. J.; Cross, G. M.	2012	Results from a large-scale epidemiologic look-back investigation of improperly reprocessed endoscopy equipment	Wrong outcomes
Hoshino, H.; Goto, H.	2012	Infection control for gastroenterological endoscopy at practitioner clinics in Japan: A questionnaire survey	Foreign language
Hsieh, Y. L.; Chen, C. H.; Wu, M. C.; Shen, C. S.; Hsu, W. H.; Wu, D. C.; Wu, J. Y.	2021	A NEW TOOL OF MAGNETIC-ASSISTED CAPSULE ENDOSCOPE FOR UPPER GASTROINTESTINAL EXAMINATION	Wrong intervention
Humphries, R. M.; Yang, S.; Kim, S.; Muthusamy, V. R.; Russell, D.; Trout, A. M.; Zaroda, T.; Cheng, Q. J.; Aldrovandi, G.; Uslan, D. Z.; Hemarajata, P.; Rubin, Z. A.	2017	Duodenoscope-Related Outbreak of a Carbapenem-Resistant Klebsiella pneumoniae Identified Using Advanced Molecular Diagnostics	Wrong intervention
Ichkhanian, Y.; Albunni, H.; Gutta, A.; Watkins, J.; Fogel, E.; Easler, J.; Saleem, N.; Gromski, M.	2024	THE EVOLUTION OF SINGLE-USE DUODENOSCOPES AT A SINGLE LARGE_VOLUME TERTIARY CARE CENTER	No endoscope data for case series
Inoue, H.; Takemura, K.; Hori, H.; Itoh, K.; Muraoka, Y.; Yoneshima, H.; Endo, M.	1992	Sclerotherapy of internal hemorrhoids using newly devised transparent disposable anorectoscope	Wrong study design
Islam, S.; Naidu, S.	2015	Outcome of post-colonoscopy rubber band ligation of symptomatic internal haemorrhoids	Wrong intervention
Jensen, D. M.; Kovacs, T. O.; Ohning, G. V.; Ghassemi, K.; Machicado, G.; Dulai, G.; Sedarat, A.; Jutabha, R.; Gornbein, J.	2016	Randomized controlled trial (RCT) of doppler endoscopic probe (DEP) assisted treatment compared to standard hemostasis of severe variceal or portal hypertensive lesion UGI bleeding	Wrong intervention
Jensen, D. M.; Ohning, G. V.	2016	Preventing delayed post-polypectomy induced ulcer hemorrhage by using doppler endoscopic probe as a guide to risk stratification & prophylactic hemostasis after polypectomy	Wrong intervention
Jensen, D. M.; Ohning, G. V.	2014	Doppler endoscopic probe (DEP) as a guide to risk stratification & prophylaxis to prevent delayed post-polypectomy induced ulcer (PPIU) hemorrhage after polypectomy	Wrong comparator
Jiao, H.; Li, J.; Li, L.; Li, Y.; Sun, D.; Liu, J.	2022	SMALL BOWEL VISUALIZATION QUALITY OF MAGNETICALLY CONTROLLED CAPSULE ENDOSCOPY IN OLDEST-OLD PATIENTS: A DESCRIPTIVE STUDY	Sample too small
Jin, H. Y.; Leng, Q.	2015	Use of disposable graduated biopsy forceps improves accuracy of polyp size measurements during endoscopy	Wrong intervention
Jones, B.; Gupta, S.	2010	Risk of cross-contamination in rigid sigmoidoscopy: the use of disposable filters	Wrong intervention
JPRN-jRCT1032230493	2023	Study of usability of Gastrointestinal Endoscopy with a Single-Use Endoscope	Study ongoing
Jurado, J. R.; Nieto, J. H. S.; Gaitan, J. O.; Bonilla, C. G.; Villarreal, R.; Acevedo, D.; Cabrera, L.; Cabrera, L. F.; Pedraza, M.	2021	Endoscopy mitigation strategy with telemedicine and low-cost device use for COVID-19 prevention: A fourth-level Colombian center experience	Wrong intervention
Kagawa, Y.; Fukuzawa, M.; Itoi, T.	2020	Novel dedicated plastic cube for infection prevention during gastrointestinal endoscopy and endoscopic retrograde cholangiopancreatography	Wrong study design
Kampf, G.; Jung, M.; Suchomel, M.; Saliou, P.; Griffiths, H.; Vos, M. C.	2020	Prion disease and recommended procedures for flexible endoscope reprocessing - a review of policies worldwide and proposal for a simplified approach	Wrong study design
Kelsey, L.; Herron-Rice, L.; Anderson, P.; Day, M.; Friis, C. M.; Gondzur, N.; Girard, D.; Malone, M. A.; Penberthy, J.; Stewart, L.; Albertson, L. E.; Schaeublin, T.; Symms, M. R.; Strohmeyer, L.; Sgna Practice Comm	2010	STANDARDS OF INFECTION CONTROL IN REPROCESSING OF FLEXIBLE GASTROINTESTINAL ENDOSCOPES	Wrong outcomes

Kim-Deobald, J.; Kozarek, R. A.; Ball, T. J.; Patterson, D. J.; Brandabur, J. J.; Raltz, S.	1993	Prospective evaluation of costs of disposable accessories in diagnostic and therapeutic ERCP	Too early
Kohli, D. R.; Aqel, B. A.; Segaran, N. L.; Harrison, M. E.; Fukami, N.; Faigel, D. O.; Moss, A.; Mathur, A.; Hewitt, W.; Katariya, N.; Pannala, R.	2023	Outcomes of endoscopic retrograde cholangiography and percutaneous transhepatic biliary drainage in liver transplant recipients with a Roux-en-Y biliary-enteric anastomosis	Wrong intervention
Kola, A.; Piening, B.; Pape, U. F.; Veltzke-Schlieker, W.; Kaase, M.; Geffers, C.; Wiedenmann, B.; Gastmeier, P.	2015	An outbreak of carbapenem-resistant OXA-48-producing Klebsiella pneumonia associated to duodenoscopy	Sample too small
Korolev, M.; Fedotov, L.; Ogloblin, A.; Mamedov, S.; Antipova, M.; Klimov, A.; Avanesyan, R.; Smirnov, A.	2018	Intraoperative complications of peroral endoscopic myotomy (POEM)	Wrong intervention
Kotovskiy, A. E.; Magomedova, B. M.; Glebov, K. G.; Martyntsov, A. A.; Mahmudova, A. K.; Prividentseva, A. S.	2021	Differentiated approach of using extraction balloon catheters in endoscopic biliary surgery	Wrong intervention
Kovaleva, J.; Meessen, N. E. L.; Peters, F. T. M.; Been, M. H.; Arends, J. P.; Borgers, R. P.; Degener, J. E.	2009	Is bacteriologic surveillance in endoscope reprocessing stringent enough?	Wrong outcomes
Kozarek, R. A.; Attia, F. M.; Sumida, S. E.; Raltz, S. L.; Roach, S. K.; Schembre, D. B.; Brandabur, J. J.; Ball, T. J.; Gluck, M.; Jiranek, G. C.; Patterson, D. J.; Bredfeldt, J. E.; Gelfand, M.; McCormick, S. E.; Drajpuch, D. B.; Moran, D. K.	2001	Reusable biopsy forceps: a prospective evaluation of cleaning, function, adequacy of tissue specimen, and durability	Wrong intervention
Kozarek, R. A.; Raltz, S. L.; Merriam, L. D.; Sumida, S. E.	1996	Disposable versus reusable biopsy forceps: A prospective evaluation of costs	Too early
Krishna, C. G.; Kanta, S.; Rao, B. H.; Nair, S. V.; Methala, S. P.; Nair, P.; Koshy, A. K.; Rajesh, G.; Sadasivan, S.; Venu, R. P.	2022	Role of disposable elevator tip in the prevention of persistent bacterial contamination of reprocessed duodenoscopes	Wrong publication type
Kwakman, J. A.; Poley, M. J.; Vos, M. C.; Bruno, M. J.	2023	Single-use duodenoscopes compared with reusable duodenoscopes in patients carrying multidrug-resistant microorganisms: a break-even cost analysis	Wrong outcomes
Kwakman, J.; Vos, M. C.; Bruno, M. J.	2020	RISK ESTIMATE OF DUODENOSCOPE-ASSOCIATED INFECTIONS (DAI) IN THE NETHERLANDS	Duplicate
Lacroute, J.; Marcantoni, J.; Petitot, S.; Weber, J.; Levy, P.; Dirrenberger, B.; Tchoumak, I.; Baron, M.; Gibert, S.; Marguerite, S.; Huppertz, J.; Gronier, O.; Derlon, A.	2023	The carbon footprint of ambulatory gastrointestinal endoscopy	Wrong outcomes
Lacroute, J.; Marcantoni, J.; Petitot, S.; Weber, J.; Levy, P.; Dirrenberger, B.; Tchoumak, I.; Baron, M.; Gibert, S.; Marguerite, S.; Huppertz, J.; Gronier, O.; Derlon, A.	2022	The carbon footprint of ambulatory gastrointestinal endoscopy	Wrong outcomes
Lagstrom, R.; Stigaard, T.; Knuhtsen, S.; Bulut, M.	2022	Diagnostic esophagogastroduodenoscopy performed using a novel sterile single-use disposable endoscope	Wrong study design
Landaeta, Jorge; Dias, Carla; Paternina, Ricardo; Tempestini, Ornella; Caraballo, Diego	2016	Experiencia colangioscopia peroral monoperador (SPYGLASS) en patologÃa biliar en un centro privado de tercer nivel en Venezuela	Foreign language
Larsen, N.; Oldin, C.; Hollaender, F.; Adamsen, S.; Jorgensen, E. P.	2022	POSB326 Organizational Assessment of US GI Endoscopy Units	Wrong study design
Larsen, S.; Kalloo, A.; Hutfless, S.	2020	The hidden cost of colonoscopy including cost of reprocessing and infection rate: the implications for disposable colonoscopes	Wrong outcomes
Larsen, S.; Russell, R. V.; Ockert, L. K.; Spanos, S.; Travis, H. S.; Ehlers, L. H.; Maerkedahl, A.	2020	Rate and impact of duodenoscope contamination: A systematic review and meta-analysis	Wrong intervention

Larsen, S.; Stromstad Travis, H.; Norgaard Thomsen, E. M.; Klinten Ockert, L.	2020	Survey-based assessment of market readiness for single-use duodenoscopes in Europe-will single-use duodenoscopes be the future?	Wrong publication type
Le, N. N. T.; Hernandez, L. V.; Vakil, N.; Guda, N.; Patnode, C.; Jolliet, O.	2022	Environmental and health outcomes of single-use versus reusable duodenoscopes	Wrong study design
Leblanc, S.; Duchmann, J. C.; Vienne, A.; Ponchon, T.; Coumaros, D.; Laugier, R.; Bichard, P.; Charachon, A.; Vedrenne, B.; Prat, F.	2011	Yield of spyglass cholangioscopy-guided biopsies in indeterminate biliary strictures: preliminary results of a multicenter french prospective study	Wrong publication type
Leblanc, S.; Vienne, A.; Duchmann, J. C.; Ponchon, T.; Bichard, P.; Laugier, R.; Coumaros, D.; Charachon, A.; Vedrenne, B.; Chaussade, S.; Prat, F.	2012	Diagnostic yield of spyglass cholangioscopy-guided biopsies in indeterminate biliary strictures and primary sclerosing cholangitis	Wrong intervention
Lee, W. M.; Moon, J. H.; Lee, Y. N.; Shin, I. S.; Myeong, J. H.; Kim, H. K.; Chung, J. C.; Lee, T. H.; Cha, S. W.; Cho, Y. D.; Park, S. H.	2022	The Role of Single-Operator Cholangioscopy for the Diagnosis and Treatment of Intraductal Papillary Neoplasm of the Bile Duct	Wrong intervention
Lejeune, C.; Prost, P.; Michiels, C.; Roullaud-Guenfoudi, M. P.; Phelip, J. M.; Martin, L.; Rassiat, E.; Faivre, J.	2001	[Disposable versus reusable biopsy forceps. A prospective cost analysis in the gastrointestinal endoscopy unit of the Dijon University Hospital]	Wrong intervention
Leng, Q.; Jin, H. Y.	2015	Measurement system that improves the accuracy of polyp size determined at colonoscopy	Wrong intervention
Li, D. F.; Shi, R. Y.; Tian, Y. H.; Xu, Z. L.; Zhou, Y. S.; Sun, X. J.; Cai, J. W.; Fang, Y. Y.; Peng, H.; Wang, J. M.; et al.,	2021	Die DurchfÃ¼hrbarkeit und Sicherheit von Einweg-Endoskopen bei der Untersuchung des oberen Gastrointestinaltrakts im Vergleich zu konventionellen Endoskopen: eine multizentrische, randomisierte, parallele, Nichtunterlegenheits-Studie	Foreign language
Lim, C. H.; Choi, M. G.; Baeg, M. K.; Moon, S. J.; Kim, J. S.; Cho, Y. K.; Park, J. M.; Lee, I. S.; Kim, S. W.; Choi, K. Y.	2014	Novel disposable transnasal endoscopy for assessment of esophageal motor function	Wrong comparator
Lim, C. H.; Choi, M. G.; Kim, W. C.; Kim, J. S.; Cho, Y. K.; Park, J. M.; Lee, I. S.; Kim, S. W.; Choi, K. Y.; Chung, I. S.	2012	Performance and cost of disposable biopsy forceps in upper gastrointestinal endoscopy: comparison with reusable biopsy forceps	Wrong intervention
Lisotti, A.; Fusaroli, P.; Napoleon, B.; Cominardi, A.; Zagari, R. M.	2022	Single-use duodenoscopes for the prevention of endoscopic retrograde cholangiopancreatography -related cross-infection - from bench studies to clinical evidence	Wrong study design
Lisotti, A.; Zagari, R. M.; Fusaroli, P.; Napoleon, B.	2022	Optimal safety and pooled technical success rate for ERCP performed with single-use duodenoscopes	Systematic review for checking only
Liu, P.; Zhang, X.; Lin, H. P.; Jin, H. J.; Leng, Q.; Zhang, J. H.; Zhang, Y.; Yao, H.; Wu, K. L.	2013	[Study on accuracy of endoscopic polyp size measurement by disposable graduated biopsy forceps]	Foreign language
Liu, T.; He, S.; Li, Q.; Wang, H.	2023	Cap-assisted endoscopic sclerotherapy is effective for rectal mucosal prolapse associated outlet obstructive constipation	Wrong intervention
Lopez-Munoz, P.; Martin-Cabezuelo, R.; Lorenzo-Zuniga, V.; Vilarino-Feltrer, G.; Tort-Ausina, I.; Vidaurre, A.; Pons Beltran, V.	2023	Life cycle assessment of routinely used endoscopic instruments and simple intervention to reduce our environmental impact	Wrong outcomes
Loyola, M.; Bocian, S.; Babb, E.; Diskey, A.; Friis, C. M.; Granato, A.; Schmit, A.; Selking, S.; Benitez-Romero, M.; Burkle, B. K.; Foliacci, T.; Fonkalsrud, L.; Luteran, D.; Myers, L. A.; Quillin, C. M.; Wahinehookae, C.; Cain, M.	2020	Management of Endoscopic Accessories, Valves, and Water and Irrigation Bottles in the Gastroenterology Setting	Not empirical
Lozano, M. D.; Subtil, J. C.; Miravalles, T. L.; Echeveste, J. I.; Prieto, C.; Betes, M.; Cienfuegos, F. J. A.; Idoate, M. A.	2011	EchoBrush may be superior to standard eus-guided fna in the evaluation of cystic lesions of the pancreas	Wrong intervention

Lubbe, J.; Arnelo, U.; Lundell, L.; Swahn, F.; Tornqvist, B.; Jonas, E.; Lohr, J. M.; Enochsson, L.	2015	ERCP-guided cholangioscopy using a single-use system: nationwide register-based study of its use in clinical practice	Wrong intervention
Ma, G. K.; Pegues, D. A.; Kochman, M. L.; Alby, K.; Fishman, N. O.; Saunders, M.; Grous, C.; Dempsey, D. T.; Ginsberg, G. G.	2018	Implementation of a systematic culturing program to monitor the efficacy of endoscope reprocessing: outcomes and costs	Wrong outcomes
Ma, W. S.; Ooi, M.; Safaeian, R.; Huynh, D.; Tse, E.; Nguyen, N. Q.	2020	IMPACT OF THE SAME DAY, UN-SEDATED, ULTRATHIN DISPOSABLE GASTROSCOPY IN THE MANAGEMENT OF CIRRHOTIC PATIENTS WHO ATTENDED OUTPATIENT CLINIC: A PROSPECTIVE LONGITUDINAL STUDY	Wrong study design
Makino, K.; Arora, N.; Tilden, D.; Bingham, W.	2013	Cost-effectiveness of capsule endoscopy (Pillcam) in the diagnosis of small bowel Crohn's disease	Wrong publication type
Manolakopoulos, S.; Christodoulou, C.; Giannousis, I.; Kotsikoros, N.; Theocharis, S. E.; Stefanidis, G.	2011	Application, safety and clinical outcome of spyglass direct visualization system in every day clinical practice. A single center experience	Wrong publication type
Martinek, J.; Maluskova, J.; Stefanova, M.; Tuckova, I.; Suchanek, S.; Vackova, Z.; Krajciova, J.; Kollar, M.; Zavoral, M.; Spicak, J.	2015	Improved specimen adequacy using jumbo biopsy forceps in patients with Barrett's esophagus	Wrong comparator
Masciangelo G; Cecinato P; Bacchilega I; Masetti M; Ferrari R; Zagari RM; Napoleon B; Sassatelli R; Fusaroli P; Lisotti A	2024	Urgent ERCP performed with single-use duodenoscope (SUD) in patients with moderate-to-severe cholangitis: Single-center prospective study.	Sample too small
Masciangelo, G.; Cecinato, P.; Bacchilega, I.; Masetti, M.; Ferrari, R.; Zagari, R. M.; Napoleon, B.; Sassatelli, R.; Fusaroli, P.; Lisotti, A.	2023	Urgent ERCP performed with single-use duodenoscope (SUD) in patients with moderate-to-severe cholangitis: Single-center prospective study	Sample too small
Matasov, S.; Borisova, A.	2006	"Colonoscope with disposable cartridges": Self-propelling - Does not need desinfection!	Not empirical
Mateen, H.; Basar, M. R.; Ahmed, A. U.; Ahmad, M. Y.	2017	Localization of Wireless Capsule Endoscope: A Systematic Review	Wrong outcomes
Matsuura, N.; Uedo, N.; Shichijo, S.; Kanesaka, T.; Maekawa, A.; Takeuchi, Y.; Higashino, K.; Ishihara, R.	2018	Diagnostic accuracy and safety of deep biopsy using forceps in patients with submucosal tumors of the upper and lower gastrointestinal tract: A retrospective single-center trial	Wrong intervention
Mayinger, B.; Hochberger, J.; Strenkert, M.; Martus, P.; Hahn, E. G.	1998	Endosheath: A disposable, sheathed, flexible gastroscopy: A prospective randomized trial	Too early
Mayinger, B.; Strenkert, B.; Martus, P.; Kunz, B.; Hahn, E. G.; Hochberger, J.	1998	[Disposable protection for flexible gastroenterologic endoscopy: prospective comparative evaluation of a new gastroscopy system (Endosheath) compared to the standard fiberglass gastroscope]	Foreign language
Mayinger, B.; Strenkert, M.; Hochberger, J.; Martus, P.; Kunz, B.; Hahn, E. G.	1999	Disposable-sheath, flexible gastroscope system versus standard gastroscopes: a prospective, randomized trial	Too early
McCafferty, C. E.; Aghajani, M. J.; Abi-Hanna, D.; Gosbell, I. B.; Jensen, S. O.	2018	An update on gastrointestinal endoscopy-associated infections and their contributing factors	Wrong outcomes
McCarty, T. R.; Obaid, L. A.; Bazarbashi, A. N.; Hathorn, K.; Thompson, C. C.	2019	EFFICACY AND SAFETY OF NOVEL, DISPOSABLE ENDOSCOPIC SCISSORS: A SINGLE-CENTER FEASIBILITY STUDY	Wrong intervention
McGoran, J.; Bennett, A.; Cooper, J.; De Caestecker, J.; Lovat, L. B.; Guha, N.; Ragunath, K.; Sami, S. S.	2019	Acceptability to patients of screening disposable transnasal endoscopy: qualitative interview analysis	Wrong study design
McLemore, E. C.; Weston, L. A.; Coker, A. M.; Jacobsen, G. R.; Talamini, M. A.; Horgan, S.; Ramamoorthy, S. L.	2014	Transanal minimally invasive surgery for benign and malignant rectal neoplasia	Wrong intervention
Mean, M.; Mallaret, M. R.; Bichard, P.; Shum, J.; Zarski, J. P.	2006	Gastrointestinal endoscopes cleaned without detergent substance following an automated endoscope washer/disinfector dysfunction	Wrong outcomes
Mege, D.; Bridoux, V.; Maggiori, L.; Tuech, J. J.; Panis, Y.	2017	What is the best tool for transanal endoscopic microsurgery (TEM)? A case-matched study in 74 patients comparing a standard platform and a disposable material	Wrong intervention

Mikhail, N. N.; Lewis, D. L.; Omar, N.; Taha, H.; El-Badawy, A.; Abdel-Mawgoud, N.; Abdel-Hamid, M.; Strickland, G. T.	2007	Prospective study of cross-infection from upper-GI endoscopy in a hepatitis C-prevalent population	Wrong intervention
Mittal, N.; Clark, I. W.; Hillman, L.; Gough, S.; Shadbolt, B.	2013	Reusable biopsy forceps versus disposable biopsy forceps for gastrointestinal endoscopic biopsies	Wrong intervention
Miyauchi, M.; Hayashida, M.; Yokota, H.	2014	Utility of ultrathin transnasal esophagogastroduodenoscopy for poisoned patients	Wrong intervention
Muralidharan, S.; Breese, M.; Chalikonda, D.; Kim, G.; Andrade, M.; Chen, D.; Siddiqui, U.	2024	COMPARISON OF NOVEL AND REUSABLE DUODENOSCOPES IN A SINGLE CENTER SHOW SIMILAR CONTAMINATION RATES THAT DO NOT CORRELATE WITH CLINICAL INFECTION	Wrong outcomes
Muscarella, L. F.	2014	Risk of transmission of carbapenem-resistant Enterobacteriaceae and related "superbugs" during gastrointestinal endoscopy	Wrong study design
Muscarella, L. F.	2019	Use of ethylene-oxide gas sterilisation to terminate multidrug-resistant bacterial outbreaks linked to duodenoscopes	Wrong outcomes
Muthusamy, V. R.; Ross, A. S.	2018	Economic burden of emergent practices of duodenoscopes reprocessing and surveillance: Balancing risk-and costcontainment	Wrong comparator
Mutignani, M.; Bonato, G.; Dioscoridi, L.; Cottone, I.; Cintolo, M.; Palermo, A.; Bravo, M.; Pugliese, F.; Capasso, M.; Palmeri, E.; Donnarumma, D.; Forti, E.	2024	PREVALENCE OF CARBAPENEMASE-PRODUCING ORGANISMS (CPO) COLONIZATION BEFORE AND AFTER ENDOSCOPIC RETROGRADE CHOLANGIOPANCREATOGRAPHY (ERCP)	Wrong outcomes
Nagl, S.; Ebigbo, A.; Goelder, S. K.; Roemmele, C.; Neuhaus, L.; Weber, T.; Braun, G.; Probst, A.; Schnoy, E.; Kafel, A. J.; Muzalyova, A.; Messmann, H.	2021	Underwater vs Conventional Endoscopic Mucosal Resection of Large Sessile or Flat Colorectal Polyps: A Prospective Randomized Controlled Trial	Wrong intervention
Namburar, S.; von Renteln, D.; Damianos, J.; Bradish, L.; Barrett, J.; Aguilera-Fish, A.; Cushman-Roisin, B.; Pohl, H.	2022	Estimating the environmental impact of disposable endoscopic equipment and endoscopes	Wrong outcomes
Napoleon, B.; Gonzalez, J. M.; Grandval, P.; Lisotti, A.; Laquiere, A.; Boustiere, C.; Barthet, M.; Donatelli, G.; Vanbiervliet, G.	2021	ID: 3520699 EVALUATION OF THE PERFORMANCES OF A SINGLE-USE DUODENOSCOPE: A PROSPECTIVE MULTICENTER FRENCH STUDY	Duplicate
Nct,	2023	Single-use Versus Reusable Gastroscopes in Patients With Upper Gastrointestinal Bleeding	Not empirical
NCT06369896	2024	Digital Single-operator Cholangioscopy and Intraductal Ultrasound for Nonicteric Biliary Strictures	Wrong intervention
NCT06581016	2024	Performance Characteristics and Technical Outcomes of Single-use Vs. Reusable Gastroscopes Evaluated in the or	Duplicate
Nelson, D. B.; Bosco, J. J.; Curtis, W. D.; Faigel, D. O.; Kelsey, P. B.; Laing, K.; Leung, J. W.; Mills, M. R.; Smith, P.; Tarnasky, P. R.; VanDam, J.; Wassef, W. Y.	1999	ASGE Technology status evaluation report. Automatic endoscope reprocessors. February 1999. American Society for Gastrointestinal Endoscopy	Too early
Ng, Z. Q.; Elsabagh, A.; Wijesuriya, R.	2020	Post-colonoscopy appendicitis: Systematic review of current evidence	Wrong comparator
Nguyen, N.; Toscano, L.; Schoeman, M.; Phan, V. A.; Ow, T.; Huynh, D. K.	2015	Ultra-thin disposable gastroscope is safe, feasible, and highly accurate for screening and surveillance of gastro-esophageal varices in patients with liver cirrhosis: A prospective study	Duplicate
Nielsen, K.	2023	HSD49 Impact of Endoscope Choice on Nurse Time in GI Surgical Procedures: A Prospective Study in a Danish University Hospital	Wrong outcomes
Nishimura, Y.; Watanabe, M.; Suzuki, T.	1979	Diagnosis of tumors of cholangio-pancreatic tracts by endoscopic retrograde cholangio-pancreatography (ERCP). The significance of combination of ERCP and selective arteriography (SAG) II. Diagnosis of tumors of the hepato-biliary tracts	Too early
NL-OMON38086; Universitair Medisch Centrum Utrecht, Yes	2012	E.G.Scan* research protocol: ' Disposable, transnasal versus conventional upper gastrointestinal endoscopy: a diagnostic accuracy study in patients referred for diagnostic evaluation.*	Not empirical

NL-OMON40705; Universitair Medisch Centrum Utrecht, Yes	2014	Disposable, transnasal versus conventional upper gastrointestinal endoscopy: a diagnostic accuracy study in patients referred for diagnostic evaluation of the esophagus	Not empirical
NL-OMON49147; Boston Scientific, Yes	2021	Initial Case Series with Exalt Single-Use Duodenoscope * Expanded User Experience	Not empirical
Norgaard Thomsen, E. M.; Larsen, S.; Sig Sorensen, A.	2020	The estimated contamination rate of reproccessed gastroscopes: A systematic review and meta-analysis	Wrong study design
O'Connor, K. W.	1997	ERCP recycling	Wrong study design
Ofosu, A.; Ramai, D.; Mozell, D.; Facciorusso, A.; Juakiem, W.; Adler, D. G.; Barakat, M. T.	2022	Analysis of reported adverse events related to single-use duodenoscopes and duodenoscopes with detachable endcaps	Wrong outcomes
Ofstead, C. L.; Hopkins, K. M.; Quick, M. R.; Brooks, K. B.; Eiland, J. E.; Wetzler, H. P.	2019	A Systematic Review of Disposable Sheath Use During Flexible Endoscopy	Systematic review for checking only
O'Horo, J. C.; Farrell, A.; Sohail, M. R.; Safdar, N.	2016	Carbapenem-resistant Enterobacteriaceae and endoscopy: An evolving threat	Wrong intervention
Okimoto, K.; Matsumura, T.; Fujie, M.; Akizue, N.; Matsusaka, K.; Kato, J.; Kato, N.	2023	Endoscopic submucosal dissection for early gastric cancer, using a disposable endoscope	Wrong study design
Oldin, C.; Hollaender, F.; Larsen, N.; Adamsen, S.	2022	POSB229 Organizational Assessment of Duodenoscopes in US Hospitals	Wrong outcomes
Paikos, D.; Gatopoulou, A.; Moschos, J.; Koulaouzidis, A.; Bhat, S.; Tzilves, D.; Soufleris, K.; Tragiannidis, D.; Katsos, I.; Tarpagos, A.	2007	Banding hemorrhoids using the O'Regan Disposable Bander. Single center experience	Wrong intervention
Parsi, M. A.; Sullivan, S. A.; Goodman, A.; Manfredi, M.; Navaneethan, U.; Pannala, R.; Smith, Z. L.; Thosani, N.; Banerjee, S.; Maple, J. T.	2016	Automated endoscope reprocessors	Wrong outcomes
Pascual Torres, D.; Bru Budesca, X.	2003	[Costs of digestive endoscopy in a level II university hospital]	Wrong comparator
Pasricha, P. J.; Miller, S.; Carter, F.; Humphries, R.	2020	Novel and effective disposable device that provides 2-way protection to the duodenoscope from microbial contamination	Wrong study design
Patel, N.; Chick, J. F. B.; Gemmete, J.; Pampati, R.; Johnson, E.; Srinivasa, R.	2018	Interventional radiology-operated endoscopy using the LithoVue disposable endoscope: Approach, technical success, clinical outcomes, and complications	Sample too small
Patel, N.; Srinivasa, R. N.; Gemmete, J. J.; Chick, J. F. B.	2018	Disposable single-use choledochoscopy may facilitate recanalization of occlusive biliary anastomotic strictures	Wrong study design
Perisetti, A.; Desai, M.; Bourke, M. J.; Penman, I.; Repici, A.; Reddy, D. N.; Tajiri, H.; Rex, D. K.; Hassan, C.; Sharma, P.	2023	Production and possible reduction of greenhouse gases produced during GI endoscopy activity: a systematic review of available literature	Wrong outcomes
Petersen, B. T.; Adler, D. G.; Chand, B.; Conway, J. D.; Croffie, J. M. B.; DiSario, J. A.; Mishkin, D. S.; Shah, R. J.; Somogyi, L.; Tierney, W. M.; Song, Lmwk	2009	Automated endoscope reprocessors	Wrong outcomes
Pohl, H.; Von Renteln, D.; Gayam, S.; Anderson, J.; Robertson, D.; Thiel, C.	2023	CARBON EMISSIONS FROM A FIT VERSUS A COLONOSCOPY SCREENING PROGRAM ENVIRONMENTAL IMPACT OF TRAVEL AND WASTE	Wrong outcomes
Pourghodrat, A.; Dehghani, H.; Nelson, C. A.; Oleynikov, D.; Dasgupta, P.; Terry, B. S.	2014	Disposable fluidic self-propelling robot for colonoscopy	Wrong intervention
Prat, F.; Spieler, J. F.; Paci, S.; Pallier, C.; Fritsch, J.; Choury, A. D.; Pelletier, G.; Raspaud, S.; Nordmann, P.; Buffet, C.	2004	Reliability, cost-effectiveness, and safety of reuse of ancillary devices for ERCP	Wrong outcomes
Qifeng, L. O. U.; Zhen, Jiang; Xuanli, Shen; Wencong, M. A.; Xiaofeng, Zhang	2016	Comparison of two flushing suction methods in SpyGlass choledochoscopy system	Wrong intervention

Radaelli, F.; Meucci, G.; Minoli, G.; Italian Assoc Hosp, Gastroenterolog	2008	Colonoscopy practice in Italy: A prospective survey on behalf of the Italian Association of Hospital Gastroenterologists	Wrong intervention
Ragunath, K.; Inoue, M.; Schmidt, M.; Rutland, T.; Tyler, A.; Hendrie, D.	2023	Cost analysis of reusable gastrointestinal endoscopes in a real-world setting	Wrong outcomes
Rahman, I.; Boger, P.; Patel, P.; Walter, B.; Schmidbaur, S.; Albers, D.; Schumacher, B.; Meining, A.	2019	Bougiecap dilatation device: Novel endoscopic method for treatment of oesophageal strictures-results from a multicentre study	Wrong publication type
Raijman, I.; Nguyen, D.; Fishman, D. S.	2011	Single center experience with spyglass for biliary disease	Wrong study design
Raijman, I.; Shah, R.; Brauer, B. C.; Tarnasky, P. R.; Kedia, P.; Patel, S.; Desai, A. P.; Tyberg, A.; Sharaiha, R. Z.; Kahaleh, M.	2016	Digital single operator pancreatoscopy in the management of pancreatic stones	Wrong intervention
Raijman, I.; Tarnasky, P.; Kedia, P.; Shah, R.; Nguyen, D.; Fishman, D.; Desai, A.; Patel, S.; Mondal, U.; Rosenkranz, I.; Sharaiha, R. Z.; Tyberg, A.; Kahaleh, M.	2016	Single operator digital cholangioscopy (SODC): Multicenter experience in 300 patients	Wrong intervention
Raltz, S.; Kozarek, R. A.; Kim-Deobald, J.; Pethigal, P.; Moorhouse, M. A.	1994	The impact of disposable equipment on room fee reimbursement for endoscopic retrograde cholangiopancreatography	Wrong intervention
Ramai, D.; Smit, E.; Kani, H. T.; Papaefthymiou, A.; Warner, L.; Chandan, S.; Dhindsa, B.; Facciorusso, A.; Gkolfakis, P.; Ofosu, A.; Barakat, M.; Adler, D. G.	2023	Cannulation rates and technical performance evaluation of commericially available single-use duodenoscopes for endoscopic retrograde cholangiopancreatography: A systematic review and meta-analysis	Systematic review for checking only
Rameshshanker, R.; Tsiamoulos, Z.; Wilson, A.; Rajendran, A.; Bassett, P.; Tekkis, P.; Saunders, B. P.	2020	Endoscopic cuff-assisted colonoscopy versus cap-assisted colonoscopy in adenoma detection: randomized tandem study-DEtection in Tandem Endocuff Cap Trial (DETECT)	Wrong comparator
Rameshshanker, R.; Tsiamoulos, Z.; Wilson, A.; Saunders, B.	2018	Endocuff assisted colonoscopy significantly improve adr comparing to cap assisted colonoscopy: A randomised study (DETECT)	Wrong intervention
Rao, H. B.; Pillai, K. T.; Krishnapriya, S.; Koshy, A. K.; Venu, R. P.	2020	PREVENTION OF BACTERIAL CONTAMINATION OF DUODENOSCOPES USING A DISPOSABLE ELEVATOR TIP	Wrong intervention
Rastogi, A.; Bansal, A.; Rao, D. S.; Gupta, N.; Wani, S. B.; Shipe, T.; Gaddam, S.; Singh, V.; Sharma, P.	2011	A prospective, randomized, controlled trial comparing Cap Assisted Colonoscopy (CAC) and High Definition White Light Colonoscopy (HDWL) for the detection of colon polyps	Wrong intervention
Rastogi, A.; Gupta, N.; Rao, D. S.; Wani, S.; Sharma, P.; Bansal, A.	2012	Higher detection of serrated polyps with cap assisted colonoscopy-results from a prospective, randomized controlled trial	Wrong intervention
Rauwers, A. W.; Troelstra, A.; Fluit, A. C.; Wissink, C.; Loeve, A. J.; Vleggaar, F. P.; Bruno, M. J.; Vos, M. C.; Bode, L. G.; Monkelbaan, J. F.	2019	Independent root-cause analysis of contributing factors, including dismantling of 2 duodenoscopes, to investigate an outbreak of multidrug-resistant Klebsiella pneumoniae	Wrong intervention
Repici, A.; Khalaf, K.; Troncone, E.; Subramaniam, S.; Hassan, C.; Bhandari, P.	2024	International Delphi Consensus Study on disposable single-use endoscopy: A path to clinical adoption	Not empirical
Rerknimitr, R.	2024	Getting the Bugs Out: Disposable Duodenoscopes Have a Shallower Learning Curve but Face a Steep Climb in Usability	Not empirical
Rex, D. K.; Khashab, M.; Raju, G. S.; Pasricha, J.; Kozarek, R.	2005	Insertability and safety of a shape-locking device for colonoscopy	Wrong intervention
Ribeiro, T.; Morais, R.; Monteiro, C.; Carvalho, A.; Barros, S.; Fernando, A.; Pioche, M.; de Santiago, E. R.; Macedo, G.	2024	Estimating the environmental impact of endoscopic activity at a tertiary center: a pilot study	Wrong outcomes
Ribeiro, T.; Morais, R.; Monteiro, C.; Carvalho, A.; Barros, S.; Macedo, G.	2022	ESTIMATING THE ENVIRONMENTAL IMPACT OF ENDOSCOPIC ACTIVITY AT A TERTIARY CENTER: A PILOT STUDY	Wrong outcomes
Rismani, M.; Jones, W.; Oza, V. M.	2023	Usage of Single-Use Duodenoscope Is Not Cost Prohibitive: A Single-Center Experience	Wrong outcomes

Rivallin, P.; Lorenzo, D.; Hedjoudje, A.; Prat, F.	2022	Single-use duodenoscopes are an efficient tool for emergency ERCP in real life	Sample too small
Rizzo, J.; Bernstein, D.; Gress, F.	2000	A performance, safety and cost comparison of reusable and disposable endoscopic biopsy forceps: a prospective, randomized trial	Wrong intervention
Robles-Medranda, C.; Arevalo-Mora, M.; Puga-Tejada, M.; Egas-Izquierdo, M.; Baquerizo-Burgos, J.; Alcivar-Vasquez, J.; Del Valle, R.; Cohn, J.; Corbett, S.; Steinberg, S.	2022	SINGLE-USE ULTRASOUND ENDOSCOPE ATTACHED TO A STANDARD SCOPE: FIRST HUMAN CASES AND RESULTS OF A PROSPECTIVE PILOT STUDY	Sample too small
Robles-Medranda, C.; Oleas, R.; Alcivar-Vasquez, J.; Pitanga-Lukashok, H.	2020	Sterile disposable elevator cap in the reduction of cross-contamination	Wrong study design
Romagnuolo, J.; Currie, G.	2005	Noninvasive vs. selective invasive biliary imaging for acute biliary pancreatitis: an economic evaluation by using decision tree analysis	Wrong comparator
Rosch, T.; Adler, A.; Pohl, H.; Wettschureck, E.; Koch, M.; Wiedenmann, B.; Hoepffner, N.	2008	A motor-driven single-use colonoscope controlled with a hand-held device: a feasibility study in volunteers	Wrong patient population
Ross, A. S.; Baliga, C.; Verma, P.; Duchin, J.; Gluck, M.	2015	A quarantine process for the resolution of duodenoscope-associated transmission of multidrug-resistant Escherichia coli	Wrong intervention
Rosztoczy, A.; Roka, R.; Vadaszi, K.; Benko, E.; Pak, P.; Wittmann, T.; Madacsy, L.	2013	The role of transnasal capsule esophagoscope (e.g scan) for the prediction of high risk endoscopic lesions in patients with upper GI bleeding: A pilot trial of feasibility, safety, and utility	Sample too small
Rothstein, R. I.; Littenberg, B.	1995	Disposable, sheathed, flexible sigmoidoscopy: a prospective, multicenter, randomized trial. The Disposable Endoscope Study Group	Too early
Rothstein, R. I.; Littenberg, B.	1993	DISPOSABLE SHEATHED FLEXIBLE SIGMOIDOSCOPY - A PROSPECTIVE MULTICENTER RANDOMIZED TRIAL	Wrong publication type
Rubio, C. A.; Slezak, P.; Befrits, R.	1994	The costs of colonoscopy in patients with ulcerative pan-colitis in Sweden	Wrong intervention
Rustagi, T.; Irani, S.; Reddy, D. N.; Abu Dayyeh, B. K.; Baron, T. H.; Gostout, C. J.; Levy, M. J.; Martin, J.; Petersen, B. T.; Ross, A.; Topazian, M. D.	2017	Radiofrequency ablation for intraductal extension of ampullary neoplasms	Wrong intervention
Rybinski, F.; Winters, M.; Abshagen, C.; Niess, J.; Zimmerli, M.; Heinrich, H.	2023	Waste audit- Swiss data on waste production and composition in a large endoscopy unit	Wrong outcomes
Saji, A. P.; Soliman, F.; Horwood, J.; Hargest, R.	2023	High resolution anoscopy for anal intraepithelial neoplasia surveillance: new service delivery in Wales	Wrong intervention
Sakai, Paulo; Sugai, Beatriz Monica; Maluf Filho, Fauze; Guadagnin, Aurora R.; Abe, Mineo; Tomishigue, Toshiro; Andreolli, JoÃ¤o Carlos; Ishioka, Shinichi	1992	PrevenÃ§Ã¤o da transmissÃ¤o do vÃrus B da hepatite durante o programa de escleroterapia endoscÃ³pica das varizes esofÃ¡gicas	Too early
Saleem, N.; Ali, F.; Kamal, F.; Sherman, S.; Gromski, M.	2021	Adverse events associated with the use of FDA-approved innovative duodenoscope designs: An analysis of medical device reports (MDRS) from the manufacturer and user facility device experience (MAUDE) database	Wrong study design
Sami, S. S.; Subramanian, V.; Ortiz-Fernandez-Sordo, J.; Saeed, A. H.; Singh, S.; Iyer, P. G.; Ragunath, K.	2014	Performance characteristics of unsedated ultrathin video endoscopy in the assessment of the upper gastrointestinal (GI) tract: Systematic review and meta-analysis	Wrong intervention
Sandler, R. S.; Cummings, M. S.; Keku, T. O.; Terse, A.; Mehta, N.	2000	Disposable versus reusable biopsy forceps for colorectal epithelial cell proliferation in humans	Wrong intervention
Sardinha, T. C.; Wexner, S. D.; Gilliland, J.; Daniel, N.; Kroll, M.; Lee, E.; Wexler, J.; Hudzinski, D.; Glass, D.	1997	Efficiency and productivity of a sheathed fiberoptic sigmoidoscope compared with a conventional sigmoidoscope	Sample too small
Sawatzki, M.; Brand, S.; Franke, A.; Hauptle, R.; Frei, N.	2020	Virtual chromoendoscopy combined with endocuff for screening colonoscopy is feasible achieving a high adenoma detection rate: results of a prospective single center study	Wrong intervention

Schafer, T. W.; Hollis-Perry, K. M.; Mondragon, R. M.; Brann, O. S.	2002	An observer-blinded, prospective, randomized comparison of forceps for endoscopic esophageal biopsy	Wrong intervention
Schoalvinck, D.; Bergman, J.; Kestens, C.; Siersema, P.; Vleggaar, F.; Canto, M.; Cosby, H.; Abrams, J.; Lightdale, C.; Tejeda-Ramirez, E.; DeMeester, S.; Greene, C.; Jobe, B.; Peters, J.; Weusten, B.	2013	Clinical evaluation of a new cryoballoon focal ablation system for the elimination of Barrett's esophagus	Wrong study design
ScholLvinck, D.; Bergman, J. J.; Kestens, C.; Siersema, P. D.; Vleggaar, F. P.; Canto, M. I.; Cosby, H.; Abrams, J. A.; Lightdale, C. J.; Tejeda-Ramirez, E.; Demeester, S. R.; Greene, C. L.; Jobe, B. A.; Peters, J. H.; Weusten, B. L.	2014	Clinical evaluation of a new cryoballoon focal ablation system for the elimination of Barrett's esophagus	Wrong intervention
Scholvinck, D.; Bergman, J.; Kestens, C.; Siersema, P.; Vleggaar, F.; Canto, M.; Cosby, H.; Abrams, J.; Lightdale, C.; Tejeda-Ramirez, E.; Demeester, S.; Greene, C.; Jobe, B.; Peters, J.; Weusten, B.	2014	Clinical evaluation of a new cryoballoon focal ablation system for the elimination of Barrett's esophagus	Wrong intervention
Scholz, P. M.; Kirstein, M. M.; Solbach, P. C.; Vonberg, R. P.	2023	[A systematic analysis of nosocomial outbreaks of nosocomial infections after gastrointestinal endoscopy]	Wrong study design
Schroy, P. C., 3rd; Wilson, S.; Afdhal, N.	1996	Feasibility of high-volume screening sigmoidoscopy using a flexible fiberoptic endoscope and a disposable sheath system	Too early
Sekine, K.; Yasuda, I.; Sato, K.; Doi, S.; Mabuchi, M.; Takano, Y.; Tsujikawa, T.; Kuniyoshi, N.; Iwashita, T.; Miyazaki, T.; Kawamoto, M.; Takahashi, M.	2018	Comparison of the diagnostic sensitivity of a novel peroral cholangioscopy-guided target biopsy to that of conventional endoscopic transpapillary forceps biopsy in patients with suspected bile duct cancer	Wrong publication type
Seva-Pereira, G.; Trombeta, V. L.; Capochim Romagnolo, L. G.	2014	Transanal minimally invasive surgery (TAMIS) using a new disposable device: our initial experience	Wrong intervention
Shah, R. J.; Raijman, I.; Brauer, B.; Gumustop, B.; Pleskow, D. K.	2017	Performance of a fully disposable, digital, single-operator cholangiopancreatoscope	Wrong intervention
Shah, R. J.; Raijman, I.; Hawes, R.; Gumustop, B.; Hasan, M.; Brauer, B.; Fukami, N.; Pleskow, D. K.	2015	A U.S. multi-center first human use experience using the fully disposable, digital single-operator cholangiopancreatoscope (DSOCP)	Wrong intervention
Shahid, H. M.; Tyberg, A.; Bareket, R.; Sarkar, A.; Simon, A.; Gurram, K.; Gress, F. G.; Bhenswala, P.; Chalikonda, D. M.; Loren, D. E.; Kowalski, T. E.; Kumar, A.; Vareedayah, A. A.; Abhyankar, P. R.; Parker, K.; Gabr, M. M.; Nieto, J.; De Latour, R.; Zolotarevsky, M. V.; Barber, J.; Zolotarevsky, E.; Vazquez-Sequeiros, E.; Gaidhane, M.; Andalib, I.; Kahaleh, M.	2022	COMPARING THE SAFETY AND EFFICACY OF TWO COMMERCIALLY AVAILABLE SINGLE-USE DUODENOSCOPES: A MULTICENTER STUDY	Duplicate
Shamah, S. P.	2024	Let's cut to the "core" of the issue!	Wrong comparator
Shellenberger, M.; Diehl, D.	2010	"Real life" experience with spyglass: Outcomes and costs	Wrong outcomes
Shenoy, E. S.; Pierce, V. M.; Walters, M. S.; Moulton-Meissner, H.; Lawsin, A.; Lonsway, D.; Shugart, A.; McAllister, G.; Halpin, A. L.; Zambrano-Gonzalez, A.; Ryan, E. E.; Suslak, D.; DeJesus, A.; Barton, K.; Madoff, L. C.; McHale, E.; DeMaria, A.; Hooper, D. C.	2019	Transmission of Mobile Colistin Resistance (mcr-1) by Duodenoscope	Wrong intervention

Shi, D.; Wang, Q. Z.	2012	Clinical effects of using a reloading multiband ligator with an SD-c conductor for endoscopic variceal ligation over subsequent sessions	Wrong intervention
Shiha, M. G.; Nandi, N.; Hutchinson, A. J.; Raju, S. A.; Tai, F. W. D.; Elli, L.; Penny, H. A.; Sanders, D. S.	2024	Cost-benefits and environmental impact of the no-biopsy approach for the diagnosis of coeliac disease in adults	Wrong outcomes
Shike, M.; Fireman, Z.; Eliakim, R.; Segol, O.; Sloyer, A.; Cohen, L. B.; Goldfarb-Albak, S.; Repici, A.	2008	Sightline ColonoSight system for a disposable, power-assisted, non-fiber-optic colonoscopy (with video)	Too early
Shin, I. L. S.; Moon, J. H.; Lee, Y. N.; Kim, H. K.; Lee, T. H.; Yang, J. K.; Cha, S. W.; Cho, Y. D.; Park, S. H.	2022	Comparison of Disposable Digital Single-Operator Cholangioscopy Versus Direct Peroral Cholangioscopy for the Management of Intraductal Superficial Lesions of the Bile Duct	Wrong intervention
Shin, I. S.; Moon, J. H.; Lee, Y. N.; Kim, H. K.; Chung, J. C.; Lee, T. H.; Yang, J. K.; Cho, Y. D.; Park, S. H.	2024	Comparison of disposable digital single-operator cholangioscopy versus direct peroral cholangioscopy for the diagnosis of intraductal superficial lesions of the bile duct	Wrong intervention
Shin, I. S.; Moon, J. H.; Lee, Y. N.; Myeong, J. H.; Park, J. W.; Lee, T. H.; Yang, J. K.; Cho, Y. D.; Park, S. H.	2023	Comparison of Disposable Digital Single-Operator Cholangioscopy Versus Direct Peroral Cholangioscopy for the Management of Intraductal Superficial Lesions of the Bile Duct	Wrong intervention
Shiraishi, F.; Hiratsuka, H.; Ebata, K.	1992	[Utility of SCG (screening colonography) and combined sigmoidoscopy-SCG in thorough physical checkups]	Too early
Skinner, M. J.; Storm, A. C.; Thompson, C. C.	2017	Novel disposable endoscopic scissors: A comparative study with traditional reusable scissors	Wrong intervention
Slivka, A.; Beyna, T.; Carr-Locke, D.; Chahal, P.; Costamagna, G.; Devereaux, B.; Giovannini, M.; Goenka, M.; Khor, C.; Lau, J.; May, G.; Muthusamy, V. R.; Patel, S.; Petersen, B.; Pleskow, D.; Raijman, I.; Duvvur, N.; Repici, A.; Ross, A.; Sejpal, D.; Sherman, S.; Siddiqui, U.; Ziady, C.; Peetermans, J.; Rousseau, M.; Bruno, M.	2023	GLOBAL PROSPECTIVE CASE SERIES OF ERCP USING A SINGLE-USE DUODENOSCOPE	Duplicate
Slivka, A.; Ross, A.; Sejpal, D. V.; Petersen, B. T.; Bruno, M. J.; Pleskow, D. K.; Muthusamy, V. R.; Chennat, J.; Calvin, L.; Krishnamoorthi, R.; Martin, J. A.; Chandrasekhara, V.; Law, R.; Abu Dayyeh, B. K.; Levy, M. J.; Storm, A. C.; Poley, J. W.; Cohen, J. M.; Thaker, A. M.; Peetermans, J.; Rousseau, M.; Tirrell, G. P.; Kozarek, R. A.	2021	ID: 3520553 A SINGLE-USE DUODENOSCOPE FOR ENDOSCOPIC RETROGRADE CHOLANGIOPANCREATOGRAPHY PERFORMED BY ENDOSCOPISTS WITH A WIDE RANGE OF EXPERIENCE	Duplicate
Smadi, Y.; Thomas, J.; Bittar, K.; Friedlander, J.; Bornstein, J.	2023	OFFICE-BASED UNSEDATED TRANSNASAL ESOPHAGOGASTRODUODENOSCOPY (TN-EGD) USING SINGLE-USE GASTROSCOPES: A PEDIATRIC EOSINOPHILIC ESOPHAGITIS SINGLE CENTER EXPERIENCE	Sample too small
Sonaiya, S.; Marino, R.; Agollari, K.; Sharma, P.; Desai, M.	2023	Environmentally sustainable gastroenterology practice: Review of current state and future goals	Wrong outcomes
Stipa, F.; Tierno, S. M.; Russo, G.; Burza, A.	2022	Trans-anal minimally invasive surgery (TAMIS) versus trans-anal endoscopic microsurgery (TEM): a comparative case-control matched-pairs analysis	Wrong intervention
Stolz, C.; Ghumman, A.; Wong, M.; Bonnichsen, M.; Saxena, P.; Kaffes, A.	2019	Safety and feasibility of the Endotics robotic colonoscopy system in select patients: Interim analysis of a prospective study	Sample too small
Stromstad Travis, H.; Larsen, S.; Vinther Russell, R.; Holger Ehlers, L.	2020	A cost-utility analysis comparing the total cost of reusable duodenoscopes to single-use duodenoscopes	Wrong outcomes

Swaroop, V. S.; Dhir, V.; Mohandas, K. M.; Wagle, S. D.; Vazifdar, K. F.; Gopalakrishnan, G.; Sharma, O. P.; Jagannath, P.; Desouza, L. J.	1997	Endoscopic palliation of malignant obstructive jaundice using resterilized accessories: an audit of success, complications, mortality and cost	Wrong intervention
Tai, F. W. D.; Healy, A.; Thokala, P.; Zammit, S. C.; Sidhu, R.; McAlindon, M.	2023	Cost comparison of oral, transnasal and magnet assisted capsule endoscopy in the examination of the upper gastrointestinal tract in patients with dyspepsia	Wrong publication type
Talati, R. K.; Goodman, A.; Pochapin, M. B.	2021	Medical device safety in gastroenterology: FDA recalls of duodenoscopes, 2015-2020	Wrong intervention
Talento, P.; Ferreri, G.; Maggiore, S.; Cudazzo, E.; De Cesare, C.; Milazzo, F.	2022	High resolution anoscopy in patients with anal dysplasia	Wrong study design
Tampio, A. J. F.; Marzouk, M. F.	2021	Robot-assisted sialolithotomy with sialoendoscopy: a review of safety, efficacy and cost	Wrong intervention
Tannapfel, A.; Aust, D.; Baretton, G. B.; Blaker, H.; Esposito, I.; Langner, C.; Vieth, M.; Wardelmann, E.; Kirchner, T.; Schirmacher, P.	2015	[Remove polyps and discard. Way or wrong way?]	Foreign language
TCTR20220912003; Thailand Science, Research; Innovation Fund Chulalongkorn University, Yes	2022	Comparison of bacterial contamination of disposable distal cap duodenoscopes and detachable elevator duodenoscopes after manual cleaning, a randomized trial	Not empirical
Thavamani, A.; Ryan, M.; Leinwand, K.; Ramraj, R.; Schroeder, S.; Menard-Katcher, P.; Bhardwaj, V.; Franciosi, J.; Friedlander, J.; Sabe, R.	2023	THE USE OF A NOVEL ULTRATHIN GASTROSCOPE FOR UNSEDATED TRANSNASAL ENDOSCOPY IN CHILDREN AND ADULTS FOR EVALUATION OF UPPER GASTROINTESTINAL DISORDERS	Sample too small
Thavamani, A.; Ryan, M.; Leinwand, K.; Ramraj, R.; Schroeder, S.; Menard-Katcher, P.; Bhardwaj, V.; Friedlander, J.; Sabe, R.	2023	USE OF A NOVEL ULTRATHIN GASTROSCOPE FOR UNSEDATED TRANSNASAL ENDOSCOPY IN CHILDREN AND ADULTS FOR EVALUATION OF UPPER GASTROINTESTINAL TRACT DISORDERS	Sample too small
Thomas, R. A.; Rao, D. S.; Gupta, N.; Gaddam, S.; Wani, S. B.; Singh, V.; Shipe, T.; Sharma, P.; Bansal, A.; Rastogi, A.	2011	Does cap-assisted colonoscopy (CAC) significantly impact surveillance interval recommendations compared to standard colonoscopy (SC)? Results from a randomized controlled trial	Wrong publication type
Thosani, N.; Alcivar, J. M.; Siddiqui, S.; Pawa, S.; Rogith, D.; Oleas, R.; Patil, P.; Dedania, B.; Rashtak, S.; Pawa, R.; Badillo, R.; Ramireddy, S.; Guha, S.; DaVee, T.; Robles-Medranda, C.	2020	Prospective Multicenter International Trial Evaluating Performance of Disposable Elevator Cap Duodenoscope during Endoscopic Retrograde Cholangiopancreatography (deCAP Trial)	No endoscope data for case series
Tinckler, L. F.	1990	Choledochoscopy via the cystic duct: evaluation of a new ultrathin disposable choledochoscope	Too early
Tomiato, L. H.; Maluf-Filho, F.	2011	Disposable versus reusable biopsy endoscopic forceps: A cost analysis	Wrong intervention
Traverso, G.	2015	A self-propelled colon scope	Wrong study design
Trindade, A. J.; Copland, A.; Bhatt, A.; Bucobo, J. C.; Chandrasekhara, V.; Krishnan, K.; Parsi, M. A.; Kumta, N.; Law, R.; Pannala, R.; Rahimi, E. F.; Saumoy, M.; Trikudanathan, G.; Yang, J.; Lichtenstein, D. R.	2021	Single-use duodenoscopes and duodenoscopes with disposable end caps	Not empirical
Tumino E; Sacco R; Bertini M; Bertoni M; Parisi G; Capria A	2010	Endotics system vs colonoscopy for the detection of polyps.	Wrong intervention
Turk, D. J.; Kozarek, R. A.; Botoman, V. A.; Patterson, D. J.; Ball, T. J.	1991	Disposable endoscopic biopsy forceps: comparison with standard forceps of sample size and adequacy of specimen	Sample too small
Udine, M.; Huff, M. L.; Tsay, K.; Diab, A. F.; Sujka, J.; DuCoin, C.; Docimo, S., Jr.	2024	Disposable Gastrointestinal Scopes: A Systematic Review	Systematic review for checking only
Urayama, S.; Kozarek, R.; Raltz, S.	1996	Evaluation of per-procedure equipment costs in an outpatient endoscopy center	Too early

Uribe Rivera, A. K.; Seeliger, B.; Saldivar, C. A.; Houghton, E.; Rodriguez, F.; Acquafresca, P.; Palermo, M.; Gimenez, M. E.	2023	Percutaneous transhepatic cholangioscopy for benign and malignant biliary disease using a novel short single-operator cholangioscope	Wrong intervention
Valderrama, S.; Miranda, C. J. L.; Rubio, A. P. G.; Gualtero, S.; Fraile, G. C. C.; Escobar, Y.; Amador, A. C. H.	2016	Successful control of an endoscopic retrograde cholangiopancreatography-associated nosocomial outbreak caused by klebsiella pneumoniae carbapenemase producing klebsiella pneumoniae in a university hospital in Bogota, Colombia	Wrong publication type
van der Ploeg, K.; de Jonge, P. J. F.; Lammers, W. J.; Koch, A. D.; Vos, M. C.; Paulsen, V.; Aabakken, L.; Bruno, M.	2024	Performance of a single-use gastroscope for esophagogastroduodenoscopy: Prospective evaluation	Duplicate
Van Remoortel, H.; Borra, V.; De Buck, E.; Compernolle, V.; Vandekerckhove, P.	2018	Is an endoscopic examination associated with transfusion-transmissible infections? A systematic review and meta-analysis	Wrong outcomes
Van Steenbergen, W.; Van Aken, L.; Volders, W.; Kesteloot, K.	2001	Cost analysis of ERCP in a university hospital	Wrong comparator
Varma, P.; Bonney, D.; Swan, M. P.	2018	The use of Endocuff Vision in the detection of polyps during colonoscopy: A preliminary analysis	Wrong intervention
Vasudeva, R.; Sheley, J.; Woods, B.; Greenhalgh, J.; Howden, C. W.	1995	HIGH-VOLUME SCREENING FOR COLORECTAL NEOPLASIA USING A DISPOSABLE SHEATH FLEXIBLE SIGMOIDOSCOPY (FS) SYSTEM	Wrong publication type
Walker, R. S.; Vanagunas, A. D.; Williams, P.; Chodash, H. B.	1997	Therapeutic ERCP: a cost-prohibitive procedure?	Too early
Walter, B.; Schmidbaur, S.; Rahman, I.; Hann, A.; Duarte, P.; Meining, A.	2018	The bougiecap: A new method for treatment of oesophageal strictures	Wrong intervention
Wang P; Xu T; Ngamruengphong S; Makary MA; Kalloo A; Hutfless S	2018	Rates of infection after colonoscopy and osophagogastroduodenoscopy in ambulatory surgery centres in the USA.	Wrong outcomes
Wang, M.; Snyder, G. M.	2023	Duodenoscopes With Disposable Elevator Caps-An Incremental Reduction in Infection Risk for Patients	Wrong study design
Waye, J. D.; Heigh, R. I.; Fleischer, D. E.; Leighton, J. A.; Gurudu, S.; Aldrich, L. B.; Li, J.; Ramrakhiani, S.; Edmundowicz, S. A.; Early, D. S.; Jonnalagadda, S.; Bresalier, R. S.; Kessler, W. R.; Rex, D. K.	2010	A retrograde-viewing device improves detection of adenomas in the colon: a prospective efficacy evaluation (with videos)	Wrong intervention
Wendorf, K. A.; Kay, M.; Baliga, C.; Weissman, S. J.; Gluck, M.; Verma, P.; D'Angeli, M.; Swoveland, J.; Kang, M. G.; Eckmann, K.; Ross, A. S.; Duchin, J.	2015	Endoscopic retrograde cholangiopancreatography-associated AmpC Escherichia coli outbreak	Wrong outcomes
Willcox, J.; Turner, M.	2011	Comparison of Ambu aScope and Olympus re-usable fibrescope	Wrong study design
Willmore, J.; Ellis, E.; Etches, V.; Labrecque, L.; Osiowy, C.; Andonov, A.; McDermaid, C.; Majury, A.; Achonu, C.; Maher, M.; MacLean, B.; Levy, I.	2015	Public health response to a large-scale endoscopy infection control lapse in a nonhospital clinic	Wrong outcomes
Woods, A.; Nguyen, C. C.; Islam, M. S. U.; Lovell, N. H.; Nho Do, T.; Tsai, D.	2023	Towards a single-use, low-cost endoscope for gastroenterological diagnostics	Wrong study design
Woods, K. L.; Anand, B. S.; Cole, R. A.; Osato, M. S.; Genta, R. M.; Malaty, H.; Gurer, I. E.; Rossi, D. D.	1999	Influence of endoscopic biopsy forceps characteristics on tissue specimens: results of a prospective randomized study	Too early
Wu, W.; Han, Y.; Kong, D.	2022	A novel technique for endoscopic removal of foreign bodies using a balloon and transparent cap	Wrong study design
Yadlapati, S.; Jarrett, S.; Lo, K.; Baik, D.	2023	Performance and Pooled Success Rate of Single Use Duodenoscopes for Endoscopic Retrograde Cholangiopancreatography	Systematic review for checking only
Yan, K.; Van Munster, S.; Chen, J.; Liu, F.; Zhao, D.; Li, W.; He, S.; Zhang, Y.; Dou, L.; Liu, Y.; Liu, X.;	2018	Endoscopic cryoballoon ablation is safe, welltolerated and highly effective in the eradication of esophageal squamous cell neoplasia	Wrong comparator

Xue, L.; Lu, N.; Dawsey, S.; Bergman, J. J.; Weusten, B. L. A. M.; Wang, G. Q.			
Yang, R.; Naritoku, W.; Laine, L.	1994	Prospective, randomized comparison of disposable and reusable biopsy forceps in gastrointestinal endoscopy	Too early
Yang, R.; Naritoku, W.; Laine, L.	1993	PROSPECTIVE RANDOMIZED COMPARISON OF DISPOSABLE AND REUSABLE BIOPSY FORCEPS IN GASTROINTESTINAL ENDOSCOPY	Too early
Yang, R.; Ng, S.; Nichol, M.; Laine, L.	2000	A cost and performance evaluation of disposable and reusable biopsy forceps in GI endoscopy	Wrong intervention
Yang, R.; Vuitch, F.; Wright, K.; McCarthy, J.	1990	Adequacy of disposable biopsy forceps for gastrointestinal endoscopy: a direct comparison with reusable forceps	Too early
Yano, T.	2023	Impact and potential use of disposable single-use endoscopes	Not empirical
Yetkin, F.; Ersoy, Y.; Kuzucu, C.; Otlu, B.; Parmaksiz, N.; Seckin, Y.	2017	An outbreak associated with multidrug-resistant pseudomonas aeruginosa contamination of duodenoscopes and an automated endoscope reprocessor	Wrong intervention
Yzet, C.; Figueiredo, M.; Michoud, C.; Lafeuille, P.; Rivory, J.; Pioche, M.	2022	Ecological impact of endoscopic dilatation using the bougie cap device: a low-tech innovation to reduce waste by 99 %	Wrong study design
Zeb, S.; Haider, W.; Rajput, M. S.; Arshad, I.; Memon, G. F.; Keerio, S. H.	2022	Diagnostic Accuracy of Serum Albumin for Diagnosis of Esophageal Varices among Patients of Chronic Liver Disease taking Endoscopy as Gold Standard	Wrong comparator
Zhang, Q.; Cai, J. Q.; Wang, Z.	2021	Usefulness of tumor traction with a snare and endoclips in gastric submucosal tumor resection: a propensity-score-matching analysis	Wrong comparator
Zhang, Q.; Lian, Z. Y.; Cai, J. Q.; Bai, Y.; Wang, Z.	2022	Safety and effectiveness of mucosal traction using a snare combined with endoclips to assist the resection of esophageal intraepithelial neoplasia: a propensity score matching analysis	Wrong comparator
Zhang, T.; Xu, L. J.; Xiang, J.; He, Z.; Peng, Z. Y.; Huang, G. M.; Ji, G. Z.; Zhang, F. M.	2015	Cap-assisted endoscopic sclerotherapy for hemorrhoids: Methods, feasibility and efficacy	Wrong intervention
Zhang, X.; Kong, J.; Tang, P.; Wang, S.; Hyder, Q.; Sun, G.; Zhang, R.; Yang, Y.	2011	Current status of cleaning and disinfection for gastrointestinal endoscopy in China: a survey of 122 endoscopy units	Wrong outcomes
Zhu, R.; Zhao, Q.	2023	Clinical trial study on minimally invasive treatment of internal hemorrhoids under endoscope by disposable electronic gastroscope	Wrong intervention
Zuber, T. J.	1994	Disposable sheath endoscopes: a new office technology	Too early
Zuckerman, M.; Michael, M.; Bashashati, M.; Jia, Y.; Othman, M.; Carrion, A.; Dodoo, C.; Dwivedi, A.; Elhanafi, S.	2020	Outcomes of retrograde enteroscopy using a through-the-scope balloon device: A single-center experience	Wrong intervention
Zullo, A.; Chiovelli, F.; Esposito, E.; Hassan, C.; Casini, B.	2023	Can Gastric Juice Analysis with EndoFasterÂ® Reduce the Environmental Impact of Upper Endoscopy?	Wrong outcomes
NCT06543316	2024	Accelerated Marginal Ulcer Healing With Low-Thermal Argon Plasma Endoscopic Treatment	Wrong intervention
NCT02243930	2014	Cap-assisted Sigmoidoscopy - Reduction in Pain, Reduction in Investigation Time and Increased Rate of Success, When Examined by the Less-experienced Endoscopist. A Prospective Randomized Clinical Trial	Duplicate
NCT00990782	2009	CEPELA I Trial: Capsule Endoscopy for Post-Ablation Esophageal Lesion Assessment	Wrong intervention
NCT04040504	2019	Clinical Efficacy and Infection Risk of ERCP Using a Novel Duodenoscope With a Disposable Cap: a Randomized Controlled Trial	Duplicate
NCT05429203	2022	Comparison of Outcomes in Patients Undergoing Endoscopic Retrograde Cholangiopancreatoscopy (ERCP) Using Duodenoscope With Single-use Distal Cover and Conventional Reusable Duodenoscope: A Randomized Controlled Trial	Duplicate
NCT02759094	2016	Evaluation of Safety and Effectiveness of the RefluxStop Device in the Management of Gastroesophageal Reflux Disease (GERD)	Wrong intervention

NCT01085513	2010	Intestinal Motility Evaluation by Endoluminal Image Analysis Using Capsule Endoscopy (CE-EIA): a Multi-center Clinical Trial	Duplicate
NCT05822635	2023	Intraoperative Endoscopy Using a Thin, Single-Use, Flexible Cholangiopancreatoscope	Sample too small
NCT04724291	2020	Magnetically Controlled Capsule for Assessment of Gastric Mucosa in Symptomatic Patients	Duplicate
NCT04447976	2020	A Multi-center Prospective Study to Evaluate Performance of Disposable TIP Duodenoscope Designed to Prevent Future Outbreak Related to Contaminated Duodenoscope	Duplicate
NCT05225909	2022	Multi-centre Prospective Observational Cohort Study: To Assess the Performance of Single Use Duodenoscope (aScope)	Study ongoing
NCT04712253	2021	Multicenter Retrospective International Study to Evaluate Outcomes and Safety of Patients Undergoing ERCP Using a Single-use Cholangioscope (SpyGlass DS II) and Single-use Duodenoscope (Exalt) - The MESE Study	Study ongoing
NCT04197843	2019	Navigational Capsule Endoscopy as a Triage Tool in the Management of Hemodynamically Stable Patients With Acute Upper Gastrointestinal Bleeding a Single Center Prospective Cohort Study	Duplicate
NCT02495480	2015	A Novel Disposable Sheathed Gastroscopic System for Cecal Intubationï¼šSafety and Efficacy in Unsedated Colonoscopies	Duplicate
NCT05211258	2021	A Novel Portable Upper Gastrointestinal Endoscopy System Applied in Remote Populations: A Pilot Study	Sample too small
NCT03205072	2017	Per-Oral Cholangioscopy in Liver Transplantation Patients	Wrong intervention
NCT06581016	2024	Performance Characteristics and Technical Outcomes of Single-use Versus Reusable Gastroscopes Evaluated in the Operating Room in Acute, Subacute and Elective Patients	Study ongoing
NCT05303662	2022	Prevalence of Multidrug Resistant Micro-organism Carriage in Patients Undergoing an Endoscopic Retrograde Cholangiopancreatography in Four Different Countries	Duplicate
NCT04671095	2020	Prospective Observational Cohort Study: To Assess the Performance of Single Use Duodenoscope	Duplicate
NCT04401033	2020	Prospective Observational Study Evaluating the Increased Risk of SARS-CoronaVirus-2 (SARS-CoV-2) Infection Associated With Endoscopic Procedures (DECORE Study)	Duplicate
NCT04143698	2019	Randomized Trial Comparing Reusable and Disposable Duodenoscopes for ERCP	Duplicate
NCT04687774	2020	Real World Study of Exalt D Single-use Duodenoscope in ERCP Procedures in China	Sample too small
NCT04210401	2019	Registry of Culture Results From Reusable Duodenoscopes	Study ongoing
NCT03155165	2016	Retroviewâ„¢ vs. Conventional Colonoscopy: it is Time to Change?	Wrong intervention
NCT02498041	2015	Study to Compare the Efficacy and Patient Tolerance of Ultrathin Nasal Endoscopy to Detect Barrett's Esophagus Compared With Conventional Endoscopy to Inform a Future Multicentre Screening Trial	Wrong intervention
NCT06200961	2023	Use of Sedation-Free Transnasal Endoscopy to Improve Access and Lower Costs of Endoscopic Evaluations in a Bariatric Medical and Surgical Program	Study ongoing
NCT06369896	2024	The Usefulness of Disposable Digital Single-operator Cholangioscopy and Intraductal Ultrasound in the Assessment of Nonicteric Biliary Strictures: A Prospective Observational Study	Study ongoing
NCT02609100	2015	Video Capsule Endoscopy Versus Colonoscopy in Patients With Melena and Negative Upper Endoscopy: A Randomized Controlled Trial	Sample too small
