# Supplementary material for: Diagnostic and Clinical Performance of Single‐Use Versus Reusable Gastrointestinal Endoscopes: A Systematic Review and Meta‐Analyses
Source: J Gastroenterol Hepatol. 2026 Aug 7;41(9):2571–87. doi: 10.1111/jgh.70637 (PMC13534234; doi:10.1111/jgh.70637)
Supplement: Supplementary file 4 [file JGH-41-2571-s004.pdf]

Diagnostic and clinical performance of single-use versus reusable gastrointestinal endoscopes: a systematic review and meta-analyses

Supporting information Data S5: Risk of bias tables

Authors

Natalie Tyldesley-Marshall, Yen-Fu Chen,* Jefferson Buendia, Anna Brown, Thomas Matthews, Janette Parr, Shivashri Chockalingam, Adel Elfeky, Amy Grove, Lazaros Andronis, Stuart R Coles, Mandana Zanganeh, Bu Hayee, Anjan Dhar, Shaji Sebastian, Ramesh Arasaradnam on behalf of the SUMU-Endo Project

*Correspondence to: Prof. Yen-Fu Chen

Centre for Evidence and Implementation Science, Park House, Edgbaston, University of Birmingham, Birmingham B15 2TT, United Kingdom

y.chen.25@bham.ac.uk

Department of Health and Welfare, College of City Management, University of Taipei, Taipei, Taiwan

yfchen@utaipei.edu.tw

Risk of bias tables

1. COCHRANE RISK OF BIAS: Duodenoscopes

Author (year)	Risk of bias arising from randomization process	Risk of bias due to deviations from intended interventions	Risk of bias from missing outcomes	Risk of bias in measurement of the outcome	Risk of bias in selection of the reported result	Overall risk of bias
Bang et al., 2021	LOW	SOME CONCERNS	LOW	SOME CONCERNS	LOW	SOME CONCERNS
Forbes et al., 2023	LOW	LOW	LOW	LOW	LOW	LOW

2. COCHRANE RISK OF BIAS: Other upper GI endoscopes

Author (year)	Risk of bias arising from randomization process	Risk of bias due to deviations from intended interventions	Risk of bias from missing outcomes	Risk of bias in measurement of the outcome	Risk of bias in selection of the reported result	Overall risk of bias
Sami et al., 2015	LOW	LOW	SOME CONCERNS*	LOW	LOW	LOW/SOME CONCERNS
Römmele et al., 2024	SOME CONCERNS	SOME CONCERNS	LOW	SOME CONCERNS	SOME CONCERNS	SOME CONCERNS
Wang et al., 2024	SOME CONCERNS	SOME CONCERNS	LOW	SOME CONCERNS	SOME CONCERNS	SOME CONCERNS
Luo et al., 2022	LOW	SOME CONCERNS	LOW	SOME CONCERNS	SOME CONCERNS	SOME CONCERNS
Li et al., 2022	LOW	SOME CONCERNS	LOW	HIGH	HIGH	HIGH
Jin et al., 2014	SOME CONCERNS	SOME CONCERNS	LOW	LOW	LOW	SOME CONCERNS

*Low risk only for the outcome of participation rates, which were based on all randomised patients. For endoscopic outcomes, only patients actually participated were analysed. There were substantial and differential drop-outs between groups, which could introduce bias.

3. COCHRANE RISK OF BIAS: Lower GI endoscopes and studies across different types of endoscope

Author (year)	Risk of bias arising from randomization process	Risk of bias due to deviations from intended interventions	Risk of bias from missing outcomes	Risk of bias in measurement of the outcome	Risk of bias in selection of the reported result	Overall risk of bias
Xu et al., 2024	LOW	SOME CONCERNS	LOW	LOW	HIGH	HIGH
Wei et al., 2022	HIGH	SOME CONCERNS	LOW	SOME CONCERNS	SOME CONCERNS	HIGH

4. QUADAS: Other upper GI endoscopes

Author (Year)	Risk of bias				Applicability concerns		
	Patient selection	Index test	Reference standard	Patient flow	Patient selection	Index test	Reference standard
Sami et al., 2019	UNCLEAR	LOW	LOW	LOW	LOW	LOW	LOW
Huynh et al., 2017	LOW	LOW	LOW	LOW	LOW	LOW	LOW
Kang et al., 2019	LOW	LOW	LOW	UNCLEAR	LOW	LOW	LOW
Sami et al., 2016	LOW	LOW	LOW	LOW	LOW	LOW	LOW
Halland et al., 2015	UNCLEAR	LOW	LOW	LOW	LOW	LOW	LOW
Aedo et al., 2014	LOW	LOW	LOW	LOW	LOW	LOW	LOW
Choi et al., 2014	UNCLEAR	LOW	UNCLEAR	UNCLEAR	UNCLEAR	LOW	LOW
Cho et al., 2013	UNCLEAR	LOW	LOW	UNCLEAR	LOW	LOW	LOW
Shariff et al.,.2016	LOW	LOW	LOW	LOW	LOW	LOW	LOW
Jin et al., 2017	LOW	UNCLEAR	UNCLEAR	LOW	LOW	LOW	LOW

5. ROBINS-I: Duodenoscopes

Author (year)	Confounding	Patient selection	Classification of interventions	Deviation from interventions	Missing data	Measurement of outcomes	Selection of reported result	Overall bias	Predicted direction of bias
Hutfless et al., 2022	MODERATE	LOW	LOW	LOW	LOW	LOW	LOW	LOW	UNPREDICTABLE
Straulino et al., 2019	MODERATE	LOW	LOW	LOW	LOW	LOW	LOW	MODERATE	UNPREDICTABLE
Gromski et al., 2021	MODERATE	LOW	LOW	LOW	LOW	LOW	LOW	MODERATE	UNPREDICTABLE
Gupta et al., 2021	MODERATE	LOW	LOW	LOW	LOW	LOW	LOW	MODERATE	UNPREDICTABLE
Liao et al., 2023	MODERATE	LOW	LOW	LOW	LOW	LOW	LOW	MODERATE	UNPREDICTABLE
Razzak et al., 2024	MODERATE	LOW	LOW	LOW	LOW	LOW	LOW	MODERATE	UNPREDICTABLE
Machado et al., 2024	MODERATE	LOW	LOW	LOW	LOW	LOW	LOW	MODERATE	UNPREDICTABLE
Barakat et al., 2024	MODERATE	LOW	LOW	LOW	LOW	LOW	LOW	MODERATE	UNPREDICTABLE
Lee et al., 2023	MODERATE	LOW	LOW	LOW	LOW	LOW	LOW	MODERATE	UNPREDICTABLE
Shiratori et al., 2024	MODERATE	LOW	LOW	LOW	LOW	LOW	LOW	LOW	UNPREDICTABLE
Pona et al., 2023	SERIOUS	LOW	LOW	LOW	LOW	LOW	LOW	SERIOUS	UNPREDICTABLE

Gambaccini et al., 2023	MODERATE	LOW	LOW	LOW	LOW	LOW	LOW	LOW	UNPREDICTABLE
Shahid et al., 2022	SERIOUS	LOW	LOW	LOW	LOW	LOW	LOW	SERIOUS	UNPREDICTABLE

6. ROBINS-I: Other upper GI endoscopes

**Author (year)**	**Confounding**	**Patient selection**	**Classification of interventions**	**Deviation from interventions**	**Missing data**	**Measurement of outcomes**	**Selection of reported result**	**Overall bias**	**Predicted direction of bias**
Jin et al., 2015	SERIOUS	LOW	LOW	LOW	LOW	LOW	LOW	SERIOUS	UNPREDICTABLE

7. NILHB: Duodenoscopes

Author (Year)	1. Is the review based on a focused question that is adequately formulated and described?	2. Were eligibility criteria for included and excluded studies predefined and specified?	3. Did the literature search strategy use a comprehensive, systematic approach?	4. Were titles, abstracts, and full-text articles dually and independently reviewed for inclusion and exclusion to minimize bias?	5. Was the quality of each included study rated independently by two or more reviewers using a standard method to appraise its internal validity?	6. Were the included studies listed along with important characteristics and results of each study?	7. Was publication bias assessed?	8. Was heterogeneity assessed? (Meta-analyses only.)	Quality Rating (Good, Fair, or Poor)
Kwakman et al., 2022	Yes	No	Yes	No	Not reported	Yes	Yes	N/A - Not meta-analysis	Fair

8. NILHB: Lower GI endoscopes and studies across different types of endoscope

Author (Year)	1. Is the review based on a focused question that is adequately formulated and described ?	2. Were eligibility criteria for included and excluded studies predefine d and specified?	3. Did the literature search strategy use a comprehensive , systematic approach?	4. Were titles, abstracts, and full-text articles dually and independentl y reviewed for inclusion and exclusion to minimize bias?	5. Was the quality of each included study rated independentl y by two or more reviewers using a standard method to appraise its internal validity?	6. Were the included studies listed along with important characteristic s and results of each study?	7. Was publicatio n bias assessed?	8. Was heterogeneit y assessed? (Meta-analyses only.)	Qualit y Rating (Good, Fair, or Poor)
Deb et al., 2022	Yes	Not reported	Yes	No	No	Yes	Yes	N/A - Not meta-analysis	Fair

9. CASE SERIES: Duodenoscopes

Author (Year)	1. Was the study question or objective clearly stated?	2. Was the study population clearly and fully described, including a case definition?	3. Were the cases consecutive?	4. Were the subjects comparable?	5. Was the intervention clearly described?	6. Were the outcome measures clearly defined, valid, reliable, and implemented consistently across all study participants?	7. Was the length of follow-up adequate?	8. Were the statistical methods well-described?	9. Were the results well-described?
Napoleon et al., 2022	YES	YES	YES	YES	YES	YES	YES	YES	YES
Slivka et al., 2021	YES	YES	YES	YES	YES	YES	YES	YES	YES
Muthusamy et al., 2020	YES	YES	YES	YES	YES	YES	YES	YES	YES
Persyn et al., 2022	YES	YES	YES	YES	YES	YES	YES	YES	YES
Venkatachalapathy et al., (2023)	YES	YES	CANNOT DETERMINE	YES	YES	CANNOT DETERMINE	YES	NO	CANNOT DETERMINE
Bruno et al. 2023	YES	YES	YES	YES	YES	YES	YES	YES	YES
Fugazza et al., 2024	YES	YES	YES	YES	YES	YES	YES	YES	YES

10. CASE SERIES: Other upper GI endoscopes

Author (Year)	1. Was the study question or objective clearly stated?	2. Was the study population clearly and fully described, including a case definition?	3. Were the cases consecutive?	4. Were the subjects comparable?	5. Was the intervention clearly described?	6. Were the outcome measures clearly defined, valid, reliable, and implemented consistently across all study participants?	7. Was the length of follow-up adequate?	8. Were the statistical methods well-described?	9. Were the results well-described?
Eqbal et al., 2021	YES	YES	CANNOT DETERMINE	YES	YES	YES	YES	YES	YES
Chung et al., 2012	YES	YES	CANNOT DETERMINE	YES	YES	YES	CANNOT DETERMINE	YES	YES
Peery et al., 2011	YES	CANNOT DETERMINE	CANNOT DETERMINE	CANNOT DETERMINE	YES	YES	CANNOT DETERMINE	YES	NO
Dowds et al., (2024)	YES	NO	CANNOT DETERMINE	YES	YES	YES	CANNOT DETERMINE	CANNOT DETERMINE	YES
Lee et al., (2024)	YES	YES	YES	YES	YES	YES	CANNOT DETERMINE	CANNOT DETERMINE	YES
VanDePloeg et al., 2023	YES	YES	CANNOT DETERMINE	YES	YES	YES	YES	YES	YES
Streckfuss et al., 2014	YES	NO	CANNOT DETERMINE	YES	YES	YES	CANNOT DETERMINE	NO	YES
