# Supplementary material for: Diagnostic and Clinical Performance of Single‐Use Versus Reusable Gastrointestinal Endoscopes: A Systematic Review and Meta‐Analyses
Source: J Gastroenterol Hepatol. 2026 Aug 7;41(9):2571–87. doi: 10.1111/jgh.70637 (PMC13534234; doi:10.1111/jgh.70637)
Supplement: Supplementary file 5 [file JGH-41-2571-s005.pdf]

Diagnostic and clinical performance of single-use versus reusable gastrointestinal endoscopes: a systematic review and meta-analyses

Supporting information Data S1: Deviations from review protocol

Authors

Natalie Tyldesley-Marshall, Yen-Fu Chen,* Jefferson Buendia, Anna Brown, Thomas Matthews, Janette Parr, Shivashri Chockalingam, Adel Elfeky, Amy Grove, Lazaros Andronis, Stuart R Coles, Mandana Zanganeh, Bu Hayee, Anjan Dhar, Shaji Sebastian, Ramesh Arasaradnam on behalf of the SUMU-Endo Project

*Correspondence to: Prof. Yen-Fu Chen

Centre for Evidence and Implementation Science, Park House, Edgbaston, University of Birmingham, Birmingham B15 2TT, United Kingdom

y.chen.25@bham.ac.uk

Department of Health and Welfare, College of City Management, University of Taipei, Taipei, Taiwan

yfchen@utaipei.edu.tw

Deviations from review protocol

Search strategy and selection criteria

Our initial scoping of the literature suggested that studies directly comparing SU or partially SU versus MU GI endoscopes for clinical infection risk were likely to be limited. We therefore intended to include studies of any designs that provided a quantitative estimate of infection risk following any GI endoscopy to enable indirect comparison between SU, partially SU and MU endoscopes in the absence of direct comparative studies. However, it became clear that such a strategy would result in a large amount of data for MU endoscopes very little data for SU endoscopes, with serious concerns of their comparability. We therefore focused on systematic reviews that provided an estimate of the risk of infection arising from transmission of exogenous pathogens by contaminated MU endoscopes, which is the risk that SU endoscopes aim to eliminate. Therefore, studies including solely MU GI endoscope data were included only if they provided an estimation of the risk of clinical infection associated with exogenous pathogens, or allowed this to be calculated.

Following advice from the Advisory Group, we restricted the included studies to be from 2010, to maintain relevance to current clinical practice.

Additional searches were undertaken to identify systematic reviews, and their reference lists examined. We retained those evaluating clinical infections associated with exogenous pathogens.

If no full-text was available, then multiple documents from one study were merged into one record in Covidence, and assessed as an abstract. Lee et al., (2023) was a trial registry record plus an abstract; Römmele et al., (2024) was a trial registry record plus an abstract; and Venkatachalapathy et al., (2024) was a trial registry record plus posted results (though required no merging of records in Covidence). However, the following studies with multiple documents were analysed as a full text, and data extracted from documents other than, and including, the full text. Bang et al., (2020) was a full text, plus an abstract reporting the same study. Barakat et al., (2024) was a full-text, plus an abstract, as the abstract contained more detailed results for a technical outcome. Van de Ploeg et al., (2023) was a full-text, plus a later published correction (identified in an updated search).

We excluded SU cholangioscopes such as *SpyGlass* (Olympus) as they were not seen as a substitute for similar MU counterparts, and were mainly used for specific clinical needs. One exception was Fugazza et al., (2024), which evaluated a fully SU duodenoscope (EXALT Model D) when used in combination with SpyGlass. SU robotic endoscopes were also excluded as they were primarily used only in research settings.

Data analysis

The protocol stated: “Data will be extracted by one reviewer and checked by another reviewer, with discrepancies resolved by discussions.” However, assisted by the functionality of Covidence, we had two reviewers extract data independently. Consensus was agreed, with the help of a third if necessary. The data extraction form did not include conflicts of interest, as stated in the protocol, because this field was omitted due to clerical error.

We planned to, but did not, assess the overall certainty of the evidence using the GRADE framework. Given the small number of studies associated with individual endoscope models and many weaknesses in study designs, most of the assessments would result in a designation of very low certainty for comparative evidence.

We were also unable to carry out planned subgroup analyses for clinical infection risk due to lack of data. As no direct comparative evidence for SU or partially SU compared with MU endoscopes with regard to clinical infection risk was found, we considered the possibility to carry out indirect comparisons as described in our protocol. We did not proceed with these comparisons because of the very heterogeneous sets of studies between SU, partially SU, and MU studies in terms of patient populations, methods of evaluating and reporting infection-related events, study periods (which have implications for endoscope designs, reprocessing methods and other concomitant interventions such as antibiotic prophylaxis); all of which could confound the results of indirect comparisons, and which would be impossible to adjust for. Proceeding with such comparisons would likely have produced unreliable estimates that could be misinterpreted and quoted.

The protocol stated: “If a meta-analysis is conducted, a random effects model will be used for primary analysis with a fixed effect model used as a sensitivity analysis.” We did not carry out sensitivity analyses using a fixed effect model as the results would be the same where no statistical heterogeneity was found, while the underlying assumption for the fixed effect model would be unlikely to hold where substantial heterogeneity was observed.

We appraised the systematic reviews with the National Heart, Lung and Blood Institute (NHLBI) tool as we found that the questions in AMSTAR 2, which was designed mainly for reviews of intervention effectiveness, was not a good fit for the included systematic reviews, which focused on infection risks.
