# Supplementary material for: Diagnostic and Clinical Performance of Single‐Use Versus Reusable Gastrointestinal Endoscopes: A Systematic Review and Meta‐Analyses
Source: J Gastroenterol Hepatol. 2026 Aug 7;41(9):2571–87. doi: 10.1111/jgh.70637 (PMC13534234; doi:10.1111/jgh.70637)
Supplement: Supplementary file 6 [file JGH-41-2571-s006.pdf]

Diagnostic and clinical performance of single-use versus reusable gastrointestinal endoscopes: a systematic review and meta-analyses

Supporting information Data S4: Additional evidence from included studies

Authors

Natalie Tyldesley-Marshall, Yen-Fu Chen,* Jefferson Buendia, Anna Brown, Thomas Matthews, Janette Parr, Shivashri Chockalingam, Adel Elfeky, Amy Grove, Lazaros Andronis, Stuart R Coles, Mandana Zanganeh, Bu Hayee, Anjan Dhar, Shaji Sebastian, Ramesh Arasaradnam on behalf of the SUMU-Endo Project

*Correspondence to: Prof. Yen-Fu Chen

Centre for Evidence and Implementation Science, Park House, Edgbaston, University of Birmingham, Birmingham B15 2TT, United Kingdom

y.chen.25@bham.ac.uk

Department of Health and Welfare, College of City Management, University of Taipei, Taipei, Taiwan

yfchen@utaipei.edu.tw

Additional evidence from included studies

1. Duodenoscopes

1.1 Technical performance

Supplementary Table 1. Comparative evidence on the number of attempts to cannulate / intubate

Study, publication type, study design, study location & case complexity (Bold = RCT)	Findings	P value
SU vs MU		
**Bang et al. 2021, RCT**<br>**USA**<br>**ASGE grade 3-4: 16% (SU) vs 14% (MU)**	EXALT Model D (Boston Scientific) vs TJF-180 (Olympus)<br>***Attempt to achieve ductal access***<br>Median (IQR)<br>2 (1-6) vs 5 (2-22)	0·013
Gambaccini et al. 2023, NRCS<br>Italy<br>ASGE grade 3-4: 100%	EXALT Model D (Boston Scientific) vs MU (unspecified)<br>***Number of attempts at ERCP cannulation***<br>Mean (SD)<br>13 (9) vs 11 (8)	0·683
SU vs SU		
Shahid et al. 2023, NRCS<br>Spain & USA<br>ASGE grade 3-4: 39% (EXALT Model D) vs 45% (aScope)	EXALT model D (Boston Scientific) vs aScope (Ambu)<br>***Number of ERCP cannulation attempts***<br>Mean (SD not reported)<br>1 vs 1	NR
SU vs MUDC vs MU		
Gromski et al. 2021, NRCS<br>USA<br>ASGE grade 3-4: not reported	EXALT Model D (Boston Scientific) vs TJF-190 (Olympus) vs TJF-160/TJF-180 (Olympus)<br>***Number of attempts to cannulate desired duct***<br>Mean (SD not reported)<br>2 vs 2 vs 3	NR

Abbreviations: ASGE: American Society for Gastrointestinal Endoscopy; MU – multiple-use; MUDC – multiple-use endoscopes with disposable # caps; NRCS – non-randomised comparative study; RCT - randomised controlled trial; SD: standard deviation; SU – single-use.

Yellow = Full-text article, White = Abstract

Supplementary Table 2. Comparative evidence on successful cannulation / intubation rate, difficult cannulation and need for advanced techniques

Study, publication type, study design, study location & case complexity (Bold = RCT)	Findings	P value
SU vs MU		
**Bang et al. 2021, RCT**<br>**USA**<br>**ASGE grade 3-4: 16% (SU) vs 14% (MU)**	EXALT Model D (Boston Scientific) vs TJF-180 (Olympus)<br>***Cannulation of desired duct***<br>96% (46/48) vs 100% (50/50)<br><br>***Need for advanced techniques***<br>15% (7/48) vs 22% (11/50)	0·237*<br><br>0·343†
Gambaccini et al. 2023, NRCS<br>Italy<br>ASGE grade 3-4: 100%	EXALT Model D (Boston Scientific) vs MU (unspecified)<br>***ERCP cannulation success rate***<br>100% (25/25) vs 93% (140/150)<br>***Need for advanced techniques***<br>16% (4/25) vs 13% (20/150)	0·156

		0·720†
Razzak et al. 2023, NRCS<br>USA<br>ASGE grade 3-4: 17% (SU) vs 23% (MU)	EXALT Model D (Boston Scientific) vs TJF-180 (Olympus)<br>***Difficult cannulation***<br>21% ‡ (11/53) vs 14% (11/80)	0·29
SU vs MUDC		
Machado et al. 2023, NRCS<br>USA<br>ASGE grade 3-4: 62%§	EXALT Model D (Boston Scientific)(n=52) vs TJF-Q190V (Olympus)(n=104)<br>***Oesophageal intubation***<br>98% (51/52) vs 100% (104/104)<br><br>***Duodenal intubation***<br>94% (49/52) vs 100% (104/104)<br><br>***Biliary cannulation***<br>87% (45/52) vs 100% (104/104)	0·333*<br><br>0·036*<br><br><0·001*
MUDC vs MU		
**Forbes et al. 2023, RCT**<br>**Canada**<br>**ASGE grade 3-4: 24% (MUDC) vs 24% (MU)**	ED34-i10T2 (Pentax) vs ED34-i10T (Pentax)<br>***Cannulation of common bile duct (where targeted)***<br>96% (231/241) vs 94% (219/234)	0·09
Barakat et al. 2024, NRCS<br>USA<br>ASGE grade 3-4: not reported	Olympus Medical Systems Evis Exera III / TJF-Q190V (Olympus) vs TJF-Q180V (Olympus)<br>***Need for advanced techniques (for index¶ ERCPs)***<br>8/27 (30%) vs<br>1/35 (3%)(P=0·01).	0·01

Abbreviations: ASGE: American Society for Gastrointestinal Endoscopy; MU – multiple-use; MUDC – multiple-use endoscopes with disposable caps; NR – not reported; NRCS – non-randomised comparative study; RCT - randomised controlled trial; SU – single-use;

Yellow = Full-text article, White = Abstract

* p-value was not reported and was calculated by review authors (Fisher's exact test).

† p-value was not reported and was calculated by review authors (Pearson Chi-squared test).

‡ Percentages were rounded to the nearest integers, due to the low numbers of participants in the included studies. The exception was made in the case of those where rounding to the nearest integer would lead to 0% despite n being above 0, which would appear incorrect. Exceptions were also made for studies with thousands of patients; p values; confidence intervals; infection risk estimates; and diagnostic accuracy outcomes. This is consistent throughout the appendix.

§ For all study participants (i.e. % not separately reported for SU and MU; the number shown was for patients with ASGE grade 3; other grades were not reported.¶Index ERCPs refers to "first-time ERCPs performed on native ampullas for which cannulation is typically most challenging".

Supplementary Table 3. Proportion of cross-over to MU endoscopes for SU endoscopes and MUDC and success rates following cross-over

Study, publication type, study design, study location & case complexity (Bold = RCT)	Findings
**SU**	
**Bang et al. 2021, RCT**<br>**USA**<br>**ASGE grade 3-4 (for SU): 16%**	EXALT Model D (Boston Scientific)<br>***Proportion crossed over to MU***<br>4% (2/48)<br><br>***Success rate following cross-over***<br>0% (0/2)
Machado et al. 2023, NRCS<br>USA<br>ASGE grade 3-4: 62%*	EXALT Model D (Boston Scientific)<br>***Proportion crossed over to MU***<br>13% (7/52)<br><br>***Success rate following cross-over***<br>71% (5/7)

Gambaccini et al. 2023, NRCS<br>Italy<br>ASGE grade 3-4: 100%	EXALT Model D (Boston Scientific)<br>***Proportion crossed over to MU***<br>0% (0/25)<br><br>***Success rate following cross-over***<br>Not applicable
Razzak et al. 2023, NRCS<br>USA<br>ASGE grade 3-4 (for SU): 17%	EXALT Model D (Boston Scientific)<br>***Proportion crossed over to MU***<br>6% (3/53)<br><br>***Success rate following cross-over***<br>67% (2/3)
Shahid et al. 2023, NRCS<br>Spain & USA<br>ASGE grade 3-4: EXALT Model D 39%, aScope 45%	EXALT Model D (Boston Scientific)<br>***Proportion crossed over to MU***<br>11% (14/129)<br><br>***Success rate following cross-over***<br>93% (13/14)<br><br>aScope (Ambu)<br>***Proportion crossed over to MU***<br>22% (16/72)<br><br>***Success rate following cross-over***<br>100% (16/16)
Gromski et al. 2021, NRCS<br>USA<br>ASGE grade 3-4: not reported	EXALT Model D (Boston Scientific)<br>***Proportion crossed over to MU***<br>8% (4/51)<br><br>***Success rate following cross-over***<br>100% (4/4)
Gupta et al. 2021, NRCS<br>USA<br>ASGE grade 3 (for SU): 48% (no participants were grade 4 cases)	EXALT Model D (Boston Scientific)<br>***Proportion crossed over to MU***<br>0% (0/25)<br><br>***Success rate following cross-over***<br>Not applicable
Fugazza et al. 2024, case series<br>Belgium, India, Italy, Saudi Arabia, USA<br>ASGE grade 3-4: 100%	Model D and SpyGlass DS II (Boston Scientific) (SU cholangioscope)<br>***Proportion crossed over to MU***<br>2% (1/66)<br><br>***Success rate following cross-over***<br>100% (1/1)
Venkatachalapathy, 2023, case series<br>Republic of Ireland<br>ASGE grade 3-4: excluded grade 4, but details not reported	EXALT Model D (Boston Scientific)<br>***Proportion crossed over to MU***<br>7% (4/59)<br><br>***Success rate following cross-over***<br>75% (3/4)
Bruno et al. 2023, case series<br>Australia, Canada, China, France, Germany, Hong Kong, India, Italy, Netherlands, Singapore, South Africa, USA<br>ASGE grade 3-4: 36%	EXALT Model D (Boston Scientific)<br>***Proportion crossed over to MU***<br>7% (37/551)<br><br>***Success rate following cross-over***<br>70% (26/37)
Napoléon et al. 2022, case series<br>France<br>ASGE grade 3-4: 40%	EXALT Model D (Boston Scientific)<br>***Proportion crossed over to MU***<br>5% (3/60)<br><br>***Success rate following cross-over***<br>0% (0/3)
Persyn et al. 2022, case series<br>Belgium<br>ASGE grade 3-4: 52%	EXALT Model D (Boston Scientific)<br>***Proportion crossed over to MU***<br>8% (4/52)<br><br>***Success rate following cross-over***<br>50% (2/4)
Slivka et al. 2021, case series<br>Netherlands & USA	EXALT Model D (Boston Scientific)<br>***Proportion crossed over to MU***

ASGE grade 3-4: 41%	10% (19/200)<br><br>***Success rate following cross-over***<br>84% (16/19)
Muthusamy et al. 2020, case series<br>USA<br>ASGE grade 3-4: 45%	EXALT Model D (Boston Scientific)<br>***Proportion crossed over to MU***<br>3% (2/60)<br><br>***Success rate following cross-over***<br>100% (2/2)
**MU-SUDC**	
Barakat et al. 2024, NRCS<br>USA<br>ASGE grade 3-4: not reported	Olympus Medical Systems Evis Exera III /Olympus TJF-Q190V<br>***Proportion crossed over to MU***<br>20% (9/46)<br><br>***Success rate following cross-over***<br>67% (6/9)
Gromski et al. 2021, NRCS<br>USA<br>ASGE grade: not reported	TJF-190 (Olympus)<br>***Proportion crossed over to MU***<br>0% (0/49)<br><br>***Success rate following cross-over***<br>Not applicable

Abbreviations: ASGE: American Society for Gastrointestinal Endoscopy; MU – multiple-use; MUDC – multiple-use endoscopes with disposable cap; NRCS – non-randomised comparative study; RCT - randomised controlled trial; SU – single-use;

Yellow = Full-text article, White = Abstract

*For all study participants (i.e. % not separately reported for SU and MU; the number shown was for patients with ASGE grade 3; other grades were not reported.

Supplementary Table 4. Comparative evidence on ERCP successful procedure completion (excluding successful cross-over)

Study, publication type, study design, study location & case complexity (Bold = RCT)	Findings	P value
**SU vs MU**		
Gambaccini et al. 2023, NRCS<br>Italy<br>ASGE grade 3-4: 100%	EXALT Model D (Boston Scientific) vs MU (unspecified)<br>***Successful ERCP***<br>100% (25/25) vs 93% (140/150)	0·089
Razzak et al. 2023, NRCS<br>USA<br>ASGE grade 3-4: 17% (SU) vs 23% (MU)	EXALT Model D (Boston Scientific) vs TJF-180 (Olympus)<br>***Successful ERCP***<br>88% (47/53) vs 94% (75/80)	0·030
**SU vs MUDC**		
Machado et al. 2023, NRCS<br>USA<br>ASGE grade 3-4: 62%*	EXALT Model D (Boston Scientific) vs TJF-Q190V (Olympus)<br>***Successful ERCP***<br>87% (45/52) vs 100% (104/104)	<0·001†
**SU vs SU**		
Shahid et al. 2023, NRCS<br>Spain & USA<br>ASGE grade 3-4: 39% (EXALT Model D) vs 45% (aScope)	EXALT model D (Boston Scientific) vs aScope (Ambu)<br>***Successful ERCP***<br>92% (119/129) vs 92% (66/72)	0·884‡
Liao et al. 2023, NRCS<br>USA<br>ASGE grade 3-4: 36% (EXALT Model D) vs 72% (aScope)	EXALT Model D (Boston Scientific) vs aScope (Ambu)<br>***Successful ERCP***<br>98% (55/56) vs 100% (53/53)	1·000†
**SU vs MUDC vs MU**		
Gromski et al. 2021, NRCS<br>USA<br>ASGE grade 3-4: not reported	EXALT Model D (Boston Scientific) vs TJF-190 (Olympus) vs TJF-160/180<br>***Success ERCP***<br>92% (47/51) vs 100% (49/49) vs 100% (514/514)	<0·001†
**MUDC vs MU**		
**Forbes et al. 2023, RCT**<br>**Canada**<br>**ASGE grade 3-4: 24% (MUDC) vs 24% (MU)**	ED34-i10T2 (Pentax) vs ED34-i10T (Pentax)<br>***Successful ERCP***<br>95% (245/259) vs 91% (235/259)	0·13
Lee et al. 2023, NRCS<br>South Korea<br>ASGE grade 3-4: not reported	MU duodenoscopes with SU end-caps (unspecified) vs MU (unspecified)<br>Successful ERCP<br>95% (96/101) vs 85% (81/95)	0·15

Abbreviations: ASGE: American Society for Gastrointestinal Endoscopy; MU – multiple-use; MUDC – multiple-use endoscopes with disposable cap; NRCS – non-randomised comparative study; RCT - randomised controlled trial; SU – single-use;

Yellow = Full-text article, White = Abstract

*For all study participants (i.e. % not separately reported for SU and MU; the number shown was for patients with ASGE grade 3; other grades were not reported.

†p-value was not reported and was calculated by review authors (Fisher's exact test).

‡p-value was not reported and was calculated by review authors (Pearson Chi-squared test).

Supplementary Table 5. Technical outcomes – Defect rate: Duodenoscopes

Author, year, country, publication	Description of population, centre,	Description of multiple-use (MU)	SU group vs MU group -

type [a], dates of procedures, trial registration	single-use (SU) endoscope, multiple-use (MU) endoscope with disposable cap (MUDC) / average age (years), % female, ASGE complexity, indications	endoscope / SU elevator cap	Defect / device failure rate
**SU duodenoscopes: EXALT Model D (Boston Scientific)**			
Bang et al., 2021 (FT)<br><br>USA<br><br>Dates of procedures: 01/2020 to 03/2020<br><br>Trial registration: NCT04143698	Patients with native papilla requiring ERCP at centre for interventional endoscopy.<br>Model D (n=48) vs TJF-180 (n=50)<br>Mean age (SD): 67 (14) vs 61 (18)<br>Female: 46% vs 54%<br>ASGE complexity<br>Grade 1: 19% vs 24%<br>Grade 2: 65% vs 62%<br>Grade 3: 8% vs 4%<br>Grade 4: 8% vs 10%.<br>Main indications:<br>Biliary: 92% vs 86%,<br>Pancreatic: 6% vs 12%.	TJF-180 (Olympus),<br>MU duodenoscope (n=50)	N/A
Machado et al., 2024 (AB)<br><br>USA<br><br>Dates of procedures: 06/2020 to 12/2022<br><br>Trial registration: NR	Adult patients requiring ERCP at a tertiary care cancer centre.<br>Exalt Model D (n=52) vs TJF-Q190V (Olympus) (n=104)<br>Mean age (IQR): 64 (54-72)<br>Female: 40%<br>ASGE complexity<br>Grade 3: 62%<br>Prior ERCP: 63% vs 31%.		N/A
Gambaccini et al., 2023 (AB)<br><br>Italy<br><br>Dates of procedures: 07/2020 to 07/2022<br><br>Trial registration: NR	Liver transplanted patients with positivity to Klebsiella Pneumoniae Carbapenemase infection undergoing complex ERCP in Endoscopy Unit.<br>Exalt Model D (n=25) vs MU (n=150).<br>Age: NR<br>Female: NR<br>ASGE complexity (n=25)<br>Grade 3: 68%<br>Grade 4: 32%<br>Main indications:<br>Clinic-laboratory alterations, common bile duct stones, biliary leaks, and strictures (% NR).	MU duodenoscope (details NR)<br>(n=150)	N/A
Liao et al., 2023 (AB)<br><br>USA<br><br>Dates of procedures: 01/2021 to 04/2023<br><br>Trial registration: NR	Consecutive adult patients undergoing ERCP at a tertiary centre<br>Exalt Model D (n=56) vs aScope (Ambu) (n=53)<br>Mean age: 58 vs 55<br>Female: 49% vs 60%<br>ASGE complexity<br>Grade 1: 27% vs 15%<br>Grade 2: 38% vs 13%<br>Grade 3: 34% vs 57%<br>Grade 4: 2% vs 15%.<br>Main indications:<br>Biliary stricture: 15%,<br>Pancreatis: 15%,<br>Bile stone: 14%.	N/A	N/A
Razzak et al., 2023 (AB)<br><br>USA	All adult patients undergoing ERCP at a tertiary medical centre.	TJF-180 (Olympus),<br>MU duodenoscope (n=80)	N/A

Dates of procedures: 01/2020 to 02/2023<br><br>Trial registration: NR	Exalt Model D (n=53) vs TJF-180 (n=80) during the first week of each month in 2022.<br>Mean age: 70 (± 12) vs 69 (± 14)<br>Female: 43% vs 49%<br>ASGE complexity<br>Grades 3-4: 17% vs 23%<br>Main indications:<br>Diabetes: 30% vs 20%.		
Shahid et al., 2023 (FT)<br><br>Spain and USA<br><br>Dates of procedures: 06/2020 to 11/2021<br><br>Trial registration: NCT05041608	Patients undergoing ERCP at nine academic centres.<br>Exalt Model D (n=129) vs aScope (Ambu) (n=72).<br>Mean age: 63 vs 65<br>Female: 49% vs 58%<br>ASGE complexity<br>Grade 1: 19% vs 15%<br>Grade 2: 42% vs 40%<br>Grade 3: 27% vs 42%<br>Grade 4: 12% vs 3%.<br>Main indications:<br>Biliary: 73% vs 78%,<br>Pancreatic: 14% vs 15%,<br>Other: 13% vs 7%.	N/A	Scope malfunction:<br>Model D: 3 (3%). aScope: 2 (3%).
Gromski et al., 2021 (AB)<br><br>USA<br><br>Dates of procedures: 01/2020 to 04/2020<br><br>Trial registration: NR	Patient undergoing ERCPs at one institution. Model D was used for high-risk criteria, based on an internal algorithm.<br>Exalt Model D (n=51) vs TJF-190 (Olympus) (MUDC) (n=49) vs TJF-160/180 (n=514).<br>Median age (IQR): 61 (17) vs 57 (18) vs 60 (2).<br>Female: 43% vs 41% vs 54%<br>ASGE complexity: NR<br>Main indications:<br>Biliary: 90% vs 82% vs 79%<br>Pancreatic: 10% vs 18% vs 21%.	TJF-160/TJF-180 (Olympus), MU duodenoscope (n=514)	N/A
Gupta et al., 2021 (AB)<br><br>USA<br><br>Dates of procedures: 09/2020 to 11/2020<br><br>Trial registration: NR	Consecutive adult patients with native biliary anatomy undergoing ERCP for any indication in one institution.<br>Exalt Model D (n=25) vs TJF-180 (n=25).<br>Age: NR<br>Female: NR<br>ASGE complexity<br>Grade 1: 48% vs 24%<br>Grade 2: 4% vs 8%<br>Grade 3: 48% vs 68%<br>Main indications: NR	TJF-180 (Olympus),<br>MU duodenoscope (n=25)	N/A
Fugazza et al., 2024 (FT)<br><br>Belgium, India, Italy, Saudi Arabia, USA<br><br>Dates of procedures: 05/2020 to 09/2021<br><br>Trial registration: NR	Consecutive adult patients with clinical indications for cholangioscopy / pancreatoscopy in 10 tertiary centres.<br>Exalt Model D and SpyGlass DS II (Boston Scientific)(SU cholangioscope) (N=66).<br>Mean age (SD): 67 (± 16)<br>Female: 39%<br>ASGE complexity<br>Grade 3: 71%<br>Grade 4: 29%<br>Main indications:<br>Indeterminate biliary stricture: 46%,<br>Complex biliary stone removal: 42%.	N/A	N/A

Venkatachalapathy 2023 (AB)<br><br>Republic of Ireland and UK<br><br>Dates of procedures: 03/2021 to 08/2023<br><br>Trial registration: NCT04671095	Consecutive patients undergoing ERCP at four hospitals.<br>Exalt Model D (N=59).<br>Median age: 64<br>Female: 53%<br>ASGE complexity:<br>Grade 1 – 3: 100%.<br>Main indications:<br>Common bile duct stones: 56%,<br>Bile duct strictures: 44%.	N/A	N/A
Bruno et al., 2023 (FT)<br><br>Australia, Canada, China, France, Germany, Hong Kong, India, Italy, Netherlands, Singapore, South Africa, USA<br><br>Dates of procedures: 03/2021 to 11/2022<br><br>Trial registration: NCT04103749	Consecutive adult patients undergoing ERCP at 22 academic centres.<br>Exalt Model D (N=551).<br>Mean age: 60<br>Female: 60%<br>ASGE complexity<br>Grade 1: 10%<br>Grade 2: 52%<br>Grade 3: 29%<br>Grade 4: 7%<br>None: 3%.<br>Main indications:<br>Bile duct stones/gallstones (current): 31%,<br>Biliary or pancreatic stricture, unresolved: 23%,<br>Chronic pancreatitis: 12%,<br>Pancreatic tumour: 11%.	N/A	N/A
Napoléon et al., 2022 (FT)<br><br>France<br><br>Dates of procedures: 09/2020 to 09/2020<br><br>Trial registration: 2020-A00346-33	Consecutive adult patients undergoing ERCP in six centres.<br>Exalt Model D (N=60).<br>Median age: 66<br>Female: 57%<br>ASGE complexity<br>Grade 1: 2%<br>Grade 2: 58%<br>Grade 3: 35%<br>Grade 4: 5%.<br>Main indications:<br>Common bile duct stone: 42%,<br>Malignant biliary obstruction: 27%,<br>Chronic pancreatitis/ pancreatic duct intervention: 20%	N/A	2 (3%)
Persyn et al., 2022 (FT)<br><br>Belgium<br><br>Dates of procedures: 10/2020 to 11/2020<br><br>Trial registration: NR	Patients scheduled for ERCP at a single tertiary referral centre.<br>Exalt Model D (N=52).<br>Mean age: 65 (9-98)<br>Female: 48%<br>ASGE complexity<br>Grade 1: 0%<br>Grade 2: 48%<br>Grade 3: 33%<br>Grade 4: 19%.<br>Main indications:<br>Common bile duct stones 29%,<br>Benign extrahepatic stricture 14%,<br>Benign intrahepatic stricture 12%.	N/A	N/A
Slivka et al., 2021 (FT)<br><br>Netherlands and USA<br><br>Dates of procedures: 01/2020 to 02/2021	Adult patients undergoing ERCP at seven healthcare centres.<br>Exalt Model D (N=200).<br>Mean age: 63<br>Female: 60%<br>ASGE complexity	N/A	N/A

Trial registration: NCT04223830	Grade 1: 10%<br>Grade 2: 47%<br>Grade 3: 30%<br>Grade 4: 11%<br>None: 3%.<br>Main indications:<br>Previous ERCPS: 65%,<br>Prior biliary or pancreatic sphincterotomy: 53%,<br>Prior stent placement: 44%.		
Muthusamy et al., 2020 (FT)<br><br>USA<br><br>Dates of procedures: 04/2019 to 05/2019<br><br>Trial registration: NCT03701958	Consecutive adult patients undergoing ERCP at six academic medical centres.<br>Exalt Model D (N=73).<br>60 ERCPs; 13 roll-in manoeuvres only.<br>Mean age: 64<br>Female: 31%<br>ASGE complexity (n=60)<br>Grade 1: 12%<br>Grade 2: 43%<br>Grade 3: 43%<br>Grade 4: 2%.<br>Main indications:<br>Placement of pancreatic stents: 44%,<br>Removal of pancreatic stents: 43%,<br>Evaluation of a biliary stricture/filling defect: 28%.	N/A	N/A
**Other SU duodenoscopes**			
Shiratori et al., 2024 (AB)<br><br>USA<br><br>Dates of procedures: 07/2020 to 09/2023<br><br>Trial registration: NR	Data from Registry-Medicare database for patients undergoing ERCP in the United States.<br>SU (details NR) (n=3,143) vs MU (n=132,577).<br>Age: NR<br>Female: NR<br>ASGE complexity: NR<br>Main indications: NR	MU duodenoscope (details NR)<br>(n=132,577)	N/A
Pona et al., 2023 (AB)<br><br>USA<br><br>Dates of procedures: 09/2022 to 10/2022<br><br>Trial registration: NR	Patients undergoing procedures that involved a side-viewing duodenoscope where patient turnover time [c] was not impacted by patient or service-related issues at one tertiary centre.<br>SU (details NR) (n=21) vs MU (n=37).<br>Age: NR<br>Female: NR<br>ASGE complexity: NR<br>Main indications: NR	MU duodenoscope (details NR)<br>(n=37)	N/A
Hutfless et al., 2022 (FT)<br><br>USA<br><br>Dates of procedures: 01/2015 to 12/2021<br><br>Trial registration: NR	Medicare Fee-For-Service claims (2015–2021) for patients undergoing ERCP at hospitals and Ambulatory Service Centres.<br>SU (details NR) (n=711) vs MU (n=822,864)<br>Age: NR<br>Female: 53%.<br>ASGE complexity: NR<br>Main indications:<br>Pancreatitis or biliary condition: 81%.	MU duodenoscope (details NR)<br>(n=822,864)	N/A
**MU duodenoscope with SU elevator / end-cap**			
Forbes et al., 2023 (FT)<br><br>Canada	Patients undergoing ERCP for any indication at two tertiary ERCP centres.		N/A

Dates of procedures: 12/2019 to 02/2022<br><br>Trial registration: NCT04040504	ED34-i10T2 (Pentax) (MUDC) (n=259) vs ED34-i10T (n=259).<br>Mean age (SD): 60 (17) vs 61 (17)<br>Female: 54% vs 46%<br>ASGE complexity<br>Grade 1: 6% vs 6%<br>Grade 2: 70% vs 70%<br>Grade 3: 22% vs 22%<br>Grade 4: 2% vs 2%.<br>Main indications:<br>Suspected or confirmed biliary stones: 44% vs 38%,<br>Repeat procedure including stent removal or exchange 20% vs 22%,<br>Suspected or confirmed malignant biliary obstruction 9% vs 12%.	ED34-i10T (Pentax Medical), MU duodenoscope (n=259)	
Barakat et al., 2024 (FT)<br><br>USA<br><br>Dates of procedures: 2021 to 2022 (18-month period)<br><br>Trial registration: NR	Paediatric patients (1-18 years) undergoing ERCP at two centres.<br>Evis Exera III / TJF-Q190V (Olympus) (MUDC) (n=46) vs TJF-Q180V (n=60).<br>Mean age (SD): 14 (± 4)<br>Female: NR<br>ASGE complexity: NR<br>Main indications:<br>Biliary indication: 77%,<br>Pancreatic indication: 23%	TJF-Q180V (Olympus), MU duodenoscope (n=60)	N/A
Lee et al., 2023 (AB)<br><br>South Korea<br><br>Dates of procedures: NR<br><br>Trial registration: NR	Patients undergoing ERCP at one centre.<br>MUDC (details NR)(n=101) vs MU (n=95).<br>Age: NR<br>Female: NR<br>ASGE complexity: NR<br>Main indications:<br>Naïve papilla: 34% vs 51% (P=0·04).	MU duodenoscope (details NR)<br>(n=95)	N/A
Straulino et al., 2019 (AB)<br><br>Germany<br><br>Dates of procedures: NR<br><br>Trial registration: NR	Patients undergoing ERCP at one centre.<br>ED34-i10T2 (Pentax)(MUDC) (n=34) vs MU (n=37).<br>Age: NR<br>Female: NR<br>ASGE complexity: NR<br>Main indications: NR	MU duodenoscope (details NR)<br>(n=37)	N/A
**MU endoscopes only**			
Kwakman et al., 2022 (FT)<br><br>The Netherlands<br><br>Dates of publications: 01/2008 to 12/2019<br><br>Trial registration: N/A	Patients undergoing ERCPs in The Netherlands. (Outbreaks in three University Medical Centres.)<br>Age: NR<br>Female: NR<br>ASGE complexity: NR<br>Main indications: NR<br><br>N=3 studies	Olympus TJF-180V MU duodenoscope for 2 outbreaks,<br>Olympus (model unknown) MU duodenoscope for 1 outbreak.	N/A

[a] AB: conference abstract; FT – full-text article.

AB: conference abstract; FT: full-text article; MU: multiple-use (endoscope); MUDC: multiple-use endoscope with disposable cap; N/A: Not applicable; NR: not reported; PR: posted results; SD: standard deviation; SU: single-use (endoscope); TR: trial registry record.

Supplementary Table 6. Technical outcomes – Image quality: Duodenoscopes

Author, year, country, publication type [a], dates of procedures, trial registration	SU group vs MU group - Image quality	SU group vs MU group - Successful visualisation
**SU duodenoscopes: EXALT Model D (Boston Scientific)**		
Bang et al., 2021 (FT)<br><br>USA<br><br>Dates of procedures: 01/2020 to 03/2020<br><br>Trial registration: NCT04143698	1 Easiest – 5 Most difficult<br>Mean (SD):<br><br>Image quality:<br>2 (1) vs 1 (0) (p<0·001).	N/A
Machado et al., 2024 (AB)<br><br>USA<br><br>Dates of procedures: 06/2020 to 12/2022<br><br>Trial registration: NR	N/A	N/A
Gambaccini et al., 2023 (AB)<br><br>Italy<br><br>Dates of procedures: 07/2020 to 07/2022<br><br>Trial registration: NR	0 Easiest – 10 Most difficult<br>Median (Range):<br><br>Endoscopic image quality: 10 (8-10).<br><br>Radiologic image quality: 7 (3-10).	N/A
Liao et al., 2023 (AB)<br><br>USA<br><br>Dates of procedures: 01/2021 to 04/2023<br><br>Trial registration: NR	N/A	N/A
Razzak et al., 2023 (AB)<br><br>USA<br><br>Dates of procedures: 01/2020 to 02/2023<br><br>Trial registration: NR	N/A	N/A
Shahid et al., 2023 (FT)<br><br>Spain and USA	1 High – 5 Low<br>Median:<br><br>Location and visualisation quality:<br>Model D: 2,	N/A

Dates of procedures:<br>06/2020 to 11/2021<br><br>Trial registration:<br>NCT05041608	aScope: 3.<br><br>Model D:<br>Majority said good visualisation,<br>aScope:<br>Majority said good visualisation.	
Gromski et al., 2021 (AB)<br><br>USA<br><br>Dates of procedures:<br>01/2020 to 04/2020<br><br>Trial registration:<br>NR	1 Best – 5 Worst<br>Mean:<br><br>Image quality (SU vs MUDC vs MU):<br>4 vs 2 vs 3 ($p<0{\cdot}001$).	NR
Gupta et al., 2021 (AB)<br><br>USA<br><br>Dates of procedures:<br>09/2020 to 11/2020<br><br>Trial registration:<br>NR	N/A	N/A
Fugazza et al., 2024 (FT)<br><br>Belgium, India, Italy, Saudi Arabia, USA<br><br>Dates of procedures:<br>05/2020 to 09/2021<br><br>Trial registration:<br>NR	N/A	N/A
Venkatachalapathy 2023 (AB)<br><br>Republic of Ireland and UK<br><br>Dates of procedures:<br>03/2021 to 08/2023<br><br>Trial registration:<br>NCT04671095	Image quality:<br>NR	N/A
Bruno et al., 2023 (FT)<br><br>Australia, Canada, China, France, Germany, Hong Kong, India, Italy, Netherlands, Singapore, South Africa, USA<br><br>Dates of procedures:<br>03/2021 to 11/2022<br><br>Trial registration:<br>NCT04103749	N/A	N/A

Napoléon et al., 2022 (FT)<br><br>France<br><br>Dates of procedures: 09/2020 to 09/2020<br><br>Trial registration: 2020-A00346-33	Percentage rated clinically satisfactory<br><br>Image quality and brightness: 60 (100%)<br>Visualization of landmark on screen: 60 (100%)<br><br>Percentage rated comparable to MU duodenoscope<br><br>Image quality and brightness: 55 (92%)<br>Visualization of landmark on screen: 60 (100%).	N/A
Persyn et al., 2022 (FT)<br><br>Belgium<br><br>Dates of procedures: 10/2020 to 11/2020<br><br>Trial registration: NR	1 Not preferred – 5 Comparable to MU duodenoscope<br>Median:<br><br>Visualisation of important landmarks on monitor: 5<br>Image quality / appearance/ brightness: 5.	N/A
Slivka et al., 2021 (FT)<br><br>Netherlands and USA<br><br>Dates of procedures: 01/2020 to 02/2021<br><br>Trial registration: NCT04223830	1 Not preferred – 5 Comparable to MU duodenoscope<br>Median (range):<br><br>Visualisation of important landmarks on monitor: 4 (1-5)<br>Image quality / appearance/ brightness: 4 (1-5).	N/A
Muthusamy et al., 2020 (FT)<br><br>USA<br><br>Dates of procedures: 04/2019 to 05/2019<br><br>Trial registration: NCT03701958	1 Not preferred – 5 Comparable to MU duodenoscope<br>Median (range):<br><br>Visualisation of important landmarks on monitor: 5 (1-5)<br>Image quality / appearance/ brightness: 4 (1-5).	N/A
**Other SU duodenoscopes**		
Shiratori et al., 2024 (AB)<br><br>USA<br><br>Dates of procedures: 07/2020 to 09/2023<br><br>Trial registration: NR	N/A	N/A
Pona et al., 2023 (AB)<br><br>USA<br><br>Dates of procedures: 09/2022 to 10/2022<br><br>Trial registration: NR	N/A	N/A

Hutfless et al., 2022 (FT)<br><br>USA<br><br>Dates of procedures: 01/2015 to 12/2021<br><br>Trial registration: NR	N/A	N/A
USA<br><br>01/2017 to 12/2017<br><br>Trial registration: NR	N/A	N/A
**MU duodenoscope with SU elevator / end-cap**		
Forbes et al., 2023 (FT)<br><br>Canada<br><br>Dates of procedures: 12/2019 to 02/2022<br><br>Trial registration: NCT04040504	N/A	N/A
Barakat et al., 2024 (FT)<br><br>USA<br><br>Dates of procedures: 2021 to 2022 (18-month period)<br><br>Trial registration: NR	N/A	N/A
Lee et al., 2023 (AB)<br><br>South Korea<br><br>Dates of procedures: NR<br><br>Trial registration: NR	N/A	N/A
Straulino et al., 2019 (AB)<br><br>Germany<br><br>Dates of procedures: NR<br><br>Trial registration: NR	N/A	N/A
**MU endoscopes only**		
Kwakman et al., 2022 (FT)<br><br>The Netherlands	N/A	N/A

Dates of publications: 01/2008 to 12/2019<br><br>Trial registration: N/A	

[a] AB: conference abstract; FT – full-text article.

AB: conference abstract; FT: full-text article; IQR: interquartile range; MU: multiple-use (endoscope); MUDC – multiple-use endoscopes with disposable cap; N/A: Not applicable; NR: not reported; PR: posted results; SD: standard deviation; SU: single-use (endoscope); TRR: trial registry record.

Supplementary Table 7. Technical outcomes – Operator rating: Duodenoscopes

Author, year, country, publication type [a], dates of procedures, trial registration	SU group vs MU group - Manoeuvres successful	SU group vs MU group - Operator rating: manoeuvres	SU group vs MU group - Operator rating: performance characteristics	SU group vs MU group - Operator rating: overall satisfaction
**SU duodenoscopes: EXALT Model D (Boston Scientific)**				
Bang et al., 2021 (FT)<br><br>USA<br><br>01/2020 to 03/2020<br><br>Trial registration: NCT04143698	N/A	1 Easiest – 5 Most difficult<br>Mean (SD):<br><br>Overall ability to perform biliary interventions:<br>1 (0.5) vs 1 (0.5) (p<0·427). (Further detail in Supp. Table 3)<br><br>Overall ability to perform pancreatic/ other interventions:<br>1 (0.5) vs 1 (0.5) (p<0·427). (Further detail in Supp. Table 4)	1 Easiest – 5 Most difficult<br>Mean (SD):<br><br>Overall manoeuvrability:<br>1 (1) vs 1 (0.5) (p<0·065). (Further detail in Supp. Table 1)<br><br>Overall mechanical and imaging characteristics:<br>2 (1) vs 1 (0·5) (p<0·001). (Further detail in Supp. Table 2)	N/A
Machado et al., 2024 (AB)<br><br>USA<br><br>Dates of procedures: 06/2020 to 12/2022<br><br>Trial registration: NR	Passage to stomach:<br>50 (96%) vs<br>104 (100%)<br>Biliary sphincterotomy:<br>2 (4%) vs<br>36 (35%)(p<0·001).<br>Clearance of bile duct stones:<br>2 (4%) vs 10 (10%)<br>Placement of biliary stents:<br>48 (92%) vs 82 (78%)<br>Placement of pancreatic stents:<br>1 (2%) vs 6 (6%)<br>Balloon dilation of biliary stricture:<br>15 (29%) vs 56 (54%)	N/A	N/A	N/A
Gambaccini et al., 2023 (AB)<br><br>Italy<br><br>Dates of procedures: 07/2020 to 07/2022<br><br>Trial registration: NR	N/A	N/A	0 Worst – 10 Best<br>Median (range):<br>SU only<br><br>Ease of intubation of the oesophagus:<br>9 (4-10)<br>Navigability/push of the endoscope along the viscera:<br>10 (7-10).	N/A

			Suction insufflation performance: 10 (3-10)<br>Stability maintaining position: 9 (8-10)<br>Tip control and deflection: 8 (5-10)<br>Ability to selectively cannulate: 8 (5-10)<br>Elevator performance: 10 (8-10)<br>Operative channel performance with various devices: 10 (8-10)	
Liao et al., 2023 (AB)<br><br>USA<br><br>Dates of procedures: 01/2021 to 04/2023<br><br>Trial registration: NR	N/A	N/A	N/A	N/A
Razzak et al., 2023 (AB)<br><br>USA<br><br>Dates of procedures: 01/2020 to 02/2023<br><br>Trial registration: NR	N/A	N/A	N/A	N/A
Shahid et al., 2023 (FT)<br><br>Spain and USA<br><br>Dates of procedures: 06/2020 to 11/2021<br><br>Trial registration: NCT05041608	SUs only<br>Stone extraction: 53 (41%) vs 37 (51%)<br>Cholangioscopy: 62 (48%) vs 6 (8%).	Model D:<br>Appropriate papilla orientation:<br>Majority said easy to perform.<br>Cannulation efficiency:<br>Majority said easy to perform.<br>Sphincterotomy efficiency:<br>Majority said easy to perform.<br>aScope:<br>Appropriate papilla orientation:<br>Majority said easy to perform.<br>Cannulation efficiency:<br>Majority said easy to perform.<br>Sphincterotomy efficiency:<br>Majority said easy to perform.	1 High – 5 Low<br>Median:<br><br>Manoeuvrability based on papillary orientation:<br>Model D: 1,<br>aScope: 2.<br>Elevator efficiency:<br>Model D: 2,<br>aScope: 2.<br><br>Model D:<br>Elevator efficiency:<br>Majority said minimal to moderate difficulty.<br>Hand strain:<br>Majority said easy to perform.<br>aScope:<br>Elevator efficiency:<br>Majority said minimal to moderate difficulty.<br>Hand strain:<br>Majority said easy to perform.	Overall comparison to MU endoscope:<br>Model D:<br>Majority said inferior.<br>aScope:<br>Majority said inferior.
Gromski et al., 2021 (AB)<br><br>USA<br><br>Dates of procedures: 01/2020 to 04/2020<br><br>Trial registration: NR	N/A	N/A	1 Best – 5 Worst<br>Mean:<br><br>Scope stiffness:<br>4 vs 3 vs 3 ($p<0{\cdot}001$).<br>Air water button function:<br>4 vs 3 vs 3 ($p<0{\cdot}001$).<br>Elevator function:<br>4 vs 3 vs 3 ($p<0{\cdot}001$).<br>Hand strain:<br>4 vs 3 vs 3 ($p<0{\cdot}001$).	N/A
Gupta et al., 2021 (AB)<br><br>USA	N/A	N/A	N/A	1 Best – 5 Worst<br>Mean:<br><br>4 vs 5.

Dates of procedures: 09/2020 to 11/2020<br><br>Trial registration: NR				
Fugazza et al., 2024 (FT)<br><br>Belgium, India, Italy, Saudi Arabia, USA<br><br>Dates of procedures: 05/2020 to 09/2021<br><br>Trial registration: NR	N/A	N/A	N/A	1 Unsatisfied – 10 Very satisfied Mean (SD):<br><br>9 (±1).
Venkatachalapathy 2023 (AB)<br><br>Republic of Ireland and UK<br><br>Dates of procedures: 03/2021 to 08/2023<br><br>Trial registration: NCT04671095	N/A	N/A	Ease of intubation: NR	Qualitative assessment of the duodenoscope: NR
Bruno et al., 2023 (FT)<br><br>Australia, Canada, China, France, Germany, Hong Kong, India, Italy, Netherlands, Singapore, South Africa, USA<br><br>Dates of procedures: 03/2021 to 11/2022<br><br>Trial registration: NCT04103749	N/A	N/A	N/A	1 Worst – 10 Best Median (range):<br><br>8 (0-10)
Napoléon et al., 2022 (FT)<br><br>France<br><br>Dates of procedures: 09/2020 to 09/2020<br><br>Trial registration: 2020-A00346-33	Deep biliary cannulation: 54/54 (100%)<br>Double guidewire technique for cannulation: 4/4 (100%)<br>Septotomy: 3/3 100%)<br>Biliary sphincterotomy: 35/35 (100%)<br>Biliary large balloon dilation: 1/1 (100%)<br>CBD stone clearance: 25/25 (100%)<br>Mechanical lithotripsy (CBD stone): 2/2 (100%)<br>Biliary stent placement: 20/20 (100%)<br>Biliary stent removal: 9/9 (100%)<br>Biliary stent exchange: 8/8 (100%)<br><br>(Further detail in Table 2)	Percentage rated clinically satisfactory<br><br>Oesophagus intubation: 60 (100%)<br>Crossing stomach and pylorus: 60 (100%)<br>Crossing duodenal bulb and superior angle: 60 (100%)<br>Reaching the deepest point of the duodenum: 60 (100%)<br>Evaluation of the papilla: 55 91%<br><br>(Further detail in Table 3)	Percentage rated clinically satisfactory<br><br>Range of manoeuvrability: 60 (100%)<br>Suction performance: 60 (100%)<br>Scope in short or long position as required: 60 (100%)<br>Twisting the scope and orienting the tip: 60 (100%)<br>Scope stability during cannulation: 60 (100%)<br>Elevator's function: 60 (100%)<br><br>(Further detail in Table 3)	1 Worst – 10 Best Median (IQR):<br><br>9 (7-9)<br><br>Endoscopist satisfaction <6: 2 (3%)<br><br>Percentage rated comparable to MU duodenoscope<br><br>Oesophagus intubation: 58 (97%)<br>Crossing stomach and pylorus: 57 (95%)

				(Further detail in Table 3)
Persyn et al., 2022 (FT)<br><br>Belgium<br><br>Dates of procedures: 10/2020 to 11/2020<br><br>Trial registration: NR	N/A	N (%), Preferred to MU duodenoscope, Neutral, Not preferred to MU duodenoscope<br><br>Biliary sphincterotomy 12, 0/12 (0%), 10/12 (83%), 2/12 (17%),<br>Cannulation of main bile duct 47, 0/47 (0%), 43/47 (92%), 4/47 (9%),<br>Cannulation of pancreatic duct 13, 0/13 (0%), 12/13 (92%), 1/13 (8%).<br><br>(Further detail in Table 2)	1 Not preferred – 5 Comparable to MU duodenoscope<br>Median:<br><br>Ease and ability to intubate oesophagus: 5<br>Ease and ability to transverse stomach and pylorus: 5 Inadvertent slippage out of the duodenum: 5<br><br>(Further detail in Figure 2)	1 Unsatisfied – 10 Very satisfied<br>Median:<br><br>8
Slivka et al., 2021 (FT)<br><br>Netherlands and USA<br><br>Dates of procedures: 01/2020 to 02/2021<br><br>Trial registration: NCT04223830	Total manoeuvres: 702/721 (97%)	N/A	1 Not preferred – 5 Comparable to MU duodenoscope<br>Median (range):<br><br>Ease and ability to intubate oesophagus: 4 (1-5)<br>Ease and ability to transverse stomach and pylorus: 4 (1-5)<br>Inadvertent slippage out of the duodenum: 4 (1-5)<br><br>(Further detail in Table 4)	1 Unsatisfied – 10 Very satisfied<br>Median (range):<br><br>8 (1-10)
Muthusamy et al., 2020 (FT)<br><br>USA<br><br>Dates of procedures: 04/2019 to 05/2019<br><br>Trial registration: NCT03701958	Total manoeuvres: 702/721 (97%)	n, Preferred to MU duodenoscope, Neutral, Not preferred to MU duodenoscope<br><br>Biliary sphincterotomy 14, 0/14 (0%), 14/14 (100%), 2/14 (0%)<br>Pancreatic sphincterotomy 1, 0/1 (0%), 1/1 (100%), 0/1 (0%), Cannulation of pancreatic duct 7, 0/7 (0%), 7/7 (100%), 0/7 (0%).<br><br>(Further detail in Table 3)	1 Not preferred – 5 Comparable to MU duodenoscope<br>Median (range):<br><br>Ease and ability to intubate oesophagus: 5 (2-5)<br>Ease and ability to transverse stomach and pylorus: 5 (3-5)<br>Inadvertent slippage out of the duodenum: 4 (1-5)<br><br>(Further detail in Table 3).	1 Unsatisfied – 10 Very satisfied<br>Median (range):<br><br>9 (1-10)
**Other SU duodenoscopes**				
Shiratori et al., 2024 (AB)<br><br>USA<br><br>Dates of procedures: 07/2020 to 09/2023<br><br>Trial registration: NR	N/A	N/A	N/A	N/A
Pona et al., 2023 (AB)<br><br>USA<br><br>Dates of procedures: 09/2022 to 10/2022	N/A	N/A	N/A	N/A

Trial registration: NR				
Hutfless et al., 2022 (FT)<br><br>USA<br><br>Dates of procedures: 01/2015 to 12/2021<br><br>Trial registration: NR	N/A	N/A	N/A	N/A
USA<br><br>01/2017 to 12/2017<br><br>Trial registration: NR	N/A	N/A	N/A	N/A
**MU duodenoscope with SU elevator / end-cap**				
Forbes et al., 2023 (FT)<br><br>Canada<br><br>Dates of procedures: 12/2019 to 02/2022<br><br>Trial registration: NCT04040504	N/A	N/A	N/A.	N/A
Barakat et al., 2024 (FT)<br><br>USA<br><br>Dates of procedures: 2021 to 2022 (18-month period)<br><br>Trial registration: NR	N/A	N/A	N/A	N/A
Lee et al., 2023 (AB)<br><br>South Korea<br><br>Dates of procedures: NR<br><br>Trial registration: NR	N/A	N/A	N/A	N/A
Straulino et al., 2019 (AB)<br><br>Germany<br><br>Dates of procedures: NR<br><br>Trial registration: NR	N/A	N/A	N/A	N/A
**MU endoscopes only**				
Kwakman et al., 2022 (FT)<br><br>The Netherlands	N/A	N/A	N/A	N/A

Dates of publications: 01/2008 to 12/2019<br><br>Trial registration: N/A	

[a] AB: conference abstract; FT – full-text article.

AB: conference abstract; FT: full-text article; IQR: interquartile range; MU: multiple-use (endoscope); N/A: Not applicable; NR: not reported; PR: posted results; SD; Standard deviation; SU: single-use (endoscope); TR: trial registry record.

Supplementary Table 8. Technical outcomes – Duration: Duodenoscopes

Author, year, country, publication type [a], dates of procedures, trial registration	SU group vs MU group - Time to landmarks / cannulate / intubate	SU group vs MU group - Time for procedure	SU group vs MU group - Time for (pre-) cleaning / discarding / reprocessing instruments / turning around room / discharging patient / transportation
**SU duodenoscopes: EXALT Model D (Boston Scientific)**			
Bang et al., 2021 (FT)<br><br>USA<br><br>Dates of procedures: 01/2020 to 03/2020<br><br>Trial registration: NCT04143698	Time to papilla:<br>Mean (SD) seconds:<br>34 (56) vs 33 (82).<br><br>Time to cannulate:<br>Mean (SD) seconds:<br>240 (650) vs 360 (524) (p=0·010).	Mean (SD) minutes: 23 (19) vs 23 (15).<br><br>Median minutes: 14.8 vs 18.3.	N/A
Machado et al., 2024 (AB)<br><br>USA<br><br>Dates of procedures: 06/2020 to 12/2022<br><br>Trial registration: NR	N/A	Mean (SD) minutes:<br>Exalt D<br>43 (32)<br>TJF-Q190V<br>55 (39).	N/A
Gambaccini et al., 2023 (AB)<br><br>Italy<br><br>Dates of procedures: 07/2020 to 07/2022	Time to papilla:<br>Mean (±SD) seconds:<br>37 (±41) vs 33 (±43).<br><br>Time to cannulate:	Mean (SD) minutes: 44 (±38) vs 38 (±43).	N/A

Trial registration: NR	Mean (±SD) seconds: 488 (±511) vs 416 (±550).		
Liao et al., 2023 (AB)<br><br>USA<br><br>Dates of procedures: 01/2021 to 04/2023<br><br>Trial registration: NR	N/A	N/A	N/A
Razzak et al., 2023 (AB)<br><br>USA<br><br>Dates of procedures: 01/2020 to 02/2023<br><br>Trial registration: NR	N/A	Mean minutes: 48 vs 46.	N/A
Shahid et al., 2023 (FT)<br><br>Spain and USA<br><br>Dates of procedures: 06/2020 to 11/2021<br><br>Trial registration: NCT05041608	N/A	Mean minutes: Model D: 73, aScope: 87.	N/A
Gromski et al., 2021 (AB)<br><br>USA<br><br>Dates of procedures: 01/2020 to 04/2020<br><br>Trial registration: NR	N/A	Mean minutes: 35 vs 37 vs 36.	N/A
Gupta et al., 2021 (AB)<br><br>USA<br><br>Dates of procedures: 09/2020 to 11/2020<br><br>Trial registration: NR	Time to ampulla:<br>Mean (SD) minutes:<br>3 (2) vs 4 (1).<br><br>Time to cannulate:<br>Mean (SD) minutes:<br>11 (7) vs 13 (10).	Mean (SD) minutes: 40 (23) vs 46 (42).	Mean (SD) minutes: 1 (1) vs 2 (1).
Fugazza et al., 2024 (FT)<br><br>Belgium, India, Italy, Saudi Arabia, USA<br><br>Dates of procedures: 05/2020 to 09/2021	N/A	Median (IQR) minutes: 64 (15-189).	N/A

Trial registration: NR			
Venkatachalapathy 2023 (AB)<br><br>Republic of Ireland and UK<br><br>Dates of procedures: 03/2021 to 08/2023<br><br>Trial registration: NCT04671095	N/A	NR	N/A
Bruno et al., 2023 (FT)<br><br>Australia, Canada, China, France, Germany, Hong Kong, India, Italy, Netherlands, Singapore, South Africa, USA<br><br>Dates of procedures: 03/2021 to 11/2022<br><br>Trial registration: NCT04103749	N/A	Median (range) minutes:<br>24 (2-157).	N/A
Napoléon et al., 2022 (FT)<br><br>France<br><br>Dates of procedures: 09/2020 to 09/2020<br><br>Trial registration: 2020-A00346-33	N/A	N/A	N/A
Persyn et al., 2022 (FT)<br><br>Belgium<br><br>Dates of procedures: 10/2020 to 11/2020<br><br>Trial registration: NR	N/A	Mean minutes:<br>56.	N/A
Slivka et al., 2021 (FT)<br><br>Netherlands and USA<br><br>Dates of procedures: 01/2020 to 02/2021<br><br>Trial registration: NCT04223830	Time to cannulate:<br>NR.	Median (SD) minutes:<br>26 (3-125).	N/A
Muthusamy et al., 2020 (FT)<br><br>USA<br><br>Dates of procedures:	N/A	N (%) minutes:<br>≥20 24 (40%),<br>21-40 19 (32%),<br>40-60 8 (13%),	N/A

04/2019 to 05/2019<br><br>Trial registration:<br>NCT03701958		≥60 9 (15%).	
**Other SU duodenoscopes**			
Shiratori et al., 2024 (AB)<br><br>USA<br><br>Dates of procedures:<br>07/2020 to 09/2023<br><br>Trial registration:<br>NR	N/A	N/A	N/A
Pona et al., 2023 (AB)<br><br>USA<br><br>Dates of procedures:<br>09/2022 to 10/2022<br><br>Trial registration:<br>NR	N/A	N/A	Mean (range) minutes:<br>37 (22-52) vs 33 (26-41). [b]
Hutfless et al., 2022 (FT)<br><br>USA<br><br>Dates of procedures:<br>01/2015 to 12/2021<br><br>Trial registration:<br>NR	N/A	N/A	N/A
USA<br><br>Dates of procedures:<br>01/2017 to 12/2017<br><br>Trial registration:<br>NR	N/A	N/A	N/A
**MU duodenoscope with SU elevator / end-cap**			
Forbes et al., 2023 (FT)<br><br>Canada<br><br>Dates of procedures:<br>12/2019 to 02/2022<br><br>Trial registration:<br>NCT04040504	N/A	Median (IQR) minutes:<br><br>14 (10-24) vs 17 (11-27) (p=N/A).	N/A
Barakat et al., 2024 (FT)<br><br>USA<br><br>Dates of procedures:<br>2021 to 2022<br>(18-month period)	N/A	N/A	N/A

Trial registration: NR			
Lee et al., 2023 (AB)<br><br>South Korea<br><br>Dates of procedures: NR<br><br>Trial registration: NR	N/A	N/A	N/A
Straulino et al., 2019 (AB)<br><br>Germany<br><br>Dates of procedures: NR<br><br>Trial registration: NR	Time to papilla duodena:<br>Mean seconds:<br>81 vs 74.<br><br>Time to intubation of the common bile duct:<br>Mean seconds:<br>391 vs 746).	N/A	Time to manual pre-clean:<br>Mean seconds: 325 vs 305.
**MU endoscopes only**			
Kwakman et al., 2022 (FT)<br><br>The Netherlands<br><br>Date of publications: 01/2008 – 12/2019<br><br>Trial registration: N/A	N/A	N/A	N/A

[a] AB: conference abstract; FT – full-text article. [b] Patient turnover time was defined as time when patient out of room to the time the next patient in room; cases where the turnover was impacted by other issues such as intravenous access, or a late patient, or patient out of room delay due to patient status or post anaesthesia care unit delays were excluded.

AB: conference abstract; FT: full-text article; IQR: interquartile range; MU: multiple-use (endoscope); N/A: Not applicable; NR: not reported; PR: posted results; SD: Standard deviation; SU: single-use (endoscope); TRR trial registry record.

1.2 Test Accuracy

No included studies of SU duodenoscopes evaluated test accuracy.

1.3 Infection risk and adverse events

Supplementary Table 9. Adverse events and infection rates: Duodenoscopes

Author, year, country, publication type [a], dates of procedures, trial registration	SU group - N / % non-infectious adverse events (AEs)	SU group - n / % infectious AEs [b]	MU group - n / % non-infectious AEs	MU group - n / % infectious AEs [b]
**SU duodenoscopes: EXALT Model D (Boston Scientific)**				
Bang et al., 2021 (FT)<br><br>USA<br><br>Dates of procedures: 01/2020 to 03/2020<br><br>Trial registration: NCT04143698	2 patients (4%) reported an AE within 7 days: 1 patient (2%) pancreatitis and hospitalisation.	1 patient (2%): cholangitis, E coli bacteraemia, sepsis, and death. Attributed to ongoing endogenous infection*.	4 patients (8%): 2 patients (4%) pancreatitis (1 mild; 1 moderate), 1 (2%) mild bleeding, 1 (2%) atrial fibrillation, cardiogenic shock, and death.	Reported 0.
Machado et al., 2024 (AB)<br><br>USA<br><br>Dates of procedures: 06/2020 to 12/2022<br><br>Trial registration: NR	Model D<br>1 patient (2%) reported an AE: 1 (2%) mild pancreatitis.<br>TJF-Q190V<br>7 patients (6%): 3 (3%) mild pancreatitis, 2 (2%) abdominal pain, 2 (2%) post-sphincterotomy bleeding.	None reported	N/A	N/A
Gambaccini et al., 2023 (AB)<br><br>Italy<br><br>Dates of procedures: 07/2020 to 07/2022<br><br>Trial registration: NR	10 patients (42%) reported an AE: 3 (12%) pancreatitis, 2 (8%) abdominal or epigastric pain, 1 (4%) nausea and vomiting	2 patients (8%) fever	NR	NR
Liao et al., 2023 (AB)<br><br>USA<br><br>Dates of procedures: 01/2021 to 04/2023<br><br>Trial registration: NR	Model D:<br>1 (2%) reported peri-operative AE:<br>1 (2%) intrahepatic duct leak.<br>3 (5%) reported extended AE within 10 days:<br>1 (2%) sphincterotomy bleed, 1 (2%) pneumoperitoneum, 1 (2%) cholecystitis.<br><br>aScope:<br>peri-operative AE:<br>0 (0%) reported peri-operative (P=1.0),<br>0 (0%) reported extended AEs (P=0.24).	None reported	N/A	N/A

Author, year, country, publication type [a], dates of procedures, trial registration	SU group - N / % non-infectious adverse events (AEs)	SU group - n / % infectious AEs [b]	MU group - n / % non-infectious AEs	MU group - n / % infectious AEs [b]
Razzak et al., 2023 (AB)<br><br>USA<br><br>Dates of procedures: 01/2020 to 02/2023<br><br>Trial registration: NR	13 patients (25%) reported an AE. 1 bleeding event due to long linear gastric ulcer caused by direct scope trauma upon withdrawal. (No further detail.)<br>30-day readmission rate 15%.	None reported	9 patients (11%). (No further detail.)<br><br>30-day readmission rate 9%	None reported
Shahid et al., 2023 (FT)<br><br>Spain and USA<br><br>Dates of procedures: 06/2020 to 11/2021<br><br>Trial registration: NCT05041608	23 patients (12%) reported AE* within 7 days:<br>Model D: 13 (11%). 5 (4%) post-ERCP pancreatitis.<br><br>aScope: 10 (14%). 3 (4%) post-ERCP pancreatitis. (Further detail NR.)	None reported	NR	NR
Gromski et al., 2021 (AB)<br><br>USA<br><br>Dates of procedures: 01/2020 to 04/2020<br><br>Trial registration: NR	NR	NR	NR	NR
Gupta et al., 2021 (AB)<br><br>USA<br><br>Dates of procedures: 09/2020 to 11/2020<br><br>Trial registration: NR	NR	NR	NR	NR
Fugazza et al., 2024 (FT)<br><br>Belgium, India, Italy, Saudi Arabia, USA<br><br>Dates of procedures: 05/2020 to 09/2021<br><br>Trial registration: NR	4 patients (6%) reported an AE* within 14 days: 1 (2%) bleeding (mild), 2 (3%) post-ERCP pancreatitis (1 severe/<br>1 mild).	1 (2%) cholangitis (moderate).	N/A	N/A
Venkatachalapathy 2023 (AB)<br><br>Republic of Ireland and UK<br><br>Dates of procedures:	NR	Risk of infection post-procedure: 10%.	N/A	N/A

Author, year, country, publication type [a], dates of procedures, trial registration	SU group - N / % non-infectious adverse events (AEs)	SU group - n / % infectious AEs [b]	MU group - n / % non-infectious AEs	MU group - n / % infectious AEs [b]
03/2021 to 08/2023<br><br>Trial registration: NCT04671095				
Bruno et al., 2023 (FT)<br><br>Australia, Canada, China, France, Germany, Hong Kong, India, Italy, Netherlands, Singapore, South Africa, USA<br><br>Dates of procedures: 03/2021 to 11/2022<br><br>Trial registration: NCT04103749	43 patients (8%) experienced 46 SAEs related to the device or procedure within 30 days: 18 (3%) developed pancreatitis, 6 (1%) experienced pain, 5 (1%) gastrointestinal bleed, 3 (0.5%) developed cholecystitis, 2 (0·5%) perforation (11%). Fatal-related SAEs: 3 (1%), including 1 fatal on day 70.	3 (0·5%) developed sepsis, 2 (0.5%) developed cholangitis, 2 (0.5%) developed fever, 2 (0.5%) developed bacteraemia.	N/A	N/A
Napoléon et al., 2022 (FT)<br><br>France<br><br>Dates of procedures: 09/2020 to 09/2020<br><br>Trial registration: 2020-A00346-33	3 patients (5%) experienced an AE* within 7 days: 1 (2%) acute pancreatitis, biliary pain 1 (2%), 1 (2%) death*.	Reported 0.	N/A	N/A
Persyn et al., 2022 (FT)<br><br>Belgium<br><br>Dates of procedures: 10/2020 to 11/2020<br><br>Trial registration: NR	1 patient (2%) reported an AE* within 7 days: 1 (2%) post-sphincterotomy bleeding.	Reported 0.	N/A	N/A
Slivka et al., 2021 (FT)<br><br>Netherlands and USA<br><br>Dates of procedures: 01/2020 to 02/2021<br><br>Trial registration: NCT04223830	13 patients (7%) reported 18 SAEs within 7 days: 5 (3%) bleeding events: 3 (2%) acute pancreatitis: epigastric or abdominal pain: 2 (1%); 1 (0·5%) choledocholithiasis, 1 (0·5%) nausea and vomiting.	2 (1%) cholangitis, 1 (0·5%) systemic inflammatory response syndrome, 1 (0.5%) fever, 1 (0.5%) suspected pneumonia, 1 (0.5%) urinary tract infection.	N/A	N/A
Muthusamy et al., 2020 (FT)<br><br>USA<br><br>Dates of procedures: 04/2019 to 05/2019	5 patients reported an SAE* within 7 days.<br>Roll-in manoeuvres: 1 (8%) post-ERCP pancreatitis.<br>ERCPs: 4 (7%) 2 (3%) post-ERCP pancreatitis, 1 (2%)	Reported 0.	N/A	N/A

Author, year, country, publication type [a], dates of procedures, trial registration	SU group - N / % non-infectious adverse events (AEs)	SU group - n / % infectious AEs [b]	MU group - n / % non-infectious AEs	MU group - n / % infectious AEs [b]
Trial registration: NCT03701958	post-sphincterotomy bleeding, 1 (2%) hospitalisation.			
**Other SU duodenoscopes**				
Shiratori et al., 2024 (AB)<br><br>USA<br><br>Dates of procedures: 07/2020 to 09/2023<br><br>Trial registration: NR	68 patients (2%) experienced pancreatitis.<br><br>7-day readmission rate 117 (4%),	7-day post-ERCP infection rate: 51 (2%), odds ratio 1·30 (95% CI, 0.98-1.73)(p=0·07).<br><br>30 -day post-ERCP infection rate: 138 (4%), odds ratio 1.11 (95% CI, 0.94-1·33)(p=0·23).<br><br>Post-ERCP infection risk factors: History of hospitalization within 30 days before ERCP, (odds ratio=1·99, 95%CI 1·86-2·13), infection at the ERCP (bacteraemia)(odds ratio=2·14, 95%CI 1·87-2·45).	2,815 patients (2%) experienced pancreatitis.<br><br>7-day readmission rate 4,534 (3%).	7-day post-ERCP infection rate: 1,675 (1%)<br><br>30 -day post-ERCP infection rate: 5,349 (4%).
Pona et al., 2023 (AB)<br><br>USA<br><br>Dates of procedures: 09/2022 to 10/2022<br><br>Trial registration: NR	NR	NR	NR	NR
Hutfless et al., 2022 (FT)<br><br>USA<br><br>Dates of procedures: 01/2015 to 12/2021<br><br>Trial registration: NR	NR	7-day post-ERCP hospitalisation for infection rate: 10/711 patients (1%) 30 -day post-ERCP infection rate: 25/711 (3%).<br><br>Odds ratio of hospitalisation for infection after ERCP, SU vs MU: 7-day infection: 0·32 (0·17 to 0·60) 30-day infection: 0·47 (0·31 to 0·69)<br><br>Post-ERCP hospitalisation for infection risk factors: Urgent ERCPs, chronic conditions, being	NR	NR

Author, year, country, publication type [a], dates of procedures, trial registration	SU group - N / % non-infectious adverse events (AEs)	SU group - n / % infectious AEs [b]	MU group - n / % non-infectious AEs	MU group - n / % infectious AEs [b]
		older, non-white, or male.		
USA<br><br>Dates of procedures: 01/2017 to 12/2017<br><br>Trial registration: NR	N/A	N/A	NR	399 (2%) hospitalisation for infection within 7 days. 913 (6%) hospitalisation for infection within 30 days.<br><br>Post-ERCP infection risk factors: Urgent ERCPs, chronic conditions, being non-white, or male.
**MU duodenoscope with SU elevator / end-cap**				
Forbes et al., 2023 (FT)<br><br>Canada<br><br>Dates of procedures: 12/2019 to 02/2022<br><br>Trial registration: NCT04040504	21 patients (8%) experienced an SAE* within 30 days: 8 patients (3%) pancreatitis, 5 (2%) bleeding, 1 (0·5%), perforation, 4 (2%) cardiorespiratory events. 1 (0·5%) death.	2 (1%) cholangitis.<br><br>Number of scopes with CFU or growth: 2 (1%) elevator region, 5 (2%) instrument channel, 1 (1%) both areas.<br><br>Persistent microbial contamination detected in 8/208 (4%) (P =0·004), relative risk 0.34 (95% CI, 0.16-0·75). Number needed to treat to avoid persistent contamination: 14 (95% CI, 8·1-42·7).	18 patients (7%)*: 9 patients (4%) pancreatitis (P>0·99), 3 (1%) bleeding (P=·72), 0 (0%) perforation (P>0·99), 2 (1%) cardiorespiratory events (P=.69). 1 (0.5%) death (P>0·99).	3 (1%) cholangitis (P>0·99).<br><br>Number of scopes with CFU or growth: 2 (1%) elevator region, 21 (10%) instrument channel, 1 (1%) both areas.<br><br>Persistent microbial contamination detected in 24/214 (11%).
Barakat et al., 2024 (FT)<br><br>USA<br><br>Dates of procedures: 2021 to 2022 (18-month period)<br><br>Trial registration: NR	10 patients (22%) mucosal / oropharyngeal trauma (including lacerations), 4 (9%) mild post-ERCP pancreatitis.	Reported 0.	0 patients (0%) mucosal / oropharyngeal trauma (including lacerations)(P<0·05). 1 (3%) mild post-ERCP pancreatitis (P<0·05).	Reported 0.
Lee et al., 2023 (AB)<br><br>South Korea<br><br>Dates of procedures: NR<br><br>Trial registration: NR	16 patients (16%) experienced an AE. (Details NR)	10 (10%) post-ERCP cholangitis. 1 (1%) caused by MDR pathogens.	14 patients (15%) experienced an AE. (Details NR)	10 (11%) post-ERCP cholangitis (P=0·90 vs MUDC), 2 (2%) caused by MDR pathogens (P=0·39 vs MUDC).
Straulino et al., 2019 (AB)<br><br>Germany<br><br>Dates of procedures: NR	2 patients (6%) experienced an AE. 1 (3%) bleeding, 1 (3%) pancreatitis.	None reported	3 patients (8%): 2 (5%) pancreatitis, 1 (3%) bleeding,	None reported

Author, year, country, publication type [a], dates of procedures, trial registration	SU group - N / % non-infectious adverse events (AEs)	SU group - n / % infectious AEs [b]	MU group - n / % non-infectious AEs	MU group - n / % infectious AEs [b]
Trial registration: NR				
**MU endoscopes only**				
Kwakman et al., 2022 (FT)<br><br>The Netherlands<br><br>Dates of publications: 01/2008 to 12/2019<br><br>Trial registration: N/A	N/A	N/A	None reported	Attack rate: ≥ 8·3%-35% per contaminated duodenoscope.<br>Duodenoscope-associated colonisation (DAC) rate:<br>52 (Estimated number ERCPs 181,209– 227,006)<br>Minimum estimated DAC risk 0·023% – 0·029% approx.<br>Duodenoscope-associated infection (DAI) rate:<br>21 (Estimated number ERCPs 181,209– 227,006)<br>Minimum estimated DAI risk 0·01% approx.<br>(Further detail in Table 1)<br>UMC Utrecht: > 200 colony-forming units (CFU)/20mL and 34 CFU/20mL of the microorganism of interest (gut flora), detected in cultures.

[a] AB: conference abstract; FT – full-text article. [b] Where a study explicitly reported no occurrence of infection, "Reported 0" is registered here. Where the study authors reported (other) adverse events but no infectious adverse events were mentioned, "None reported" is registered here.

AB: conference abstract; AE: Adverse Event; DAC: duodenoscope-associated colonisation; DAI: duodenoscope-associated infection; ERCP: endoscopic retrograde cholangiopancreatography; FT: full-text article; MU: multiple-use (endoscope); MUDC: multiple-use endoscopes with disposable cap; N/A: not applicable; NR: not reported; PR: posted results; SAE: Serious Adverse Event; SU: single-use endoscopes; TRR: trial registry record: UMC: University Medical Centres.

*Not related to endoscope

1.4 Other outcomes

Supplementary Table 10. Clinical outcomes / Patient Reported Outcomes: Duodenoscopes

Author, year, country, publication type [a], dates of procedures, trial registration	Clinical outcomes/ Patient Reported Outcome Measures (PROMs)
**EXALT Model D (Boston Scientific)**	
Bang et al., 2021 (FT)<br><br>USA<br><br>Dates of procedures: 01/2020 to 03/2020<br><br>Trial registration: NCT04143698	N/A
Machado et al., 2024 (AB)<br><br>USA<br><br>Dates of procedures: 06/2020 to 12/2022<br><br>Trial registration: NR	N/A
Gambaccini et al., 2023 (AB)<br><br>Italy<br><br>Dates of procedures: 07/2020 to 07/2022<br><br>Trial registration: NR	N/A
Liao et al., 2023 (AB)<br><br>USA<br><br>Dates of procedures: 01/2021 to 04/2023<br><br>Trial registration: NR	N/A
Razzak et al., 2023 (AB)<br><br>USA<br><br>Dates of procedures: 01/2020 to 02/2023<br><br>Trial registration: NR	N/A
Shahid et al., 2023 (FT)<br><br>Spain, USA<br><br>Dates of procedures: 06/2020 to 11/2021<br><br>Trial registration: NCT05041608	N/A
Gromski et al., 2021 (AB)	N/A

USA<br><br>Dates of procedures: 01/2020 to 04/2020<br><br>Trial registration: NR	
Gupta et al., 2021 (AB)<br><br>USA<br><br>Dates of procedures: 09/2020 to 11/2020<br><br>Trial registration: NR	N/A
Fugazza et al., 2024 (FT)<br><br>Belgium, India, Italy, Saudi Arabia, USA<br><br>Dates of procedures: 05/2020 to 09/2021<br><br>Trial registration: NR	N/A
Venkatachalapathy 2023 (AB)<br><br>Republic of Ireland and UK<br><br>Dates of procedures: 03/2021 to 08/2023<br><br>Trial registration: NCT04671095	Patient discomfort<br>N (%) rating by HCP:<br><br>In room:<br>Comfortable or mild discomfort: 60 (100%)<br>In recovery:<br>Comfortable or mild discomfort: 59 (99%)<br><br>Quality of life<br>(EQ-ED-5L questionnaire):<br>1 No problem, 2 Moderate problem, 3 Severe problem<br><br>“increased progressively over the follow-up days. However, patients with bile duct stone indication and males reported significantly better health states than patients with bile duct stricture indication or females.”
Bruno et al., 2023 (FT)<br><br>Australia, Canada, China, France, Germany, Hong Kong, India, Italy, Netherlands, Singapore, South Africa, USA<br><br>Dates of procedures: 03/2021 to 11/2022<br><br>Trial registration: NCT04103749	N/A
Napoléon et al., 2022 (FT)<br><br>France<br><br>Dates of procedures: 09/2020 to 09/2020	N/A

Trial registration: 2020-A00346-33	
Persyn et al., 2022 (FT)<br><br>Belgium<br><br>Dates of procedures: 10/2020 to 11/2020<br><br>Trial registration: NR	N/A
Slivka et al., 2021 (FT)<br><br>Netherlands and USA<br><br>Dates of procedures: 01/2020 to 02/2021<br><br>Trial registration: NCT04223830	N/A
Muthusamy et al., 2020 (FT)<br><br>USA<br><br>Dates of procedures: 04/2019 to 05/2019<br><br>Trial registration: NCT03701958	N/A
**Other SU duodenoscopes**	
Shiratori et al., 2024 (AB)<br><br>USA<br><br>Dates of procedures: 07/2020 to 09/2023<br><br>Trial registration: NR	N/A
Pona et al., 2023 (AB)<br><br>USA<br><br>Dates of procedures: 09/2022 to 10/2022<br><br>Trial registration: NR	N/A
Hutfless et al., 2022 (FT)<br><br>USA<br><br>Dates of procedures: 01/2015 to 12/2021<br><br>Trial registration: NR	N/A
USA<br><br>Dates of procedures: 01/2017 to 12/2017<br><br>Trial registration: NR	N/A
**MU duodenoscope with SU elevator / end-cap**	
Forbes et al., 2023 (FT)<br><br>Canada	N/A

Dates of procedures: 12/2019 to 02/2022<br><br>Trial registration: NCT04040504	
Barakat et al., 2024 (FT)<br><br>USA<br><br>Dates of procedures: 2021 to 2022 (18-month period)<br><br>Trial registration: NR	N/A
Lee et al., 2023 (AB)<br><br>South Korea<br><br>Dates of procedures: NR<br><br>Trial registration: NR	N/A
Straulino et al., 2019 (AB)<br><br>Germany<br><br>Dates of procedures: NR<br><br>Trial registration: NR	N/A
**MU endoscopes only**	
Kwakman et al., 2022 (FT)<br><br>The Netherlands<br><br>Dates of publications: 01/2008 to 12/2019<br><br>Trial registration: N/A	N/A

[a] AB: conference abstract; FT – full-text article. [b] Patient turnover time was defined as time when patient out of room to the time the next patient in room; cases where the turnover was impacted by other issues such as intravenous access, or a late patient, or patient out of room delay due to patient status or post anaesthesia care unit delays were excluded.

AB: conference abstract; FT: full-text article; HCP: healthcare professional; IQR: interquartile range; MU: multiple-use (endoscope); N/A: not applicable; NR: not reported; PR: posted results; SU: single-use (endoscope); TRR: trial registry record.

Patient views: Duodenoscopes

No included studies of SU duodenoscopes reported patient views.

Supplementary Table 11. Views of staff using, procuring, cleaning, and / or disposing: Duodenoscopes

Author, year, country, publication type [a], dates of	Views of people in using, procuring, cleaning and / or disposing of endoscopes

procedures, trial registration	
**SU duodenoscopes: EXALT Model D (Boston Scientific)**	
Bang et al., 2021 (FT)<br><br>USA<br><br>Dates of procedures: 01/2020 to 03/2020<br><br>Trial registration: NCT04143698	N/A
Machado et al., 2024 (AB)<br><br>USA<br><br>Dates of procedures: 06/2020 to 12/2022<br><br>Trial registration: NR	N/A
Gambaccini et al., 2023 (AB)<br><br>Italy<br><br>Dates of procedures: 07/2020 to 07/2022<br><br>Trial registration: NR	N/A
Liao et al., 2023 (AB)<br><br>USA<br><br>Dates of procedures: 01/2021 to 04/2023<br><br>Trial registration: NR	N/A
Razzak et al., 2023 (AB)<br><br>USA<br><br>Dates of procedures: 01/2020 to 02/2023<br><br>Trial registration: NR	N/A
Shahid et al., 2023 (FT)<br><br>Spain and USA<br><br>Dates of procedures: 06/2020 to 11/2021<br><br>Trial registration: NCT05041608	N/A
Gromski et al., 2021 (AB)<br><br>USA<br><br>Dates of procedures: 01/2020 to 04/2020<br><br>Trial registration: NR	N/A
Gupta et al., 2021 (AB)<br><br>USA<br><br>Dates of procedures:	N/A

09/2020 to 11/2020<br><br>Trial registration: NR	
Fugazza et al., 2024 (FT)<br><br>Belgium, India, Italy, Saudi Arabia, USA<br><br>Dates of procedures: 05/2020 to 09/2021<br><br>Trial registration: NR	N/A
Venkatachalapathy 2023 (AB)<br><br>Republic of Ireland and UK<br><br>Dates of procedures: 03/2021 to 08/2023<br><br>Trial registration: NCT04671095	N/A
Bruno et al., 2023 (FT)<br><br>Australia, Canada, China, France, Germany, Hong Kong, India, Italy, Netherlands, Singapore, South Africa, USA<br><br>Dates of procedures: 03/2021 to 11/2022<br><br>Trial registration: NCT04103749	N/A
Napoléon et al., 2022 (FT)<br><br>France<br><br>Dates of procedures: 09/2020 to 09/2020<br><br>Trial registration: 2020-A00346-33	N/A
Persyn et al., 2022 (FT)<br><br>Belgium<br><br>Dates of procedures: 10/2020 to 11/2020<br><br>Trial registration: NR	N/A
Slivka et al., 2021 (FT)<br><br>Netherlands and USA<br><br>Dates of procedures: 01/2020 to 02/2021<br><br>Trial registration: NCT04223830	N/A
Muthusamy et al., 2020 (FT)<br><br>USA	N/A

Dates of procedures: 04/2019 to 05/2019<br><br>Trial registration: NCT03701958	
**Other SU duodenoscopes**	
Shiratori et al., 2024 (AB)<br><br>USA<br><br>Dates of procedures: 07/2020 to 09/2023<br><br>Trial registration: NR	N/A
Pona et al., 2023 (AB)<br><br>USA<br><br>Dates of procedures: 09/2022 to 10/2022<br><br>Trial registration: NR	N/A
Hutfless et al., 2022 (FT)<br><br>USA<br><br>Dates of procedures: 01/2015 to 12/2021<br><br>Trial registration: NR	N/A
USA<br><br>Dates of procedures: 01/2017 to 12/2017<br><br>Trial registration: NR	N/A
**MU duodenoscope with SU elevator / end-cap**	
Forbes et al., 2023 (FT)<br><br>Canada<br><br>Dates of procedures: 12/2019 to 02/2022<br><br>Trial registration: NCT04040504	N/A
Barakat et al., 2024 (FT)<br><br>USA<br><br>Dates of procedures: 2021 to 2022 (18-month period)<br><br>Trial registration: NR	Thematic language analysis around when using the SU end cap.<br><br>Stiffness<br>“The tip of the duodenoscope (with single-use end cap) doesn't seem to angulate as well for advancing the scope to the upper esophagus”<br>“When I remove the duodenoscope (with single-use end cap) there is often some superficial laceration with bleeding in the oropharynx and this doesn't happen with the standard duodenoscope.”<br>“Passing scope through oropharynx is more challenging and frequently must ask for anesthesia to assist.”<br>Alignment<br>“The field of view is greater, but it seems to make cannulation more challenging rather than easier.”

	"Perhaps due to the narrow pediatric lumens, the cap, and the altered field of view, more often than not the scope is not parallel to the ampulla. It's just not the best angle for cannulation or instrumentation."<br>"I try to use this in older/bigger teenage patients as opposed to younger patients, would not try it in anybody less than 25-30 Kg."<br>Cannulation<br>"The field of view (of the duodenoscope with single-use end cap) is greater, but it seems to make cannulation more challenging rather than easier."<br>"I find myself using needle knife more often with this scope than with a standard duodenoscope because initial cannulation is more difficult and less successful."<br>"When I remove the duodenoscope (with single-use end cap) there is often some superficial laceration with bleeding in the oropharynx and this doesn't happen with the standard duodenoscope."<br>"In older patients it is more possible to cannulate with this scope, but harder for the younger ones."	
Lee et al., 2023 (AB)<br><br>South Korea<br><br>Dates of procedures: NR<br><br>Trial registration: NR	N/A	
Straulino et al., 2019 (AB)<br><br>Germany<br><br>Dates of procedures: NR<br><br>Trial registration: NR	Feeling from the examiner was that with the disposable elevator cap the fixation of the guidewire and use of the cholangioscope was easier. The cleaning was subjective more comfortable with SU cap.	
**MU endoscopes only**		
Kwakman et al., 2022 (FT)<br><br>The Netherlands<br><br>Dates of publications: 01/2008 to 12/2019<br><br>Trial registration: N/A	N/A	

[a] AB: conference abstract; FT – full-text article. [b] Patient turnover time was defined as time when patient out of room to the time the next patient in room; cases where the turnover was impacted by other issues such as intravenous access, or a late patient, or patient out of room delay due to patient status or post anaesthesia care unit delays were excluded.

AB: conference abstract; ERCP: endoscopic retrograde cholangiopancreatography; FT: full-text article; MU: multiple-use (endoscope); N/A: Not applicable; NR: not reported; PR: posted results; SU: single-use (endoscope); TRR: trial registry record.

2. Other upper gastrointestinal (UGI) endoscopes

2.1 Technical performance

No comparative evidence on the number of attempts to cannulate/intubate was reported.

Supplementary Table 12. Comparative evidence on successful intubation rate

Study, publication type, study design, study location & case complexity (Bold = DTA or RCT)	Findings	P value
SU vs MU		
**Sami et al. 2019, DTA**<br>**UK, USA**	EG Scan II (IntroMedic) vs GIF-H260 / GIF-H180 (Olympus)<br>***Successful intubation***<br>89% (178/200) vs 100% (200/200)	<0·001
**Huynh et al. 2017, DTA**<br>**Australia**	EG Scan II (IntroMedic) vs QF 180 (Olympus)<br>***Successful intubation***<br>100% (48/48) vs 100% (48/48)	NR
**Sami et al. 2016, DTA**<br>**UK**	EG Scan II (IntroMedic) vs GIF-H260 (Olympus)<br>***Successful intubation***<br>94% (47/50) vs 96% (48/50)	1·000*
Sheathed MU vs MU		
**Sami et al. 2015, RCT**<br>**USA**	EndoSheath® TNE – 5000 (Vision® Sciences) mobile unit vs EndoSheath® TNE – 5000 (Vision® Sciences) hospital outpatient endoscopy suite vs GIF-180 (Olympus)<br>***Successful intubation***<br>79% (60/76) vs 96% (69/72) vs 100% (61/61)	0·06

Abbreviations: DTA – diagnostic test accuracy; MU – multiple-use; N/A – not applicable; NR – not reported; NRCS – non-randomised comparative study; RCT - randomised controlled trial; SU – single-use;

Yellow = Full-text article, White = Abstract

*p-value was not reported and was calculated by review authors (Fisher's exact test).

Supplementary Table 13. Proportion of cross-over to MU endoscopes for SU endoscopes and success rates following cross-over

Study, publication type, study design, study location & case complexity (Bold = RCT)	Findings
van der Ploeg et al. 2023, case series<br><br>Netherlands, Norway	aScope Gastro (Ambu)<br>***Proportion crossed over to MU***: 3% (2/60)<br>***Successful intubation after crossover***: 100% (2/2)

Abbreviations: MU – multiple-use; SU – single-use; RCT - randomised controlled trial.

Yellow = Full-text article, White = Abstract

Supplementary Table 14. Comparative evidence on successful procedure completion for upper GI endoscopes (excluding successful cross-over)

Study, publication type, study design, study location & case complexity (Bold = RCT & fully paired DTA)	Findings	P value
**SU vs MU**		
Halland et al. 2020, DTA<br>USA	EG Scan II (IntroMedic)<br>Comparative evidence not reported	NR
**Sami et al. 2019, DTA<br>UK, USA**	EG Scan II (IntroMedic)<br>***Successful scan***<br>89% (178/200) vs 100% (200/200)	<0·001
**Huynh et al. 2017, DTA<br>Australia**	EG Scan II (IntroMedic)<br>***Successful gastroscopy***<br>100% (48/48) vs 100% (48/48)	NR
**Sami et al. 2016, DTA<br>UK**	EG Scan II (IntroMedic)<br>***Complete evaluation***<br>88% (44/50) vs 92% (46/50)	0·411
**Aedo et al. 2014, DTA<br>Mexico**	EG Scan I (IntroMedic) vs xGIF-1260 (Olympus)<br>***Successful evaluation***<br>100% (96/96) vs 100% (96/96)	NR
Römmele et al., 2024, RCT,<br>Germany	*SU gastroscope (detail NR)*<br>***Reaching the duodenum and adequate assessment***<br>100% (47/47) vs 100% (45/45)<br><br>***Successful haemostasis***<br>86% (19/22) vs 100% (24/24)	NR<br><br>0·101
**Luo et al. 2022, RCT<br>China**	XZING-W200B (Huizhou Xianzan)<br>***Endoscopy completion***<br>100% (55/55) vs 100%(55/55)	95% CI –6·5285% to 6·5285%*
Jin et al. 2015, NRCS<br>China	***Successful mucosal resection***<br>100% (28/28) vs 100% (26/26).	NR
**Sheathed MU vs MU**		
**Jin et al. 2014, RCT<br>China**	Modified GIF-V70 (Olympus), with SU GIF-V70-type sheath (Shenda)<br>***Successful gastroscopic examination***<br>100% (568/568) vs 100% (552/552).	NR

*Based on Newcombe-Wilson score method

Abbreviations: DTA – diagnostic test accuracy; MU – multiple-use; NR – not reported; RCT - randomised controlled trial; SU – single-use.

Yellow = Full-text article, White = Abstract

Supplementary Table 15. Technical outcomes – Defect rate: Other upper GI endoscopes

Author, year, country, publication type [a], dates of procedures, trial registration	Description of population, centre, single-use (SU) / sheathed multiple-use (MU) endoscope, average age (years), % female, indications	Description of multiple-use (MU) endoscope /, , study design	SU group vs MU group - Defect / device failure rate

**SU upper gastrointestinal (UGI) endoscopes:**<br>**EG Scan (IntroMedic), ultrathin transnasal capsule**			
Halland et al., 2020 (AB)<br><br>USA<br><br>Dates of procedures: NR<br><br>Trial registration: NR	Patients with and without Barrett's Oesophagus referred for endoscopy.<br>EG Scan II (IntroMedic)<br>Age: NR<br>Female: 40%<br>Main indications:<br>Barrett's Oesophagus: 33%.	MU endoscope (details NR)<br><br>DTA - N=54<br>(46 completed both)	N/A
Kang et al., 2019 (FT)<br><br>South Korea<br><br>Dates of procedures: 03/2016 to 07/2016<br><br>Trial registration: NR	Adult patients with gastrointestinal symptoms, or requiring screening endoscopy for gastric cancer at one hospital.<br>EG Scan III (IntroMedic).<br>Mean age (±SD): 43 (± 15)<br>Female: 59%<br>Main indications:<br>Dyspeptic symptoms: 59%,<br>Gastroesophageal reflux disease: 29%,<br>Screening endoscopy for gastric cancer: 12%.	GIF-H260 (Olympus), MU endoscope<br><br>DTA - N=50<br>(40 completed both)	N/A
Sami et al., 2019 (FT)<br><br>UK, USA<br><br>Dates of procedures: 07/2012 to 10/2015<br><br>ISRCTN 70595405 / NCT02066233	Patients with and without Barrett's oesophagus recruited from three referral centres.<br>EG Scan II (IntroMedic)<br>Mean age: 58<br>Female: 33%<br>Main indications: NR	GIF-260H / GIF-H180 (Olympus),<br>MU endoscope<br><br>DTA - N=200<br>(178 completed both)	Technical failure (probe):<br>2 (1%) vs N/A.
Huynh et al., 2017 (FT)<br><br>Australia<br><br>Dates of procedures: 09/2013 to 11/2014<br><br>ACTRN126160011034 59	Consecutive adult patients with liver cirrhosis requiring surveillance / screening of gastro-oesophageal varices in one institution.<br>EG Scan II (IntroMedic)<br>Mean age (± SD): 56 (± 1)<br>Female: 21%<br>Main indications:<br>Hepatitis C virus: 31%,<br>Ethanol-related liver disease: 27%.	QF 180 (Olympus),<br>MU gastroscope<br><br>DTA - N=48<br>(48 completed both)	N/A
Jin et al., 2017 (AB)<br><br>South Korea<br><br>Dates of procedures: NR<br><br>Trial registration: NCT03009383	Consecutive patients suspected of oesophageal foreign body.<br>EG Scan (version NR) (IntroMedic)<br>Median age (range): 56 (26 - 80)<br>Female: 30%<br>Main indications:<br>Odynophagia: 52%,<br>Dysphagia: 26%.	MU endoscope (details NR)<br><br>DTA - N=27<br>(25 completed both)	Technical failure:<br>2 (7%) vs N/A.

Sami et al., 2016 (FT)<br><br>UK<br><br>Dates of procedures: 07/2012 to 02/2014<br><br>Trial registration: ISRCTN: 70595- 405	Consecutive adult patients with cirrhosis, requiring surveillance / screening of oesophageal varices.<br>EG Scan II (IntroMedic)<br>Mean age: 59<br>Female: 27%<br>Main indications:<br>Oesophageal varices: 49%.	GIF-H260 (Olympus), MU endoscope<br><br>DTA - N=50<br>(45 completed both)	N/A
Eqbal et al., 2021 (FT)<br><br>Australia<br><br>Dates of procedures: NR<br><br>Trial registration: NR	Adult patients with cirrhosis requiring surveillance endoscopy in a regional tertiary centre.<br>EG Scan II, given trans-orally (N=40).<br>Median age (IQR): 60 (57–67)<br>Female: 20%<br>Main indications:<br>Hepatitis C: 50%,<br>Alcohol-related liver disease: 29%,<br>Non-alcoholic fatty liver disease: 20%.	N/A<br><br>Case series	N/A
Aedo et al., 2014 (FT)<br><br>Mexico<br><br>Dates of procedures: 11/2011 to 02/2012<br><br>Trial registration: NR	Consecutive adult patients referred for the evaluation of oesophageal disease<br>EG Scan I (IntroMedic)<br>Mean age (range): 50 (18 to 79)<br>Female: 56%<br>Main indications:<br>Reflux symptoms: 43%,<br>Suspect of oesophageal varices: 24%,<br>Epigastric pain: 15%,<br>Upper GI bleeding: 11%	XGIF-160 (Olympus)<br><br>DTA – N=96<br>(96 completed both)	N/A
Choi et al., 2014 (FT)<br><br>South Korea<br><br>Dates of procedures: 01/2012 to 09/2012<br><br>Trial registration: WHO ICTRP KCT0000298	Adult patients presenting with symptoms or signs of gastrointestinal bleeding (including melena, haematemesis, haematochezia, acute-onset anaemia).<br>EG Scan I (IntroMedic)<br>Mean age (±SD): 64 (±14)<br>Female: 29%<br>Main indications:<br>Melena: 36%,<br>Haematemesis: 33%,<br>Haematochezia: 22%,<br>Anaemia: 9%	MU endoscope (details NR)<br><br>DTA – N=129<br>(129 completed both)	N/A
Cho et al., 2013 (FT)<br><br>South Korea<br><br>Dates of procedures: 01/2011 to 03/2011<br><br>Trial registration: NR	Adult patients with clinical manifestations suspected of being acute GI bleeding.<br>EG Scan I (IntroMedic)<br>Mean age (range): 72 (47-89)<br>Female: 31%<br>Main indications:<br>Melena: 46%,<br>Haematemesis: 31%,<br>Haematochezia: 23%	MU endoscope (GIF-Q260)[c]<br><br>DTA – N=13<br>(13 completed both)	N/A
Chung et al., 2012 (FT)<br><br>South Korea	Adult patients referred for the evaluation of oesophageal diseases.	N/A	N/A

Dates of procedures: 10/2010 to 02/2011<br><br>Trial registration: NR	EG Scan I (IntroMedic) (n=50).<br>Mean age (±SD): 49 (±14)<br>Female: 52%<br>Main indications:<br>Known / suspected gastroesophageal reflux disease: 84%,<br>Known / suspected oesophageal varices: 16%		
**Other SU UGI endoscopes**			
Römmele et al., 2024 (AB)<br><br>Germany<br><br>Dates of procedures: 03/2023 to 11/2023<br><br>Trial registration: NCT06192355	Adult patients presenting with upper gastrointestinal bleeding in one medical centre.<br>SU gastroscope (details NR)(n=47) vs MU (n=45).<br>Mean age: 70<br>Female: 34 (37%)<br>Main indications:<br>Upper gastrointestinal bleeding: 100%.	MU endoscope (details NR) (n=45)<br><br>RCT	N/A
Wang et al., 2024 (AB)<br><br>China<br><br>Dates of procedures: 04/2023 to 06/2023<br><br>Trial registration: NR	Adult patients that underwent gastrointestinal endoscopy for gastrointestinal bleeding in one medical centre.<br>SU gastroscope (details NR) (n=21)<br>Age: NR vs NR (p>0.05)<br>Female: NR vs NR (p>0.05)<br>Main indications:<br>NR vs NR (p>0.05).	Pentax (model NR), MU gastroscope (n=21)<br><br>RCT	Equipment death rate:<br>(exact figures NR) (P>0·05).
Li et al., 2022 (FT)<br><br>China<br><br>Dates of procedures: 12/2019 to 06/2020<br><br>Trial registration: ChiCTR2000029945	Adult patients scheduled for upper gastrointestinal endoscopy in two hospitals.<br>PR-IPD002 (Shenzhen PengRui) (n=70)<br>Mean age (±SD): 44 (±11) vs 43 (±10)<br>Female: 66% vs 61%<br>Main indications:<br>Abdominal distension: 36% vs 33%,<br>Abdominal pain: 33% vs 35%,<br>Screening: 21% vs 23%.	GIF-260 (Olympus), MU endoscope (n=69)<br><br>RCT	N/A
Luo et al., 2022 (FT)<br><br>China<br><br>Dates of procedures: 06/2020 to 12/2020<br><br>Trial registration: NR	Adult patients scheduled for gastroscopy in two hospitals.<br>XZING-W200B (Huizhou Xianzan) (n=55)<br>Mean age (±SD): 37 (40±13) vs 39 (43±14)<br>Female: 64% vs 67%<br>Main indications: NR	GIF-HQ290 and GIF-H290 (Olympus),<br>MU gastroscope (n=55)<br><br>RCT	Technical failure:<br>0 (0%) vs 0 (0%).
Dowds et al., 2024 (AB)<br><br>USA<br><br>Dates of procedures: 12/2022 to 11/2023	Paediatric patients scheduled for follow up evaluation for eosinophilic esophagitis in a children's hospital.<br>Gastroscope (EvoEndo, model NR) (N=78).<br>Age (range): 8-21	N/A<br><br>Case series	N/A

Trial registration: NR	Female: NR<br>Main indications:<br>Eosinophilic esophagitis: 100%.		
Lee et al., 2024 (AB)<br><br>USA<br><br>Dates of procedures: 09/2022 to 11/2023<br><br>Trial registration: NR	Paediatric patients with eosinophilic esophagitis in a paediatric department scheduled for transnasal endoscopy.<br>Gastroscope (EvoEndo, model NR) (N=49).<br>Median age (range): 14 (7-20)<br>Female: NR<br>Main indications:<br>Eosinophilic esophagitis: 100%.	N/A<br><br>Case series	N/A
van der Ploeg et al., 2023 (FT)<br><br>Netherlands, Norway<br><br>Dates of procedures: NR<br><br>Trial registration: NCT05504434	Adult patients scheduled for esophagogastroduodenoscopy in an endoscopy dept, and intensive care unit.<br>aScope Gastro (Ambu) (N=60).<br>Mean age (range): 62 (54-74)<br>Female: 27%<br>Main indications:<br>Suspected gastrointestinal bleeding: 18%,<br>Nutritional tube placement: 17%,<br>Treatment oesophageal stricture: 15%.	N/A<br><br>Case series	Device failure: 0 (0%).
**Sheathed MU endoscopes**			
Shariff et al., 2016 (FT)<br><br>UK<br><br>Dates of procedures: NR<br><br>Trial registration: NR	Consecutive adult patients scheduled for diagnostic endoscopy in tertiary referral centre.<br>Ultrathin Transnasal EndoSheath® TNE – 5000 (Vision® Sciences).<br>Median age (range): 59 (24-76)<br>Female: 40%<br>Main indications:<br>Included dyspepsia, anaemia, suspected coeliac disease and abdominal pain, Barrett's Oesophagus with a length ≥2 cm (% NR).	GIF-Q240Z (Olympus), MU endoscope<br><br>DTA - N=25 (21 completed both)	N/A
Sami et al., 2015 (FT)<br><br>USA<br><br>Dates of procedures: 03/2011 to 10/2013<br><br>Trial registration: NCT01288612	Patients identified by reported frequency of reflux symptoms, consented to screen in two medical centres.<br>Ultrathin Transnasal EndoSheath® TNE – 5000 (Vision® Sciences).<br>TNE – 5000 (mobile unit) (n=76) vs TNE – 5000 (hospital outpatient endoscopy suite) (n=72) vs MU (n=61).<br>Mean age (SD): 65 (± 9) vs 64 (± 9) vs 66 (± 9)	GIF-180 (Olympus), MU (n=61)<br><br>RCT	N/A

	Female: 58% vs 51% vs 53%<br>Main indications:<br>Reflux symptoms ≥1/week: 15% vs 14% vs 13%, Reflux symptoms <1/week: 34% vs 44% vs 46%.		
Jin et al., 2014 (FT)<br><br>China<br><br>Dates of procedures: 07/2010 to 09/2011<br><br>Trial registration: NR	Adult patients needing gastroscopic examinations.<br>Modified GIF-V70 (Olympus) with SU GIF-V70-type sheath (Shenda) (n=568). vs GIF-V70 (n=552).<br>Mean age (±SD): 35 (±11) vs 35 (±10)<br>Female: 10% vs 9%<br>Main indications: NR	GIF-V70 (Olympus), MU gastroscope (n=552)<br><br>RCT	N/A
Jin et al., 2015 (AB)<br><br>China<br><br>Dates of procedures: NR<br><br>Trial registration: NR	Adult patients with gastric hyperplastic polyps needing endoscopic mucosal resection in military general hospital.<br>Sheathed MU gastroscope (Shenda, model NR) (n=28) vs MU gastroscope (n=26).<br>Age: NR<br>Female: NR<br>Main indications: NR	MU gastroscope (details NR) (n=26)<br><br>Non-randomised comparative study	N/A
Streckfuss et al., 2014 (FT)<br><br>Germany<br><br>Dates of procedures: NR<br><br>Trial registration: NR	Patients with throat complaints including dysphagia, globus pharyngeus, and dysphonia, given screening esophagoscopy for gastroesophageal reflux and laryngopharyngeal reflux in laryngological clinic.<br>Ultrathin Transnasal EndoSheath® TNE – 5000 (Vision® Sciences), with SU TV-2.1 sheath (Vision® Sciences) (n=55)<br>.<br>Mean age (±SD): 53 (±16)<br>Female: 36%<br>Main indications:<br>Dysphagia: 24%,<br>Globus pharyngeus: 15%,<br>Heartburn: 15%,<br>Chronic throat clearing / cough: 13%,<br>Dysphonia: 13%,<br>Epigastric soreness: 13%.	Case series	N/A
Peery et al., 2011 (AB)<br><br>USA<br><br>Dates of procedures: NR<br><br>Trial registration: NR	Patients from primary care population in two outpatient centres.<br>Sheathed MU Ultrathin Transnasal EndoSheath® TNE – 5000 (Vision® Sciences) (n=264).<br>Mean age (±SD): 55 (±9)<br>Female: 59%<br>Main indications: NR	Case series	N/A

[a] AB - conference abstract; FT – full-text article; [b] the study also included a pilot RCT (n=20) examining microbial contamination, which is not reported her; [c] MU endoscope was not described, but GIF-Q260 was mentioned in the image shown in the article for conventional esophagogastroduodenoscopy. AB: conference abstract; CI: confidence interval; DTA: diagnostic test accuracy study; FT: full-text article; GI: gastrointestinal; MU: multiple-use (endoscope); N/A: not applicable; NR: not reported; RCT - randomised controlled trial; SD: standard deviation; SU: single-use (endoscope); UGI: upper gastrointestinal.

Supplementary Table 16. Technical outcomes – Image quality: Other upper GI endoscopes

Author, year, country, publication type [a], dates of procedures, trial registration	SU group vs MU group - Image quality	SU group vs MU group - Successful visualisation
**SU upper gastrointestinal (UGI) endoscopes: EG Scan (IntroMedic), ultrathin transnasal capsule**		
Halland et al., 2020 (AB)<br><br>USA<br><br>Dates of procedures: NR<br><br>Trial registration: NR	N/A	N/A
Kang et al., 2019 (FT)<br><br>South Korea<br><br>Dates of procedures: 03/2016 to 07/2016<br><br>Trial registration: NR	1 Worst – 9 Best<br>Mean:<br><br>Visualisation in oesphagogastric junction: 7 vs N/A<br><br>Visualisation in the cardia: 4 vs N/A,<br>Visualisation at an angle: 5 vs N/A,<br>Visualisation in the antrum: 4 vs N/A,<br>Visualisation in the duodenum: 1 vs N/A.	N/A
Sami et al., 2019 (FT)<br><br>UK, USA<br><br>Dates of procedures: 07/2012 to 10/2015<br><br>ISRCTN 70595405 / NCT02066233	N/A	Complete visualisation:<br>175 (98%) vs<br>177 (99%) (p=0·3146).
Huynh et al., 2017 (FT)<br><br>Australia<br><br>Dates of procedures: 09/2013 to 11/2014<br><br>ACTRN12616001103459	N/A	N/A
Jin et al., 2017 (AB)<br><br>South Korea<br><br>Dates of procedures: NR<br><br>Trial registration: NCT03009383	N/A	N/A
Sami et al., 2016 (FT)<br><br>UK	N/A	Visualisation of the whole oesophagus and identification of the landmarks:<br>44 (88%) vs<br>46 (92%) (p=0·411).

Dates of procedures:<br>07/2012 to 02/2014<br><br>Trial registration: ISRCTN: 70595- 405		
Eqbal et al., 2021 (FT)<br><br>Australia<br><br>Dates of procedures: NR<br><br>Trial registration: NR	N/A	N/A
Aedo et al., 2014 (FT)<br><br>Mexico<br><br>Dates of procedures:<br>11/2011 to 02/2012<br><br>Trial registration: NR	N/A	Visualisation of pylorus:<br><br>41 (43%) vs NR.<br><br>Identification of the landmark (duodenum):<br><br>35 (36%) vs NR.<br><br>Percentage of the oesophagogastric junction appropriately evaluated (i,e., ≥ 75 % visualised)<br><br>91 (95%) vs NR.
Choi et al., 2014 (FT)<br><br>South Korea<br><br>Dates of procedures:<br>01/2012 to 09/2012<br><br>Trial registration: WHO ICTRP KCT0000298	N/A	N/A
Cho et al., 2013 (FT)<br><br>South Korea<br><br>Dates of procedures:<br>01/2011 to 03/2011<br><br>Trial registration: NR	N/A	N/A
Chung et al., 2012 (FT)<br><br>South Korea<br><br>Dates of procedures:<br>10/2010 to 02/2011<br><br>Trial registration: NR	N/A	Percentage of the z-line successfully visualised<br><br>100% 8 (17%)<br>≥ 75 % 17 (37%)<br>50 %– 75 % 13 (28%)<br>< 50 % 8 (17%)
**Other SU UGI endoscopes**		
Römmele et al., 2024 (AB)<br><br>Germany<br><br>Dates of procedures:<br>03/2023 to 11/2023<br><br>Trial registration: NCT06192355	N/A	
Wang et al., 2024 (AB)<br><br>China<br><br>Dates of procedures:<br>04/2023 to 06/2023<br><br>Trial registration:<br>NR	Image quality score:<br>NR vs NR (P>0·05).	
Li et al., 2022 (FT)<br><br>China	Image quality rated 'good' or 'excellent': 98% vs 100% (p<0·99).	N/A

Dates of procedures: 12/2019 to 06/2020<br><br>Trial registration: ChiCTR2000029945		
Luo et al., 2022 (FT)<br><br>China<br><br>Dates of procedures: 06/2020 to 12/2020<br><br>Trial registration: NR	Image quality rated ‘acceptable’: 55 (100%) vs 55 (100%) (95% CI, 0·9347-1·0000).<br><br>(Further detail in Table 4.)	N/A
Dowds et al., 2024 (AB)<br><br>USA<br><br>Dates of procedures: 12/2022 to 11/2023<br><br>Trial registration: NR	N/A	N/A
Lee et al., 2024 (AB)<br><br>USA<br><br>Dates of procedures: 09/2022 to 11/2023<br><br>Trial registration: NR	N/A	N/A
van der Ploeg et al., 2023 (FT)<br><br>Netherlands, Norway<br><br>Dates of procedures: NR<br><br>Trial registration: NCT05504434	1 Much worse – 5 Much better (than reusable gastroscope)<br><br>Image size, Image stability, Distant focus, Close focus, Lighting, and Overall picture and video quality<br><br>(See Figure 2 for further detail, divided by research site.	N/A
**Sheathed MU endoscopes**		
Shariff et al., 2016 (FT)<br><br>UK<br><br>Dates of procedures: NR<br><br>Trial registration: NR	0 Poor – 10 Excellent<br>Mean (standard error of mean):<br><br>Optical quality: 4 (±0·5) vs 7 (±0·5)(p=<0·0001).	N/A
Sami et al., 2015 (FT)<br><br>USA<br><br>Dates of procedures: 03/2011 to 10/2013<br><br>Trial registration: NCT01288612	N/A	Visualisation of the whole oesophagus and identification of the landmarks:<br>75 (99%) vs<br>69 (96%) vs<br>61 (100%) (p=0·08). [b]
Jin et al., 2014 (FT) [c]<br><br>China<br><br>Dates of procedures: 07/2010 to 09/2011<br><br>Trial registration: NR	N/A	N/A
Jin et al., 2015 (AB)<br><br>China<br><br>Dates of procedures: NR<br><br>Trial registration: NR	N/A	N/A
Streckfuss et al., 2014 (FT)<br><br>Germany		

Dates of procedures: NR<br><br>Trial registration: NR	N/A	N/A
Peery et al., 2011 (AB)<br><br>USA<br><br>Dates of procedures: NR<br><br>Trial registration: NR	N/A	N/A

[a] AB - conference abstract; FT – full-text article; [b] P values for this study from comparisons between three groups. [c] The study also included a pilot RCT (n=20) examining microbial contamination, which is not reported here.

AB: conference abstract;, FT: full-text article; MU: multiple-use (endoscope); N/A: not applicable; NR: not reported; RCT - randomised controlled trial; SU: single-use (endoscope); UGI: upper gastrointestinal.

Supplementary Table 17. Technical outcomes – Operator rating: Other upper GI endoscopes

Author, year, country, publication type [a], dates of procedures, trial registration	SU group vs MU group - Manoeuvres successful	SU group vs MU group - Operator rating: manoeuvres	SU group vs MU group - Operator rating: performance characteristics	SU group vs MU group - Operator rating: overall satisfaction
**SU upper gastrointestinal (UGI) endoscopes: EG Scan (IntroMedic), ultrathin transnasal capsule**				
Halland et al., 2020 (AB)<br><br>USA<br><br>Dates of procedures: NR<br><br>Trial registration: NR	N/A	N/A	N/A	N/A
Kang et al., 2019 (FT)<br><br>South Korea<br><br>Dates of procedures: 03/2016 to 07/2016<br><br>Trial registration: NR	N/A	Examination rated 'adequate':<br><br>41 (84%) vs<br><br>N/A (Data NR).	1 Worst – 9 Best<br><br>(Mean/median NR)<br><br>Insertion into oesophagus: 8 vs N/A,<br><br>Insertion into oesophago-gastric junction: 8 vs N/A<br><br>Handling in oesophagus: 8 vs N/A,<br><br>Insertion into pyloric channel: 2 vs N/A,<br><br>Handling in axis rotation: 5 vs N/A,	N/A

Author, year, country, publication type [a], dates of procedures, trial registration	SU group vs MU group - Manoeuvres successful	SU group vs MU group - Operator rating: manoeuvres	SU group vs MU group - Operator rating: performance characteristics	SU group vs MU group - Operator rating: overall satisfaction
			Handling in J turn: 5 vs N/A<br><br>Handling in duodenum: 1 vs N/A.	
Sami et al., 2019 (FT)<br><br>UK, USA<br><br>Dates of procedures: 07/2012 to 10/2015<br><br>ISRCTN 70595405 /<br><br>NCT02066233	N/A	N/A	N/A	N/A
Huynh et al., 2017 (FT)<br><br>Australia<br><br>Dates of procedures: 09/2013 to 11/2014<br><br>ACTRN12616001103459	N/A	N/A	N/A	N/A
Jin et al., 2017 (AB)<br><br>South Korea<br><br>Dates of procedures: NR<br><br>Trial registration: NCT03009383	N/A	N/A	N/A	N/A
Sami et al., 2016 (FT)<br><br>UK<br><br>Dates of procedures: 07/2012 to 02/2014<br><br>Trial registration: ISRCTN: 70595- 405	N/A	N/A	N/A	N/A
Eqbal et al., 2021 (FT)	N/A	N/A	N/A	N/A

Author, year, country, publication type [a], dates of procedures, trial registration	SU group vs MU group - Manoeuvres successful	SU group vs MU group - Operator rating: manoeuvres	SU group vs MU group - Operator rating: performance characteristics	SU group vs MU group - Operator rating: overall satisfaction
Australia<br><br>Dates of procedures: NR<br><br>Trial registration: NR				
Aedo et al., 2014 (FT)<br><br>Mexico<br><br>Dates of procedures: 11/2011 to 02/2012<br><br>Trial registration: NR	N/A	N/A	N/A	N/A
Choi et al., 2014 (FT)<br><br>South Korea<br><br>Dates of procedures: 01/2012 to 09/2012<br><br>Trial registration: WHO ICTRP KCT0000298	Inserted into stomach: 128/129 (99%) vs NR.<br><br>Inserted into duodenum: 4/129 (3%) vs NR.	N/A	N/A	N/A
Cho et al., 2013 (FT)<br><br>South Korea<br><br>Dates of procedures: 01/2011 to 03/2011<br><br>Trial registration: NR	N/A	N/A	N/A	N/A
Chung et al., 2012 (FT)<br><br>South Korea<br><br>Dates of procedures: 10/2010 to 02/2011<br><br>Trial registration: NR	N/A	N/A	N/A	N/A
**Other SU UGI endoscopes**				
Römmele et al., 2024 (AB)<br><br>Germany<br><br>Dates of procedures: 03/2023 to 11/2023<br><br>Trial registration: NCT06192355	N/A	N/A	N/A	N/A
Wang et al., 2024 (AB)<br><br>China<br><br>Dates of procedures:	N/A	N/A	Clinical operability: NR vs NR (P>0·05).	N/A

Author, year, country, publication type [a], dates of procedures, trial registration	SU group vs MU group - Manoeuvres successful	SU group vs MU group - Operator rating: manoeuvres	SU group vs MU group - Operator rating: performance characteristics	SU group vs MU group - Operator rating: overall satisfaction
04/2023 to 06/2023<br><br>Trial registration: NR				
Li et al., 2022 (FT)<br><br>China<br><br>Dates of procedures: 12/2019 to 06/2020<br><br>Trial registration:<br><br>ChiCTR2000029945	N/A	N/A	Manoeuvrability satisfaction: 98% vs 100% (p<0·99).	N/A
Luo et al., 2022 (FT)<br><br>China<br><br>Dates of procedures: 06/2020 to 12/2020<br><br>Trial registration: NR	N/A	N/A	Clinical operability rated 'acceptable':<br><br>55 (100%) vs 55 (100%)(CI, 0·9347-1.0000),<br><br>flexibility (body<br><br>rigidity, knob operation, and sharp angle adaptability): 55 (100%) vs 55 (100%),<br><br>(Further detail in Table 5.)	N/A
Dowds et al., 2024 (AB)<br><br>USA<br><br>Dates of procedures: 12/2022 to 11/2023<br><br>Trial registration: NR	N/A	N/A	N/A	N/A
Lee et al., 2024 (AB)<br><br>USA<br><br>Dates of procedures: 09/2022 to 11/2023<br><br>Trial registration: NR	N/A	N/A	N/A	N/A
van der Ploeg et al., 2023 (FT)<br><br>Netherlands, Norway<br><br>Dates of procedures: NR<br><br>Trial registration: NCT05504434	N/A	N/A	1 Much worse – 5 Much better (than reusable gastroscope)<br><br>(See Figure 2 for further detail, divided by research site.	Overall satisfaction<br><br>Comparable to reusable scopes: 51 (91%),<br><br>Inferior to reusable scopes: 5 (9%).

Author, year, country, publication type [a], dates of procedures, trial registration	SU group vs MU group - Manoeuvres successful	SU group vs MU group - Operator rating: manoeuvres	SU group vs MU group - Operator rating: performance characteristics	SU group vs MU group - Operator rating: overall satisfaction
**Sheathed MU endoscopes**				
Shariff et al., 2016 (FT)<br>UK<br>Dates of procedures: NR<br>Trial registration: NR	N/A	N/A	N/A	N/A
Sami et al., 2015 (FT)<br>USA<br>Dates of procedures: 03/2011 to 10/2013<br>Trial registration: NCT01288612	N/A	N/A	N/A	N/A
Jin et al., 2014 (FT) [b]<br>China<br>Dates of procedures: 07/2010 to 09/2011<br>Trial registration: NR	N/A	N/A	N/A	N/A
Jin et al., 2015 (AB)<br>China<br>Dates of procedures: NR<br>Trial registration: NR	N/A	N/A	N/A	N/A
Streckfuss et al., 2014 (FT)<br>Germany<br>Dates of procedures: NR<br>Trial registration: NR	N/A	N/A	N/A	N/A
Peery et al., 2011 (AB)<br>USA<br>Dates of procedures: NR<br>Trial registration: NR	N/A	N/A	N/A	N/A

[a] AB - conference abstract; FT – full-text article; [b] The study also included a pilot RCT (n=20) examining microbial contamination, which is not reported here.

AB: conference abstract; CI: confidence Interval; FT: full-text article; IQR: interquartile range; MU: multiple-use (endoscope); N/A: not applicable; NR: not reported; RCT - randomised controlled trial; SU: single-use (endoscope); UGI: upper gastrointestinal.

Supplementary Table 18. Technical outcomes – Duration: Other upper GI endoscopes

Author, year, country, publication type [a], dates of procedures, trial registration	SU group vs MU group - Time to landmarks / cannulate / intubate	SU group vs MU group - Time for procedure	SU group vs MU group - Time for (pre-) cleaning / discarding / reprocessing instruments / turning around room / discharging patient / transportation
**SU upper gastrointestinal (UGI) endoscopes: EG Scan (IntroMedic), ultrathin transnasal capsule**			
Halland et al., 2020 (AB)<br>USA<br>Dates of procedures: NR<br>Trial registration: NR	N/A	N/A	N/A
Kang et al., 2019 (FT)<br>South Korea<br>Dates of procedures: 03/2016 to 07/2016<br>Trial registration: NR	N/A	N/A	N/A
Sami et al., 2019 (FT)<br>UK, USA<br>Dates of procedures: 07/2012 to 10/2015<br>ISRCTN 70595405 / NCT02066233	N/A	Mean (SD) minutes:<br>4 (±4) vs N/A.	N/A
Huynh et al., 2017 (FT)<br>Australia<br>Dates of procedures: 09/2013 to 11/2014<br>ACTRN12616001103459	N/A	N/A	N/A

Author, year, country, publication type [a], dates of procedures, trial registration	SU group vs MU group - Time to landmarks / cannulate / intubate	SU group vs MU group - Time for procedure	SU group vs MU group - Time for (pre-) cleaning / discarding / reprocessing instruments / turning around room / discharging patient / transportation
Jin et al., 2017 (AB)<br><br>South Korea<br><br>Dates of procedures: NR<br><br>Trial registration: NCT03009383	N/A	N/A	N/A
Sami et al., 2016 (FT)<br><br>UK<br><br>Dates of procedures: 07/2012 to 02/2014<br><br>Trial registration: ISRCTN: 70595-405	N/A	Mean (SD) minutes:<br>8 (±2) vs N/A.	N/A
Eqbal et al., 2021 (FT)<br><br>Australia<br><br>Dates of procedures: NR<br><br>Trial registration: NR	N/A	Median (IQR) minutes:<br>2 (1-3).	N/A
Aedo et al., 2014 (FT)<br><br>Mexico<br><br>Dates of procedures: 11/2011 to 02/2012<br><br>Trial registration: NR	N/A	Mean (range) minutes:<br>5 (3-8) vs NR.	N/A
Choi et al., 2014 (FT)<br><br>South Korea<br><br>Dates of procedures: 01/2012 to 09/2012<br><br>Trial registration: WHO ICTRP KCT0000298	N/A	Mean (range) minutes:<br>5 (± 2) vs NR.	N/A
Cho et al., 2013 (FT)<br><br>South Korea<br><br>Dates of procedures: 01/2011 to 03/2011<br><br>Trial registration: NR	N/A	N/A	N/A
Chung et al., 2012 (FT)<br><br>South Korea<br><br>Dates of procedures: 10/2010 to 02/2011<br><br>Trial registration: NR	N/A	N/A	N/A
**Other SU UGI endoscopes**			

Author, year, country, publication type [a], dates of procedures, trial registration	SU group vs MU group - Time to landmarks / cannulate / intubate	SU group vs MU group - Time for procedure	SU group vs MU group - Time for (pre-) cleaning / discarding / reprocessing instruments / turning around room / discharging patient / transportation
Römmele et al., 2024 (AB)<br><br>Germany<br><br>Dates of procedures:<br>03/2023 to 11/2023<br><br>Trial registration: NCT06192355	N/A	NR	Length of stay:<br>NR
Wang et al., 2024 (AB)<br><br>China<br><br>Dates of procedures:<br>04/2023 to 06/2023<br><br>Trial registration:<br>NR	N/A	Operation time:<br>NR	Time for equipment preparation:<br>NR vs NR<br>SU shorter (P<0·05).<br><br>Time for transportation:<br>NR vs NR<br>SU shorter<br>(P<0·05).<br><br>Time for decontamination:<br>0 vs NR<br>SU shorter
Li et al., 2022 (FT)<br><br>China<br><br>Dates of procedures:<br>12/2019 to 06/2020<br><br>Trial registration:<br>ChiCTR2000029945	N/A	Operating time<br>Mean (± SD) minutes:<br>8 (±4) vs 5 (±3)<br>(p<0·001).	N/A
Luo et al., 2022 (FT)<br><br>China<br><br>Dates of procedures:<br>06/2020 to 12/2020<br><br>Trial registration: NR	Insertion time<br>Mean (± SD) minutes:<br>4 (±3) vs 2 (±1)<br>(p<0·001).<br><br>Withdrawal time<br>Mean (± SD) minutes:<br>4 (±2) vs 3 (±1)<br>(p<0·001).	Mean (SD) minutes:<br>8 (±4) vs 5 (±2)<br>(p=0·000).	N/A
Dowds et al., 2024 (AB)<br><br>USA<br><br>Dates of procedures:<br>12/2022 to 11/2023<br><br>Trial registration: NR	N/A	N/A	N/A
Lee et al., 2024 (AB)<br><br>USA<br><br>Dates of procedures:<br>09/2022 to 11/2023<br><br>Trial registration: NR	N/A	Mean minutes:<br>7.	Check in until discharge:<br>Mean minutes:<br>70).

Author, year, country, publication type [a], dates of procedures, trial registration	SU group vs MU group - Time to landmarks / cannulate / intubate	SU group vs MU group - Time for procedure	SU group vs MU group - Time for (pre-) cleaning / discarding / reprocessing instruments / turning around room / discharging patient / transportation
van der Ploeg et al., 2023 (FT)<br><br>Netherlands, Norway<br><br>Dates of procedures: NR<br><br>Trial registration: NCT05504434	N/A	Median (Q1, Q3) minutes: 10 (6, 5).	N/A
**Sheathed MU endoscopes**			
Shariff et al., 2016 (FT)<br><br>UK<br><br>Dates of procedures: NR<br><br>Trial registration: NR	N/A	N/A	N/A
Sami et al., 2015 (FT)<br><br>USA<br><br>Dates of procedures: 03/2011 to 10/2013<br><br>Trial registration: NCT01288612	N/A	Mean (SD) minutes:<br>9 (3) vs 8 (3) vs 9 (2) (p=<0·001). [b]	Recovery time (from extubation to discharge):<br>Mean (SD) minutes:<br>16 (±3) vs 19 (±4) vs 67 (±6) (p=<0·001). [b]
Jin et al., 2014 (FT) [c]<br><br>China<br><br>Dates of procedures: 07/2010 to 09/2011<br><br>Trial registration: NR	N/A	Mean (SD) minutes:<br>5 (±1) vs 4 (±1) (p=0·000).	Reprocessing time:<br>Mean (SD) minutes:<br>5 (±0·5) vs 35 (±0·5) (p=<0·000).<br><br>Instrument turnaround time:<br>Mean (SD) minutes:<br>10 (±1) vs 4 (±1) (p=<0·000).
Jin et al., 2015 (AB)<br><br>China<br><br>Dates of procedures: NR<br><br>Trial registration: NR	N/A	Mean (± SD) minutes:<br>9 (±2) vs 8 (±2) (p=0·000).	Reprocessing time:<br>Mean (± SD) minutes:<br>5 (±0·5) vs 35 (±0·5) (p=<0·000).
Streckfuss et al., 2014 (FT)<br><br>Germany<br><br>Dates of procedures: NR<br><br>Trial registration: NR	N/A	Time for examination:<br>Mean minutes: 11.<br><br>Time for whole procedure:<br>Mean minutes: 25.	Time for preparation of patient and endoscope:<br>Mean minutes:<br>8.<br>Time for cleaning endoscope:<br>Mean minutes:<br>5.

Author, year, country, publication type [a], dates of procedures, trial registration	SU group vs MU group - Time to landmarks / cannulate / intubate	SU group vs MU group - Time for procedure	SU group vs MU group - Time for (pre-) cleaning / discarding / reprocessing instruments / turning around room / discharging patient / transportation
Peery et al., 2011 (AB)<br><br>USA<br><br>Dates of procedures: NR<br><br>Trial registration: NR	N/A	Mean (±SD) minutes:<br><br>4 (±2).	N/A

AB: conference abstract; BO: Barrett's oesophagus; FT: full-text article; IQR: interquartile range; MU: multiple-use (endoscope); N/A: not applicable; NR: not reported; Q1 first quartile; Q3: third quartile; SD: standard deviation; RCT - randomised controlled trial; SU: single-use (endoscope); UGI: upper gastrointestinal.

[a] AB - conference abstract; FT – full-text article; [b] P values for this study from comparisons between three groups. [c] The study also included a pilot RCT (n=20) examining microbial contamination, which is not reported here.

2.2 Test accuracy

Supplementary Table 19. Diagnostic accuracy: Other upper gastrointestinal (UGI) endoscopes

<table>
<tr><th>Author, year, country, publication type [a], dates of procedures, trial registration</th><th>Diagnostic accuracy</th></tr>
<tr><td colspan="2">SU upper gastrointestinal (UGI) endoscopes:
EG Scan (IntroMedic), ultrathin transnasal capsule</td></tr>
<tr><td>Halland et al., 2020 (AB)

USA

Dates of procedures: NR

Trial registration: NR</td><td>
Sensitivity
BO 73·3% (95% CI 44·9% to 92·0%)
Esophagitis 50% (95% CI 16·1% to 84.0%)
Specificity
BO 87.1% (95% CI 70.1% to 96.3%)
Esophagitis 89·5% (95% CI 75·2% to 97·0%)
PPV
BO 11/15
Esophagitis 4/8
NPV
BO 27/31
Esophagitis 34/38
<table>
<tr><th></th><th>MU – BO present</th><th>MU – BO absent</th><th>Total</th></tr>
<tr><td>SU – BO Present</td><td>11</td><td>4</td><td>15</td></tr>
<tr><td>SU – BO Absent</td><td>4</td><td>27</td><td>31</td></tr>
<tr><td>Total</td><td>15</td><td>31</td><td>46</td></tr>
</table>
<table>
<tr><th></th><th>MU – Esophagitis Present</th><th>MU – Esophagitis Absent</th><th>Total</th></tr>
<tr><td>SU – Esophagitis Present</td><td>4</td><td>4</td><td>8</td></tr>
<tr><td>SU – Esophagitis Absent</td><td>4</td><td>34</td><td>38</td></tr>
<tr><td>Total</td><td>8</td><td>38</td><td>46</td></tr>
</table>
</td></tr>
<tr><td>Kang et al., 2019 (FT)

South Korea

Dates of procedures: 03/2016 to 07/2016

Trial registration: NR</td><td>
<table>
<tr><th>For SU</th><th>Patients n (%)</th><th>Sensitivity</th><th>Specificity</th><th>PPV</th><th>NPV</th></tr>
<tr><td>Oesophagus Hiatal hernia</td><td>18 (37%)</td><td>0·889 (0·653–0.986)</td><td>1·000 (0·888-1·000)</td><td>1·000 (0·794-1·000)</td><td>0·939 (0·798-0·993)</td></tr>
<tr><td>Reflux esophagitis</td><td>14 (29%)</td><td>0·929 (0·661–0·998)</td><td>1·000 (0.900–1·000)</td><td>1·000 (0·753–1·000)</td><td>0·972 (0·855–0·999)</td></tr>
<tr><td>Barrett's oesophagus</td><td>5 (10%)</td><td>1·000 (0·478–1·000)</td><td>1·000 (0·920–1·000)</td><td>1·000 (0·478–1·000)</td><td>1·000 (0·920–1·000)</td></tr>
<tr><td>Stomach Erosive gastritis</td><td>13 (27%)</td><td>0·846 (0·54 –0·981)</td><td>0·861 (0·705–0.953)</td><td>0·688 (0·413–0·890)</td><td>0·939 (0·798–0·993)</td></tr>
<tr><td>Gastric ulcer</td><td>6 (12%)</td><td>0·500 (0·118–0·882)</td><td>0·791 (0·640–0·900)</td><td>0·250 (0·055–0·572)</td><td>0·919 (0·781–0·983)</td></tr>
</table>
</td></tr>
</table>

Gastric tumour	2 (4%)	0·000 (0·000–0·842)	0·702 (0·551-0·827)	0·000 (0·000-0·232)	0·943 (0·808-0·993)

Sami et al., 2019 (FT)<br><br>UK, USA<br><br>Dates of procedures: 07/2012 to 10/2015<br><br>ISRCTN 70595405 / NCT02066233	**SU**<br>**Sensitivity**<br>Any length BO 0·90 (0·83–0·96)<br>Long-segment BO 0·95 (0·83–0·99)<br>Short-segment BO 0·87 (0·75–0·95)<br>**Specificity**<br>Any length BO 0·91 (0·82–0·96)<br>Long-segment BO 1.0 (0·96–1·00)<br>Short-segment BO 0·91 (0·82–0·96) (as reported by original authors; 0.94 based on re-constructed 2x2 table)<br>**AUROC**<br>Any length BO 0·91 (0·86–0·95)<br>Long-segment BO 0·98 (0·94–1·0)<br>Short-segment BO 0·89 (0·83–0·94)<br>**PPV**<br>0·92<br>**NPV**<br>0·90
Huynh et al., 2017 (FT)<br><br>Australia<br><br>Dates of procedures: 09/2013 to 11/2014<br><br>ACTRN12616001103 459	**SU**<br>**Sensitivity**<br>Any varices 25/26 (96%)<br>Large varices 9/10 (90%)<br>High risk oesophageal varices (HROV) 10/10 (100%)<br>**Specificity**<br>Any varices 86%<br>Accuracy 92%<br>Large varices 100%<br>Accuracy 98%<br>HROV 100%<br>Accuracy 100%<br>**PPV**<br>Any varices 89%<br>Large varices 100%<br>HROV 100%<br>**NPV**<br>Any varices 95%<br>Large varices 97%<br>HROV 100%<br>**Kappa**<br>Any varices 0·83<br>Large varices 0·93<br>HROV 1·0<br>**Video recordings (interobserver agreement)**<br>Any gastroesophageal varices 0·74<br>Large varices 0·94<br>HROV 0·82<br><br>**MU**<br>Oesophageal varices 26 patients (54%).<br>Of the 26,10 (21%) were HROV. 16 (33%) were small low-risk varices.<br>25 (96%) were confirmed on SU.
Jin et al., 2017 (AB)<br><br>South Korea<br><br>Dates of procedures: NR<br><br>Trial registration: NCT03009383	**SU**<br>Any size OFB<br>Sensitivity**:** 91%<br>Specificity: 100%<br>Accuracy: 92%<br>PPV: 100%<br>NPV 60%<br><br>Patients with FB > 10 mm in size<br>Sensitivity: 95%

	Specificity: 100%<br>Accuracy: 96%<br>PPV: 100%<br>NPV: 75%
Sami et al., 2016 (FT)<br><br>UK<br><br>Dates of procedures: 07/2012 to 02/2014<br><br>Trial registration: ISRCTN: 70595- 405	**Sensitivity**<br>Diagnosis of any size OV<br>0·82 (95% confidence interval (CI) 0·60–0·95).<br>Diagnosis of medium / large OV<br>0·92 (95% CI 0·62–1·0).<br>**Specificity**<br>Diagnosis of any OV<br>0·78 (95% CI 0·56–0·93).<br>Diagnosis of medium/large OV<br>0·97 (95% CI 0·84–1·0).<br>**AUROC**<br>Any OV<br>0·80 (95% CI 0·68–0·92).<br>Medium/large OV<br>0·94 (95% CI 0·86–1·0).<br>**Kappa**<br>Any OV<br>0·45 (95% CI 0·40–0·49)<br>Small OV<br>0·19 (95% CI) 0·15–0·24)<br>Medium/large OV<br>0·47 (95% CI 0·42–0·52).
Eqbal et al., 2021 (FT)<br><br>Australia<br><br>Dates of procedures: NR<br><br>Trial registration: NR	N/A
Aedo et al., 2014 (FT)<br><br>Mexico<br><br>Dates of procedures: 11/2011 to 02/2012<br><br>Trial registration: NR	(see tables below)

Erosive gastroesophageal reflux disease (GERD)

		Conventional EGD		
		Yes	No	Total
EG scan	Yes	13	6	19
	No	16	61	77
	Total	29	67	96

Barrett's oesophagus

		Conventional EGD		
		Yes	No	Total
EG scan	Yes	8	4	12
	No	4	80	84
	Total	12	84	96

Oesophageal varices

		Conventional EGD		
		Yes	No	Total
EG scan	Yes	20	2	22
	No	1	73	74
	Total	21	75	96

Hiatal Hernia

		Conventional EGD		
		Yes	No	Total
EG scan	Yes	13	7	20
	No	20	56	76
	Total	33	63	96

**For SU**	Sensitivity	Specificity	PPV	NPV	Accuracy	Kappa (SE), 95% CI

Hiatal hernia	39·4 (23·4-57·7)	88·9 (77·8-95)	65 (41-83·7)	73·7 (62·1-82·8)	71·9 (61·6-80·3)	0·98 (0·103), 0·196-0·600
Barrett's oesophagus	66·7 (35·4-88·7)	95 (87·6-98·5)	66·7 (35·4-88·7)	95·2 (87·6-98·5)	91·7 (83·8-96·1)	0·619 (0·123), 0·378-0·860
Erosive GERD	44·8 (27-64)	91 (80·9-96·3)	68·4 (43·5-86·4)	79·2 (68·2-87·3)	77·1 (67·2-84·8)	0·398 (0·100). 0·196-0·600
Oesophageal varices	95·2 (74·1-99·8)	97·3 (89·8-99·5)	90·9 (69·3-98·4)	98·6 (91·7-99·9)	96·8 (90·5-99·1)	All: 0·910 (0·051) 0·810-1·010; large varices: 0·822 (0·086), 0·653-0·911; small varices: 0·591 (0·151), 0·294-0·880

Choi et al., 2014 (FT)

South Korea

Dates of procedures: 01/2012 to 09/2012

Trial registration: WHO ICTRP KCT0000298

Identification of gastrointestinal bleeding

		Conventional EGD		
		Yes	No	Total
EG scan	Yes	52	7	59
	No	29	41	70
	Total	81	48	129

Sensitivity (%): 64.2
Specificity (%): 85.4
Accuracy (%): 72.1

Cho et al., 2013 (FT)

South Korea

Dates of procedures: 01/2011 to 03/2011

Trial registration: NR

Identification of active gastrointestinal bleeding

		Conventional EGD		
		Yes	No	Total
EG scan	Yes	7	0	7
	No	1	5*	6
	Total	8	5	13

Sensitivity (%): 87.5
PPV (%): 100
NPV (%): 80 (as reported by the author; 83% based on the 2x2 table above)

Identification of inactive gastrointestinal bleeding

		Conventional EGD		
		Yes	No	Total
EG scan	Yes	4	1	5
	No	0	8	8
	Total	4	9	13

Specificity (%): 100 (as reported by the original authors; 88.9% based on the 2x2 table above).

Chung et al., 2012 (FT)

South Korea

Dates of procedures: 10/2010 to 02/2011

Trial registration: NR

N/A

Other SU UGI endoscopes

Römmele et al., 2024 (AB)<br><br>Germany<br><br>Dates of procedures: 03/2023 to 11/2023<br><br>Trial registration: NCT06192355	N/A
Wang et al., 2024 (AB)<br><br>China<br><br>Dates of procedures: 04/2023 to 06/2023<br><br>Trial registration: NR	N/A
Li et al., 2022 (FT)<br><br>China<br><br>Dates of procedures: 12/2019 to 06/2020<br><br>Trial registration: ChiCTR2000029945	N/A
Luo et al., 2022 (FT)<br><br>China<br><br>Dates of procedures: 06/2020 to 12/2020<br><br>Trial registration: NR	N/A
Dowds et al., 2024 (AB)<br><br>USA<br><br>Dates of procedures: 12/2022 to 11/2023<br><br>Trial registration: NR	N/A
Lee et al., 2024 (AB)<br><br>USA<br><br>Dates of procedures: 09/2022 to 11/2023<br><br>Trial registration: NR	N/A
van der Ploeg et al., 2023 (FT)<br><br>Netherlands, Norway<br><br>Dates of procedures: NR<br><br>Trial registration: NCT05504434	N/A
**Sheathed MU endoscopes**	

Shariff et al., 2016 (FT)<br><br>UK<br><br>Dates of procedures: NR<br><br>Trial registration: NR	**Sensitivity**<br>BO 100% (95% CI, 75% – 100%)<br>Intestinal metaplasia (IM) 67% (95% CI, 30% – 93%)<br>**Specificity**<br>BO 100% (95% CI, 74% – 100%)<br>IM 100% (95% CI, 79% – 100%)<br>**PPV**<br>BO 100%<br>IM 100%<br>**NPV**<br>BO 100%<br>**Kappa**<br>BO 0·738 (95% CI, 0·41 to 1·00)
Sami et al., 2015 (FT)<br><br>USA<br><br>Dates of procedures: 03/2011 to 10/2013<br><br>Trial registration: NCT01288612	N/A
Jin et al., 2014 (FT) [c]<br><br>China<br><br>Dates of procedures: 07/2010 to 09/2011<br><br>Trial registration: NR	Cancer: 2 (0·5%) vs 1 (0·5%)<br>Peptic ulcer: 75 (13%) vs 70 (13%)<br>Reflux esophagitis: 45 (8%) vs 53 (10%)<br>Gastritis: 87 (15%) vs 101 (18%)<br>Esophagogastric varices: 2 (0.5%) vs 4 (1%)<br>Polyps: 4 (1%) vs 3 (0.5%)<br>Submucosal lesions: 6 (1%) vs 7 (1%)<br>No apparent abnormality: 347 (61%) vs 313 (57%).<br><br>Total pathology detection rate<br>39% vs 43%. (P = 0·136).
Jin et al., 2015 (AB)<br><br>China<br><br>Dates of procedures: NR<br><br>Trial registration: NR	N/A
Streckfuss et al., 2014 (FT)<br><br>Germany<br><br>Dates of procedures: NR<br><br>Trial registration: NR	N/A
Peery et al., 2011 (AB)<br><br>USA<br><br>Dates of procedures: NR<br><br>Trial registration: NR	N/A

[a] AB - conference abstract; FT – full-text article; [b] Crossover was defined as the need to change to the alternate endoscope type when requisite interventions could not be performed successfully using the assigned endoscope; [c] the study also included a pilot RCT (n=20) examining microbial contamination, which is not reported here. [c] Endoscopists were from one centre (Beijing Military General Hospital) but the endoscopic procedures were carried out in 47 local medical units in Inner Mongolia, Shanxi and Hebei provinces in China. [d] One hour is unlikely to be sufficient time for infectious adverse events to develop.

AB: conference abstract; AUROC: (Area under ROC curve); BO: Barrett's oesophagus; CI: Confidence interval; EGD: esophagogastroduodenoscopy; FT: full-text article; HROV: high risk oesophageal varices; IM: intestinal metaplasia; IQR: interquartile range; MU: multiple-use (endoscope); N/A: not applicable; NPV: negative predictive value; NR: not reported; OV: oesophageal varices; PPV: positive predictive value; RCT - randomised controlled trial; SE: standard error; SU: single-use (endoscope); UGI: upper gastrointestinal.

*^One of these was judged as active bleeding by both methods but was later found to be haemoptysis (ingestion of blood from lung cancer).

2.3 Infection risk and adverse events

Supplementary Table 20. Adverse events and infection rates: Other upper GI endoscopes

Author, year, country, publication type [a], dates of procedures, trial registration	SU group - n / % non-infectious adverse events (AEs)	SU group – n / % infectious AEs	MU group – n / % non-infectious AEs	MU group – n / % infectious AEs
**SU upper gastrointestinal (UGI) endoscopes: EG Scan (IntroMedic), ultrathin transnasal capsule**				
Halland et al., 2020 (AB)<br><br>USA<br><br>Dates of procedures: NR<br><br>Trial registration: NR	1 patient (2%) experienced an AE: 1 (2%) self-limiting epistaxis.	None reported	None reported	None reported
Kang et al., 2019 (FT)<br><br>South Korea<br><br>Dates of procedures: 03/2016 to 07/2016<br><br>Trial registration: NR	SAEs reported: 0.<br>AEs reported: 0.	None reported	SAEs reported: 0.<br>AEs reported: 0.	None reported
Sami et al., 2019 (FT)<br><br>UK, USA<br><br>Dates of procedures: 07/2012 to 10/2015<br><br>ISRCTN 70595405 / NCT02066233	SAEs reported: 0.<br>5 patients (3%) experienced an AE: 2 (1%) probe technical failure and terminated procedure, 2 (1%) vasovagal symptoms, 1 (1%) self-limiting epistaxis within 14 days.	None reported	None reported	None reported
Huynh et al., 2017 (FT)<br><br>Australia<br><br>Dates of procedures: 09/2013 to 11/2014<br><br>ACTRN12616001103459	SAEs reported: 0.<br>1 patient (2%) experienced an AE: 1 (%) abdominal bloating and distension within 7 days.	None reported	None reported	None reported
Jin et al., 2017 (AB)<br><br>South Korea<br><br>Dates of procedures: NR<br><br>Trial registration: NCT03009383	SAEs reported: 0.<br>Odynophagia (52%) and dysphagia (26%) (which procedure NR).	None reported	See SU group.	See SU group.

Author, year, country, publication type [a], dates of procedures, trial registration	SU group - n / % non-infectious adverse events (AEs)	SU group – n / % infectious AEs	MU group – n / % non-infectious AEs	MU group – n / % infectious AEs
Sami et al., 2016 (FT)<br><br>UK<br><br>Dates of procedures: 07/2012 to 02/2014<br><br>Trial registration: ISRCTN: 70595- 405	SAEs reported: 0. 4 patients (9%) reported an AE: 4 (9%) self-limiting epistaxis within 2 weeks.	None reported	None reported	None reported
Eqbal et al., 2021 (FT)<br><br>Australia<br><br>Dates of procedures: NR<br><br>Trial registration: NR	1 patient (3%) reported an AE: 1 (3%) transient tachycardia within 2 weeks.	None reported	N/A	N/A
Aedo et al., 2014 (FT)<br><br>Mexico<br><br>Dates of procedures: 11/2011 to 02/2012<br><br>Trial registration: NR	NR	NR	NR	NR
Choi et al., 2014 (FT)<br><br>South Korea<br><br>Dates of procedures: 01/2012 to 09/2012<br><br>Trial registration: WHO ICTRP KCT0000298	60 (47%) nasal pain, 26 (20%) nausea, 11 (9%) epistaxis, 9 (7%) cough, 1 (1%) other.	None reported	NR	NR
Cho et al., 2013 (FT)<br><br>South Korea<br><br>Dates of procedures: 01/2011 to 03/2011<br><br>Trial registration: NR	Reported 0.	None reported	NR	NR
Chung et al., 2012 (FT)<br><br>South Korea<br><br>Dates of procedures: 10/2010 to 02/2011<br><br>Trial registration: NR	SAEs reported: 0. AEs reported: 0	None reported	N/A	N/A
**Other SU UGI endoscopes**				

Author, year, country, publication type [a], dates of procedures, trial registration	SU group - n / % non-infectious adverse events (AEs)	SU group – n / % infectious AEs	MU group – n / % non-infectious AEs	MU group – n / % infectious AEs
Römmele et al., 2024 (AB)<br><br>Germany<br><br>Dates of procedures: 03/2023 to 11/2023<br><br>Trial registration: NCT06192355	NR	NR	NR	NR
Wang et al., 2024 (AB)<br><br>China<br><br>Dates of procedures: 04/2023 to 06/2023<br><br>Trial registration: NR	SAEs: None reported.<br>AEs reported: NR.	None reported.	SAEs: None reported.<br>AEs reported: NR (P>0·05).	None reported.
Li et al., 2022 (FT)<br><br>China<br><br>Dates of procedures: 12/2019 to 06/2020<br><br>Trial registration: ChiCTR2000029945	0 patients reported an AE within 1 day [b].	Reported 0.	Reported 0 (P=1).	Reported 0 (P=1).
Luo et al., 2022 (FT)<br><br>China<br><br>Dates of procedures: 06/2020 to 12/2020<br><br>Trial registration: NR	0 patients reported an AE within 1 hour [b].	Reported 0.	Reported 0.	Reported 0.
Dowds et al., 2024 (AB)<br><br>USA<br><br>Dates of procedures: 12/2022 to 11/2023<br><br>Trial registration: NR	SAEs reported: 0.<br>AEs: None reported.	SAEs reported: 0.<br>AEs: None reported.	N/A	N/A
Lee et al., 2024 (AB)<br><br>USA<br><br>Dates of procedures: 09/2022 to 11/2023<br><br>Trial registration: NR	SAEs: None reported.<br>2 patients (4%) reported an AE: 1 (2%) syncope. 1 (2%) transient elevated blood pressure.	SAEs: None reported.<br>SAEs: None reported.	N/A	N/A
van der Ploeg et al., 2023 (FT)<br><br>Netherlands, Norway<br><br>Dates of procedures: NR	SAEs reported: 0.<br>AEs reported: 0.	None reported	N/A	N/A

Author, year, country, publication type [a], dates of procedures, trial registration	SU group - n / % non-infectious adverse events (AEs)	SU group – n / % infectious AEs	MU group – n / % non-infectious AEs	MU group – n / % infectious AEs
Trial registration: NCT05504434				
**Sheathed MU endoscopes**				
Shariff et al., 2016 (FT)<br><br>UK<br><br>Dates of procedures: NR<br><br>Trial registration: NR	None reported	None reported	None reported	None reported
Sami et al., 2015 (FT) [c]<br><br>USA<br><br>Dates of procedures: 03/2011 to 10/2013<br><br>Trial registration: NCT01288612	Mobile unit: SAEs reported: 0. 23 patients (30%) experienced an AE within 30 days: 12 patients (16%) sore throat, 4 (5%) headache, 3 (4%) bloating, 2 (3%) abdominal/chest discomfort, 1 (1%) epistaxis, 1 (1%) nasal pain.<br><br>Hospital outpatient: SAEs reported: 0. 20 patients (26%) experienced an AE within 30 days: 10 patients (14%) sore throat, 4 (6%) bloating, 4 (6%) abdominal/chest discomfort, 1 (1%) epistaxis, 1 (1%) headache, 0 (0%) nasal pain.	None reported	SAEs reported: 0. 20 patients (3%) experienced an AE within 30 days: 8 patients (13%) sore throat (p=0·7), 2 (3%) bloating (p=0·83), 4 (7%) abdominal/chest discomfort (p=0·51), 0 (0%) epistaxis (p=1·0), 0 (0%) headache (p=0·16), 0 (0%) nasal pain (p=1·0). [e]	None reported
Jin et al., 2014 (FT) [d]<br><br>China<br><br>Dates of procedures: 07/2010 to 09/2011<br><br>Trial registration: NR	SAEs reported: 0. AEs reported: 0.	None reported	SAEs reported: 0. AEs reported: 0.	None reported
Jin et al., 2015 (AB)<br><br>China<br><br>Dates of procedures: NR<br><br>Trial registration: NR	Reported 0.	Reported 0.<br><br>No microbial contamination detected in each gastroscope after procedures.	Reported 0.	Reported 0.<br><br>No microbial contamination detected in each gastroscope after procedures.
Streckfuss et al., 2014 (FT)<br><br>Germany	Reported 0.	None reported	N/A	N/A

Author, year, country, publication type [a], dates of procedures, trial registration	SU group - n / % non-infectious adverse events (AEs)	SU group – n / % infectious AEs	MU group – n / % non-infectious AEs	MU group – n / % infectious AEs
Dates of procedures: NR<br><br>Trial registration: NR				
Peery et al., 2011 (AB)<br><br>USA<br><br>Dates of procedures: NR<br><br>Trial registration: NR	SAEs reported: 0. 5 patients (2%) experienced an AE. This included light-headedness, nausea, headache, and nasal irritation. (Further details NR.)	None reported	N/A	N/A

[a] AB - conference abstract; FT – full-text article; [b] One hour or one day is unlikely to be sufficient time for infectious adverse events to develop. [c] P values for this study from comparisons between three groups. [d] the study also included a pilot RCT (n=20) examining microbial contamination, which is not reported here.

AB: conference abstract; AE: Adverse Event; FT: full-text article; IQR: interquartile range; MU: multiple-use (endoscope); N/A: not applicable; NR: not reported; RCT - randomised controlled trial; SAE: Serious Adverse Event; SU: single-use (endoscope); UGI: upper gastrointestinal.

2.4 Other outcomes

Supplementary Table 21. Clinical outcomes / Patient Reported Outcomes: Other upper GI endoscopes

Author, year, country, publication type [a], dates of procedures, trial registration	Clinical and/or patient reported outcomes
**SU upper gastrointestinal (UGI) endoscopes:<br>EG Scan (IntroMedic), ultrathin transnasal capsule**	
Halland et al., 2020 (AB)<br><br>USA<br><br>Dates of procedures: NR<br><br>Trial registration: NR	Tolerability<br>0 Best – 10 Worst<br>Mean:<br><br>Nasal pain: 4<br>Gagging: 3,<br>Discomfort: 3,<br>Choking: 1.<br>vs N/A (Data NR).
Kang et al., 2019 (FT)<br><br>South Korea<br><br>Dates of procedures:<br>03/2016 to 07/2016<br><br>Trial registration: NR	0 Best – 9 Worst<br>Mean (±SD):<br><br>Overall discomfort:<br>3 (±1) vs 4 (±1) (P=0·030),<br>Anxiety for endoscopy:<br>2 (±1) vs 3 (±1) (P=0·373),<br>Pain:<br>3 (±1) vs 4 (±1) (P=0·239),<br>Nausea and vomiting:<br>3 (±1) vs 4 (±1) (P=0·051),<br>Abdominal fullness:<br>3 (±1) vs 4 (±1) (P=0.044).
Sami et al., 2019 (FT)<br><br>UK, USA<br><br>Dates of procedures:<br>07/2012 to 10/2015<br><br>ISRCTN 70595405 /<br>NCT02066233	Spielberger State Trait Anxiety (STAI-Y):<br>(20 – 80 range)<br>Higher scores = higher anxiety<br>Mean (±standard error of the mean):<br><br>Pre-endoscopy: 33 (±1) (for both),<br>Post-endoscopy:<br>SU: 31 (±1) (P=0·0003).<br>MU: 29 (±1), (P=0·0001).
Huynh et al., 2017 (FT)<br><br>Australia<br><br>Dates of procedures:<br>09/2013 to 11/2014<br><br>ACTRN12616001103459	0 Best – 9 Worst<br>Mean (±SD):<br><br>Pain:<br>Pre-endoscopy: 1 (±0·5),<br>During endoscopy: 2 (±0·5),<br>(P=0·045).<br><br>STAI form Y1 (state):<br>Mean (±SD):<br><br>Pre-endoscopy: 47 (±1),<br>During endoscopy: 47 (±9), (P=0·96).
Jin et al., 2017 (AB)<br><br>South Korea<br><br>Dates of procedures: NR<br><br>Trial registration:<br>NCT03009383	N/A

Sami et al., 2016 (FT)<br><br>UK<br><br>Dates of procedures:<br>07/2012 to 02/2014<br><br>Trial registration: ISRCTN: 70595- 405	N/A
Eqbal et al., 2021 (FT)<br><br>Australia<br><br>Dates of procedures: NR<br><br>Trial registration: NR	N/A
Aedo et al., 2014 (FT)<br><br>Mexico<br><br>Dates of procedures:<br>11/2011 to 02/2012<br><br>Trial registration: NR	Level of discomfort for nasal pain and nausea:<br>No or only mild pain: 77 (80%)<br>Moderate: 0 (0%)<br>Severe: 0 (0%)<br>Did not experience nausea 84 (88%)
Choi et al., 2014 (FT)<br><br>South Korea<br><br>Dates of procedures:<br>01/2012 to 09/2012<br><br>Trial registration:<br>WHO ICTRP KCT0000298	N/A
Cho et al., 2013 (FT)<br><br>South Korea<br><br>Dates of procedures:<br>01/2011 to 03/2011<br><br>Trial registration: NR	N/A
Chung et al., 2012 (FT)<br><br>South Korea<br><br>Dates of procedures:<br>10/2010 to 02/2011<br><br>Trial registration: NR	Epistaxis:<br>None: 46 (100%)<br>Mild: 0 (0%)<br>Moderate: 0 (0%)<br>Severe: 0 (0%)<br><br>Nasal pain:<br>None: 2 (4%)<br>Mild: 30 (65%)<br>Moderate: 14 (30%)<br>Severe: 0 (0%)<br><br>Nausea:<br>None: 13 (28%)<br>Mild: 23 (50%)<br>Moderate: 10 (22%)<br>Severe: 0 (0%)<br><br>Sore throat:<br>None: 20 (44%)<br>Mild: 16 (35%)<br>Moderate: 10 (22%)<br>Severe: 0 (0%)<br><br>Choling sensation:<br>None: 35 (76%)<br>Mild: 9 (20%)<br>Moderate: 2 (4%)<br>Severe: 0 (0%)

**Other SU UGI endoscopes**	
Römmele et al., 2024 (AB)<br><br>Germany<br><br>Dates of procedures:<br>03/2023 to 11/2023<br><br>Trial registration: NCT06192355	N/A
Wang et al., 2024 (AB)<br><br>China<br><br>Dates of procedures:<br>04/2023 to 06/2023<br><br>Trial registration:<br>NR	N/A
Li et al., 2022 (FT)<br><br>China<br><br>Dates of procedures:<br>12/2019 to 06/2020<br><br>Trial registration:<br>ChiCTR2000029945	N/A
Luo et al., 2022 (FT)<br><br>China<br><br>Dates of procedures:<br>06/2020 to 12/2020<br><br>Trial registration: NR	Pre-procedure:<br>Systolic blood pressure stability:<br>76% vs 93% (P=0·022),<br>Diastolic blood pressure stability:<br>82% vs 83% (P=0·870),<br>Heart rate stability:<br>86% vs 87% (P=0·841).<br><br>During procedure:<br>Systolic blood pressure stability:<br>84% vs 78% (P=0·438),<br>Diastolic blood pressure stability:<br>75% vs 74% (P=0·955),<br>Heart rate stability:<br>84% vs 83% (P=0·966).<br><br>10 ± 5 minutes after procedure:<br>Systolic blood pressure stability:<br>87% vs 78% (P=0·207),<br>Diastolic blood pressure stability:<br>87% vs 75% (P=0·089),<br>Heart rate stability:<br>78% vs 75% (P=0·654).<br><br>60 ± 15 minutes after procedure:<br>Systolic blood pressure stability:<br>100% vs 91% (P=0·057),<br>Diastolic blood pressure stability:<br>95% vs 95% (P=1·000),<br>Heart rate stability:<br>84% vs 73% (P=0·166).
Dowds et al., 2024 (AB)<br><br>USA<br><br>Dates of procedures:<br>12/2022 to 11/2023<br><br>Trial registration: NR	TransNasal Endoscopy ease score (TNEase):<br>1 With Ease, 5 Procedure terminated<br>Mean (±SD):<br><br>1 (±1).
Lee et al., 2024 (AB)<br><br>USA<br><br>Dates of procedures:<br>09/2022 to 11/2023	TransNasal Endoscopy ease score (TNEase):<br>1 With Ease, 5 Procedure terminated<br>n (%) rating by HCP:<br><br>With ease: 34 (69%).<br>Minimal complaints: 8 (16%).<br>Moderate complaints: 5 (10%).

Trial registration: NR	Significant complaints: 0 (0%).<br>Procedure terminated: 2 (4%).
van der Ploeg et al., 2023 (FT)<br><br>Netherlands, Norway<br><br>Dates of procedures: NR<br><br>Trial registration: NCT05504434	N/A
**Sheathed MU endoscopes**	
Shariff et al., 2016 (FT)<br><br>UK<br><br>Dates of procedures: NR<br><br>Trial registration: NR	N/A
Sami et al., 2015 (FT)<br><br>USA<br><br>Dates of procedures:<br>03/2011 to 10/2013<br><br>Trial registration:<br>NCT01288612	Tolerability<br>0 No pain – 10 Severe pain<br>Mean (±SD):<br><br>Pain: 3 (±9) [sic] vs 3 (±2) vs 0.5 (±1) (p=<0·001),<br>Choking: 1 (±2) vs 1 (±2) vs 0 (±0) (p=<0·001),<br>Gagging: 1 (±2) vs 1 (±2) vs 0.5 (±1) (p=<0·001),<br>Anxiety: 3 (±3) vs 2 (±2) vs 1 (±2) (p=<0·001).<br><br>Overall tolerance<br>0 Good – 10 Poor tolerance<br>Mean (±SD):<br><br>Overall tolerance: 2 (±2) vs 2 (±2) vs 0.5 (±1) (p=<0·001).
Jin et al., 2014 (FT) [c]<br><br>China<br><br>Dates of procedures:<br>07/2010 to 09/2011<br><br>Trial registration: NR	Patient-reported discomfort level<br><br>No obvious discomfort: 54 (10%) vs 59 (11%),<br>Mild discomfort: 364 (64%) vs 373 (68%),<br>Moderate discomfort: 146 (26%) vs 118 (21%),<br>Severe discomfort: 4 (1%) vs 2 (0.5%).
Jin et al., 2015 (AB)<br><br>China<br><br>Dates of procedures: NR<br><br>Trial registration: NR	N/A
Streckfuss et al., 2014 (FT)<br><br>Germany<br><br>Dates of procedures: NR<br><br>Trial registration: NR	Discomfort<br>1 No discomfort – 5 Severe discomfort<br>Mean (±SD):<br><br>Overall discomfort: 2 (±1),<br>Endoscopy of the pharynx: 2 (±1),<br>Endoscopy of the passage of the nose: 2 (±1),<br>Endoscopy of the oesophagus: 2 (±1),<br>Time: 1 (SD NR).
Peery et al., 2011 (AB)<br><br>USA<br><br>Dates of procedures: NR<br><br>Trial registration: NR	Patient-reported acceptability (SU):<br>“excellent” in all domains.<br><br>0 None – 10 Maximal<br>Median:<br><br>Choking during procedure: 1,<br>Choking during insertion: 1,<br>Gagging during procedure: 2,<br>Gagging during insertion: 2,<br>Pain during procedure: 2,

Pain during insertion: 2.
(Further detail in Figure 1)

---

[a] AB - conference abstract; FT – full-text article; [b] Crossover was defined as the need to change to the alternate endoscope type when requisite interventions could not be performed successfully using the assigned endoscope; [c] the study also included a pilot RCT (n=20) examining microbial contamination, which is not reported here. [c] Endoscopists were from one centre (Beijing Military General Hospital) but the endoscopic procedures were carried out in 47 local medical units in Inner Mongolia, Shanxi and Hebei provinces in China.

AB: conference abstract; FT: full-text article; HCP: healthcare professional; MU: multiple-use (endoscope); N/A: not applicable; NR: not reported; RCT - randomised controlled trial; SD: standard deviation; STAI: Spielberger State Trait Anxiety; SU: single-use (endoscope); TNEasee: TransNasal Endoscopy ease score; UGI: upper gastrointestinal.

Supplementary Table 22.Patient views: Other upper GI endoscopes

Author, year, country, publication type [a], dates of procedures, trial registration	Views of patients
**SU upper gastrointestinal (UGI) endoscopes:<br>EG Scan (IntroMedic), ultrathin transnasal capsule**	
Halland et al., 2020 (AB)<br><br>USA<br><br>Dates of procedures: NR<br><br>Trial registration: NR	N/A
Kang et al., 2019 (FT)<br><br>South Korea<br><br>Dates of procedures: 03/2016 to 07/2016<br><br>Trial registration: NR	Tolerability<br>0 Best – 9 Worst<br>Mean (±SD):<br><br>Overall satisfaction:<br>2±1 vs 3±1 (P=0·273),<br>Intention to retry endoscopy: 3±1 vs 3±1 (P=0·399).
Sami et al., 2019 (FT)<br><br>UK, USA<br><br>Dates of procedures: 07/2012 to 10/2015<br><br>ISRCTN 70595405 / NCT02066233	Acceptability<br>0 Worst ever experience – 10 Best ever experience<br>Mean (±standard error of the mean)<br><br>7 (±2) vs 6 (±3) (p=0·0004).<br><br>Preference:<br>91 (54%) vs 28 (17%) (p<0·001).<br>(Further detail in Table 2)

Huynh et al., 2017 (FT)<br><br>Australia<br><br>Dates of procedures: 09/2013 to 11/2014<br><br>ACTRN12616001103459	25 (52%) preferred (unsedated) SU,<br>19 (40%) preferred (sedated) MU,<br>4 (8%) equally satisfied.
Jin et al., 2017 (AB)<br><br>South Korea<br><br>Dates of procedures: NR<br><br>Trial registration: NCT03009383	N/A
Sami et al., 2016 (FT)<br><br>UK<br><br>Dates of procedures: 07/2012 to 02/2014<br><br>Trial registration: ISRCTN: 70595- 405	Tolerability<br>0 Worst ever experience – 10 Best ever experience<br>Mean (±SD):<br><br>Overall experience:<br>Day 0:<br>8 (±2) vs 7 (±3) (p=0·058),<br>Day 14:<br>7 (±2) vs 5 (±3) (p<0·001).<br><br>Preference:<br>Day 0:<br>26 (77%) vs 8 (24%) (p<0·001),<br>Day 14:<br>21 (78%) vs 6 (22%) (p<0·001).<br>(Further detail in Table 2)
Eqbal et al., 2021 (FT)<br><br>Australia<br><br>Dates of procedures: NR<br><br>Trial registration: NR	N/A
Aedo et al., 2014 (FT)<br><br>Mexico<br><br>Dates of procedures: 11/2011 to 02/2012<br><br>Trial registration: NR	N/A
Choi et al., 2014 (FT)<br><br>South Korea<br><br>Dates of procedures: 01/2012 to 09/2012<br><br>Trial registration: WHO ICTRP KCT0000298	N/A
Cho et al., 2013 (FT)<br><br>South Korea<br><br>Dates of procedures: 01/2011 to 03/2011<br><br>Trial registration: NR	N/A

Chung et al., 2012 (FT)<br><br>South Korea<br><br>Dates of procedures: 10/2010 to 02/2011<br><br>Trial registration: NR	N/A
**Other SU UGI endoscopes**	
Römmele et al., 2024 (AB)<br><br>Germany<br><br>Dates of procedures: 03/2023 to 11/2023<br><br>Trial registration: NCT06192355	N/A
Wang et al., 2024 (AB)<br><br>China<br><br>Dates of procedures: 04/2023 to 06/2023<br><br>Trial registration: NR	N/A
Li et al., 2022 (FT)<br><br>China<br><br>Dates of procedures: 12/2019 to 06/2020<br><br>Trial registration: ChiCTR2000029945	N/A
Luo et al., 2022 (FT)<br><br>China<br><br>Dates of procedures: 06/2020 to 12/2020<br><br>Trial registration: NR	N/A
Dowds et al., 2024 (AB)<br><br>USA<br><br>Dates of procedures: 12/2022 to 11/2023<br><br>Trial registration: NR	N/A
Lee et al., 2024 (AB)<br><br>USA<br><br>Dates of procedures: 09/2022 to 11/2023<br><br>Trial registration: NR	N/A
van der Ploeg et al., 2023 (FT)<br><br>Netherlands, Norway	N/A

Dates of procedures: NR<br><br>Trial registration: NCT05504434	
**Sheathed MU endoscopes**	
Shariff et al., 2016 (FT)<br><br>UK<br><br>Dates of procedures: NR<br><br>Trial registration: NR	Overall experience:<br>0 Worst ever experience – 10 Best ever experience<br>Mean (±Standard error of mean):<br><br>7 (±0·5) vs 4 (±0·5) (p=0·0006).<br><br>Preference<br>12 (60%) preferred (unsedated) SU,<br>5 (25%) preferred (sedated) MU,<br>3 (15%) no preference.<br><br>Common comments by patients preferring SU: "nasal felt less intrusive", "easier to tolerate", and "I was able to talk".
Sami et al., 2015 (FT)<br><br>USA<br><br>Dates of procedures: 03/2011 to 10/2013<br><br>Trial registration: NCT01288612	Willing to have procedure again:<br>60 (79%) vs 60 (83%) vs 57 (93%) (p=0·001).
Jin et al., 2014 (FT) [b]<br><br>China<br><br>Dates of procedures: 07/2010 to 09/2011<br><br>Trial registration: NR	N/A
Jin et al., 2015 (AB)<br><br>China<br><br>Dates of procedures: NR<br><br>Trial registration: NR	N/A
Streckfuss et al., 2014 (FT)<br><br>Germany<br><br>Dates of procedures: NR<br><br>Trial registration: NR	N/A
Peery et al., 2011 (AB)<br><br>USA<br><br>Dates of procedures: NR<br><br>Trial registration: NR	Patient-reported acceptability (SU):<br>0 None – 10 Maximal<br>Median:<br><br>Overall tolerability: 2.<br><br>(2 is low, though results are described as "excellent" in the text. Maybe score should have been reverse, or 'issue with' was omitted.)

[a] AB - conference abstract; FT – full-text article. [b] Endoscopists were from one centre (Beijing Military General Hospital) but the endoscopic procedures were carried out in 47 local medical units in Inner Mongolia, Shanxi and Hebei provinces in China.

AB: conference abstract; FT: full-text article; MU: multiple-use (endoscope); N/A: not applicable; NR: not reported; SD: standard deviation; SU: single-use (endoscope); UGI: upper gastrointestinal.

Views of staff using, procuring, cleaning, and / or disposing: Other upper GI endoscopes

No included studies of SU upper GI endoscopes reported views of staff using, procuring, cleaning, and / or disposing: the endoscopes.

3. Lower GI endoscopes and studies across different types of endoscope

3.1 Technical performance

Supplementary Table 23. Comparative evidence on successful intubation rates for colonoscopes

Study, publication type, study design, study location & case complexity (Bold = DTA or RCT)	Findings	P value
SU vs MU		
**Wei et al. 2022, RCT**<br>**China**	XZING-C200 (HuiZhou Xzing)) vs CF- HQ290ZI, CF-H290I vs PCF-TYPE-Q260AZI (Olympus)<br>***Successful caecal intubation***<br>100% (45/45) vs 100% (45/45).	1·000

Abbreviations: DTA – diagnostic test accuracy; MU – multiple-use; RCT – Randomised Controlled Trial; SU – single-use

Yellow = Full-text article, White = Abstract

Supplementary Table 24 Comparative evidence on ERCP successful procedure completion (excluding successful cross-over)

Study, publication type, study design, study location & case complexity (Bold = RCT)	Findings	P value
SU vs MU		
**Xu et al. 2024, RCT**<br>**China**	EndoFresh® XZING-W200B (HuiZhou Xzing) vs EG-600WR (Fujifilm)<br><br>***Successful endoscopic rubber band ligation***<br>100% (21/21) vs 100% (21/21).	NR
**Wei et al. 2022, RCT**<br>**China**	XZING-C200 (HuiZhou Xzing)) vs CF- HQ290ZI, CF-H290I vs PCF-TYPE-Q260AZI (Olympus)<br><br>***Successful endoscopic mucosal resection***<br>100% (19/19) vs 100% (17/17).	1·000

Abbreviations: MU – multiple-use; NR – not reported; RCT – Randomised Controlled Trial; SU – single-use

Yellow = Full-text article, White = Abstract

Supplementary Table 25. Technical outcomes – Defect rate: Lower GI endoscopes and studies across different types of endoscope

Author, year, country, publication type [a], dates of procedures, trial registration	Description of population, centre, single-use (SU) endoscope, average age (years), % female, indications	Description of multiple-use (MU) endoscope	SU group vs MU group - Defect / device failure rate
**SU endoscopes**			
Xu et al., 2024 (FT)<br><br>China<br><br>Dates of procedures: 05/2022 to 06/2022<br><br>Trial registration: ChiCTR2200060014	Adult patients with symptomatic internal hemorrhoids scheduled for endoscopic rubber band ligation.<br>SU colonoscope EndoFresh® XZING-W200B (HuiZhou Xzing) (n=21) vs EG-600WR (n=21).<br>Mean age: 40 (±7) vs 41 (±9)<br>Female: 57% vs 57%<br>Main indications:<br>Haemorrhoids Grade III: 57% vs 67%,<br>Grade II: 43% vs 33%.	EG-600WR (Fujifilm), MU gastroscope (n=21)	Device failure: 0 (0%) vs 0 (0%).
Wei et al., 2022 (FT)<br><br>China<br><br>Dates of procedures: 04/2021 to 04/2022<br><br>Trial registration: ChiCTR2100045084	Adult patients scheduled for colonoscopy.<br>SU colonoscope XZING-C200 (HuiZhou Xzing) (n=45) vs GIF-HQ290/GIF-H290) (n=45).<br>Mean age: 43 (±14) vs 43 (±13)<br>Female: 47% vs 47%<br>Main indications:<br>Prior history of polyp: 44% vs 56%,<br>Bloating: 38% vs 27%,<br>Abdominalgia: 31% vs 33%.	CF- HQ290ZI, CF-H290I, and PCF-TYPE-Q260AZI (Olympus), MU colonoscope (n=45)	Device failure/malfunction: 0 (0%) vs 0 (0%).
**MU endoscopes only**			
Deb et al., 2022 (FT)<br><br>Multiple countries<br><br>Published by 10/2020<br><br>Trial registration: N/A	Patients undergoing GI endoscopy.<br>Age: NR<br>Female: NR<br>Main indications: NR<br><br>N=118 studies	MU endoscope (details NR)	N/A

[a] AB - conference abstract; FT – full-text article

AB: conference abstract; FT: full-text article; GI: gastrointestinal; MU: multiple-use (endoscope); N/A: not applicable; NR – not reported; SU: single-use (endoscope).

Supplementary Table 26. Technical outcomes – Image quality: Lower GI endoscopes and studies across different types of endoscope

Author, year, country, publication type [a], dates of procedures,	SU group vs MU group - Image quality	SU group vs MU group -

trial registration		Successful visualisation	
**SU endoscopes**			
Xu et al., 2024 (FT)<br><br>China<br><br>Dates of procedures: 05/2022 to 06/2022<br><br>Trial registration: ChiCTR2200060014	Image clarity:<br>A - Good brightness, contrast, and clarity / B - Fair / C - Poor<br><br>A: 18 (86%) vs 20 (95%), B: 3 (14%) / 1 (5%), C: 0 (0%) vs 0 (0%)(p=0·60).	N/A	
Wei et al., 2022 (FT)<br><br>China<br><br>Dates of procedures: 04/2021 to 04/2022<br><br>Trial registration: ChiCTR2100045084	0 Missing sites / unclear images – 4 No missing sites and extremely clear images<br>Mean (±SD):<br><br>Image quality score: 26 (±1) vs 27 (±1)(p<0·001).<br><br>(Further detail in Table 3.)	Success rate photographing iconic anatomical sites:<br>45 (100%) vs 45 (100%) (CI - 7·8654%, 7·8654%).	
**MU endoscopes**			
Deb et al., 2022 (FT)<br><br>Multiple countries<br><br>Published by 10/2020<br><br>Trial registration: N/A	N/A	N/A	

[a] AB - conference abstract; FT – full-text article

AB: conference abstract; FT: full-text article; GI: gastrointestinal; MU: multiple-use (endoscope); N/A: not applicable; NR – not reported; SD: standard deviation; SU: single-use (endoscope).

Supplementary Table 27. Technical outcomes – Duration: Lower GI endoscopes and studies across different types of endoscope

Author, year, country, publication type [a], dates of procedures, trial registration	SU group vs MU group - Time to landmarks / cannulate / intubate	SU group vs MU group - Time for procedure / retroflexion	SU group vs MU group - Time for (pre-) cleaning / discarding / reprocessing instruments / turning around room / discharging patient / transportation
**SU endoscopes**			

Xu et al., 2024 (FT)<br><br>China<br><br>Dates of procedures: 05/2022 to 06/2022<br><br>Trial registration: ChiCTR2200060014	N/A	Time from forward to retroflexed position<br><br>Median (range) seconds:<br><br>11 (9-15) vs 10 (8-12)(p=0·215).	N/A
Wei et al., 2022 (FT)<br>China<br><br>Dates of procedures: 04/2021 to 04/2022<br><br>Trial registration: ChiCTR2100045084	Entry time<br><br>Mean (± SD) seconds:<br><br>288 (±121) vs 292 (±1) (p=0·910).<br><br>Withdrawal time<br><br>Mean (± SD) seconds:<br><br>482 (±89) vs 367 (±192) (p=0·001).	Procedure time<br><br>Mean (± SD) seconds:<br><br>466 (±181) vs 206 (±110) (p<0·001).	N/A
**MU endoscopes only**			
Deb et al., 2022 (FT)<br><br>Multiple countries<br><br>Published by 10/2020<br><br>Trial registration: N/A	N/A	N/A	N/A

[a] AB - conference abstract; FT – full-text article

AB: conference abstract; FT: full-text article; GI: gastrointestinal; MU: multiple-use (endoscope); N/A: not applicable; NR – not reported; SD; standard deviation; SU: single-use (endoscope).

3.2 Test accuracy

Supplementary Table 28. Diagnostic accuracy: Lower GI endoscopes and studies across different types of endoscope

Author, year, country, publication type [a], dates of procedures, trial registration	Diagnostic accuracy
**SU endoscopes**	
Xu et al., 2024 (FT)<br><br>China<br><br>Dates of procedures: 05/2022 to 06/2022<br><br>Trial registration: ChiCTR2200060014	N/A
Wei et al., 2022 (FT)<br><br>China<br><br>Dates of procedures: 04/2021 to 04/2022<br><br>Trial registration: ChiCTR2100045084	Polyp detection rate:<br><br>21 vs 22 (P=1·000).<br><br>Adenoma detection rate:<br><br>14 vs 11 (P=0·638).<br><br>Diminutive adenoma detection rate:<br><br>11 vs 23 (P=0·016).
**MU endoscopes only**	
Deb et al., 2022 (FT)<br><br>Multiple countries<br><br>Published by 10/2020<br><br>Trial registration: N/A	N/A

[a] AB - conference abstract; FT – full-text article

AB: conference abstract; FT: full-text article; GI: gastrointestinal; MU: multiple-use (endoscope); N/A: not applicable; NR – not reported; NSC: non-screening colonoscopy; OGD: oesophagogastroduodenoscopy; RCT: randomised controlled trial; SC: screening colonoscopy; SU: single-use (endoscope)

3.3 Infection risk and adverse events

Supplementary Table 29. Adverse events and infection rates: Lower GI endoscopes and studies across different types of endoscope

Author, year, country, publication type [a], dates of procedures, trial registration	Single-use (SU) group – n / % non-infectious adverse events (AEs)	SU group – n / % infectious AEs	MU group – n / % non-infectious AEs	MU group – n / % infectious AEs
**SU endoscopes**				
Xu et al., 2024 (FT)<br><br>China<br><br>Dates of procedures: 05/2022 to 06/2022<br><br>Trial registration: ChiCTR2200060014	0 patients experienced mucosa damage, bowel perforation, massive haemorrhage, or instability of vital signs within 1 hour [b].*	None reported	0 patients experienced mucosa damage, bowel perforation, massive haemorrhage, or instability of vital signs within 1 hour [b].*	None reported
Wei et al., 2022 (FT)<br><br>China<br><br>Dates of procedures: 04/2021 to 04/2022<br><br>Trial registration: ChiCTR2100045084	2 patients (4%) experienced an AE within 1 month: 1 patient (2%) immediate bleeding (1 (5%) out of 19 EMRs), 1 (2%) delayed bleeding (1 (5%) of 19 endoscopic mucosal resections (EMRs)), 0% perforation.	None reported	2 patients (4%) experienced an AE within 1 month: 1 patient (2%) immediate bleeding (1 (6%) out of 17 EMRs), 1 (2%) delayed bleeding (1 (6%) of 17 EMRs), 0% perforation.	None reported
**MU endoscopes**				
Deb et al., 2022 (FT)<br><br>Multiple countries<br><br>Published by 10/2020<br><br>Trial registration: N/A	N/A	N/A	N/A	MU infection rate:<br><br>Overall post-GI procedures: 0·2%†, Post-ERCP procedures: 0·8% (77% of these sepsis and cholangitis), Post-non-ERCP upper GI endoscopic procedures: 0·123%, Post-lower-GI endoscopic procedures: 0·073%. Colonisation with MDRO reported in 32 of 1787 endoscope-associated infections.

[a] AB - conference abstract; FT – full-text article; [b] One hour is unlikely to be sufficient time for infectious adverse events to develop.

AB: conference abstract; EMR: endoscopic mucosal resections; ERCP: endoscopic retrograde cholangiopancreatography; FT: full-text article; GI: gastrointestinal; MDRO: multi-drug-resistant organisms; MU: multiple-use (endoscope); N/A: not applicable; NR – not reported; NSC: non-screening colonoscopy; OGD: oesophagogastroduodenoscopy; SAE: Serious Adverse Event; SC: screening colonoscopy; SU: single-use (endoscope).

*Not related to endoscope

†Percentages were rounded to the nearest integers, due to the low numbers of participants in the included studies. The exception was made in the case of those where rounding to the nearest integer would lead to 0% despite n being above 0, which would appear incorrect. Exceptions were also made for studies with thousands of patients; p values; confidence intervals; infection risk estimates; and diagnostic accuracy outcomes. This is consistent throughout the appendix.

3.4 Other outcomes

Supplementary Table 30. Clinical outcomes / Patient Reported Outcomes: Lower GI endoscopes and studies across different types of endoscope

Author, year, country, publication type [a], dates of procedures, trial registration	Clinical and/or patient reported outcomes
**SU endoscopes**	
Xu et al., 2024 (FT)<br><br>China<br><br>Dates of procedures: 05/2022 to 06/2022<br><br>Trial registration: ChiCTR2200060014	Postoperative Day 1<br>18 (86%) vs 20 (95%) no bleeding,<br>2 (10%) vs 1 (5%) bleeding<5ml,<br>1 (5%) vs 0 (0%) bleeding 5-10ml,<br>0 (%) vs 0 (0%) <10ml (p=·606).<br><br>16 (76%) vs 18 (86%) no urination disorder,<br>4 (19%) vs 3 (14%) have difficulty during urination,<br>1 (5%) vs 0 (0%) urinary retention (p=·697).<br><br>13 (62%) vs 12 (57%) no pain,<br>4 (19%) vs 7 (33%) mild pain,<br>3 (14%) vs 2 (10%) moderate pain, 1 (5%) vs 0 (0%) severe pain (p=·656).<br><br>Postoperative Day 10<br>20 (95%) vs 21 (100%) no bleeding,<br>1 (5%) vs 0 (0%) bleeding<5ml,<br>0 (0%) vs 0 (0%) bleeding 5-10ml, 0 (%) vs 0 (0%) <10ml (p=1·000).<br><br>21 (100%) vs 21 (100%) no urination disorder,<br>0 (0%) vs 0 (0%) have difficulty during urination,<br>0 (0%) vs 0 (0%) urinary retention (p=1·000).<br><br>20 (95%) vs 21 (100%) no pain,<br>1 (5%) vs 0 (0%) mild pain,<br>0 (0%) vs 0 (0%) moderate pain,<br>0 (0%) vs 0 (0%) severe pain (p=1·000).<br><br>In-procedure stability<br>Systolic blood pressure stability, 86% vs 95% (not significant),<br>Diastolic blood pressure stability 67% vs 95% (P = .049),<br>Heart rate stability<br>91% vs 90% (not significant).<br><br>10 ± 5 minutes after procedure:<br>Systolic blood pressure stability 81% vs 90% (not significant),<br>Diastolic blood pressure stability

	81% vs 81% (not significant),<br>Heart rate stability<br>86% vs 86% (not significant).<br><br>Therapeutic efficacy:<br>Postoperative Day 30<br>Cured: 12 (57%) vs 13 (62%),<br>Effective: 9 (43%) vs 7 (33%),<br>Invalid: 0 (0%) vs 1 (5%)(p=·61).
Wei et al., 2022 (FT)<br><br>China<br><br>Dates of procedures:<br>04/2021 to 04/2022<br><br>Trial registration:<br>ChiCTR2100045084	N/A
**MU endoscopes only**	
Deb et al., 2022 (FT)<br><br>Multiple countries<br><br>Published by 10/2020<br><br>Trial registration: N/A	N/A

[a] AB - conference abstract; FT – full-text article

AB: conference abstract; FT: full-text article; GI: gastrointestinal; MU: multiple-use (endoscope); N/A: not applicable; SU: single-use (endoscope).

Supplementary Table 31. Technical outcomes – Operator rating: Lower GI endoscopes and studies across different types of endoscope

Author, year, country, publication type [a], dates of procedures, trial registration	SU group vs MU group - Manoeuvres successful	SU group vs MU group - Operator rating: manoeuvres	SU group vs MU group - Operator rating: performance characteristics	SU group vs MU group - Operator rating: overall satisfaction
**SU endoscopes**				
Xu et al., 2024 (FT)<br><br>China<br><br>Dates of procedures:<br>05/2022 to 06/2022<br><br>Trial registration:<br>ChiCTR2200060014	N/A	N/A	Endoscope flexibility:<br>A Easily retroflexed from the forward position / B – Roughly retroflexed / C – Failed to be retroflexed<br><br>A: 16 (76%) vs 21 (100%),<br>B: 5 (24%) / 0 (0%),<br>C: 0 (0%) vs 0 (0%)(p=·06).<br><br>Clinical operability:<br>A Best - C Worst<br><br>A: 18 (86%) vs 21 (100%),<br>B: 3 (14%) vs 0 (0%),<br>C: 0 (0%) vs 0 (0%)(p=·231).	N/A

			Endoscope matching ligation device:<br>A Perfectly matched / B – Roughly matched / C – Failed procedure<br><br>A: 21 (100%) vs 21 (100%),<br>B: 0 (0%) vs 0 (0%),<br>C: 0 (0%) vs 0 (0%)<br>"not statistically significant".<br><br>Qualified ERBL feasibility:<br>21 (100%) vs 21 (100%).	
Wei et al., 2022 (FT)<br><br>China<br><br>Dates of procedures:<br>04/2021 to 04/2022<br><br>Trial registration:<br>ChiCTR2100045084	N/A	N/A	Clinical operability rated 'acceptable':<br><br>Flexibility<br>Body rigidity: 45 (100%) vs 45 (100%)(p=0·550),<br>Knob operation: 45 (100%) vs 45 (100%)(p=0·242),<br>Sharp angle adaptability: 45 (100%) vs 45 (100%)(p=0·556),<br>Air supply: 45 (100%) vs 45 (100%)(p=0·494).<br>Water supply: 45 (100%) vs 45 (100%)(p=1·000).<br>Suction: 45 (100%) vs 45 (100%)(p=1·000).	N/A
**MU endoscopes**				
Deb et al., 2022 (FT)<br><br>Multiple countries<br><br>Published by 10/2020<br><br>Trial registration:<br>N/A	N/A	N/A	N/A	N/A

[a] AB - conference abstract; FT – full-text article

AB: conference abstract; FT: full-text article; MU: multiple-use (endoscope); ERBL: endoscopic rubber band ligation; N/A: not applicable; NR – not reported; SU: single-use (endoscope).

Views of staff using, procuring, cleaning, and / or disposing: Lower GI endoscopes and studies across different types of endoscope

No included studies of lower GI endoscopes or studies across different types of endoscopes reported views of staff using, procuring, cleaning and/or disposing the endoscopes.
