# Supplementary material for: Diagnostic and Clinical Performance of Single‐Use Versus Reusable Gastrointestinal Endoscopes: A Systematic Review and Meta‐Analyses
Source: J Gastroenterol Hepatol. 2026 Aug 7;41(9):2571–87. doi: 10.1111/jgh.70637 (PMC13534234; doi:10.1111/jgh.70637)
Supplement: Supplementary file 7 [file JGH-41-2571-s007.pdf]

Diagnostic and clinical performance of single-use versus reusable gastrointestinal endoscopes: a systematic review and meta-analyses

Supporting information Table S4: Number and types of studies evaluating individual fully or partially single-use GI endoscopes

Authors

Natalie Tyldesley-Marshall, Yen-Fu Chen,* Jefferson Buendia, Anna Brown, Thomas Matthews, Janette Parr, Shivashri Chockalingam, Adel Elfeky, Amy Grove, Lazaros Andronis, Stuart R Coles, Mandana Zanganeh, Bu Hayee, Anjan Dhar, Shaji Sebastian, Ramesh Arasaradnam on behalf of the SUMU-Endo Project

*Correspondence to: Prof. Yen-Fu Chen

Centre for Evidence and Implementation Science, Park House, Edgbaston, University of Birmingham, Birmingham B15 2TT, United Kingdom

y.chen.25@bham.ac.uk

Department of Health and Welfare, College of City Management, University of Taipei, Taipei, Taiwan

yfchen@utaipei.edu.tw

Table S4. Number and types of studies evaluating individual fully or partially single-use GI endoscopes

Endoscope make/model	Fully paired DTA study	RCT	NRCS	Case series	Total
**Duodenoscopes**					
SU					
EXALT Model D (Olympus)	0	1	1 (6)	6 (1)	8 (7)
aScope (Ambu)	0	0	1 (1)	0	1 (1)
MUDC					
D34-i10T2 (Pentax)	0	1	0 (1)	0	1 (1)
TJF-Q190V (Olympus)	0	0	1	0	1
**Other upper GI endoscopes**					
Transnasal, ultra-thin, SU					
EG Scan III, II or I (IntroMedic)	7 (1)	0	0	2*	9 (1)
Unspecified model (EvoEndo)	0	0	0	0 (2)	0 (2)
Transnasal, ultra-thin – sheathed MU					
EndoSheath TNE–5000 (Vision Sciences)	1	1	0	1 (1)	3 (1)
Standard size - SU					
PR-IPD002 (Shenzhen PengRui)	0	1	0	0	1
ZING-W200B (Huizhou Xianzan)	0	1	0	0	1
aScope Gastro (Ambu)	0	0	0	1	1
Standard size – sheathed MU					
Sheath for standard size gastroscope (Shenda)	0	1	0 (1)	0	1 (1)
**Endoscopes for lower GI tract**					
SU gastroscope (for endoscopic rubber band ligation of internal haemorrhoids)					
EndoFresh XZING-W200B (HuiZhou Xzing Technology)	0	1	0	0	1
SU colonoscope					
XZING-C200 (HuiZhou Xzing Technology)	0	1	0	0	1

DTA: diagnostic test accuracy; MU: multiple-use; MUDC: multiple-use with disposable end cap; NRCS: Non-randomised comparative study; RCT: randomised controlled trial; SU: single-use.

Numbers shown in individual cells were number of studies published with full-text articles. Numbers shown in brackets were additional studies published only as conference abstracts.

*The endoscope was administered orally rather than through nose in one of the case series (Eqbal et al. 2021).
