# Supplementary material for: Diagnostic and Clinical Performance of Single‐Use Versus Reusable Gastrointestinal Endoscopes: A Systematic Review and Meta‐Analyses
Source: J Gastroenterol Hepatol. 2026 Aug 7;41(9):2571–87. doi: 10.1111/jgh.70637 (PMC13534234; doi:10.1111/jgh.70637)
Supplement: Supplementary file 9 [file JGH-41-2571-s009.pdf]

Diagnostic and clinical performance of single-use versus reusable gastrointestinal endoscopes: a systematic review and meta-analyses

Supporting information Data S2: Search strategies

Authors

Natalie Tyldesley-Marshall, Yen-Fu Chen,* Jefferson Buendia, Anna Brown, Thomas Matthews, Janette Parr, Shivashri Chockalingam, Adel Elfeky, Amy Grove, Lazaros Andronis, Stuart R Coles, Mandana Zanganeh, Bu Hayee, Anjan Dhar, Shaji Sebastian, Ramesh Arasaradnam on behalf of the SUMU-Endo Project

*Correspondence to: Prof. Yen-Fu Chen

Centre for Evidence and Implementation Science, Park House, Edgbaston, University of Birmingham, Birmingham B15 2TT, United Kingdom

y.chen.25@bham.ac.uk

Department of Health and Welfare, College of City Management, University of Taipei, Taipei, Taiwan

yfchen@utaipei.edu.tw

Search strategies

1. Overview of all searches undertaken

Original search May/July 2023		
**Database**	**Date searched**	**Number of records**
MEDLINE (via Ovid); pilot search	24/05/23	693
MEDLINE (via Ovid); finalised search strategy	20/07/23	871
Embase (Ovid)	20/07/23	1,857
Web of Science	20/07/23	986
Cochrane Library	20/07/23	220, of which:<br>• 220 trials (CENTRAL)<br>• 0 reviews (CDSR)
Global Index Medicus (WHO)	20/07/23	95
ClinicalTrials.gov	20/07/23	189
International Clinical Trials Registry Platform (WHO)	20/07/23	146
**Total number of records retrieved: 5,057**<br>**Duplicates removed (EndNote): 2,169**<br>**Final number for screening: 2,888**		
Targeted search for systematic reviews of infection risk		
**Database / search engine**	**Date searched**	**Number of records**
MEDLINE (Ovid) – systematic reviews only	22/01/24	122
Embase (Ovid) – systematic reviews only	22/01/24	134
**Total number of records retrieved: 256**<br>**Duplicates removed within this set (EndNote): 79**<br>**Final number for screening: 177** *(of which 13 already identified via scoping review search above)*		
Search update March 2024		
**Database**	**Date searched**	**Number of records**
MEDLINE (via Ovid)	04/03/24	69
Embase (Ovid)	04/03/24	152
Web of Science	04/03/24	87
Cochrane Library	04/03/24	15 (all CENTRAL)
Global Index Medicus (WHO)	04/03/24	99
ClinicalTrials.gov	04/03/24	200
International Clinical Trials Registry Platform (WHO)	04/03/24	179
**Total number of records retrieved: 801**<br>**Duplicates removed (EndNote, both within this set and against previous searches): 588**<br>**Final number for screening: 213**		
Search update September 2024		
**Database / source**	**Date searched**	**Number of records / documents identified**
MEDLINE (via Ovid)	25/09/24	69
Embase (Ovid)	25/09/24	163
Web of Science	25/09/24	95
Cochrane Library	25/09/24	19 (all CENTRAL)

Global Index Medicus (WHO)	26/09/24	103
ClinicalTrials.gov	26/09/24	216
International Clinical Trials Registry Platform (WHO)	26/09/24	200

Total number of records retrieved: 865
Duplicates removed (EndNote, both within this set and against previous searches): 496
Final number for screening: 369

2. Search strategies: original search May/July 2023

MEDLINE (Ovid) – pilot search

Date searched: 24/05/23
Ovid MEDLINE(R) ALL <1946 to May 23, 2023>
1 exp Endoscopes, Gastrointestinal/ or exp Endoscopy, Digestive System/ 127092
2 (colonoscop* or sigmoidoscop* or duodenoscop* or esophagoscop* or oesophagoscop* or gastroscop* or enteroscop* or digestive scope?).kf,tw. 54154
3 1 or 2 150617
4 exp Gastrointestinal Diseases/di 180728
5 exp Gastrointestinal Tract/ or exp Intestines/ or exp Lower Gastrointestinal Tract/ or exp Upper Gastrointestinal Tract/ 691584
6 (gastr* or esophag* or oesophag* or upper GI or lower GI or bowel* or colon* or duoden* or intestin* or stomach or digestive tract or endoscopic retrograde cholangi* or ERCP or ileum or ileal or jejun* or enteric).kf,tw. 1889164
7 (endoscop* or videoscop* or fiberscop* or fibrescop* or (fibre adj2 scope?) or (fiber adj2 scope?)).kf,tw. 251448
8 Endoscopes/ or Endoscopy/ 63525
9 4 or 5 or 6 2179009
10 7 or 8 268901
11 9 and 10 151165
12 3 or 11 [GI endoscopes] 231387
13 Equipment Reuse/ 3183
14 (reusable or reuse* or reprocessed or re usable or re use* or re processed or "multiple use" or "multi use").kf,tw. 36438
15 13 or 14 [MU / reusable] 38077
16 Disposable Equipment/ 5282
17 (disposable or "single use" or "one time use").kf,tw. 20205
18 16 or 17 [SU / disposable] 22448
19 12 and (15 or 18) [GI endoscopes; MU or SU] 693

MEDLINE (Ovid) – finalised

Date searched: 20/07/23
Ovid MEDLINE(R) ALL <1946 to July 19, 2023>
1 exp Endoscopes, Gastrointestinal/ or exp Endoscopy, Digestive System/ 127612
2 (colonoscop* or sigmoidoscop* or duodenoscop* or esophagoscop* or oesophagoscop* or gastroscop* or enteroscop* or digestive scope? or GI scope? or GI endoscop* or choledochoscop* or cholangioscop* or pancreatoscop* or capsule endoscop*).kf,tw. 62017
3 1 or 2 154479
4 exp Gastrointestinal Diseases/di, dg, su 410747
5 exp Gastrointestinal Tract/ 693308
6 (gastr* or esophag* or oesophag* or upper GI or lower GI or bowel* or colon or colonic or duoden* or intestin* or stomach or digestive tract or endoscopic retrograde cholangi* or ERCP or ileum or ileal or jejun* or enteric).kf,tw. 1622423
7 endoscop*.kf,tw. 251931
8 Endoscopes/ or Endoscopy/ 63872
9 4 or 5 or 6 1986309
10 7 or 8 269585
11 9 and 10 153579
12 3 or 11 [GI endoscopes] 235241
13 Equipment Reuse/ 3186
14 (reusable or reuse* or reprocessed or re usable or re use* or re processed or "multiple use" or "multi use").kf,tw. 37074
15 13 or 14 [MU / reusable] 38715
16 Disposable Equipment/ 5285
17 (disposable or "single use" or "one time use").kf,tw. 20400
18 16 or 17 [SU / disposable] 22645
19 Environment/ or Carbon Footprint/ 68775

20 Environmental Pollution/ or Air Pollution/ or Air Pollution, Indoor/ or exp "Environmental Restoration and Remediation"/ or exp Water Pollution/ or exp Environmental Pollutants/ 440771
21 exp Climate Change/ or exp Greenhouse Effect/ 35669
22 exp "Conservation of Natural Resources"/ 113772
23 (environment* adj3 (impact* or conservation or cost? or resource?)).kf,tw. 46183
24 (climate change or global warming or greenhouse effect or net zero or climate impact? or greenhouse gas*).kf,tw. 78987
25 (carbon adj3 (emission? or impact? or neutral* or footprint)).kf,tw. 13384
26 (co2 adj3 (emission? or impact? or neutral* or footprint)).kf,tw. 8137
27 pollut*.kf,tw. 206727
28 sustainab*.kf,ti. 34685
29 (landfill or (waste adj2 disposa*)).kf,tw. 11986
30 (life cycle assessment* or life cycle analys?s or "cradle to grave analys*" or circular economy).kf,tw. 8432
31 exp Waste Products/ or exp Waste Management/ 153875
32 (waste adj2 manage*).kf,tw. 9016
33 (green* adj2 (endoscopy or surgery or gastroenterology)).kf,tw. 131
34 recycl*.kf,tw. 67672
35 19 or 20 or 21 or 22 or 23 or 24 or 25 or 26 or 27 or 28 or 29 or 30 or 31 or 32 or 33 or 34 [environmental impact] 877210
36 12 and (15 or 18 or 35) [GI endoscopes + reusable or single use or environmental] 893
37 exp animals/ not humans/ 5140888
38 36 not 37 871

The MEDLINE search strategy was peer reviewed by Stevie van der Mierden, Information Specialist, Netherlands Cancer Institute and Maria Tan, University of Alberta, using the PRESS Checklist: PRESS Peer Review of Electronic Search Strategies. Last updated June 29, 2022. https://www.cadth.ca/press-peer-review-electronic-search-strategies-0

Embase (Ovid)

Date searched: 20/07/23
Embase Classic+Embase <1947 to 2023 July 19>
1 exp digestive endoscope/ or exp digestive tract endoscopy/ 276131
2 (colonoscop* or sigmoidoscop* or duodenoscop* or esophagoscop* or oesophagoscop* or gastroscop* or enteroscop* or digestive scope? or GI scope? or GI endoscop* or choledochoscop* or cholangioscop* or pancreatoscop* or capsule endoscop*).kf,tw. 120083
3 1 or 2 294261
4 exp digestive system disease/di, dm, su or exp digestive system/ 2530150
5 (gastr* or esophag* or oesophag* or upper GI or lower GI or bowel* or colon or colonic or duoden* or intestin* or stomach or digestive tract or endoscopic retrograde cholangi* or ERCP or ileum or ileal or jejun* or enteric).kf,tw. 2312937
6 4 or 5 3766722
7 endoscop*.kf,tw. 411100
8 endoscope/ or endoscopy/ 148124
9 7 or 8 449143
10 6 and 9 292271
11 3 or 10 [digestive system endoscopes] 453895
12 recycling/ 32708
13 (reusable or reuse* or reprocessed or re usable or re use* or re processed or "multiple use" or "multi use").kf,tw. 46800
14 12 or 13 [MU / reusable] 74887
15 disposable equipment/ 11200
16 (disposable or "single use" or "one time use").kf,tw. 28829
17 15 or 16 [SU / disposable] 32990
18 environmental economics/ or exp environmental impact/ or environmental impact assessment/ or life cycle assessment/ 121371
19 pollution/ or air pollution/ or exp greenhouse gas emission/ or indoor air pollution/ or exp nitrogen oxide emission/ or ozone depletion/ or exp plastic pollution/ or water pollution/ or acid rain/ or sea pollution/ or water contamination/ 239250

20 pollutant/ or air pollutant/ or exp atmospheric particulate matter/ or greenhouse gas/ or persistent organic pollutant/ or water pollutant/ 136000
21 waste/ or chemical waste/ or gas waste/ or packaging waste/ or exp hospital waste/ or exp plastic waste/ or solid waste/ or wastewater/ or hospital wastewater/ 37201
22 climate change/ 57709
23 environmental protection/ or energy conservation/ or environmental sustainability/ or water conservation/ 65430
24 (environment* adj3 (impact* or conservation or cost? or resource?)).kf,tw. 53875
25 (climate change or global warming or greenhouse effect or net zero or climate impact? or greenhouse gas*).kf,tw. 78291
26 (carbon adj3 (emission? or impact? or neutral* or footprint)).kf,tw. 13925
27 (co2 adj3 (emission? or impact? or neutral* or footprint)).kf,tw. 9005
28 pollut*.kf,tw. 264759
29 sustainab*.kf,ti. 36030
30 (landfill or (waste adj2 disposa*)).kf,tw. 17089
31 (life cycle assessment* or life cycle analys?s or "cradle to grave analys*" or circular economy).kf,tw. 10885
32 (waste adj2 manage*).kf,tw. 13233
33 waste management/ or waste minimization/ 26999
34 (green* adj2 (endoscopy or surgery or gastroenterology)).kf,tw. 182
35 recycl*.kf,tw. 80548
36 18 or 19 or 20 or 21 or 22 or 23 or 24 or 25 or 26 or 27 or 28 or 29 or 30 or 31 or 32 or 33 or 34 or 35 813215
37 11 and (14 or 17 or 36) 1894
38 (exp animal/ or exp animal experiment/) not (exp human/ or exp human experiment/ or conference abstract.pt.) 5790620
39 37 not 38 1857

Web of Science Core Collection

Date searched: 20/07/23
Editions: Science Citation Index Expanded (SCI-EXPANDED) 1970-present, Social Sciences Citation Index (SSCI) 1900-present, Conference Proceedings Citation Index – Science (CPCI-S) 1990-present, Conference Proceedings Citation Index – Social Science & Humanities (CPCI-SSH) 1990-present, Emerging Sources Citation Index (ESCI) 2015-present

1	TS=(colonoscop* OR sigmoidoscop* OR duodenoscop* OR esophagoscop* OR oesophagoscop* OR gastroscop* OR enteroscop* OR "digestive scope$" OR "GI scope$" OR "GI endoscop*" OR choledochoscop* OR cholangioscop* OR pancreatoscop* OR "capsule endoscop*")	73843
2	TS=(gastr* OR esophag* OR oesophag* OR "upper GI" OR "lower GI" OR bowel* OR colon OR colonic OR duoden* OR intestin* OR stomach OR "digestive tract" OR "endoscopic retrograde cholangi*" OR ERCP OR ileum OR ileal OR jejun* OR enteric)	1816762
3	TS=(endoscop*)	274619
4	#2 AND #3	144842
5	#1 OR #4	191538
6	TS=(recycl*)	242535
7	TS=(green* NEAR/1 (endoscopy OR surgery OR gastroenterology))	111
8	TS=(waste NEAR/1 manage*)	37234
9	TS=("life cycle assessment*" OR "life cycle analys$s" OR "cradle to grave analys*" OR "circular economy")	58058
10	TS=(landfill OR (waste NEAR/1 disposa*))	56333
11	TS=(pollut*)	613737
12	TS=(co2 NEAR/2 (emission$ OR impact$ OR neutral* OR footprint))	74348
13	TS=(carbon NEAR/2 (emission$ OR impact$ OR neutral* OR footprint))	89941

14	TS=("climate change" OR "global warming" OR "greenhouse effect" OR "net zero" OR "climate impact$" OR "greenhouse gas*")	464466
15	TS=(environment* NEAR/2 (impact* OR conservation OR cost$ OR resource$))	204631
16	((TI=(sustainab*)) OR AK=(sustainab*)) OR KP=(sustainab*)	300287
17	#6 OR #7 OR #8 OR #9 OR #10 OR #11 OR #12 OR #13 OR #14 OR #15 OR #16	1764528
18	#5 AND #17	113
19	TS=(reusable OR reuse* OR reprocessed OR "re usable" OR "re use*" OR "re processed" OR "multiple use" OR "multi use")	191690
20	TS=(disposable OR "single use" OR "one time use")	30795
21	#19 OR #20	219509
22	#5 AND #21	889
23	#18 OR #22	986

The Ovid Medline search strategy was translated for use in Web of Science with the aid of the Polyglot Search Translator: Clark JM, Sanders S, Carter M, Honeyman D, Cleo G, Auld Y, et al. Improving the translation of search strategies using the Polyglot Search Translator: a randomized controlled trial. J Med Libr Assoc 2020;108(2):195-207. http://dx.doi.org/10.5195/jmla.2020.834

Cochrane Central Register of Controlled Trials and Cochrane Database of Systematic Reviews (Cochrane Library, Wiley)

Date searched: 20/07/23
Search Screen: Advanced / search manager https://www.cochranelibrary.com/advanced-search/search-manager
#1 [mh "Endoscopes, Gastrointestinal"] OR [mh "Endoscopy, Digestive System"] 7331
#2 (colonoscop* OR sigmoidoscop* OR duodenoscop* OR esophagoscop* OR oesophagoscop* OR gastroscop* OR enteroscop* OR (digestive NEXT scope?) OR (GI NEXT scope?) OR (GI NEXT endoscop*) OR choledochoscop* OR cholangioscop* OR pancreatoscop* OR (capsule NEXT endoscop*)):ti,ab,kw 13697
#3 #1 OR #2 15837
#4 [mh "Gastrointestinal Diseases"/di] OR [mh "Gastrointestinal Diseases"/dg] OR [mh "Gastrointestinal Diseases"/su] 11780
#5 [mh "Gastrointestinal Tract"] 15457
#6 (gastr* OR esophag* OR oesophag* OR "upper GI" OR "lower GI" OR bowel* OR colon OR colonic OR duoden* OR intestin* OR stomach OR "digestive tract" OR ("endoscopic retrograde" NEXT cholangi*) OR ERCP OR ileum OR ileal OR jejun* OR enteric):ti,ab,kw 152147
#7 (endoscop*):ti,ab,kw 32939
#8 [mh ^Endoscopes] OR [mh ^Endoscopy] 2881
#9 #4 OR #5 OR #6 158693
#10 #7 OR #8 32939
#11 #9 AND #10 21385
#12 #3 OR #11 29084
#13 [mh "Equipment Reuse"] 114
#14 (reusable OR reuse* OR reprocessed OR "re usable" OR ("re" NEXT use*) OR "re processed" OR "multiple use" OR "multi use"):ti,ab,kw 1230
#15 #13 OR #14 1230
#16 [mh "Disposable Equipment"] 354
#17 (disposable OR "single use" OR "one time use"):ti,ab,kw 3696
#18 #16 OR #17 3696
#19 [mh ^Environment] OR [mh ^"Carbon Footprint"] 795
#20 [mh ^"Environmental Pollution"] OR [mh ^"Air Pollution"] OR [mh ^"Air Pollution, Indoor"] OR [mh "Environmental Restoration and Remediation"] OR [mh "Water Pollution"] OR [mh "Environmental Pollutants"] 1302
#21 [mh "Climate Change"] OR [mh "Greenhouse Effect"] 48
#22 [mh "Conservation of Natural Resources"] 197
#23 [mh "Waste Products"] OR [mh "Waste Management"] 220

#24 (sustainab*):ti,kw 636
#25 (environment* NEAR/3 (impact* OR conservation OR cost? OR resource?)):ti,ab,kw 739
#26 ("climate change" OR "global warming" OR "greenhouse effect" OR "net zero" OR (climate NEXT impact?) OR (greenhouse NEXT gas*)):ti,ab,kw 248
#27 (carbon NEAR/3 (emission? OR impact? OR neutral* OR footprint)):ti,ab,kw 91
#28 (co2 NEAR/3 (emission? OR impact? OR neutral* OR footprint)):ti,ab,kw 33
#29 (pollut*):ti,ab,kw 2620
#30 (landfill OR (waste NEAR/2 disposa*)):ti,ab,kw 73
#31 (("life cycle" NEXT (assessment* OR analys?s)) OR ("cradle to grave" NEXT analys*) OR "circular economy"):ti,ab,kw 13
#32 (waste NEAR/2 manage*):ti,ab,kw 115
#33 (green* NEAR/2 (endoscopy OR surgery OR gastroenterology)):ti,ab,kw 12
#34 (recycl*):ti,ab,kw 456
#35 #19 OR #20 OR #21 OR #22 OR #23 OR #24 OR #25 OR #26 OR #27 OR #28 OR #29 OR #30 OR #31 OR #32 OR #33 OR #34 5824
#36 #15 OR #18 OR #35 10292
#37 #12 AND #36 220
All results from CENTRAL; 0 from CDSR

The Ovid Medline search strategy was translated for use in Cochrane Library with the aid of the Polyglot Search Translator: Clark JM, Sanders S, Carter M, Honeyman D, Cleo G, Auld Y, et al. Improving the translation of search strategies using the Polyglot Search Translator: a randomized controlled trial. J Med Libr Assoc 2020;108(2):195-207. http://dx.doi.org/10.5195/jmla.2020.834

Global Index Medicus

Date searched: 20/07/23
Databases: All the Regional Indexes Medici: African Index Medicus (AIM), Index Medicus for the Eastern Mediterranean Region (IMEMR), Index Medicus for the South-East Asia Region (IMSEAR), Latin America and the Caribbean Literature on Health Sciences (LILACS), Western Pacific Region Index Medicus (WPRO)
Search screen: Advanced, available at: https://search.bvsalud.org/gim/advanced/?lang=en

Search strategy: (note tw fields are Title, Abstract, Subject):
tw:((tw:(colonoscop* OR sigmoidoscop* OR duodenoscop* OR esophagoscop* OR oesophagoscop* OR gastroscop* OR enteroscop* OR "digestive scope" OR "digestive scopes" OR "GI scope" OR "GI scopes" OR "GI endoscope" OR "GI endoscopy" OR "GI endoscopes" OR "GI endoscopies" OR choledochoscop* OR cholangioscop* OR pancreatoscop* OR "capsule endoscope" OR "capsule endoscopes" OR "capsule endoscopy" OR "capsule endoscopies" OR ((gastr* OR esophag* OR oesophag* OR "upper GI" OR "lower GI" OR bowel* OR colon OR colonic OR duoden* OR intestin* OR stomach OR "digestive tract" OR "digestive system" OR "endoscopic retrograde cholangiopancreatography" OR ercp OR ileum OR ileal OR jejun* OR enteric) AND endoscop*))) AND (tw:(reusable OR reuse* OR reprocessed OR "re usable" OR "re use" OR "re used" OR "re processed" OR "multiple use" OR "multi use" OR disposable OR "single use" OR "one time use" OR sustainab* OR "environmental impact" OR "environmental impacts" OR "environmental conservation" OR "environmental cost" OR "environmental costs" OR "environmental resources" OR "natural resources" OR "climate change" OR "global warming" OR "greenhouse effect" OR "net zero" OR "climate impact" OR "climate impacts" OR "greenhouse gas" OR "greenhouse gases" OR "carbon emissions" OR "carbon neutral" OR "carbon neutrality" OR "carbon footprint" OR "co2 emissions" OR pollut* OR landfill OR "waste disposal" OR "disposable waste" OR "disposal of waste" OR "life cycle assessment" OR "life cycle analysis" OR "cradle to grave analysis" OR "circular economy" OR "waste management" OR "green endoscopy" OR "greener endoscopy" OR "green surgery" OR "greener surgery" OR "green gastroenterology" OR "greener gastroenterology" OR recycl*)))
95 results

ClinicalTrials.gov

Date searched: 20/07/23

Search screen: home page, classic https://classic.clinicaltrials.gov/ct2/home
'Other terms' box used for all searches

1.
(endoscopy OR colonoscopy OR sigmoidoscopy OR duodenoscopy OR esophagoscopy OR oesophagoscopy OR gastroscopy OR enteroscopy OR choledochoscopy OR cholangioscopy OR pancreatoscopy) AND (reusable OR "multiple use" OR "multi use") 27 results

2.
(endoscopy OR colonoscopy OR sigmoidoscopy OR duodenoscopy OR esophagoscopy OR oesophagoscopy OR gastroscopy OR enteroscopy OR choledochoscopy OR cholangioscopy OR pancreatoscopy) AND (disposable OR "single use" OR "one time use") 144 results

3.
(endoscopy OR colonoscopy OR sigmoidoscopy OR duodenoscopy OR esophagoscopy OR oesophagoscopy OR gastroscopy OR enteroscopy OR choledochoscopy OR cholangioscopy OR pancreatoscopy) AND ("environmental impact" OR climate OR sustainable) 12 results

4.
(endoscopy OR colonoscopy OR sigmoidoscopy OR duodenoscopy OR esophagoscopy OR oesophagoscopy OR gastroscopy OR enteroscopy OR choledochoscopy OR cholangioscopy OR pancreatoscopy) AND (sustainability OR "carbon footprint" OR pollution OR pollutant) 8 results

5.
(endoscopy OR colonoscopy OR sigmoidoscopy OR duodenoscopy OR esophagoscopy OR oesophagoscopy OR gastroscopy OR enteroscopy OR choledochoscopy OR cholangioscopy OR pancreatoscopy) AND ("waste disposal" OR "waste management" OR recycling) 5 studies

6.
(endoscope OR colonoscope OR sigmoidoscope OR duodenoscope OR esophagoscope OR oesophagoscope OR gastroscope OR enteroscope OR choledochoscope OR cholangioscope OR pancreatoscope) AND (reusable OR "multiple use" OR "multi use") 24 results

7.
(endoscope OR colonoscope OR sigmoidoscope OR duodenoscope OR esophagoscope OR oesophagoscope OR gastroscope OR enteroscope OR choledochoscope OR cholangioscope OR pancreatoscope) AND (disposable OR "single use" OR "one time use") 83 results

8.
(endoscope OR colonoscope OR sigmoidoscope OR duodenoscope OR esophagoscope OR oesophagoscope OR gastroscope OR enteroscope OR choledochoscope OR cholangioscope OR pancreatoscope) AND ("environmental impact" OR climate OR sustainable) 2 results

9.
(endoscope OR colonoscope OR sigmoidoscope OR duodenoscope OR esophagoscope OR oesophagoscope OR gastroscope OR enteroscope OR choledochoscope OR cholangioscope OR pancreatoscope) AND (sustainability OR "carbon footprint" OR pollution OR pollutant) 0 results

10.
(endoscope OR colonoscope OR sigmoidoscope OR duodenoscope OR esophagoscope OR oesophagoscope OR gastroscope OR enteroscope OR choledochoscope OR cholangioscope OR pancreatoscope) AND ("waste disposal" OR "waste management" OR recycling) 1 result

Total: 306 results
Total after deduplication across searches (using Endnote 20): 189

International Clinical Trials Registry Platform (WHO ICTRP)

Date searched: 20/07/23
Search screen: basic/home page https://trialsearch.who.int/

1.
(endoscop* OR colonoscop* OR sigmoidoscop* OR duodenoscop* OR esophagoscop* OR oesophagoscop* OR gastroscop* OR enteroscop* OR choledochoscop* OR cholangioscop* OR pancreatoscop*) AND (reusable OR multiple use OR multi use) 34 results

2.
(endoscop* OR colonoscop* OR sigmoidoscop* OR duodenoscop* OR esophagoscop* OR oesophagoscop* OR gastroscop* OR enteroscop* OR choledochoscop* OR cholangioscop* OR pancreatoscop*) AND (disposable OR single use OR one time use) 125 results

3.
(endoscop* OR colonoscop* OR sigmoidoscop* OR duodenoscop* OR esophagoscop* OR oesophagoscop* OR gastroscop* OR enteroscop* OR choledochoscop* OR cholangioscop* OR pancreatoscop*) AND (environmental impact OR climate OR sustainable OR sustainability OR carbon footprint OR pollution OR pollutant OR waste disposal OR waste management OR recycling) 1 result

Total: 160 results
Total after deduplication across searches (using Endnote 20): 146 results

Websites (for guidance, position statements, policy documents)

Note: only guidance or policy documents published 2018-2023 and judged to be potentially relevant to the review topic were retrieved

U.S. Food and Drug Administration (FDA): Search for FDA Guidance Documents
https://www.fda.gov/regulatory-information/search-fda-guidance-documents
Date searched: 25/09/23

Terms entered in search box:

endosco	3 results	all too old
colonosc	1 result	not relevant (bowel cleansing)
sigmoidosc	0 results	
duodenosc	0 results	
scope	9 results	0 relevant / in date
esophagosc	0 results	
gastrosc	0 results	

Total: 0 documents retrieved

Medicines and Healthcare products Regulatory Agency (MHRA)
https://www.gov.uk/government/organisations/medicines-and-healthcare-products-regulatory-agency
Date searched: 25/09/23

Browsed to:
Alerts, recalls and safety information: drugs and medical devices https://www.gov.uk/drug-device-alerts

Terms entered in search box, limited to Message type ‘National patient safety alert’ or ‘Device safety information’; Issued after 2018:

endoscope	1 result	0 relevant
endoscopy	0 results	
colonoscope	0 results	
sigmoidoscope	0 results	
duodenoscope	1 result	0 relevant
scope	3 results	0 relevant
esophagoscope	0 results	
gastroscope	0 results	

All MHRA services and information > Medical devices regulation and safety > Regulating medical devices in the UK https://www.gov.uk/guidance/regulating-medical-devices-in-the-uk

Ctrl + F for:
scop 0 results
green
enviro 1 result, not relevant

Medical devices: guidance for manufacturers on vigilance
https://www.gov.uk/government/collections/medical-devices-guidance-for-manufacturers-on-vigilance
Ctrl + F for:
scop 0 results
green
enviro 1 result, not relevant

Total: 0 documents retrieved

National Institute for Health and Care Excellence (NICE) https://www.nice.org.uk/
Date searched: 25/09/23
Terms entered in search box (results sorted by date for easy exclusion of pre-2018); Filtered by type 'Guidance', 'NICE advice' OR 'Qulaity standards':

endoscop*	237 results	0 downloaded
colonoscop*	30 results	1 downloaded
sigmoidoscop*	12 results	0 downloaded
duodenoscop*	2 results	0 downloaded
gastrointestinal scope	89 results	0 downloaded
oesophagoscope*	1 result	0 downloaded
gastroscop*	4 results	0 downloaded
green	71 results	0 downloaded
"single use"	143 results	0 downloaded
disposable	101 results	0 downloaded

Total: 1 document retrieved

The British Society of Gastroenterology https://www.bsg.org.uk/
Date searched: 26/09/23
Scanned all titles published since 2018 on the following pages:
https://www.bsg.org.uk/resource-type/clinical-resources/guidelines/ 1 downloaded
https://www.bsg.org.uk/resource-type/clinical-resources/guidance/ 1 downloaded
>British Society of Paediatric Gastroenterology, Hepatology and Nutrition *see below*
>European Society for Paediatric Gastroenterology, Hepatology and Nutrition *see below*
http://bsg.org.uk/resource-type/clinical-resources/position-statements/ 0 downloaded

Total: 2 documents retrieved

British Society of Paediatric Gastroenterology, Hepatology and Nutrition (BSPGHAN): Gastroenterology Guidelines https://bspghan.org.uk/gastroenterologyguidelines
Date searched: 26/09/23
Scanned all titles. 0 downloaded
Total: 0 documents retrieved

European Society for Paediatric Gastroenterology, Hepatology and Nutrition (ESPGHAN) https://www.espghan.org/home
Date searched: 26/09/23
Browsed to Knowledge Center > Publications > Gastroenterology and scanned all titles published since 2018 0 downloaded
Total: 0 documents retrieved

The European Society of Gastrointestinal Endoscopy (ESGE) https://www.esge.com/
Date searched: 26/09/23
Scanned all titles published since 2018 on the following pages:

https://www.esge.com/publications/guidelines/ (all topics)1 downloaded
https://www.esge.com/publications/position-statements/ 2 downloaded
Total: 3 documents retrieved

The American Society for Gastrointestinal Endoscopy https://www.asge.org/
Date searched: 26/09/23
Scanned all titles published since 2018 on the following pages:
https://www.asge.org/home/resources/key-resources/guidelines 3 downloaded, + 1 FDA news release referenced in one of the guidelines
https://www.asge.org/home/resources/key-resources/sustainable-endoscopy 1 downloaded
Total: 5 documents retrieved

World Endoscopy Organisation (WEO) https://www.worldendo.org/
Date searched: 26/09/23
Browsed to Publications > Guidelines. 0 downloaded
Total: 0 documents retrieved

The European Association for Endoscopic Surgery (EAES) https://eaes.eu/
Date searched: 26/09/23
Scanned all titles on this page: https://eaes.eu/about-eaes/committees/research/consensus-guideline-subcommittee/ 0 downloaded
Browsed to Library > Document library ; 10 documents found, 0 relevant
Total: 0 documents retrieved

Japan Gastroenterological Endoscopy Society https://www.jges.net/english
Date searched: 26/09/23
Scanned all titles published since 2018 on this page:
https://www.jges.net/english/publication/guideline 0 downloaded
Total: 0 documents retrieved

The European Society of Gastroenterology and Endoscopy Nurses and Associates (ESGENA) https://esgena.org/
Date searched: 26/09/23
Browsed to Publications > ESGE-ESGENA-Guidelines and Position Statements, which redirects to the ESGE website already searched above 0 downloaded
Total: 0 documents retrieved

Total: 11 documents retrieved from websites search

3. Search strategies: targeted search for systematic reviews of infection risk

MEDLINE (Ovid)

Date searched: 22/01/24
Ovid MEDLINE(R) ALL <1946 to January 19, 2024>

1 Meta-Analysis as Topic/ 23661
2 meta analy$.tw. 290721
3 metaanaly$.tw. 2657
4 Meta-Analysis/ 193538
5 (systematic adj (review$1 or overview$1)).tw. 314613
6 exp Review Literature as Topic/ 24344
7 or/1-6 484909
8 cochrane.ab. 143768
9 embase.ab. 165844
10 web of science.ab. 85181
11 scopus.ab. 63102
12 (cinahl or cinhal).ab. 49467
13 science citation index.ab. 3896
14 google scholar.ab. 36727
15 (sciencedirect or science direct).ab. 14391
16 or/8-15 282199
17 reference list$.ab. 22836
18 bibliograph$.ab. 23361
19 hand-search$.ab. 8838
20 relevant journals.ab. 1379
21 manual search$.ab. 6358
22 or/17-21 56417
23 selection criteria.ab. 37172
24 data extraction.ab. 34792
25 23 or 24 69213
26 Review/ 3266087
27 25 and 26 36489
28 Comment/ 1029522
29 Letter/ 1240923
30 Editorial/ 679403
31 animal/ 7376566
32 human/ 21721589
33 31 not (31 and 32) 5154582
34 or/28-30,33 7299520
35 7 or 16 or 22 or 27 575092
36 35 not 34 [SIGN filter for systematic reviews, lines 11-15 adapted] 548526
37 exp Infections/et, tm [Etiology, Transmission] 374477
38 Cross Infection/ 61798
39 Disease Transmission, Infectious/ or Infectious Disease Transmission, Patient-to-Professional/ or Infectious Disease Transmission, Professional-to-Patient/ 17794
40 (transmission or cross infection).kf,tw. 476607
41 (infectio* adj3 (risk or iatrogenic or nosocomial or hospital acquired)).kf,tw. 97060
42 ((endoscop* related or procedure related) adj1 (infectio* or event* or complication*)).kf,tw. 4920
43 37 or 38 or 39 or 40 or 41 or 42 895865
44 exp Endoscopes, Gastrointestinal/ or exp Endoscopy, Digestive System/ or Endoscopes/ or Endoscopy/ 189996
45 (endoscop* or colonoscop* or sigmoidoscop* or duodenoscop* or gastroscop* or enteroscop* or digestive scope? or GI scope? or endoscopic retrograde cholangi* or ERCP).kf,tw. 295617
46 44 or 45 [endoscopes or GI endoscopes; trying to include endoscopes in general but not broncho-, laparo-, etc] 349434
47 36 and 43 and 46 207
48 limit 47 to yr="2018 -Current" 122

The search filter for systematic reviews (lines 1-36) has been adapted from: Scottish Intercollegiate Guidelines Network (SIGN). Search filters: systematic reviews. 2021. URL: https://www.sign.ac.uk/what-we-do/methodology/search-filters/ (accessed 22/01/24).

Embase (Ovid)

Date searched: 22/01/24
Embase <1974 to 2024 January 19>

1 exp Meta Analysis/ 305477
2 ((meta adj analy$) or metaanalys$).tw. 371564
3 (systematic adj (review$1 or overview$1)).tw. 379345
4 or/1-3 607125
5 scopus.ab. 72589
6 cochrane.ab. 181589
7 embase.ab. 205884
8 web of science.ab. 95636
9 google scholar.ab. 44116
10 (cinahl or cinhal).ab. 57499
11 science citation index.ab. 4468
12 (sciencedirect or science direct).ab. 17768
13 or/5-12 348854
14 reference lists.ab. 24404
15 bibliograph$.ab. 30016
16 hand-search$.ab. 10786
17 manual search$.ab. 7432
18 relevant journals.ab. 1630
19 or/14-18 67125
20 data extraction.ab. 42106
21 selection criteria.ab. 46296
22 20 or 21 85643
23 review.pt. 3177859
24 22 and 23 39319
25 letter.pt. 1305332
26 editorial.pt. 793961
27 animal/ 1649538
28 human/ 25952080
29 27 not (27 and 28) 1210475
30 or/25-26,29 3291157
31 4 or 13 or 19 or 24 724569
32 31 not 30 [SIGN filter for Systematic reviews; lines 5-12 edited] 707322
33 pathogen transmission/ or bacterial transmission/ 16339
34 disease transmission/ 115096
35 device infection/ or infectious complication/ or cross infection/ or exp nosocomial transmission/ or infection risk/ or hospital infection/ 196838
36 (transmission or cross infection).kf,tw. 528857
37 (infectio* adj3 (risk or iatrogenic or nosocomial or hospital acquired)).kf,tw. 136444
38 ((endoscop* related or procedure related) adj1 (infectio* or event* or complication*)).kf,tw. 8519
39 33 or 34 or 35 or 36 or 37 or 38 856553
40 exp digestive endoscope/ or exp digestive tract endoscopy/ or endoscope/ or endoscopy/ 399380
41 (endoscop* or colonoscop* or sigmoidoscop* or duodenoscop* or gastroscop* or enteroscop* or digestive scope? or GI scope? or endoscopic retrograde cholangi* or ERCP).kf,tw. 482057
42 40 or 41 605118
43 32 and 39 and 42 334
44 (conference abstract or conference review).pt. 5043620
45 43 not 44 227
46 limit 45 to yr="2018 -Current" 134

The search filter for systematic reviews (lines 1-32) has been adapted from: Scottish Intercollegiate Guidelines Network (SIGN). Search filters: systematic reviews. 2021. URL: https://www.sign.ac.uk/what-we-do/methodology/search-filters/ (accessed 22/01/24).

4. Search strategies: Search update March 2024

MEDLINE (Ovid)

Date searched: 04/03/24
Ovid MEDLINE(R) ALL <1946 to March 01, 2024>

1 exp Endoscopes, Gastrointestinal/ or exp Endoscopy, Digestive System/ 129607
2 (colonoscop* or sigmoidoscop* or duodenoscop* or esophagoscop* or oesophagoscop* or gastroscop* or enteroscop* or digestive scope? or GI scope? or GI endoscop* or choledochoscop* or cholangioscop* or pancreatoscop* or capsule endoscop*).kf,tw. 63605
3 1 or 2 157259
4 exp Gastrointestinal Diseases/di, dg, su 418205
5 exp Gastrointestinal Tract/ 699649
6 (gastr* or esophag* or oesophag* or upper GI or lower GI or bowel* or colon or colonic or duoden* or intestin* or stomach or digestive tract or endoscopic retrograde cholangi* or ERCP or ileum or ileal or jejun* or enteric).kf,tw. 1657870
7 endoscop*.kf,tw. 258643
8 Endoscopes/ or Endoscopy/ 65112
9 4 or 5 or 6 2025952
10 7 or 8 276357
11 9 and 10 157272
12 3 or 11 [GI endoscopes] 240236
13 Equipment Reuse/ 3208
14 (reusable or reuse* or reprocessed or re usable or re use* or re processed or "multiple use" or "multi use").kf,tw. 38742
15 13 or 14 [MU / reusable] 40387
16 Disposable Equipment/ 5305
17 (disposable or "single use" or "one time use").kf,tw. 21089
18 16 or 17 [SU / disposable] 23334
19 Environment/ or Carbon Footprint/ 69482
20 Environmental Pollution/ or Air Pollution/ or Air Pollution, Indoor/ or exp "Environmental Restoration and Remediation"/ or exp Water Pollution/ or exp Environmental Pollutants/ 452834
21 exp Climate Change/ or exp Greenhouse Effect/ 37562
22 exp "Conservation of Natural Resources"/ 116512
23 (environment* adj3 (impact* or conservation or cost? or resource?)).kf,tw. 49520
24 (climate change or global warming or greenhouse effect or net zero or climate impact? or greenhouse gas*).kf,tw. 85266
25 (carbon adj3 (emission? or impact? or neutral* or footprint)).kf,tw. 15182
26 (co2 adj3 (emission? or impact? or neutral* or footprint)).kf,tw. 8855
27 pollut*.kf,tw. 218468
28 sustainab*.kf,ti. 38212
29 (landfill or (waste adj2 disposa*)).kf,tw. 12469
30 (life cycle assessment* or life cycle analys?s or "cradle to grave analys*" or circular economy).kf,tw. 9353
31 exp Waste Products/ or exp Waste Management/ 158806
32 (waste adj2 manage*).kf,tw. 9640
33 (green* adj2 (endoscopy or surgery or gastroenterology)).kf,tw. 149
34 recycl*.kf,tw. 70642
35 19 or 20 or 21 or 22 or 23 or 24 or 25 or 26 or 27 or 28 or 29 or 30 or 31 or 32 or 33 or 34 [environmental impact] 911749
36 12 and (15 or 18 or 35) [GI endoscopes + reusable or single use or environmental] 943
37 exp animals/ not humans/ 5200495
38 36 not 37 921
39 (2023072* or 2023073* or 202308* or 202309* or 20231* or 2024*).dt,ez,da,cb. 927855
40 38 and 39 69

Embase (Ovid)

Date searched: 04/03/24
Embase <1974 to 2024 March 01>

1 exp digestive endoscope/ or exp digestive tract endoscopy/ 279751
2 (colonoscop* or sigmoidoscop* or duodenoscop* or esophagoscop* or oesophagoscop* or gastroscop* or enteroscop* or digestive scope? or GI scope? or GI endoscop* or choledochoscop* or cholangioscop* or pancreatoscop* or capsule endoscop*).kf,tw. 120092
3 1 or 2 297368
4 exp digestive system disease/di, dm, su or exp digestive system/ 2212827
5 (gastr* or esophag* or oesophag* or upper GI or lower GI or bowel* or colon or colonic or duoden* or intestin* or stomach or digestive tract or endoscopic retrograde cholangi* or ERCP or ileum or ileal or jejun* or enteric).kf,tw. 2169761
6 4 or 5 3426958
7 endoscop*.kf,tw. 421426
8 endoscope/ or endoscopy/ 150262
9 7 or 8 459012
10 6 and 9 300073
11 3 or 10 [digestive system endoscopes] 461290
12 recycling/ 34176
13 (reusable or reuse* or reprocessed or re usable or re use* or re processed or "multiple use" or "multi use").kf,tw. 48682
14 12 or 13 [MU / reusable] 78000
15 disposable equipment/ 11234
16 (disposable or "single use" or "one time use").kf,tw. 28724
17 15 or 16 [SU / disposable] 32872
18 environmental economics/ or exp environmental impact/ or environmental impact assessment/ or life cycle assessment/ 129235
19 pollution/ or air pollution/ or exp greenhouse gas emission/ or indoor air pollution/ or exp nitrogen oxide emission/ or ozone depletion/ or exp plastic pollution/ or water pollution/ or acid rain/ or sea pollution/ or water contamination/ 232433
20 pollutant/ or air pollutant/ or exp atmospheric particulate matter/ or greenhouse gas/ or persistent organic pollutant/ or water pollutant/ 141676
21 waste/ or chemical waste/ or gas waste/ or packaging waste/ or exp hospital waste/ or exp plastic waste/ or solid waste/ or wastewater/ or hospital wastewater/ 39760
22 climate change/ 63196
23 environmental protection/ or energy conservation/ or environmental sustainability/ or water conservation/ 68285
24 (environment* adj3 (impact* or conservation or cost? or resource?)).kf,tw. 57935
25 (climate change or global warming or greenhouse effect or net zero or climate impact? or greenhouse gas*).kf,tw. 85731
26 (carbon adj3 (emission? or impact? or neutral* or footprint)).kf,tw. 16078
27 (co2 adj3 (emission? or impact? or neutral* or footprint)).kf,tw. 9807
28 pollut*.kf,tw. 269191
29 sustainab*.kf,ti. 40237
30 (landfill or (waste adj2 disposa*)).kf,tw. 17097
31 (life cycle assessment* or life cycle analys?s or "cradle to grave analys*" or circular economy).kf,tw. 12046
32 (waste adj2 manage*).kf,tw. 13805
33 waste management/ or waste minimization/ 26904
34 (green* adj2 (endoscopy or surgery or gastroenterology)).kf,tw. 209
35 recycl*.kf,tw. 83648
36 18 or 19 or 20 or 21 or 22 or 23 or 24 or 25 or 26 or 27 or 28 or 29 or 30 or 31 or 32 or 33 or 34 or 35 831996
37 11 and (14 or 17 or 36) 2022
38 (exp animal/ or exp animal experiment/) not (exp human/ or exp human experiment/ or conference abstract.pt.) 5089224
39 37 not 38 1984
40 limit 39 to dc=20230720-20241231 152

Web of Science Core Collection

Date searched: 04/03/24

Editions: Science Citation Index Expanded (SCI-EXPANDED) 1970-present, Social Sciences Citation Index (SSCI) 1900-present, Conference Proceedings Citation Index – Science (CPCI-S) 1990-present, Conference Proceedings Citation Index – Social Science & Humanities (CPCI-SSH) 1990-present, Emerging Sources Citation Index (ESCI) 2015-present

Search Query	Results
TS=(colonoscop* OR sigmoidoscop* OR duodenoscop* OR esophagoscop* OR oesophagoscop* OR gastroscop* OR enteroscop* OR "digestive scope$" OR "GI scope$" OR "GI endoscop*" OR choledochoscop* OR cholangioscop* OR pancreatoscop* OR "capsule endoscop*")	77017
TS=(gastr* OR esophag* OR oesophag* OR "upper GI" OR "lower GI" OR bowel* OR colon OR colonic OR duoden* OR intestin* OR stomach OR "digestive tract" OR "endoscopic retrograde cholangi*" OR ERCP OR ileum OR ileal OR jejun* OR enteric)	1880371
TS=(endoscop*)	286787
#2 AND #3	150927
#1 OR #4	199648
TS=(recycl*) Editions: WOS.SCI,WOS.SSCI,WOS.ISTP,WOS.ISSHP,WOS.ESCI	258505
TS=(green* NEAR/1 (endoscopy OR surgery OR gastroenterology)) Editions: WOS.SCI,WOS.SSCI,WOS.ISTP,WOS.ISSHP,WOS.ESCI	140
TS=(waste NEAR/1 manage*) Editions: WOS.SCI,WOS.SSCI,WOS.ISTP,WOS.ISSHP,WOS.ESCI	40299
TS=("life cycle assessment*" OR "life cycle analys$s" OR "cradle to grave analys*" OR "circular economy") Editions: WOS.SCI,WOS.SSCI,WOS.ISTP,WOS.ISSHP,WOS.ESCI	64888
TS=(landfill OR (waste NEAR/1 disposa*)) Editions: WOS.SCI,WOS.SSCI,WOS.ISTP,WOS.ISSHP,WOS.ESCI	59166
TS=(pollut*) Editions: WOS.SCI,WOS.SSCI,WOS.ISTP,WOS.ISSHP,WOS.ESCI	653810
TS=(co2 NEAR/2 (emission$ OR impact$ OR neutral* OR footprint)) Editions: WOS.SCI,WOS.SSCI,WOS.ISTP,WOS.ISSHP,WOS.ESCI	81513
TS=(carbon NEAR/2 (emission$ OR impact$ OR neutral* OR footprint)) Editions: WOS.SCI,WOS.SSCI,WOS.ISTP,WOS.ISSHP,WOS.ESCI	103175
TS=("climate change" OR "global warming" OR "greenhouse effect" OR "net zero" OR "climate impact$" OR "greenhouse gas*") Editions: WOS.SCI,WOS.SSCI,WOS.ISTP,WOS.ISSHP,WOS.ESCI	503246
TS=(environment* NEAR/2 (impact* OR conservation OR cost$ OR resource$)) Editions: WOS.SCI,WOS.SSCI,WOS.ISTP,WOS.ISSHP,WOS.ESCI	221213
((TI=(sustainab*)) OR AK=(sustainab*)) OR KP=(sustainab*) Editions: WOS.SCI,WOS.SSCI,WOS.ISTP,WOS.ISSHP,WOS.ESCI	330651
#6 OR #7 OR #8 OR #9 OR #10 OR #11 OR #12 OR #13 OR #14 OR #15 OR #16 Editions: WOS.SCI,WOS.SSCI,WOS.ISTP,WOS.ISSHP,WOS.ESCI	1896421
#5 AND #17 Editions: WOS.SCI,WOS.SSCI,WOS.ISTP,WOS.ISSHP,WOS.ESCI	150
TS=(reusable OR reuse* OR reprocessed OR "re usable" OR "re use*" OR "re processed" OR "multiple use" OR "multi use")	201335
TS=(disposable OR "single use" OR "one time use")	32531
#19 OR #20	230681
#5 AND #21	952

#18 OR #22 Editions: WOS.ESCI,WOS.ISSHP,WOS.ISTP,WOS.SCI,WOS.SSCI Timespan: 2023-07-20 to 2024-12-31 (Index Date)	87

Cochrane Central Register of Controlled Trials and Cochrane Database of Systematic Reviews (Cochrane Library, Wiley)

Date searched: 04/03/24
Search Screen: Advanced / search manager https://www.cochranelibrary.com/advanced-search/search-manager

#1 [mh "Endoscopes, Gastrointestinal"] OR [mh "Endoscopy, Digestive System"] 7658
#2 (colonoscop* OR sigmoidoscop* OR duodenoscop* OR esophagoscop* OR oesophagoscop* OR gastroscop* OR enteroscop* OR (digestive NEXT scope?) OR (GI NEXT scope?) OR (GI NEXT endoscop*) OR choledochoscop* OR cholangioscop* OR pancreatoscop* OR (capsule NEXT endoscop*)):ti,ab,kw 14573
#3 #1 OR #2 16970
#4 [mh "Gastrointestinal Diseases"/di] OR [mh "Gastrointestinal Diseases"/dg] OR [mh "Gastrointestinal Diseases"/su] 13477
#5 [mh "Gastrointestinal Tract"] 16118
#6 (gastr* OR esophag* OR oesophag* OR "upper GI" OR "lower GI" OR bowel* OR colon OR colonic OR duoden* OR intestin* OR stomach OR "digestive tract" OR ("endoscopic retrograde" NEXT cholangi*) OR ERCP OR ileum OR ileal OR jejun* OR enteric):ti,ab,kw 159212
#7 (endoscop*):ti,ab,kw 34684
#8 [mh ^Endoscopes] OR [mh ^Endoscopy] 2670
#9 #4 OR #5 OR #6 166449
#10 #7 OR #8 34684
#11 #9 AND #10 22448
#12 #3 OR #11 30597
#13 [mh "Equipment Reuse"] 125
#14 (reusable OR reuse* OR reprocessed OR "re usable" OR ("re" NEXT use*) OR "re processed" OR "multiple use" OR "multi use"):ti,ab,kw 1275
#15 #13 OR #14 1275
#16 [mh "Disposable Equipment"] 388
#17 (disposable OR "single use" OR "one time use"):ti,ab,kw 3942
#18 #16 OR #17 3942
#19 [mh ^Environment] OR [mh ^"Carbon Footprint"] 593
#20 [mh ^"Environmental Pollution"] OR [mh ^"Air Pollution"] OR [mh ^"Air Pollution, Indoor"] OR [mh "Environmental Restoration and Remediation"] OR [mh "Water Pollution"] OR [mh "Environmental Pollutants"] 1451
#21 [mh "Climate Change"] OR [mh "Greenhouse Effect"] 51
#22 [mh "Conservation of Natural Resources"] 207
#23 [mh "Waste Products"] OR [mh "Waste Management"] 247
#24 (sustainab*):ti,kw 694
#25 (environment* NEAR/3 (impact* OR conservation OR cost? OR resource?)):ti,ab,kw 794
#26 ("climate change" OR "global warming" OR "greenhouse effect" OR "net zero" OR (climate NEXT impact?) OR (greenhouse NEXT gas*)):ti,ab,kw 289
#27 (carbon NEAR/3 (emission? OR impact? OR neutral* OR footprint)):ti,ab,kw 106
#28 (co2 NEAR/3 (emission? OR impact? OR neutral* OR footprint)):ti,ab,kw 40
#29 (pollut*):ti,ab,kw 2856
#30 (landfill OR (waste NEAR/2 disposa*)):ti,ab,kw 90
#31 (("life cycle" NEXT (assessment* OR analys?s)) OR ("cradle to grave" NEXT analys*) OR "circular economy"):ti,ab,kw 17
#32 (waste NEAR/2 manage*):ti,ab,kw 121
#33 (green* NEAR/2 (endoscopy OR surgery OR gastroenterology)):ti,ab,kw 14
#34 (recycl*):ti,ab,kw 479
#35 #19 OR #20 OR #21 OR #22 OR #23 OR #24 OR #25 OR #26 OR #27 OR #28 OR #29 OR #30 OR #31 OR #32 OR #33 OR #34 6075

#36 #15 OR #18 OR #35 10806
#37 #12 AND #36 with Cochrane Library publication date Between Jul 2023 and Dec 2024 15

Global Index Medicus https://www.globalindexmedicus.net/

Date searched: 04/03/24
Databases: All the Regional Indexes Medici: African Index Medicus (AIM), Index Medicus for the Eastern Mediterranean Region (IMEMR), Index Medicus for the South-East Asia Region (IMSEAR), Latin America and the Caribbean Literature on Health Sciences (LILACS), Western Pacific Region Index Medicus (WPRO)

Search strategy: (note tw fields are Title, Abstract, Subject):
tw:((tw:(colonoscop* OR sigmoidoscop* OR duodenoscop* OR esophagoscop* OR oesophagoscop* OR gastroscop* OR enteroscop* OR "digestive scope" OR "digestive scopes" OR "GI scope" OR "GI scopes" OR "GI endoscope" OR "GI endoscopy" OR "GI endoscopes" OR "GI endoscopies" OR choledochoscop* OR cholangioscop* OR pancreatoscop* OR "capsule endoscope" OR "capsule endoscopes" OR "capsule endoscopy" OR "capsule endoscopies" OR ((gastr* OR esophag* OR oesophag* OR "upper GI" OR "lower GI" OR bowel* OR colon OR colonic OR duoden* OR intestin* OR stomach OR "digestive tract" OR "digestive system" OR "endoscopic retrograde cholangiopancreatography" OR ercp OR ileum OR ileal OR jejun* OR enteric) AND endoscop*))) AND (tw:(reusable OR reuse* OR reprocessed OR "re usable" OR "re use" OR "re used" OR "re processed" OR "multiple use" OR "multi use" OR disposable OR "single use" OR "one time use" OR sustainab* OR "environmental impact" OR "environmental impacts" OR "environmental conservation" OR "environmental cost" OR "environmental costs" OR "environmental resources" OR "natural resources" OR "climate change" OR "global warming" OR "greenhouse effect" OR "net zero" OR "climate impact" OR "climate impacts" OR "greenhouse gas" OR "greenhouse gases" OR "carbon emissions" OR "carbon neutral" OR "carbon neutrality" OR "carbon footprint" OR "co2 emissions" OR pollut* OR landfill OR "waste disposal" OR "disposable waste" OR "disposal of waste" OR "life cycle assessment" OR "life cycle analysis" OR "cradle to grave analysis" OR "circular economy" OR "waste management" OR "green endoscopy" OR "greener endoscopy" OR "green surgery" OR "greener surgery" OR "green gastroenterology" OR "greener gastroenterology" OR recycl*)))
99 results

ClinicalTrials.gov

Date searched: 04/03/24

10 searches run exactly as above.

Total: 323 results
Total after deduplication across searches (using Endnote 21): 200 results

International Clinical Trials Registry Platform (WHO ICTRP)

Date searched: 04/03/24

3 searches run exactly as above.

Total: 196 results
Total after deduplication across searches (using Endnote 21): 179

5. Search strategies: Search update for September 2024

MEDLINE (Ovid)

Ovid MEDLINE(R) ALL <1946 to September 24, 2024>
Date searched: 25/09/24

1 exp Endoscopes, Gastrointestinal/ or exp Endoscopy, Digestive System/ 131759
2 (colonoscop* or sigmoidoscop* or duodenoscop* or esophagoscop* or oesophagoscop* or gastroscop* or enteroscop* or digestive scope? or GI scope? or GI endoscop* or choledochoscop* or cholangioscop* or pancreatoscop* or capsule endoscop*).kf,tw. 66028
3 1 or 2 161077
4 exp Gastrointestinal Diseases/di, dg, su 425645
5 exp Gastrointestinal Tract/ 706661
6 (gastr* or esophag* or oesophag* or upper GI or lower GI or bowel* or colon or colonic or duoden* or intestin* or stomach or digestive tract or endoscopic retrograde cholangi* or ERCP or ileum or ileal or jejun* or enteric).kf,tw. 1710056
7 endoscop*.kf,tw. 268919
8 Endoscopes/ or Endoscopy/ 66136
9 4 or 5 or 6 2082503
10 7 or 8 286668
11 9 and 10 162898
12 3 or 11 [GI endoscopes] 247690
13 Equipment Reuse/ 3256
14 (reusable or reuse* or reprocessed or re usable or re use* or re processed or "multiple use" or "multi use").kf,tw. 41615
15 13 or 14 [MU / reusable] 43267
16 Disposable Equipment/ 5369
17 (disposable or "single use" or "one time use").kf,tw. 22057
18 16 or 17 [SU / disposable] 24303
19 Environment/ or Carbon Footprint/ 70222
20 Environmental Pollution/ or Air Pollution/ or Air Pollution, Indoor/ or exp "Environmental Restoration and Remediation"/ or exp Water Pollution/ or exp Environmental Pollutants/ 468275
21 exp Climate Change/ or exp Greenhouse Effect/ 40224
22 exp "Conservation of Natural Resources"/ 121286
23 (environment* adj3 (impact* or conservation or cost? or resource?)).kf,tw. 55587
24 (climate change or global warming or greenhouse effect or net zero or climate impact? or greenhouse gas*).kf,tw. 95595
25 (carbon adj3 (emission? or impact? or neutral* or footprint)).kf,tw. 17946
26 (co2 adj3 (emission? or impact? or neutral* or footprint)).kf,tw. 9990
27 pollut*.kf,tw. 235829
28 sustainab*.kf,ti. 44655
29 (landfill or (waste adj2 disposa*)).kf,tw. 13198
30 (life cycle assessment* or life cycle analys?s or "cradle to grave analys*" or circular economy).kf,tw. 10880
31 exp Waste Products/ or exp Waste Management/ 167098
32 (waste adj2 manage*).kf,tw. 10661
33 (green* adj2 (endoscopy or surgery or gastroenterology)).kf,tw. 176
34 recycl*.kf,tw. 75861
35 19 or 20 or 21 or 22 or 23 or 24 or 25 or 26 or 27 or 28 or 29 or 30 or 31 or 32 or 33 or 34 [environmental impact] 964930
36 12 and (15 or 18 or 35) [GI endoscopes + reusable or single use or environmental] 1015
37 exp animals/ not humans/ 5261402
38 36 not 37 993
39 (202403* or 202404* or 202405* or 202406* or 202407* or 202408* or 202409*).dt,ez,da,cb. 1034446
40 38 and 39 69

Embase (Ovid)

Embase <1974 to 2024 September 24>
Date searched: 25/09/24

1 exp digestive endoscope/ or exp digestive tract endoscopy/ 289819
2 (colonoscop* or sigmoidoscop* or duodenoscop* or esophagoscop* or oesophagoscop* or gastroscop* or enteroscop* or digestive scope? or GI scope? or GI endoscop* or choledochoscop* or cholangioscop* or pancreatoscop* or capsule endoscop*).kf,tw. 124245
3 1 or 2 307836
4 exp digestive system disease/di, dm, su or exp digestive system/ 2259196
5 (gastr* or esophag* or oesophag* or upper GI or lower GI or bowel* or colon or colonic or duoden* or intestin* or stomach or digestive tract or endoscopic retrograde cholangi* or ERCP or ileum or ileal or jejun* or enteric).kf,tw. 2238410
6 4 or 5 3519399
7 endoscop*.kf,tw. 436873
8 endoscope/ or endoscopy/ 157084
9 7 or 8 476184
10 6 and 9 310822
11 3 or 10 [digestive system endoscopes] 477639
12 recycling/ 36162
13 (reusable or reuse* or reprocessed or re usable or re use* or re processed or "multiple use" or "multi use").kf,tw. 51149
14 12 or 13 [MU / reusable] 82179
15 disposable equipment/ 11815
16 (disposable or "single use" or "one time use").kf,tw. 29868
17 15 or 16 [SU / disposable] 34245
18 environmental economics/ or exp environmental impact/ or environmental impact assessment/ or life cycle assessment/ 137762
19 pollution/ or air pollution/ or exp greenhouse gas emission/ or indoor air pollution/ or exp nitrogen oxide emission/ or ozone depletion/ or exp plastic pollution/ or water pollution/ or acid rain/ or sea pollution/ or water contamination/ 242675
20 pollutant/ or air pollutant/ or exp atmospheric particulate matter/ or greenhouse gas/ or persistent organic pollutant/ or water pollutant/ 149311
21 waste/ or chemical waste/ or gas waste/ or packaging waste/ or exp hospital waste/ or exp plastic waste/ or solid waste/ or wastewater/ or hospital wastewater/ 44685
22 climate change/ 69839
23 environmental protection/ or energy conservation/ or environmental sustainability/ or water conservation/ 71378
24 (environment* adj3 (impact* or conservation or cost? or resource?)).kf,tw. 63030
25 (climate change or global warming or greenhouse effect or net zero or climate impact? or greenhouse gas*).kf,tw. 93790
26 (carbon adj3 (emission? or impact? or neutral* or footprint)).kf,tw. 18224
27 (co2 adj3 (emission? or impact? or neutral* or footprint)).kf,tw. 10607
28 pollut*.kf,tw. 284529
29 sustainab*.kf,ti. 45043
30 (landfill or (waste adj2 disposa*)).kf,tw. 17776
31 (life cycle assessment* or life cycle analys?s or "cradle to grave analys*" or circular economy).kf,tw. 13385
32 (waste adj2 manage*).kf,tw. 14695
33 waste management/ or waste minimization/ 27878
34 (green* adj2 (endoscopy or surgery or gastroenterology)).kf,tw. 237
35 recycl*.kf,tw. 87841
36 18 or 19 or 20 or 21 or 22 or 23 or 24 or 25 or 26 or 27 or 28 or 29 or 30 or 31 or 32 or 33 or 34 or 35 877820
37 11 and (14 or 17 or 36) 2179
38 (exp animal/ or exp animal experiment/) not (exp human/ or exp human experiment/ or conference abstract.pt.) 5188564
39 37 not 38 2137
40 limit 39 to dc=20240304-20241231 163

Web of Science Core Collection

Date searched: 25/09/24

Editions: Science Citation Index Expanded (SCI-EXPANDED) 1970-present, Social Sciences Citation Index (SSCI) 1900-present, Conference Proceedings Citation Index – Science (CPCI-S) 1990-present, Conference Proceedings Citation Index – Social Science & Humanities (CPCI-SSH) 1990-present, Emerging Sources Citation Index (ESCI) 2015-present

#	Search Query	Results
1	TS=(colonoscop* OR sigmoidoscop* OR duodenoscop* OR esophagoscop* OR oesophagoscop* OR gastroscop* OR enteroscop* OR "digestive scope$" OR "GI scope$" OR "GI endoscop*" OR choledochoscop* OR cholangioscop* OR pancreatoscop* OR "capsule endoscop*")	80889
2	TS=(gastr* OR esophag* OR oesophag* OR "upper GI" OR "lower GI" OR bowel* OR colon OR colonic OR duoden* OR intestin* OR stomach OR "digestive tract" OR "endoscopic retrograde cholangi*" OR ERCP OR ileum OR ileal OR jejun* OR enteric)	1998912
3	TS=(endoscop*)	302610
4	#2 AND #3	160276
5	#1 OR #4	211422
6	TS=(recycl*) Editions: WOS.SCI,WOS.SSCI,WOS.ISTP,WOS.ISSHP,WOS.ESCI	278602
7	TS=(green* NEAR/1 (endoscopy OR surgery OR gastroenterology)) Editions: WOS.SCI,WOS.SSCI,WOS.ISTP,WOS.ISSHP,WOS.ESCI	170
8	TS=(waste NEAR/1 manage*) Editions: WOS.SCI,WOS.SSCI,WOS.ISTP,WOS.ISSHP,WOS.ESCI	43985
9	TS=("life cycle assessment*" OR "life cycle analys$s" OR "cradle to grave analys*" OR "circular economy") Editions: WOS.SCI,WOS.SSCI,WOS.ISTP,WOS.ISSHP,WOS.ESCI	72932
10	TS=(landfill OR (waste NEAR/1 disposa*)) Editions: WOS.SCI,WOS.SSCI,WOS.ISTP,WOS.ISSHP,WOS.ESCI	62665
11	TS=(pollut*) Editions: WOS.SCI,WOS.SSCI,WOS.ISTP,WOS.ISSHP,WOS.ESCI	703162
12	TS=(co2 NEAR/2 (emission$ OR impact$ OR neutral* OR footprint)) Editions: WOS.SCI,WOS.SSCI,WOS.ISTP,WOS.ISSHP,WOS.ESCI	88487
13	TS=(carbon NEAR/2 (emission$ OR impact$ OR neutral* OR footprint)) Editions: WOS.SCI,WOS.SSCI,WOS.ISTP,WOS.ISSHP,WOS.ESCI	118121
14	TS=("climate change" OR "global warming" OR "greenhouse effect" OR "net zero" OR "climate impact$" OR "greenhouse gas*") Editions: WOS.SCI,WOS.SSCI,WOS.ISTP,WOS.ISSHP,WOS.ESCI	546021
15	TS=(environment* NEAR/2 (impact* OR conservation OR cost$ OR resource$)) Editions: WOS.SCI,WOS.SSCI,WOS.ISTP,WOS.ISSHP,WOS.ESCI	242066
16	((TI=(sustainab*)) OR AK=(sustainab*)) OR KP=(sustainab*) Editions: WOS.SCI,WOS.SSCI,WOS.ISTP,WOS.ISSHP,WOS.ESCI	365959
17	#6 OR #7 OR #8 OR #9 OR #10 OR #11 OR #12 OR #13 OR #14 OR #15 OR #16 Editions: WOS.SCI,WOS.SSCI,WOS.ISTP,WOS.ISSHP,WOS.ESCI	2052496
18	#5 AND #17 Editions: WOS.SCI,WOS.SSCI,WOS.ISTP,WOS.ISSHP,WOS.ESCI	198
19	TS=(reusable OR reuse* OR reprocessed OR "re usable" OR "re use*" OR "re processed" OR "multiple use" OR "multi use")	212299
20	TS=(disposable OR "single use" OR "one time use")	34953
21	#19 OR #20	243793
22	#5 AND #21	1016

23	#18 OR #22 Editions: WOS.SCI,WOS.SSCI,WOS.ISTP,WOS.ISSHP,WOS.ESCI Timespan: 2024-03-04 to 2024-12-31	95

Cochrane Central Register of Controlled Trials and Cochrane Database of Systematic Reviews (Cochrane Library, Wiley)

Date searched: 25/09/24
Search Screen: Advanced / search manager https://www.cochranelibrary.com/advanced-search/search-manager

ID Search Hits
#1 [mh "Endoscopes, Gastrointestinal"] OR [mh "Endoscopy, Digestive System"] 7774
#2 (colonoscop* OR sigmoidoscop* OR duodenoscop* OR esophagoscop* OR oesophagoscop* OR gastroscop* OR enteroscop* OR (digestive NEXT scope?) OR (GI NEXT scope?) OR (GI NEXT endoscop*) OR choledochoscop* OR cholangioscop* OR pancreatoscop* OR (capsule NEXT endoscop*)):ti,ab,kw 15146
#3 #1 OR #2 17572
#4 [mh "Gastrointestinal Diseases"/di] OR [mh "Gastrointestinal Diseases"/dg] OR [mh "Gastrointestinal Diseases"/su] 13696
#5 [mh "Gastrointestinal Tract"] 16285
#6 (gastr* OR esophag* OR oesophag* OR "upper GI" OR "lower GI" OR bowel* OR colon OR colonic OR duoden* OR intestin* OR stomach OR "digestive tract" OR ("endoscopic retrograde" NEXT cholangi*) OR ERCP OR ileum OR ileal OR jejun* OR enteric):ti,ab,kw 164823
#7 (endoscop*):ti,ab,kw 36072
#8 [mh ^Endoscopes] OR [mh ^Endoscopy] 2699
#9 #4 OR #5 OR #6 172205
#10 #7 OR #8 36072
#11 #9 AND #10 23282
#12 #3 OR #11 31769
#13 [mh "Equipment Reuse"] 126
#14 (reusable OR reuse* OR reprocessed OR "re usable" OR ("re" NEXT use*) OR "re processed" OR "multiple use" OR "multi use"):ti,ab,kw 1332
#15 #13 OR #14 1332
#16 [mh "Disposable Equipment"] 392
#17 (disposable OR "single use" OR "one time use"):ti,ab,kw 4123
#18 #16 OR #17 4123
#19 [mh ^Environment] OR [mh ^"Carbon Footprint"] 604
#20 [mh ^"Environmental Pollution"] OR [mh ^"Air Pollution"] OR [mh ^"Air Pollution, Indoor"] OR [mh "Environmental Restoration and Remediation"] OR [mh "Water Pollution"] OR [mh "Environmental Pollutants"] 1472
#21 [mh "Climate Change"] OR [mh "Greenhouse Effect"] 55
#22 [mh "Conservation of Natural Resources"] 210
#23 [mh "Waste Products"] OR [mh "Waste Management"] 249
#24 (sustainab*):ti,kw 762
#25 (environment* NEAR/3 (impact* OR conservation OR cost? OR resource?)):ti,ab,kw 858
#26 ("climate change" OR "global warming" OR "greenhouse effect" OR "net zero" OR (climate NEXT impact?) OR (greenhouse NEXT gas*)):ti,ab,kw 322
#27 (carbon NEAR/3 (emission? OR impact? OR neutral* OR footprint)):ti,ab,kw 125
#28 (co2 NEAR/3 (emission? OR impact? OR neutral* OR footprint)):ti,ab,kw 43
#29 (pollut*):ti,ab,kw 2926
#30 (landfill OR (waste NEAR/2 disposa*)):ti,ab,kw 92
#31 (("life cycle" NEXT (assessment* OR analys?s)) OR ("cradle to grave" NEXT analys*) OR "circular economy"):ti,ab,kw 18
#32 (waste NEAR/2 manage*):ti,ab,kw 126
#33 (green* NEAR/2 (endoscopy OR surgery OR gastroenterology)):ti,ab,kw 16
#34 (recycl*):ti,ab,kw 490
#35 #19 OR #20 OR #21 OR #22 OR #23 OR #24 OR #25 OR #26 OR #27 OR #28 OR #29 OR #30 OR #31 OR #32 OR #33 OR #34 6332
#36 #15 OR #18 OR #35 11280

#37 #12 AND #36 with Cochrane Library publication date Between Mar 2024 and Dec 2024 19 [all CENTRAL]

Global Index Medicus https://www.globalindexmedicus.net/

Date searched: 26/09/24
Databases: All the Regional Indexes Medici: African Index Medicus (AIM), Index Medicus for the Eastern Mediterranean Region (IMEMR), Index Medicus for the South-East Asia Region (IMSEAR), Latin America and the Caribbean Literature on Health Sciences (LILACS), Western Pacific Region Index Medicus (WPRO)

Search strategy: (note tw fields are Title, Abstract, Subject):
tw:((tw:(colonoscop* OR sigmoidoscop* OR duodenoscop* OR esophagoscop* OR oesophagoscop* OR gastroscop* OR enteroscop* OR "digestive scope" OR "digestive scopes" OR "GI scope" OR "GI scopes" OR "GI endoscope" OR "GI endoscopy" OR "GI endoscopes" OR "GI endoscopies" OR choledochoscop* OR cholangioscop* OR pancreatoscop* OR "capsule endoscope" OR "capsule endoscopes" OR "capsule endoscopy" OR "capsule endoscopies" OR ((gastr* OR esophag* OR oesophag* OR "upper GI" OR "lower GI" OR bowel* OR colon OR colonic OR duoden* OR intestin* OR stomach OR "digestive tract" OR "digestive system" OR "endoscopic retrograde cholangiopancreatography" OR ercp OR ileum OR ileal OR jejun* OR enteric) AND endoscop*))) AND (tw:(reusable OR reuse* OR reprocessed OR "re usable" OR "re use" OR "re used" OR "re processed" OR "multiple use" OR "multi use" OR disposable OR "single use" OR "one time use" OR sustainab* OR "environmental impact" OR "environmental impacts" OR "environmental conservation" OR "environmental cost" OR "environmental costs" OR "environmental resources" OR "natural resources" OR "climate change" OR "global warming" OR "greenhouse effect" OR "net zero" OR "climate impact" OR "climate impacts" OR "greenhouse gas" OR "greenhouse gases" OR "carbon emissions" OR "carbon neutral" OR "carbon neutrality" OR "carbon footprint" OR "co2 emissions" OR pollut* OR landfill OR "waste disposal" OR "disposable waste" OR "disposal of waste" OR "life cycle assessment" OR "life cycle analysis" OR "cradle to grave analysis" OR "circular economy" OR "waste management" OR "green endoscopy" OR "greener endoscopy" OR "green surgery" OR "greener surgery" OR "green gastroenterology" OR "greener gastroenterology" OR recycl*)))
103 results

ClinicalTrials.gov https://clinicaltrials.gov/

Date searched: 26/09/24

10 searches run exactly as above.

Total: 350 results
Total after deduplication across searches (using Endnote 21): 216 results

International Clinical Trials Registry Platform (WHO ICTRP)

Date searched: 26/09/24

3 searches run exactly as above.

Total: 219 results
Total after deduplication across searches (using Endnote 21): 200
